# AI Promoted Virtual Screening, Structure-Based Hit Optimization, and Synthesis of Novel COVID-19 S-RBD Domain Inhibitors

**DOI:** 10.1021/acs.jcim.4c01110

**Published:** 2024-11-13

**Authors:** Ioannis Gkekas, Sotirios Katsamakas, Stelios Mylonas, Theano Fotopoulou, George Ε. Magoulas, Alia Cristina Tenchiu, Marios Dimitriou, Apostolos Axenopoulos, Nafsika Rossopoulou, Simona Kostova, Erich E. Wanker, Theodora Katsila, Demetris Papahatjis, Vassilis G. Gorgoulis, Maria Koufaki, Ioannis Karakasiliotis, Theodora Calogeropoulou, Petros Daras, Spyros Petrakis

**Affiliations:** † Institute of Applied Biosciences, Centre for Research and Technology Hellas, Thessaloniki 57001, Greece; ‡ Information Technologies Institute, Centre for Research and Technology Hellas, Thessaloniki 57001, Greece; § Institute of Chemical Biology, National Hellenic Research Foundation, 48 Vassileos Constantinou Avenue, Athens 11635, Greece; ∥ Laboratory of Biology, Department of Medicine, Democritus University of Thrace, Alexandroupolis 68100, Greece; ⊥ Max-Delbrueck-Center for Molecular Medicine in the Helmholtz Association, Berlin 13125, Germany; # Molecular Carcinogenesis Group, Department of Histology and Embryology, Medical School, National and Kapodistrian University of Athens, Athens 11635, Greece; ∇ Ninewells Hospital and Medical School, University of Dundee, DD19SY Dundee, U.K.

## Abstract

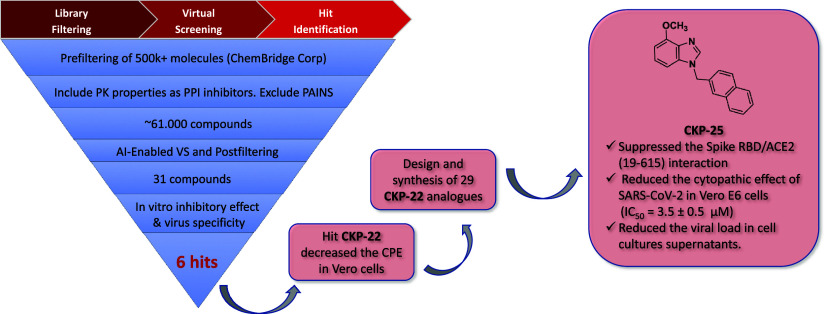

Coronavirus disease 2019 (COVID-19) is caused by a new, highly pathogenic severe-acute-respiratory syndrome coronavirus 2 (SARS-CoV-2) that infects human cells through its transmembrane spike (S) glycoprotein. The receptor-binding domain (RBD) of the S protein interacts with the angiotensin-converting enzyme II (ACE2) receptor of the host cells. Therefore, pharmacological targeting of this interaction might prevent infection or spread of the virus. Here, we performed a virtual screening to identify small molecules that block S-ACE2 interaction. Large compound libraries were filtered for drug-like properties, promiscuity and protein–protein interaction-targeting ability based on their ADME-Tox descriptors and also to exclude pan-assay interfering compounds. A properly designed AI-based virtual screening pipeline was applied to the remaining compounds, comprising approximately 10% of the starting data sets, searching for molecules that could bind to the RBD of the S protein. All molecules were sorted according to their screening score, grouped based on their structure and postfiltered for possible interaction patterns with the ACE2 receptor, yielding 31 hits. These hit molecules were further tested for their inhibitory effect on Spike RBD/ACE2 (19–615) interaction. Six compounds inhibited the S-ACE2 interaction in a dose-dependent manner while two of them also prevented infection of human cells from a pseudotyped virus whose entry is mediated by the S protein of SARS-CoV-2. Of the two compounds, the benzimidazole derivative **CKP-22** protected Vero E6 cells from infection with SARS-CoV-2, as well. Subsequent, hit-to-lead optimization of **CKP-22** was effected through the synthesis of 29 new derivatives of which compound **CKP-25** suppressed the Spike RBD/ACE2 (19–615) interaction, reduced the cytopathic effect of SARS-CoV-2 in Vero E6 cells (IC_50_ = 3.5 μM) and reduced the viral load in cell culture supernatants. Early in vitro ADME-Tox studies showed that **CKP-25** does not possess biodegradation or liver metabolism issues, while isozyme-specific CYP450 experiments revealed that **CKP-25** was a weak inhibitor of the CYP450 system. Moreover, **CKP-25** does not elicit mutagenic effect on *Escherichia coli* WP2 uvrA strain. Thus, **CKP-25** is considered a lead compound against COVID-19 infection.

## Introduction

In March 2020, the coronavirus disease 2019 (COVID-19) caused by a novel, highly pathogenic severe-acute-respiratory syndrome coronavirus 2 (SARS-CoV-2) was declared a global pandemic by the World Health Organization (WHO).^1^ COVID-19 is characterized as a serious respiratory disease that affects also the gastrointestinal system, heart, central nervous system and kidneys, with 771,679,618 confirmed cases, including 6,977,023 deaths globally, reported to WHO until November 2023.^2^ The accelerated development of effective COVID-19 vaccines and other therapeutics has offered transient solutions to resolve the pandemic.^3,4^ However, the SARS-CoV-2 statistical data for the period of January 2020–January 2022 show a rapid evolution with a 4-fold increase in daily cases worldwide,^5^ affirming that mankind is far from eradicating this pandemic.

SARS-CoV-2 belongs to the β-genus of the coronaviruses and its genome comprises 14 open reading frames encoding 16 non-structural proteins (nsp1–16), nine accessory proteins and four structural proteins [spike (S), envelope (E), membrane (M) and nucleocapsid (N)].^6^ The human infection by SARS-CoV-2 involves the binding of the transmembrane homotrimeric spike (S) glycoprotein through its receptor binding domain (RBD) of its S1 subunit to the host protein angiotensin-converting enzyme 2 (ACE2). Then, the S2 subunit of spike fuses the host and viral membranes, enabling the entry of SARS CoV-2 genome into human cells. In addition, the transmembrane serine protease 2 (TMPRSS2), acting as major host protease is required for the priming of the S protein. During the intracellular life cycle, the virus expresses and replicates its genome RNA leading to the production of coding proteins. Then, the virion is assembled and the newly synthesized SARS-CoV-2 particles are released, surrounded by the membrane of the host cells.^7−11^ Therefore, the prevention of the viral entry step represents the first point of interfering with viral transmission.

Since the beginning of the pandemic, drug repurposing^12^ was used as a first option for the discovery of new therapeutics, due to shorter clinical trials, reduced risks and lower cost for development. Thus, the combination of in silico and biological strategies allowed the effective and fast selection of promising repurposed candidates for COVID-19 treatment.^13^ Remdesivir, originally developed for the treatment of Ebola is a repurposed inhibitor of the SARS-CoV-2 RNA-dependent RNA polymerase (RdRp) and was the first drug authorized by the FDA against COVID-19.^14^ Other available treatment options for adults with COVID-19, involving repurposed drugs are Lagevrio^15,16^ and Paxlovid.^17^ Although drug repurposing may have several advantages, contrariwise it has some non-negligible disadvantages like dosing safety, specificity and delivery capability.^18^ Recently, Ensitrelvir fumaric acid a 3C-like protease inhibitor developed through joint research between Hokkaido University and Shionogi Ltd. received emergency approval in Japan in November 2022 (Xocova).^19^ In April 2023 the FDA has granted Fast Track designation for Ensitrelvir fumaric acid, while the NIH has initiated a multisite clinical trial for the evaluation in adults hospitalized with COVID-19.^20^

The spike glycoprotein is the antigen used in many COVID-19 vaccines^21^ while the specific interaction of S(RBD) with hACE2 is the current pharmacological target of therapeutic antibodies.^22,23^ However, it is subgenus and strain specific, and thus promotes the emergence of new mutated variants that in turn, result in decreased efficiency of vaccines and neutralizing antibodies.^24−30^ Furthermore, recently published data highlight that vaccine effectiveness declines significantly between three and six months post-vaccination.^31^ Moreover, there is a significant percentage of the population who for a variety of reasons refuse to or cannot be vaccinated.^32^

The interaction between S(RBD) and hACE2 is of outmost importance regulating the cross-species and human-to-human SARS-CoV-2 transmission.^33^ Hence developing drugs that could disrupt the RBD-ACE2 interaction is of great interest and significance. Despite the difficulties in inhibiting protein–protein interactions (PPIs) with small molecules, they are more advantageous than antibodies. These molecules are less sensitive to different viral strains and mutations, can be administered as oral or inhalable formulations and result in lower immunogenic side effects.^34^ Several in silico-based and in vitro screening approaches have been employed to identify potential small molecule and peptide-based inhibitors of the S(RBD)/ACE2 interaction.^34−58^ Despite the absence of potent in vitro and in vivo inhibitors of this interaction to date, these results support the feasibility of targeting this PPI by small molecules.

In the current manuscript we performed an AI-enabled virtual screening to identify compounds which would block the S(RBD)-ACE2 interaction. The identified hits were validated experimentally and the most potent (**CKP-22**) was optimized through structure-based drug design (SBDD) followed by the synthesis of the proposed derivatives. The new compounds were evaluated for the inhibition of S(RBD)/ACE2 interaction and reduction of the cytopathic effect of SARS-CoV-2 in Vero E6 cells. The most potent were further assessed for preventing infection from the original SARS-CoV-2 in vitro.

## Results and Discussion

### AI-Enabled Virtual Screening and Design

**Figure 1:**
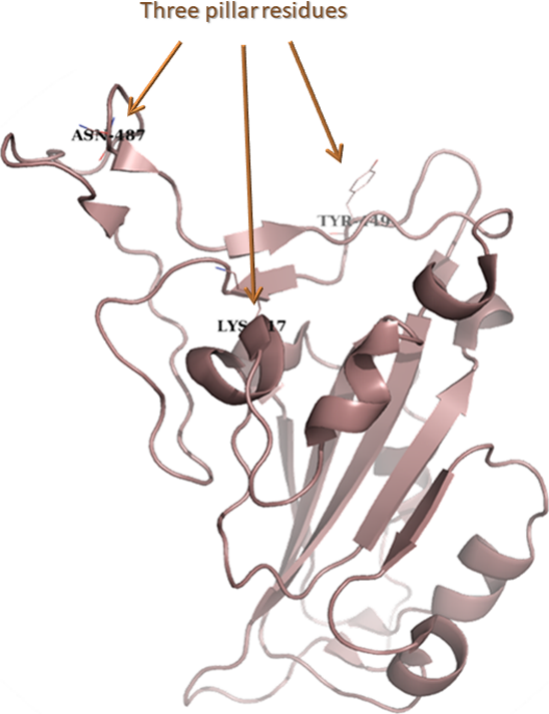
S(RBD) illustration as brown cartoon with the three pillar residues shown as sticks and labeled.

Shortly after the emergence of the COVID-19 pandemic in 2020 the crystal structure of the S(RBD)-ACE2 complex became available.^10^ This shed light on important RBD domain structural features like the interface definition enclosed by the amino acids Lys417, Tyr449 and Asn487. These three pillar residues are of high significance for ACE2 binding (Figure 1).

The lysine residue is the only amino acid sequentially belonging outside the receptor binding motif (RBM) but is recognized as highly important for the RBD-ACE2 complex formation with the contribution of two salt bridges and an equal number of hydrogen bonds. In contrast, the equivalent valine residue found in the RBD domain of SARS-CoV-1 fails to participate in the ACE2 binding interaction network, possessing a 9-fold difference in the dissociation constant.

Inspired by the example of the development of the antiviral agent Fostemsavir as a protein–protein interaction drug (PPID)^59^ we set out to identify through a Virtual-Screening (VS) campaign potent PPI compounds that could disrupt the formation of the S(RBD)-ACE2 complex.

**Figure 2:**
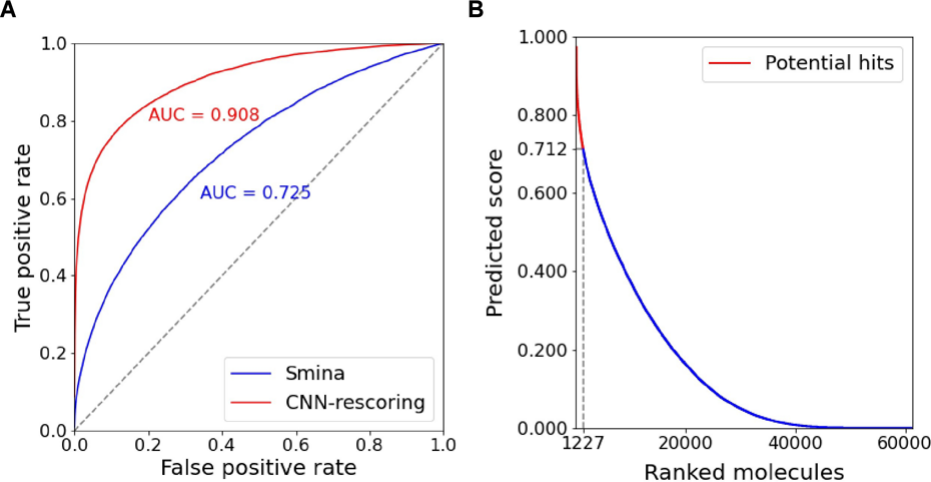
Virtual screening. (A) Comparison between the adopted CNN-rescoring approach and Smina on the DUD-E benchmark data set. (B) Molecule ranking scores after applying our VS pipeline on the initially filtered library (∼60K molecules) and selection of the top-2% (1227).

Our team has been working on the training of a new AI-enabled pipeline^60^ combining the docking result of Smina^61^ with an AI-based rescoring function that is able to distinguish more efficiently potential binding complexes.^60^ To alleviate possible inconsistencies originating from the unknown 3D structure of the input compounds used for screening, the docking step is applied on two different conformations of these compounds, created by the OpenBabel toolbox,^62^ while 50 docked poses are generated for each compound conformation. The AI-enabled pipeline progresses in three distinct steps. First, the resulted protein–substrate solutions are rescored with our custom AI-based rescoring function,^60^ which comprises a 3D convolutional neural network (CNN) that was trained on the DUD-E benchmark^63^ with the aim to automatically capture the underlying mechanism responsible for the protein–ligand binding. The 103 proteins present in the DUD-E data set were split to algebraic 3 folds based on their sequence similarity, and 3-fold training was performed for hyperparameters selection (Figure 2A). A subsequent retraining on the whole DUD-E data set provided the final model which is used to rescore the predicted protein-compound complexes. Second, in an attempt to further enhance the CNN performance, a fusion approach is applied which combines under a unified scheme the AI-scores with the Smina predicted affinities, taking into account both conformations and multiple docked poses for each compound. The third and final step comprises cluster grouping of the potential hits based on their structural similarity utilizing a 3D shape descriptor^64^ to ensure diversity among them.

With this newly trained AI promoted algorithm in hand we performed a virtual screening (VS) approach on the complete RBD surface against three commercial libraries (i.e., macrocycle, EXPRESS-Pick Stock & Coronavirus dedicated libraries) from ChemBridge Corporation, counting more than half a million compounds in total. All libraries were filtered prior to their use according to a drug-like subset of pharmacokinetic (PK) properties to exclude non-favorable compounds from the process and minimize the load. Filtering included descriptors like Lipinski’s rule of 5,^65^ Veber^66^ and Egan^66^ bioavailability scores. The two first take into consideration the number of rotatable bonds and tPSA, while Egan correlates tPSA with lipophilicity. Additional filters included phospholipidosis,^67^ GSK 4/400 rule,^67^ the Pfizer 3/75 rule^68^ and Lilly Medchem rule predictions.^69^ The latter can identify potential interference with biological assays, such as substrate chemical reactivity, instability and/or lack of druggability. In detail, the libraries were filtered utilizing FAF-drugs4^70^ to eliminate pan-assay interfering compounds (PAINS) and unwanted metabolites and, afterward, the Eli Lilly MedChem set of rules^69^ along with favorable PPI profile were applied.^71^ PPIs peculiar features do not match traditional binding pockets which have in general larger, flatter and more hydrophobic surfaces. Hence, they bind to narrow PK windows for several descriptors such as molecular weight around 421, log *P* values in the range of 3.56 and topological polar surface area (tPSA) of 89 Å^2^. These values are higher than usual binding pocket properties encountered in drugs.^72^ The above filtering resulted in the selection of 61,349 compounds that met the required criteria and acted as our screening library for the upcoming VS stage.

**Figure 3:**
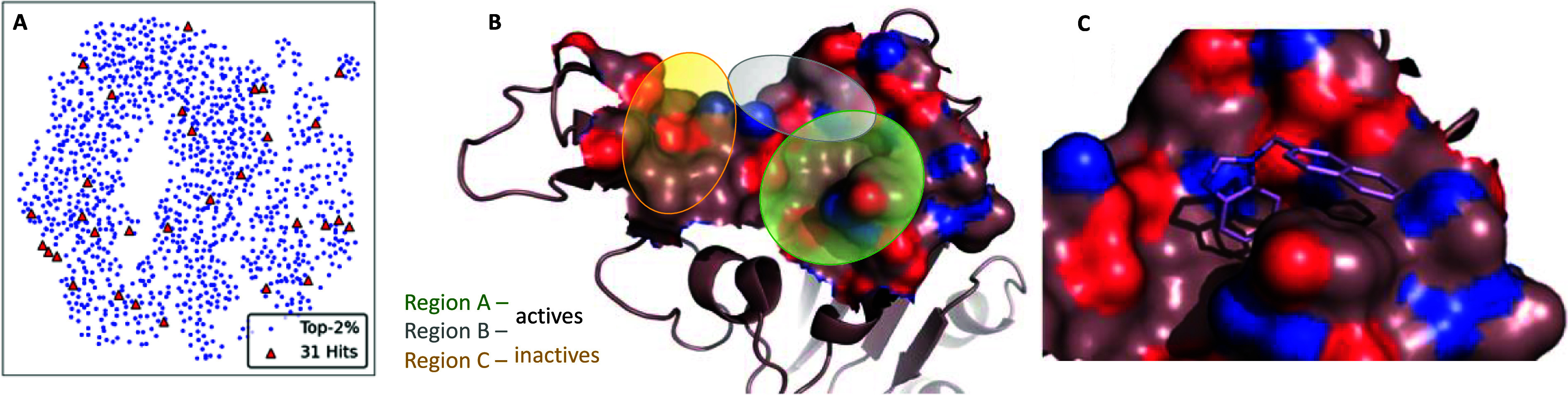
(A) UMAP visualization of the finally selected hits (*n* = 31) compared to the top-2%, on a reduced feature space of shape descriptors. (B) Crystal structure of wt-Spike protein (PDB ID: 6M0J) colored in brown as surface representation for the RBD domain. (C) Docking solution of hit compound **CKP-22** (Chembridge code: 5979349) shown as magenta-colored sticks residing in region A of the RBD domain.

In the VS step as target protein we used the crystal structure of the spike protein S1 subunit bound to the extracellular domain of the ACE2 receptor (PDB ID: 6M0J), solved by cryo-EM at a 2.45 Å resolution.^10^ From this complex, the coronavirus’s spike protein was cleaned, and blind docking was applied on the RBD domain of the S-protein with the ACE2. Our CNN scoring scheme assigned to the vast majority of the screened compounds a very low (near zero) score index, and only few of them were distinguished (Figure 2B). Furthermore, we decided to qualify as potential hits the top-2% which corresponds to 1,227 compounds with a unified score higher than 0.712. The structural-oriented grouping generated 248 clusters which were subsequently subjected to a secondary filtering applying a cutoff score above 0.85 of accepted compounds based on expert decision-making, including visualization of VS solutions. This revealed a scattering of all compounds around three distinct regions on the RBD domain (Figure 3), centering around the previously described pillar residues important for the ACE2 interactions. Hence, this post-filtering step resulted in the selection of the final 31 potential hits (see Table S1) that are quite diverse in shape (Figure 3A) and representatively cover all highlighted RBD regions (Figure 3B). Their biological evaluation, as described below (see Biological Evaluation), revealed that compounds fitting Region C are inactive, while compounds residing solely in Region A or dually in Regions A and B showed inhibitory activity (Figure 3B). Regions A and B are formed from the amino acids Arg403, Glu406, Lys417, Tyr449, Tyr453, Leu455, Gln493, Tyr495, Gly496, Phe497, Gln498, Asn501, Tyr505 and Gln506.

Among the 31 evaluated hit compounds six effectively blocked the S-ACE2 interaction and two prevented the infection of human cells from a pseudotyped virus whose entry is mediated by the S protein of SARS-CoV-2. In particular, the 1-substituted benzimidazole derivative Chembridge code: 5979349 (**CKP-22**) strongly prevented infection from the original SARS-CoV-2 in vitro at a 50 μΜ concentration. Docking of **CKP-22** revealed that the only witnessed interaction with the protein was a pi-pi stacking between Tyr505 residue and the naphthyl substituent (Figure 3C).

**Figure 4:**
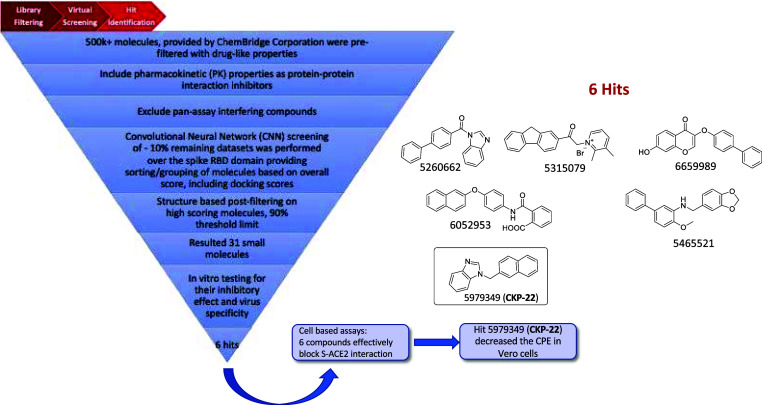
Virtual screening workflow, structures of identified hit compounds inhibiting S(RBD)-ACE2 interaction in a dose-dependent manner and of inhibitor of SARS-CoV-2 entry hit 5979349 (**CKP-22**).

A summary of the virtual screening workflow leading to the hit compound **CKP-22** is presented in Figure 4.

The benzimidazole privileged scaffold has been extensively explored among other heterocycles in drug discovery.^73,74^ In particular, compounds possessing the benzimidazole moiety were previously identified through VS as binders at RBD/ACE2 interface.^75^ Furthermore, Mudi et al. synthesized 5-membered heterocycle-substituted benzimidazole derivatives with in vitro anti SARS-CoV-2 activity. Molecular docking and MD simulations proposed that this activity is mediated by interaction with main protease (M^pro^) and non-structural proteins.^76,77^

**Figure 5:**
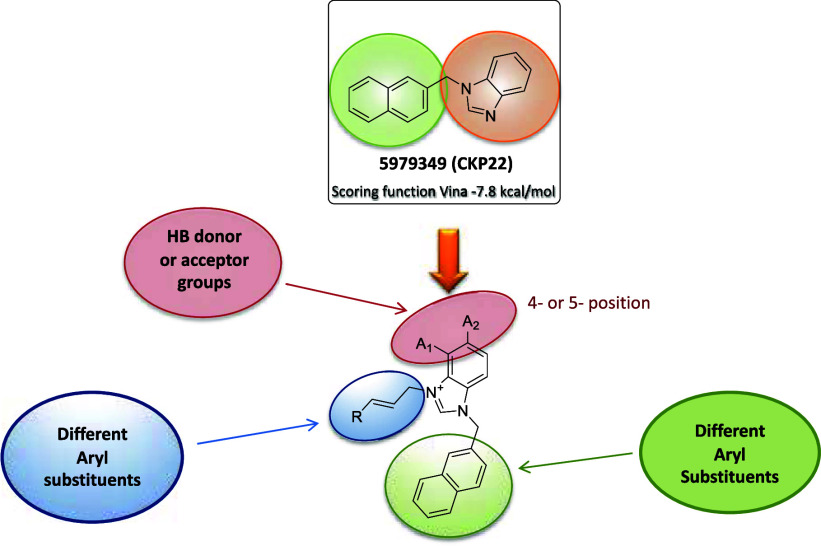
Design strategy of 5979349 (**CKP-22**) derivatives.

Following our validation of **CKP-22** as an anti-SARS-CoV-2 compound inhibiting RBD/ACE2 interaction, a SBDD approach was implemented in order to introduce additional interactions with the S-protein and increase potency. As summarized in Figure 5, we varied the substituents at positions C-4 and C-5 and incorporated different aryls replacing the naphthyl group at *N*^1^ in **CKP-22.** In an effort to extend the interaction network toward region B of the RBD domain we introduced substituents on *N*^3^ bearing a trans vinyl group stereochemistry in order to reach suitable residues (i.e., Tyr449) of this site.

**Figure 6:**
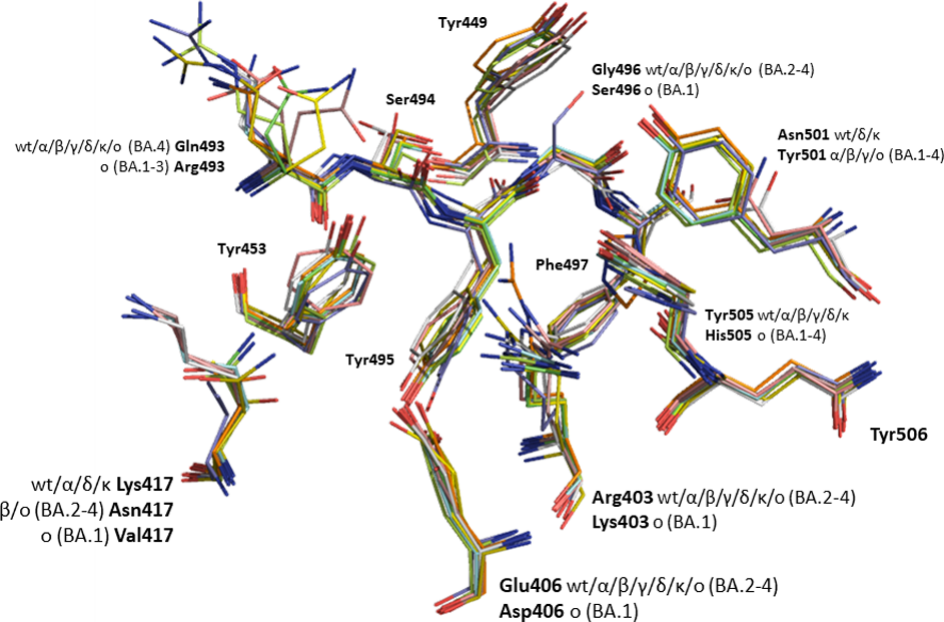
Overlay of COVID-19 line representation for S1 spike glycoprotein residues consisting described regions A and B for wt (PDB entry 6m0j), alpha (PDB entry 7ekf), beta (PDB entry 7ekg), gamma (PDB entry 7ekc), delta (PDB entry 7wbq), kappa (PDB entry 7tez), omicron BA.1 (PDB entry 7u0n), omicron BA.2 (PDB entry 7xo9), omicron BA.3 (PDB entry 7xb1) and omicron BA.4/5 (PDB entry 7zxu).

The S-protein has been continuously and rapidly mutating providing numerous variants during the evolution of the COVID-19 pandemic. However, the three pillar residues were largely maintained across variants. Our design aiming to keep the Tyr505 interaction proved crucial since this residue was one of the most conserved. The only alteration was found in the omicron variant mutations where Tyr505 was replaced by a histidine residue. Moreover, when comparing the wt with other variants it was found that Tyr449 and Asn487 are conserved, while the 417 amino acid is replaced by a Asn in beta,^78^ Thr in gamma,^78^ delta^79^ and kappa^80^ variants maintaining wt relevance, Val in omicron BA.1 variant^81^ and a Asn mutation for BA.2/3/4/5.^82−84^ All the above represent targeting/interacting residues based in our docking studies. Taking into account the epidemiological manifestation of each variant it can be understood that the most severe forms among the variants had Lys417 and Tyr505 present. On regard to regions A and B identified from our study mutation-wise (Figure 6 and Table S2), wt exhibits no difference with delta and kappa variants, in contrary to alpha, beta and kappa variants in which the Asn501 mutated to a tyrosine residue (i.e., N501Y). Omicron variants were the first to swap changes vastly on the RBD domain with the most important being the Q493R in BA.1/2/3, G496S only in BA.1, N501Y and Y505H.

Uniformly, in all docking experiments using the different variants **CKP-22** and its synthetic derivatives showed no difference in their binding mode since the mutated amino acids maintained the same or similar chemical nature.

### Chemistry

**Scheme 1:**
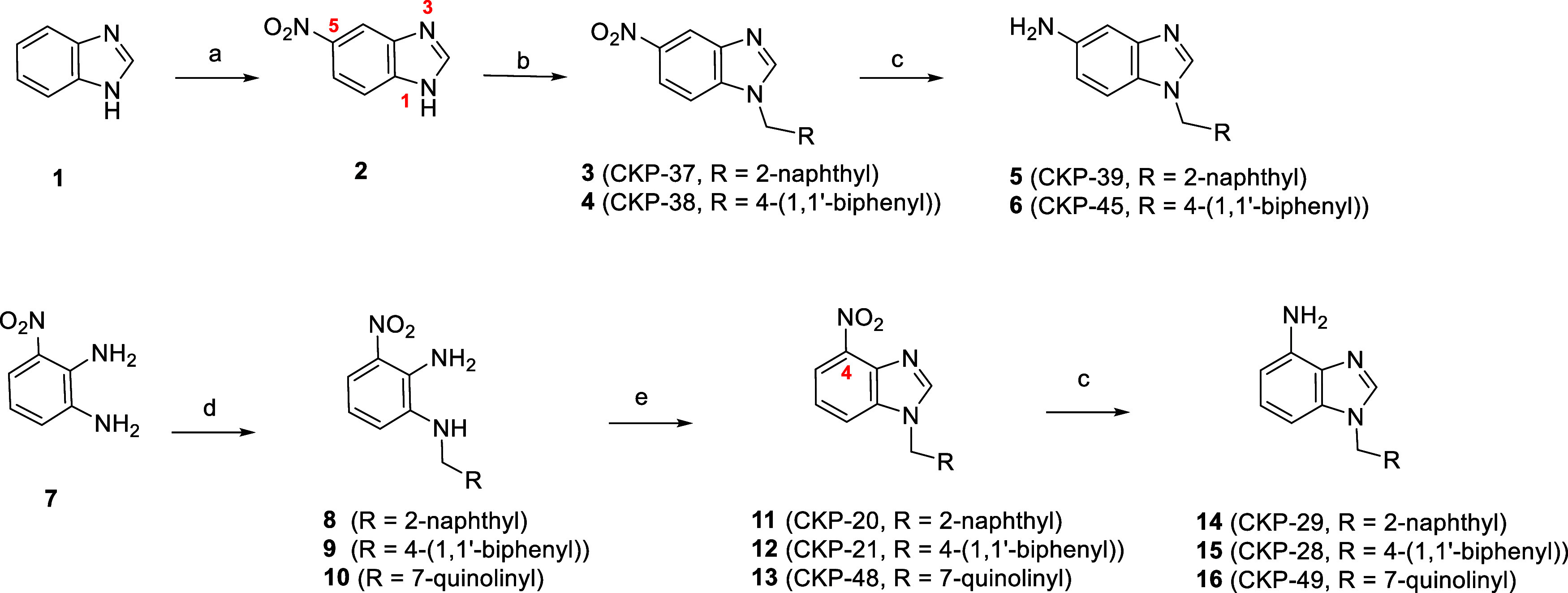
Synthesis of 4-Nitro- and 4-Amino-*N*^1^-Substituted Benzimidazoles *Reagents and conditions*: (a) HNO_3_/H_2_SO_4_, 0 °C, 3 h; (b) 2-(bromomethyl)naphthalene (for **3**) or 4-biphenylmethyl bromide (for **4**), K_2_CO_3_, DMF, 90 °C, 2 h; (c) SnCl_2_·2H_2_O, conc. HCl, gl. AcOH, rt, 1 h; (d) 2-(bromomethyl)naphthalene (for **8**) or 4-biphenylmethyl bromide (for **9**) or 7-(bromomethyl)quinoline hydrobromide salt (for **10**), K_2_CO_3_, DMF, rt, 3–18 h; (e) trimethyl orthoformate, cat. PTSA, PhMe, 80 °C, 2–18 h or trimethyl orthoformate, BF_3_·Et_2_O, DCM, rt, 5 h.

The first series of **CKP22** derivatives was obtained through variation of the *N*-aryl group and the substituents on the phenyl moiety of the benzimidazole scaffold. The synthesis of *N*^1^-substituted benzimidazoles bearing a nitro-substituent at the C-5 position was realized using 1*H*-benzo[*d*]imidazole (**1**) as starting material. Initially, **1** was treated with a mixture of HNO_3_/H_2_SO_4_ to give 5-nitro-1*H*-benzo[*d*]imidazole (**2**) quantitatively (Scheme 1). The latter was subjected to an alkylation reaction using the commercially available 2-(bromomethyl)naphthalene or 4-biphenylmethyl bromide as alkylating agents leading to compounds **3** or **4**, respectively. However, as anticipated, in both cases almost equimolar mixtures of regioisomers were obtained. As exemplified by compound **4**, the ^1^H NMR of the crude mixture showed two *N*-C*H*_2_-biphenyl signals at 5.55 and 5.50 ppm. The regioisomers of **3** and **4** were isolated by FCC and the desired configuration was established based on the observation that for benzimidazoles bearing electron-withdrawing substituents at the C-5 position, the *N*^1^-C*H*_2_-alkyl and H-2 signals resonate more upfield (lower δ) compared to the corresponding *N*^3^-C*H*_2_-alkyl signals.^85^ Finally, compounds **5** and **6** were obtained in high yields upon SnCl_2_-mediated reduction of nitro-compounds **3** and **4**, respectively.

For the synthesis of *N*^1^-substituted benzimidazoles substituted at C-4 a different synthetic strategy was implemented. The commercially available 3-nitrobenzene-1,2-diamine (**7**) was selectively alkylated with either 2-(bromomethyl)naphthalene or 4-biphenylmethyl bromide or 7-(bromomethyl)quinoline hydrobromide salt to give rise to compounds **8**–**10**, respectively (Scheme 1). For the formation of the benzimidazole core, trimethyl orthoformate was used as the CH source under mild conditions to give nitro-compounds **11**–**13**, respectively in excellent yields. Then, compounds **11**–**13** were converted to their corresponding amino-congeners **14**–**16**, upon treatment with SnCl_2_·2H_2_O.

**Scheme 2:**
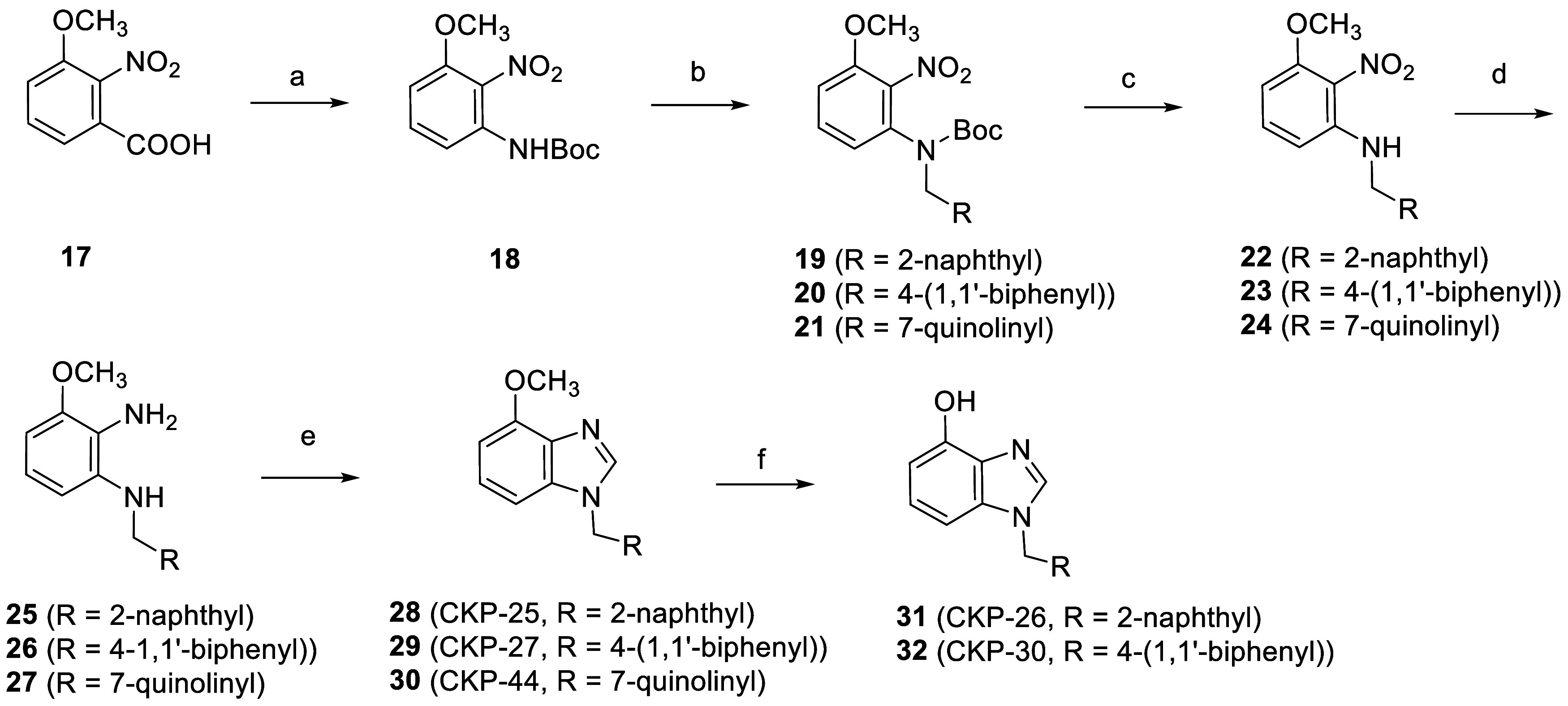
Synthesis of 4-Methoxy and 4-Hydroxy-*N*^1^-Substituted Benzimidazoles *Reagents and conditions*: (a) DPPA/TEA, *t*-BuOH, 100 °C, 4 h; (b) 2-(bromomethyl)naphthalene (for **19**) or 4-biphenylmethyl bromide (for **20**) or 7-(bromomethyl)quinoline hydrobromide salt (for **21**), NaH, DMF, rt, 3 h; (c) TFA, DCM, 0 °C, 1 h; (d) SnCl_2_, conc. HCl, MeOH, 50 °C, 18 h; (e) trimethyl orthoformate, cat. PTSA, PhMe, 80 °C, 2-18 h; (f) BF_3_·S(CH_3_)_2_, DCM, rt, 24–36 h.

The targeted *N*^1^-alkylated benzimidazole derivatives **28**–**30** bearing a methoxy group at the C-4 position, were synthesized using the commercially available 3-methoxy-2-nitrobenzoic acid (**17**) as starting material (Scheme 2). Benzoic acid **17** was subjected to a DPPA-mediated Curtius rearrangement in refluxing *tert*-butanol to afford *N*-Boc aniline **18**.^86^ The latter was then alkylated with either 2-(bromomethyl)naphthalene or 4-biphenylmethyl bromide or 7-(bromomethyl)quinoline hydrobromide salt in the presence of NaH in DMF to give compounds **19**–**21**, respectively, which in turn, were subjected to TFA-mediated removal of the Boc-protection to afford **22**–**24**. The nitro-compounds **22**–**24** were then converted to the corresponding anilines **25**–**27** with anhydrous SnCl_2_ in MeOH at 50 °C in the presence of conc. HCl. Subsequently, anilines **25**–**27** upon treatment with trimethyl orthoformate in toluene at 80 °C using catalytic amount of PTSA gave the substituted benzimidazoles **28**–**30**, respectively Finally, compounds **28** and **29**, were treated with BF_3_·S(CH_3_)_2_ in DCM to give rise to the corresponding phenols **31** and **32**.^87^

**Scheme 3:**
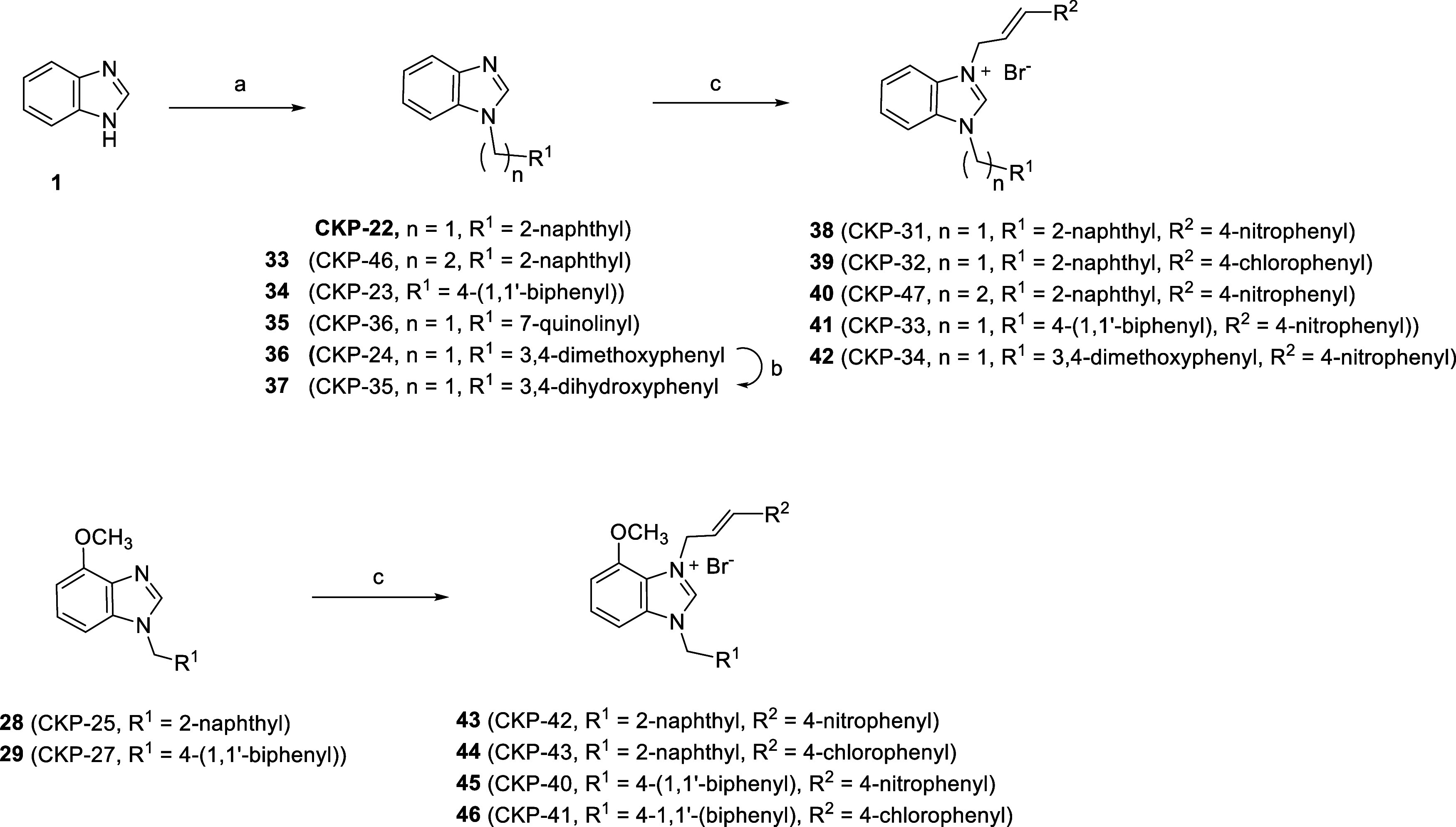
Synthesis of Benzimidazolium Salts *Reagents and conditions*: (a) 2-(bromomethyl)naphthalene (for **CKP22**) or 2-(2-bromoethyl)naphthalene (for **33**) or 4-biphenylmethyl bromide (for **34**) or 7-(bromomethyl)quinoline hydrobromide salt (for **35**), 3,4-dimethoxybenzyl methanesulfonate (for **36**), NaH, THF, rt, 1–3 h; (b) BF_3_·S(CH_3_)_2_, DCM, rt, 24 h; (c) (*E*)-4-nitro-cinnamyl bromide (for **38**, **40-43**, **45**) or (*E*)-4-chloro-cinnamyl bromide (for **39**, **44**, **46**), 1,4-Dioxane, 100 °C, 24 h.

Aiming to expand our compound library for SAR purposes, the naphthyl group of the hit compound was replaced by other aryl substituents (compounds **33**–**37**). Thus, the impact of the nature of the aryl substituent on the activity could be assessed. Hit compound **CKP-22** and derivatives **33**–**36** were easily obtained upon reaction of 1*H*-benzo[*d*]imidazole (**1**) with the appropriate bromides or mesylates in the presence of NaH in THF. Furthermore, compound **36** was treated with BF_3_·S(CH_3_)_2_ in DCM to give **37** bearing a catechol group (Scheme 3). Subsequently, **CKP-22** and **33**–**36** were further reacted with either (*E*)-4-nitro-cinnamyl bromide or (*E*)-4-chloro-cinnamyl bromide in refluxing dioxane to afford the quaternized benzimidazoles **38**–**42**.^88^ Following the same quaternization procedure, compounds **28** and **29** bearing a methoxy-substituent at the C-4 position, gave rise to compounds **43**–**46** (Scheme 3).

### Validation of Computationally Predicted Spike/ACE2 Inhibitors

**Figure 7:**
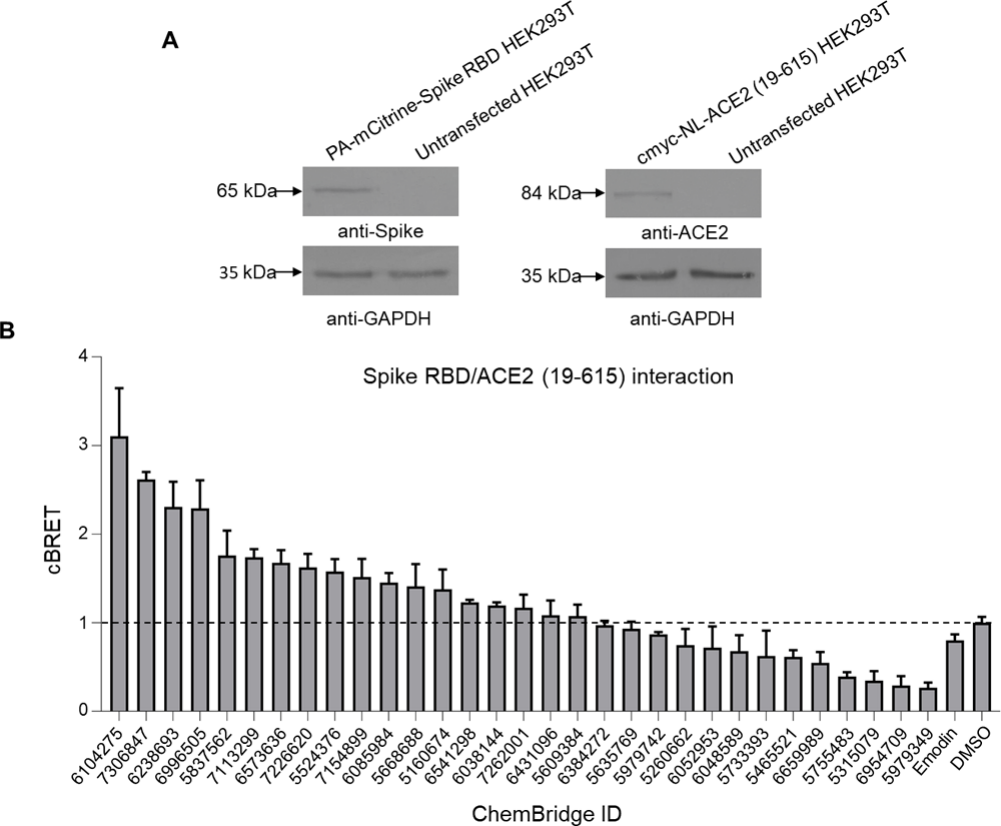
In vitro screening of computationally predicted compounds for Spike/ACE2 inhibitors. (A) Immunoblot analysis of HEK293T cells transiently transfected with expression clones encoding the PA-mCitrine-Spike RBD and cmyc-NL-ACE2 (19–615) proteins generated for the quantification of the Spike RBD/ACE2 (19–615) interaction. Immunoblots were detected with an anti-Spike antibody or an anti-ACE2 antibody. GAPDH was used as a loading control. (B) Effects of computationally predicted compounds on Spike RBD/ACE2 (19–615) interaction in LuTHy assay. The interacting proteins PA-mCitrine-Spike RBD and cmyc-NL-ACE2 (19–615) were co-produced in HEK293T cells in the presence of compounds (100 μM). The cBRET signal corresponding to Spike RBD/ACE2 (19–615) interaction was quantified 48 h post-treatment. Eleven compounds significantly reduced the interaction as compared to the cells treated with DMSO or the positive control Emodin (100 μΜ).

The activity of the qualified S(RBD)-ACE2 (19–615) inhibitors was evaluated in cell-based assays. First, expression clones of PA-mCitrine-Spike RBD and cmyc-NL-ACE2 (19–615) were generated, and their expression efficiency was assessed by immunoblotting in extracts of HEK293T cells transiently transfected with PA-mCitrine-Spike RBD or cmyc-NL-ACE2 (19–615) cDNAs. Bands corresponding to the recombinant PA-mCitrine-Spike RBD and cmyc-NL-ACE2(19–615) proteins were detected at 65 kDa and 84 kDa, respectively suggesting that expression plasmids are suitable for the study of the interaction between Spike RBD and ACE2 (19–615) (Figure 7A). Then, a cell-based LuTHy assay was set up for the quantification of Spike RBD/ACE2 (aa19–615) PPI. HEK293T cells were transiently co-transfected with PA-mCitrine-Spike RBD and cmyc-NL-ACE2 (19–615) plasmids. Control experiments were performed using combinations of PA-mCitrine-Spike RBD/NL or PA-mCitrine/cmyc-NL-ACE2 (19–615). The co-production of recombinant proteins resulted in significantly higher BRET ratios compared to control interactions (Figure S1A), suggesting that this method can be applied for the quantification of Spike RBD/ACE2 (19–615) interaction.

Then, the inhibitory effect of the 31 computationally selected hit molecules on Spike RBD/ACE2 (19–615) interaction was assessed using the previously established LuTHy assay. Compounds were tested in a non-cytotoxic concentration (100 μM) as indicated by MTT assay (Figure S1B) and those reducing Spike RBD/ACE2 (19–615) interaction were considered as positive hits. In total, 11 compounds [5979742, 5260662, 6052953, 6048589, 5733393, 5465521, 6659989, 5755483, 5315079, 6954709, 5979349 (**CKP-22**)] suppressed the interaction in vitro compared to treatment with the solvent DMSO (Figure 7B). Interestingly, the positive control Emodin^89−91^ indeed suppressed Spike/ACE2 interaction but to a lesser extent (∼27%) compared to most positive hits and only in a very high non-cytotoxic concentration (100 μM) (Figures S1C and S2).

**Figure 8:**
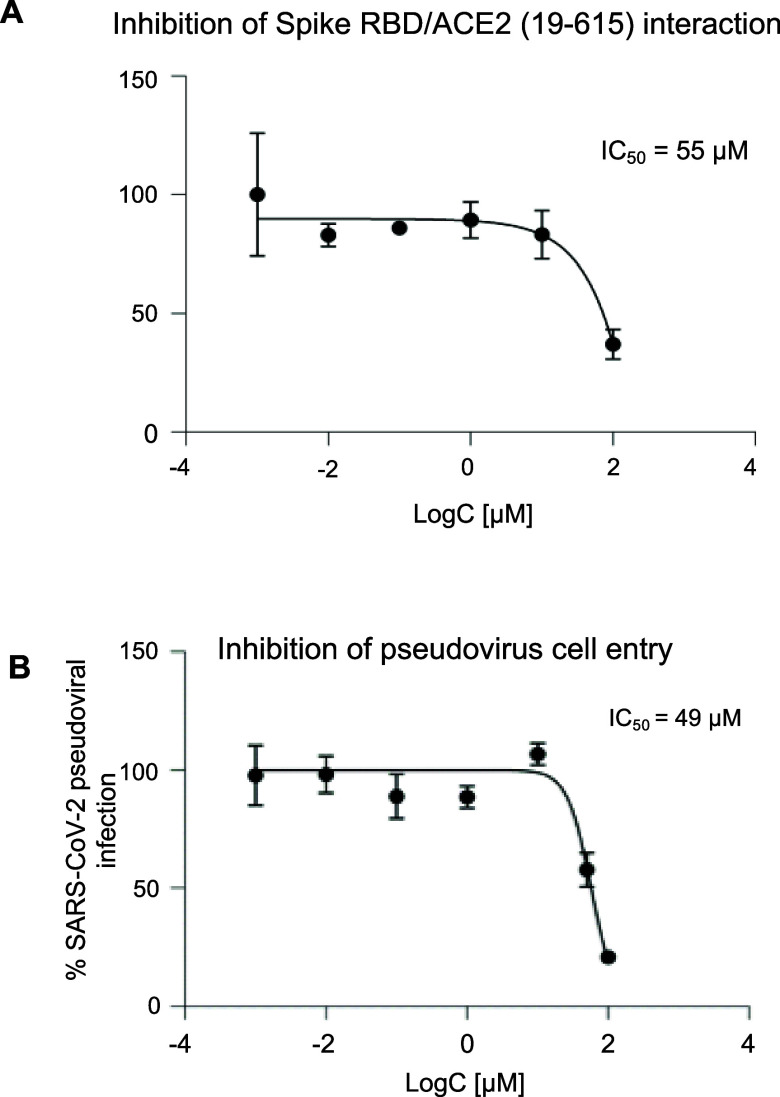
Concentration–response curves obtained for the inhibitory effect of compound 5979349 (**CKP-22**) in cell-based assays. Inhibition of (Α) Spike RDB/ACE2 (aa19–615) interaction in LuTHy assay and (Β) SARS-CoV-2 pseudovirus entry. Data are mean ± SD and were fitted with standard sigmoid curves for IC_50_ determination.

Subsequently these compounds were further assessed for their inhibitory effect in dose-dependent LuTHy assays. These experiments indicated that when tested in a non-cytotoxic concentration, 6 of the 11 selected compounds [compounds 5260662, 6052953, 5465521, 6659989, 5315079, 5979349 (**CKP-22**)] suppressed Spike RBD/ACE2 (19–615) interaction in a concentration-dependent manner, confirming their activity (Figure 8A for **CKP-22** and Figure S2).

The specificity of these compounds was further corroborated in LuTHy assays for a previously established interaction between ataxin-1 and MED-15 proteins.^92^ Interestingly, none of the compounds suppressed ATXN1-MED15 interaction (Figure S3), confirming their specific inhibitory effects on the Spike RBD/ACE2 (19–615) interaction.

Next, we tested whether the compounds suppressing the Spike RBD/ACE2 (19–615) interaction, would prevent infection with SARS-CoV-2. To this end, the six hits were evaluated for their ability to block the entry of a green fluorescent SARS-CoV-2 pseudovirus into human SH-SY5Y cells transduced with red fluorescent ACE2. The blockade of pseudovirus entry would result in a concentration-dependent reduction of the green fluorescence in infected cells. Compared to LuTHy assays, pseudoviral assays depend on the number of viable cells. Initially, the toxicity of the six hits on SH-SY5Y cells was assessed at a concentration range of 100–0.001 μΜ (Figure S4A). Subsequently, the six S(RBD)-ACE2 inhibitors were tested at non-cytotoxic concentrations for inhibition of pseudovirus cell entry. In order to remove any false-positives, results were normalized to the number of viable cells producing red ACE2 and are prone to infection with the green SARS-COV-2 pseudovirus. Two out of the 6 tested compounds, namely 1-(2-naphthylmethyl)-1*H*-benzimidazole [5979349 (**CKP-22**)] and 3-(4-biphenylyloxy)-7-hydroxy-4H-chromen-4-one (6659989) protected human cells from infection in a dose-dependent manner possessing IC_50_ values of 49 ± 2.5 and 3.2 ± 0.2 μM, respectively (Figure 8B for **CKP-22** and Figure S4B for 6659989).

**Figure 9:**
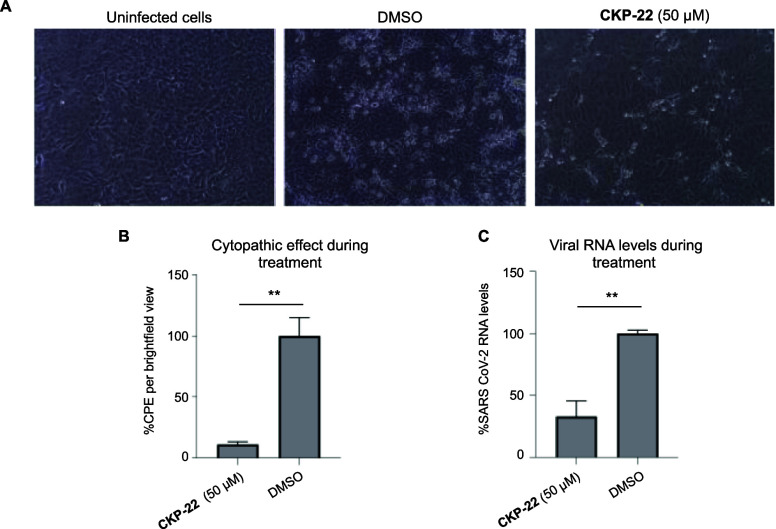
Antiviral activity of 5979349 (**CKP-22**) against SARS-CoV-2 in Vero E6 cells. (A) Brightfield microscopy of Vero E6 cells. Vero E6 cells were infected with SARS-CoV-2 in the absence and presence of compound **CKP-22** (50 μM). Two days post-infection, cells were monitored by microscopy. (B) CPE activity for **CKP-22** in cells. Cells infected with SARS-CoV-2 were cultured in the absence or presence of **CKP-22** (50 μM) for 48 h. After incubation, CPE was recorded using an inverted microscope with phase contrast. **CKP-22** decreases the cytopathic effect of SARS-CoV-2 compared to cells treated only with DMSO. (C) Quantification of SARS-CoV-2 viral load in the supernatant of Vero E6 cells. Viral RNA measured by RT-qPCR in cell supernatant infected with SARS-CoV-2 (MOI of 0.01) and treated with **CKP-22** or DMSO (positive control) for 48 h. Treatment with **CKP-22** leads to significant reduction in viral load compared to cells treated only with DMSO.

Finally, we tested whether the two inhibitors of the SARS-CoV-2 pseudoviral entry protected Vero cells from infection with SARS-CoV-2. Οnly 1-(2-naphthylmethyl)-1*H*-benzimidazole [5979349 (**CKP-22**)] at a non-cytotoxic concentration (Figure S5A), significantly and reproducibly reduced the cytopathic effect (CPE) of the virus by approximately 90% compared to control cells treated only with the solvent (Figure 9A,B). These results were further verified by a qPCR for the quantification of the viral load in the cell culture supernatant. This analysis showed that SARS-CoV-2 levels were reduced by 68% (Figure 9C) indicating that **CKP-22** efficiently blocks Spike-ACE2 interaction and strongly prevents infection and spread of SARS-CoV-2 in vitro.

### Biological Evaluation and Structure Activity Relations of Synthesized CKP-22 Derivatives

**Figure 10:**
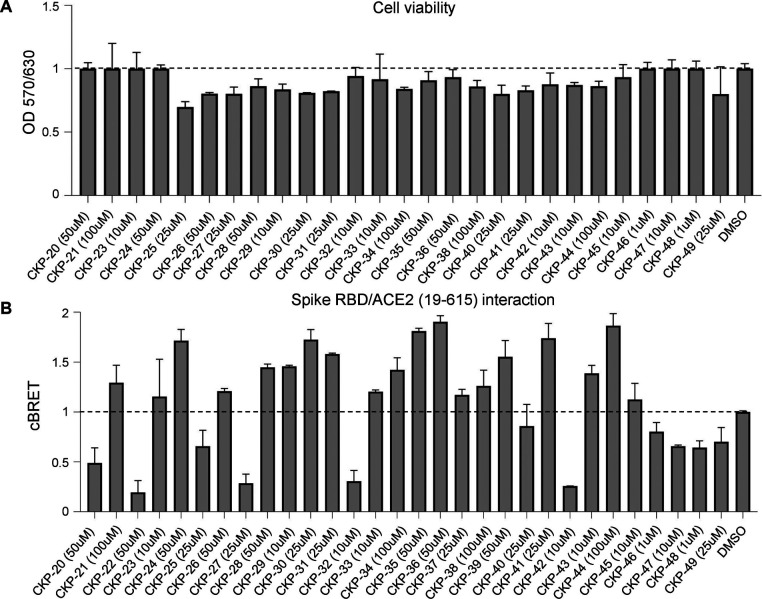
Cytotoxicity and effects of the synthesized compounds on the Spike RBD/ACE2 (aa19–615) interaction. (A) Viability of HEK293T cells treated with the new compounds. Cell viability was measured 48 h later using a MTT assay. Cells treated with DMSO served as control. (B) Effect of the new compounds on Spike RBD/ACE2 (aa19–615) interaction using a cell-based quantification assay. Interaction of PA-mCitrine-Spike RBD and cmyc-NL-ACE2 (aa19–615) proteins was quantified in HEK293T cells in the presence of the new compounds used at the highest non-cytotoxic concentration. The cBRET signal corresponding to Spike RBD/ACE2 (aa19–615) interaction was quantified 48 h post treatment. Data are presented as a mean of triplicates ± SD.

**Figure 11:**
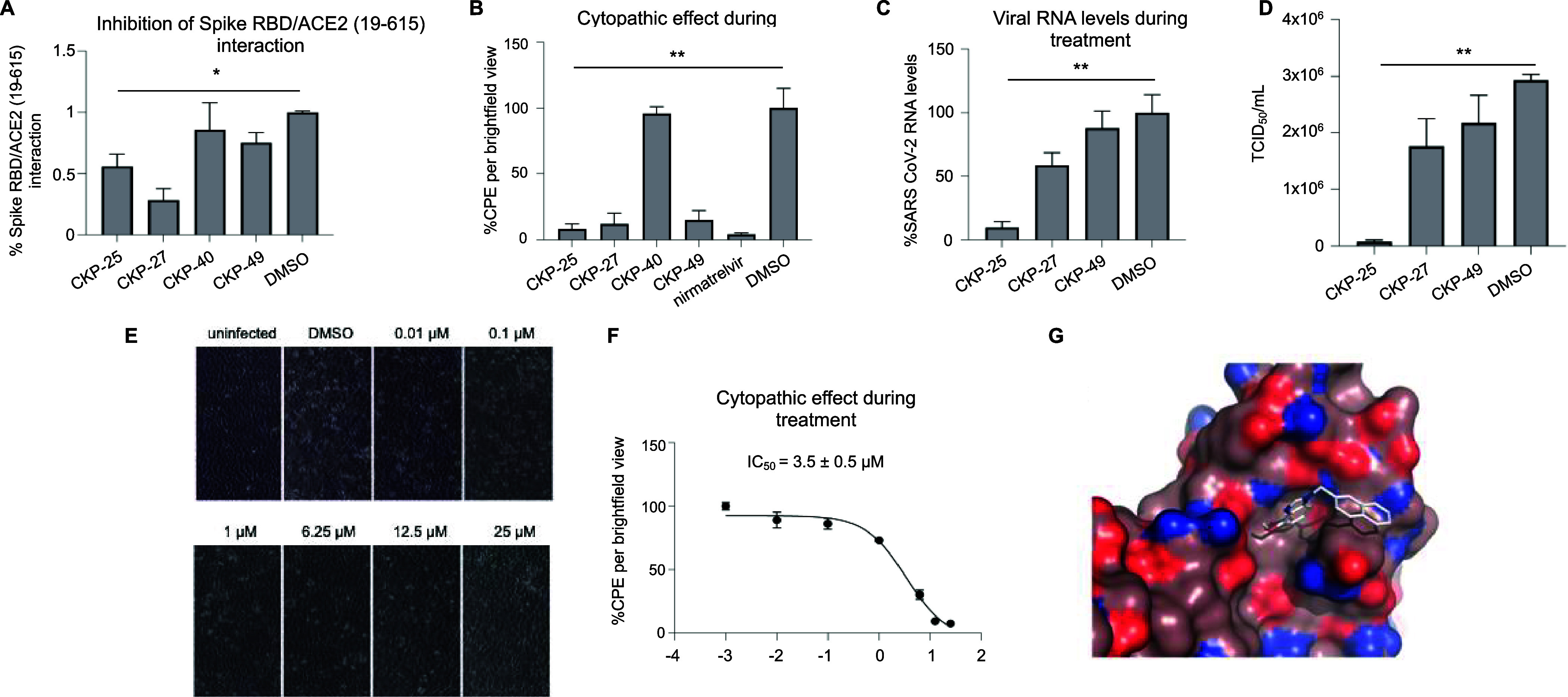
Derivatives of 1-(2-naphthylmethyl)-1H-benzimidazole (**CKP-22**) protect from infection with SARS-CoV-2. (Α) Quantification (cBRET signal) of Spike-RBD/ACE2 (19–615) interaction in the presence of **CKP-22** derivatives (**CKP-25**, **CKP-27**, **CKP-40** and **CKP-49**, 25 μM final concentration) or the solvent DMSO. (Β) SARS-CoV-2 CPE in Vero E6 cells treated with the new compounds (12.5–25 μM final concentration) compared to the solvent DMSO. Τhe commercial SARS-CoV-2 inhibitor nirmatrelvir was used as a positive control. (C) SARS-CoV-2 load in the supernatant of infected Vero E6 cells treated with derivative compounds or the solvent DMSO. (D) TCID_50_ of SARS-CoV-2 after treatment with compounds **CKP-25**, **CKP-27**, **CKP-49** or the solvent DMSO. (E) Brightfield microscopy of SARS-CoV-2 infected Vero E6 cells treated with various varying concentrations (ranging from 0.01 to 25 μM) of compound **CKP-25**. (F) Concentration-dependent effect of compound **CKP-25** inhibition in SARS-CoV-2 CPE (IC_50_ = 3.5 ± 0.5 μΜ). Treatment with DMSO was arbitrarily set to 100%. Data in bar graphs are shown as mean ± SD (*p-value < 0.05, **p-value < 0.0.01). (G) Docking solution of compound **CKP-25** shown as white colored sticks residing in region A of the RBD domain.

The 29 synthesized **CKP-22** derivatives were initially tested for their cytotoxicity against HEK293T cells (Figure 10A). Subsequently their effect on Spike RBD/ACE2 (19–615) interaction was assessed using LuTHy assay (Figure 10B and Figure 11A). Each analogue was tested at the highest non-cytotoxic concentration against HEK293T cells. **CKP-22** was included for comparison. Ten derivatives (**CKP-20**, **CKP-25**, **CKP-27**, **CKP-32**, **CKP-40**, **CKP-42**, **CKP-46**, **CKP-47**, **CKP-48** and **CKP-49**) suppressed the Spike RBD/ACE2 (19–615) interaction. In particular, **CKP-20** at 50 μΜ by 51%, **CKP-25** at 25 μΜ by 34%, **CKP-27** at 25 μΜ by 72%, **CKP-32** at 10 μΜ by 70%, **CKP-40** at 25 μΜ by 14%, **CKP-42** at 10 μΜ by 74%, **CKP-46** at 1 μΜ by 20%, **CKP-47** at 10 μΜ by 34%, **CKP-48** at 50 μΜ by 36% and **CKP-49** at 50 μΜ by 30% in comparison to **CKP-22** at 50 μΜ by 81%.

Among the 4-nitro-*N*^1^-substituted benzimidazoles (compounds **11**–**13**) compounds **11 (CKP-20**) and **13** (**CKP-48**) substituted by a naphthyl or a 7-quinolinyl group showed activity. Conversely, among their 4-amino congeners the *N*^1^-7-quinolinylmethyl-substituted **16** (**CKP-49**) suppressed the Spike RBD/ACE2 (19–615) interaction by 30% compared to the control (DMSO). Gratifyingly the 4-methoxy-substituted benzimidazole derivatives **28** (**CKP-25)** and **29** (**CKP-27)** suppressed Spike RBD/ACE2 (19–615) interaction by 34 and 72%, respectively compared to the control (DMSO). However, none of the 4-hydroxy congeners of **28** and **29**, compounds **31** (**CKP-26**) and **32** (**CKP-30**) maintained inhibitory activity. Interestingly, in this series the 4-methoxy congener of the active amino compound **16 (CKP**-**49**) [derivative **30** (**CKP-44**)] did not exhibit any effect. This points out to the fact that both the C-4 and *N*^1^ substituents are key for activity. Modifying the *N*^1^–naphthyl substituent in the hit compound **CKP-22**, by other aryl moieties, namely biphenyl, 7-quinolinyl, 3,4-dimethoxyphenyl or 3,4-dihydroxyphenyl (compounds **34**–**37**) resulted in loss of activity. In contrast when the methylene spacer between the naphthyl group and the *N*^1^ nitrogen in **CKP-22** was extended by one carbon, compound **33** (**CKP-46**) activity was retained. Finally, we proceeded in quaternization of *N*^3^ in the hit compound **CKP-22** and its ethylnaphthyl, biphenyl and 3,4-dimethoxyphenyl congeners compounds **33** (**CKP-46**), **34 (CKP-23)** and **36 (CKP-24)**, respectively. Derivatives **39** (**CKP-32**) and **40** (**CKP-47**) resulted in 70 and 35% reduction, respectively. In the case of the *N*^1^-naphthylmethyl-substituted salt the *N*^3^-4-chlorophenylethylene group is preferred (**CKP-32**), while in the case of the *N*^1^-naphthylethyl-substituted salt the *N*^3^-4-nitrophenylethylene group (**CKP-47**) results in inhibitory activity. Furthermore, among the compounds resulting from the quaternization of the active derivatives **CKP-25** and **CKP-27**, with 4-nitrophenylethylene or 4-chlorophenyethylene groups, derivatives **43**–**45**, the 4-nitrophenylethylene derivatives were active, namely **43 (CKP-42)** and **45 (CKP-40)**.

Subsequently, we set out to evaluate the antiviral activity of the active compounds against SARS-CoV-2. As a first step their toxicity against Vero E6 cells was assessed (Figure S5B). Derivatives **CKP-32** and **CKP-47** were toxic thus, they were not further tested. Out of the remaining 8 compounds only **CKP-25, CKP-27** and **CKP-49** consistently reduced the cytopathic effect of the virus with the most potent being **CKP-25.** This effect was comparable to the effect of the commercial inhibitor of SARS-CoV-2 nirmatrelvir (Paxlovid, Pfizer) (Figure 11Β). As expected, derivative **CKP40**, which was used as a negative control and did not significantly suppress the Spike RBD/ACE2(19–615) interaction, was also not protective against SARS-CoV-2. Furthermore, compounds **CKP-25, CKP-27** and **CKP-49** also reduced the viral load in the cell culture supernatant (Figure 11C). In particular, calculation of the half maximal tissue culture infectious dose (TCID_50_) indicated that compound **CKP-25** reduced by approximately 90% the viral titter (Figure 11D), while, the IC_50_ was found equal to 3.5 ± 0.5 μΜ (Figure 11E,F). Collectively, these results suggest that compound **CKP-25** protects mammalian cells from infection with SARS-CoV-2 and is a lead compound against COVID-19. Based on our docking studies compounds **CKP-22** and **CKP-25** reside in region A of the Spike RBD domain (Figures 3C and 11G, respectively). Moreover, the methoxy substituent in **CKP-25** serves as the HB acceptor resulting in an additional interaction, which turn positively influences the activity. MD simulations were performed to study the interaction behavior of **CKP-25** in parallel with any protein conformational changes that may occur. The data collected during 100 ns of simulation time (Charts S1–S4) showcase that compound **CKP-25** resides for 77.5% of the time with median RMSD of 0.3 nm in a similar manner as predicted in Figure 11G (region A of the S(RBD) domain, while for the remaining 22.5% (RMSD > 0.5 nm) it flips mainly with the naphthyl substituent toward region B. The pi-pi stacking of Tyr505 indeed plays an important role on maintaining this binding mode for **CKP-25** since beside this interaction the compound forms only one HB for the 99.9% of the simulation. Moreover, for the ∼2/3 of the 77.5% that have an RMSD above 0.2 nm we noticed a configuration that showed loss of planarity within either of the naphthyl substituent with Tyr505, the Tyr505-Arg403 or both and modifying the RBD domain. In all cases the methoxy substituent served as the HB acceptor between the ligand and the protein with adjacent residues like Tyr496 or Arg403 during our simulation (Chart S4).

### In Vitro ADME-Tox Studies

#### Calculated Water Solubility

A predicted solubility value (log *S*_w_) of compound **CKP-25** was available to us since the initial filtering over FAF4Drugs server.^70^ The method used for calculation by the server is a structure only method based on Delaney et al. ESOL method^93^ resulting to a **–4.48** log *S*_w_. Although, when the melting point (M pt) is available the most efficient method is using the “General Solubility Equation” (see eq 1).^94^

Equation 1 General Solubility Equation
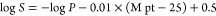
1

Therefore, the GSE based solubility for **CKP-25** according to eq 1 is log *S* = **–4.25** – 0.01(163.5 – 25) + 0.5 = **–5.13**. It is should be noted that the lipophilicity (log *P*) used in the GSE above is the calculated value using *X*log *P*3^95^) and not an experimental partition coefficient over octanol/water.

#### Microbial Mutagenicity Assay: Ames Test

To evaluate the mutagenic potential of **CKP-25**, a bacterial reverse mutation assay was performed using the *E. coli* WP2 uvrA strain. The genetic background of the bacterial strains was verified prior to testing to ensure the accuracy and reliability of the results. Positive control experiments were conducted to confirm the sensitivity of the test system and the efficacy of the S9 metabolic activation mix. Methylmethanesulfonate (without metabolic activation) and 2-aminoanthracene (with metabolic activation) were used as positive controls, both significantly increasing the number of revertant colonies grown in selective agar plates. The results, summarized in Table 1, demonstrate that **CKP-25**, tested at concentrations ranging from 12.5 to 200 μg/plate, did not induce a significant increase in the number of revertant colonies of the *E. coli* WP2 uvrA strain, when compared to the negative control (DMSO). This lack of increase was observed in the presence and absence of the S9-mix and was consistent across all tested concentrations. Thus, **CKP-25** does not exhibit mutagenic activity in AMES testing.

**Table 1:** Assessment of the Mutagenic Activity of Compound **CKP-25**

compound	–S9		+S9	
	mean revertants	STDEV(∓)	mean revertants	STDEV (∓)
CKP-25 (μg/plate)				
200	5.5	0.7	16	2.8
100	11.5	2.1	13	0
50	13	2.8	15	5.6
25	12.5	2.1	19	1.4
2.5	7.5	0.7	13	2.8
DMSO	18.5	0.7	16.5	0.7
methyl methanesulfonate	>300			
2-aminoanthracene			>300	

#### Metabolic Stability (Residual % of Time Zero)

**CKP-25** was studied for its metabolic stability. In particular, the metabolic stability of a compound can be interpreted using several approaches, as it can be ranked in terms of its intrinsic clearance (CL_int_) and in vitro *t*_1/2_ values or based on parent structure loss during metabolic reactions^96,97^ CL_in_ describes the maximum activity of liver (microsomal proteins or hepatocytes) toward a compound not influenced by other physiological determinants, such as hepatic blood flow and drug binding within the blood matrix and in vitro *t*_1/2_ expresses the time for 50% disappearance of the compound. Parent structure loss was our option for metabolic stability data interpretation of **CKP-25**. Parent structure loss is classified as very slow (<5%), slow (5–19%), moderate (20–50%), fast (50–80%) or very fast (>80%). Such categories have been defined according to set criteria, namely, high metabolism (*t*_1/2_ value of <30 min), moderate metabolism (30 min < *t*_1/2_ value of <60 min) and low metabolism (*t*_1/2_ value of >60 min).^98−100^

**CKP-25** shows slow substrate depletion, suggesting a low intrinsic clearance classification band (Table 2). For humans, a CL_int_ <8.6 μL/min/mg protein defines a low intrinsic clearance classification band, whereas a CL_int_ >47.0 μL/min/mg protein defines a high intrinsic clearance classification band. Low clearance compounds are characterized by reduced doses, enhanced exposure and prolonged half-life, being suitable for once-daily dosing.

**Table 2:** Metabolic Stability and CYP450-Mediated Metabolism Data of Compound **CKP-25**

metabolic stability in human liver microsomes (residual % of time zero)
94

CYP450s (% inhibition)						
CYP1A2	CYP2A6	CYP2B6	CYP2C9	CYP2C19	CYP2D6	CYP3A4
0	6	0	11	20	1	3

#### Isozyme-Specific CYP450 Metabolism

Human CYP450 enzymes are crucial for xenobiotic biodegradation, metabolism, and toxicity as well as xenobiotic-host and/or xenobiotic-xenobiotic interactions.^101^

Upon linear velocity conditions in vitro, the depletion rate of test-compounds may be extrapolated to (a) in vivo hepatic clearance, (b) extraction ratio, and (c) the effect of hepatic first-pass metabolism to total oral bioavailability. Biodegradation, metabolic, and toxicity liabilities can be identified early on and thus, inform structure–activity relationships.^98−100^

**Figure 12:**
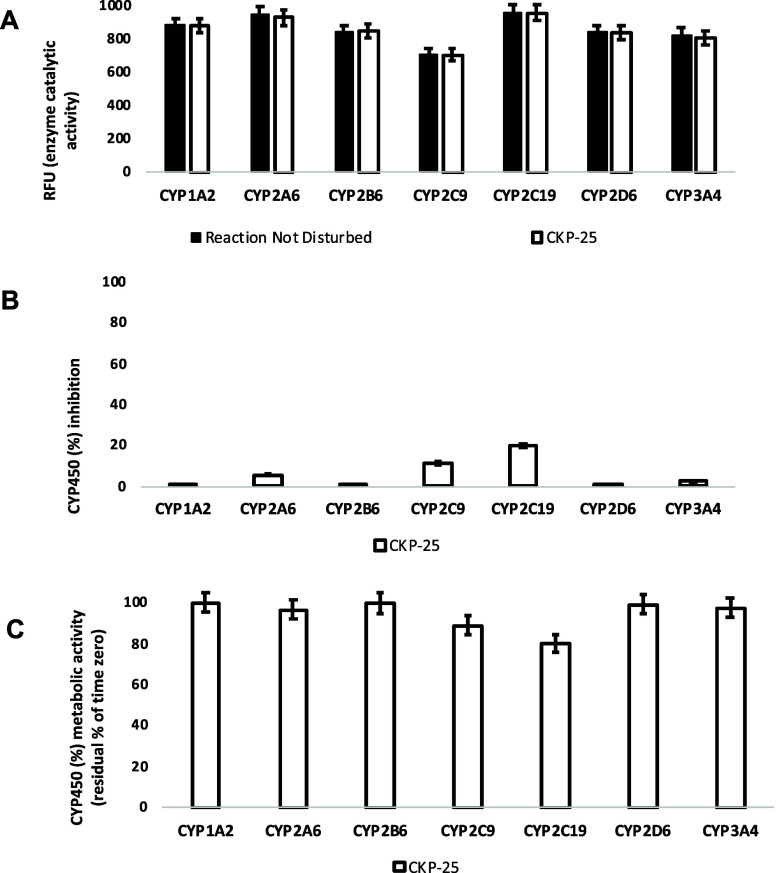
(A) Enzyme (catalytic) activity of the CYP1A2, CYP2A6, CYP2B6, CYP2C9, CYP2C19, CYP2D6, and CYP3A4 isoenzymes upon the administration of **CKP-25** at 1 μΜ (60 min). RFU, relative fluorescence units. Reaction Not Disturbed, reaction without test-compound. (B) CYP450 (%) inhibition of CYP1A2, CYP2A6, CYP2B6, CYP2C9, CYP2C19, CYP2D6, and CYP3A4 isoenzymes upon the administration of **CKP-25** at 1 μM (60 min). (C) CYP450 (%) metabolic activity of CYP1A2, CYP2A6, CYP2B6, CYP2C9, CYP2C19, CYP2D6, and CYP3A4 isoenzymes upon the administration of **CKP-25** at 1 μM (60 min).

For this, the activity of CYP1A2, CYP2A6, CYP2B6, CYP2C9, CYP2C19, CYP2D6, and CYP3A4 was assessed after the administration of **CKP-25** at 1 μM to determine (a) the oxidative (CYP-mediated) metabolic stability profile in question and (b) the enzyme metabolizing isoforms responsible (the test system consists of recombinant human CYP450 and CYP450 reductase; cytochrome b5 may also be present). Herein, no concentration-dependent effects were reported. CYP450 substrates can alter enzyme activity by blocking the enzyme active site, changing enzyme conformation, disrupting enzyme structure and/or functioning.^102,103^**CKP-25** did not decrease the enzyme (catalytic) activity of the CYP450 system tested herein. CYP450 enzyme inhibition may lead to unexpectedly high exposure of coadministered xenobiotics and hence, increase the risk for adverse effects. No product inhibition or mechanism-based inactivation of the CYP450 isoenzymes in question was obtained. Test-compounds with poor solubility can show artificially low CYP450 inhibition and thus, chemical entities with potential drug–drug interaction toxicities may be overlooked. No solubility issues were observed. Overall, **CKP-25** was a weak inhibitor of the CYP450 system (see Table 2 and Figure 12).

## Conclusions

In conclusion through AI enabled VS and subsequent in vitro screening we were able to identify benzimidazole derivative **CKP-22** as a hit compound protecting Vero E6 cells from infection with SARS-CoV-2 mediated through the inhibition of S(RBD)-ACE2 interaction. Hit to lead optimization studies revealed that 4-methoxy-1-(naphthalen-2-ylmethyl)benzimidazole (**CKP-25**) suppressed the Spike RBD/ACE2 (19–615) interaction, reduced the cytopathic effect of SARS-CoV-2 in Vero E6 cells (IC_50_ = 3.5 ± 0.5 μM) and reduced the viral load in cell cultures supernatants. In addition, the isozyme-specific CYP450 study revealed that **CKP-25** was a weak inhibitor of the CYP450 system, while it did not show biodegradation or liver metabolism (≥94% residual of time zero at *t* = 60 min) or implied safety issues. Therefore, **CKP-25** can be considered as a lead compound against COVID-19 infection.

## Experimental Section

### Pre-filtering Profiling

ChemBridge corporation precompiled macrocycle, core and dedicated SARS-CoV-2 libraries in SDF formatted files were downloaded from their Hit2Lead site. Libraries were cleaned from any salts’ counterions being present using OpenBabel v2.4.1^62^ and introduced as obtained to FAF-Drugs4 server^70^ for filtering applying drug-like properties,^65,104−108^ promiscuity and protein–protein interaction (PPI)^71^ options based on their ADME-Tox descriptors (i.e., XLOGP3,^109^ PKs, bioavailability and Lilly Medchem relaxed rules.^109^ Accepted molecules meeting all requirements were compiled in SDF formatted files and used to perform the virtual screening (VS).

### AI-Based Virtual Screening

Following the Prefiltering step, accepted molecules in SDF format were given as input to Virtual Screening against the crystal structure of the spike protein S1 subunit bound to the extracellular domain of the ACE2 receptor (PDB ID: 6M0J). The AI-enabled Virtual Screening pipeline combines the docking result of Smina^61^ with an AI-based rescoring function which operates upon Smina’s docking results and is able to distinguish more efficiently potential binding complexes.^60^ Docking was constrained on the interaction site of the S-protein with the ACE2, while 50 docked poses were generated for each compound conformation. The resulted protein-compound docked complexes were rescored with our custom AI-based rescoring function, a properly designed 3D convolutional neural network (3D-CNN), which aims to automatically capture the underlying mechanism responsible for the protein–ligand binding. A fusion approach is proposed to achieve the optimal performance, which combines the Smina predicted affinities with the new AI-score. The already qualified molecules were further grouped into clusters of structural similarity using a 3D shape descriptor.^60^

### Post-filtering

Decision-making for selection of compounds in VS data sets were based on three methods; molecular modeling, molecular properties and protein–ligand interactions formed.^110,111^

### Molecular Modeling

Ηit validation of the VS results and modeling of the benzimidazole analogues **CKP-22** compound library was performed with the program PyRx^112^ using Vina^113,114^ functionality, running in Genetic Algorithm. Crystal structures of the SARS-CoV-2 virus spike protein variants; wild-type,^10^ alpha,^78^ beta,^78^ gamma,^78^ delta,^79^ kappa,^80^ and omicron BA1^81^/BA2^82^/BA3^84^/BA4^83^ (PDB entries 6m0j, 7ekf, 7ekg, 7ekc, 7wbq, 7tez and 7u0n/7xo9/7xb1/7zxu, respectively) were obtained from the protein databank. All of them were cleaned from the respective ACE2 protein parts and prepared accordingly for docking after being aligned over the wild-type entry 6m0j^83^ with the use of software PyMol molecular graphic system v1.5.0.3 (see Table S2). All compounds were designed using PerkinElmer ChemDraw v20.0 and underwent structure minimization in MM2 force field^115^ found within PerkinElmer Chem3D v22.0.0.0, where they were eventually saved as *.mol2 extension files. Ligand preparation was done within PyRx^112^ and used thereof for molecular modeling. Based on VS the resulting search space was centered in *X*: −32.1814, *Y*: 22.2131, *Z*: 2.9869 with respective volumes *X*: 22.5547, *Y*: 24.6395, *Z*: 21.6589, generating a grid space of 12,036.6 Å.^116^ No residues modifications were made on the protein structures and the docking was performed without any constraints. While the protein is treated as rigid and the solely parameter adjustment was observed on the “exhaustiveness”, which value was set to 20 instead of the default 8.

### MD Simulations

Vina derived S(RBD) domain docking complex with inhibitor **CKP-25** was used as the input for molecular dynamics simulation using GROMACS 2023.3^117^ on a Hyper-V virtual machine running Ubuntu 24.04.1 over a typical Dell XPS desktop. A cubic box was created with 1 nm dimension, and TIP3P water molecules were added to solvate the system. The system was then neutralized by adding chloride ions which replaced equivalent solvent molecules. Parametrization of the protein–ligand complex was performed with the CHARMM36 force field July 2022 version^118^ and energy minimized. Equilibration stage was performed for NVT/NPT 100 ps with hydrogen-bond constraints. Following the equilibration we performed the MD simulation for 100 ns at 300 K using modified Berendsen thermostat with 2 fs integration time and VDW cutoff distance at 1.2 nm. Molecular dynamics trajectory was analyzed using Grace 5.1.25^119^ and VMD 1.9.4a53.^120,121^

### Chemistry

#### General

Melting points were determined with a Shanghai Micro Melting Point apparatus and are uncorrected. ^1^H NMR spectra were recorded on Varian spectrometers operating at 300 or 600 MHz and ^13^C NMR spectra were recorded at 75 or 150 MHz, using CDCl_3_, CD_3_OD, DMSO-*d*_*6*_ or acetone-*d*_*6*_ as solvents. Chemical shifts are reported in δ units, parts per million (ppm) downfield from TMS. Electro-spray ionization (ESI) mass spectra were recorded on a LC-MSn Fleet, Thermo spectrometer using MeOH as solvent. HRMS spectra were recorded in the ESI mode, on UPLC-MSn Orbitrap Velos-Thermo. Flash column chromatography (FCC) was performed on Merck silica gel 60 (230–400 mesh) and TLC on Merck 60 F254 films (0.2 mm) precoated glass plates. Spots were visualized with UV light at 254 nm and PMA stain. All solvents were dried and/or purified according to standard procedures prior to use. All reagents employed in the present work were purchased from commercial suppliers and used without further purification. Reactions were run in flame-dried glassware under an atmosphere of argon. The purity of final compounds was determined by high-performance liquid chromatography (HPLC) (Thermo Scientific HPLC SPECTRASYSTEM with a Thermo Scientific SPECTRASYSTEM UV2000 detector and a EC 250/4.6 NUCLEOSIL 100–5 C18 HD column) using the following analytical method: 20% H_2_O_0.1%TFA/80% CH_3_CN, isocratic, flow rate 1 mL/min, injection volume 20 μL, UV detection at 254 nm; The purity of all final compounds was >97%.

#### General Procedure for the Alkylation of 5-Nitrobenzimidazole

To a suspension of 5-nitrobenzimidazole (0.16 g, 1.0 mmol) and K_2_CO_3_ (0.14 g, 1.0 mmol) in DMF (2 mL), the appropriate bromide (1.0 mmol) was added. The resulting mixture was heated at 90 °C for 2 h, it was left to attain RT, diluted with water and the aqueous phase was extracted twice with EtOAc. The combined organic layers were washed with brine, dried over Na_2_SO_4_ and evaporated to dryness to give the projected compounds as a mixture of regioisomers. The desired regioisomers were isolated after FCC purification.

#### 1-(Naphthalen-2-ylmethyl)-5-nitro-1*H*-benzo[*d*]imidazole (**3**) (**CKP-37**)

Compound **3** was prepared using 2-(bromomethyl)naphthalene (0.22 g, 1.0 mmol) according to the general procedure described above. White solid, 0.15 g (50% yield); *R*_f_ = 0.29 (Hexanes/EtOAc 1:1 v/v); ^1^H NMR (600 MHz, CDCl_3_): δ 8.78 (d, *J* = 1.8 Hz, 1H), 8.36 (s, 1H), 8.19 (dd, *J* = 8.4 and 1.8 Hz, 1H), 7.88–7.82 (m, 2H), 7.80–7.78 (m, 1H), 7.68–7.65 (m, 1H), 7.54–7.50 (m, 2H), 7.41 (d, *J* = 8.4 Hz, 1H), 7.28 (dd, *J* = 8.4 and 1.8 Hz, 1H), 5.61 (s, 2H); ^13^C NMR (150 MHz, CDCl_3_): δ 146.6, 144.3, 142.4, 137.8, 133.3 (two C), 131.4, 129.7, 128.0 (two C), 127.2, 127.1, 126.7, 124.5, 119.4, 117.0, 110.6, 50.0; ESI-HRMS (*m*/*z*): calcd. for C_18_H_14_N_3_O_2_ [M + H]^+^ 304.1086; found 304.1075. HPLC analysis: *t*_R_ = 5.44 min, purity = 99.2%.

#### 1-([1,1′-Biphenyl]-4-ylmethyl)-5-nitro-1*H*-benzo[*d*]imidazole (**4**) (**CKP-38**)

Compound **4** was prepared using 4-biphenylmethyl bromide (0.25 g, 1.0 mmol) according to the general procedure described above. White solid, 0.17 g (52% yield); *R*_f_ = 0.27 (Hexanes/EtOAc 3:7 v/v); ^1^H NMR (600 MHz, CDCl_3_): δ 8.77 (s, 1H), 8.31 (s, 1H), 8.23 (d, *J* = 8.4 Hz, 1H), 7.60 (d, *J* = 7.8 Hz, 2H), 7.55 (d, *J* = 7.8 Hz, 2H), 7.46–7.40 (m, 4H), 7.38–7.34 (m, 1H), 7.28 (d, *J* = 8.4 Hz, 1H), 5.49 (s, 2H); ^13^C NMR (150 MHz, CDCl_3_): δ 146.5, 144.2, 142.1, 140.1, 137.9 (two C), 133.1, 129.1 (two C), 128.2 (two C), 128.0, 127.8 (two C), 127.2 (two C), 119.4, 117.2, 110.5, 49.4; ESI-HRMS (*m*/*z*): calcd. for C_20_H_16_N_3_O_2_ [M + H]^+^ 330.1243; found 330.1233. HPLC analysis: *t*_R_ = 7.14 min, purity = 99.5%.

#### General Procedure for *N*-Alkylation of 3-Nitro-1,2-phenylenediamine (**7**)

To a solution of 3-nitro-1,2-phenylenediamine (**7**) (0.15 g, 1 mmol) in DMF (5 mL), K_2_CO_3_ (0.28 g, 2 mmol) was added followed by the addition of the appropriate aryl bromide (1.3 mmol). The reaction mixture was stirred at RT for 6 h, diluted with water and extracted twice with EtOAc. The combined organic layers washed with water, dried over Na_2_SO_4_ and evaporated to dryness. The desired compounds were obtained in pure form after FCC purification.

#### *N*^1^-(Naphthalen-2-ylmethyl)-3-nitrobenzene-1,2-diamine (**8**)

Compound **8** was prepared using 2-(bromomethyl)naphthalene (0.29 g, 1.3 mmol) according to the general procedure described above. Red solid, 0.22 g (75% yield); *R*_f_ = 0.11 (Hexanes/EtOAc 9:1 v/v); ^1^H NMR (600 MHz, acetone-*d*_*6*_): δ 7.93 (s, 1H), 7.91–7.88 (m, 2H), 7.87–7.85 (m, 1H), 7.59 (dd, *J* = 8.4 and 1.2 Hz 1H), 7.51–7.47 (m, 3H), 6.82 (d, *J* = 7.8 Hz, 2H), 6.57 (t, *J* = 7.8 Hz, 1H), 5.20 (unresolved t, 1H), 4.61 (d, *J* = 5.4 Hz, 2H); ^13^C NMR (150 MHz, acetone-*d*_*6*_): δ 138.7, 137.6, 137.1, 134.5, 133.8, 133.0, 129.0, 128.51, 128.48, 127.0, 126.9 (two C), 126.6, 116.9, 116.0, 115.0, 49.1; ESI-HRMS (*m*/*z*): calcd. for C_17_H_14_N_3_O_2_ [M – H]^+^ 292.1086; found 292.1083.

#### *N***^1^**-([1,1′-Biphenyl]-4-ylmethyl)-3-nitrobenzene-1,2-diamine (**9**)

Compound **9** was prepared using 4-biphenylmethyl bromide (0.32 g, 1.3 mmol) according to the general procedure described above. Red solid, 0.20 g (63% yield); *R*_f_ = 0.13 (Hexanes/EtOAc 9:1 v/v); ^1^H NMR (600 MHz, acetone-*d*_*6*_): δ 7.67–7.61 (m, 4H), 7.56–7.52 (m, 2H), 7.50 (dd, *J* = 8.7 and 1.3 Hz, 1H), 7.45 (t, *J* = 7.8 Hz, 2H), 7.38–7.33 (m, 1H), 6.93–6.67 (m, 3H), 6.60 (dd, *J* = 8.8 and 7.6 Hz, 1H), 5.14 (t, *J* = 5.4 Hz, 1H), 4.49 (d, *J* = 5.4 Hz, 1H); ^13^C NMR (150 MHz, acetone-*d*_*6*_): δ 141.5, 140.7, 139.2, 138.7, 137.1, 133.0, 129.7 (two C), 129.0 (two C), 128.1, 127.8 (two C), 127.6 (two C), 116.9, 115.9, 114.9, 48.6; ESI-HRMS (*m*/*z*): calcd. for C_19_H_16_N_3_O_2_ [M – H]^+^ 318.1243; found 318.1245.

#### 3-Nitro-*N*^**1**^-(quinolin-7-ylmethyl)benzene-1,2-diamine (**10**)

Compound **10** was prepared using 7-(bromomethyl)quinoline hydrobromide salt (0.39 g, 1.3 mmol) according to the general procedure described above. Red solid, 0.19 g (66% yield); *R*_f_ = 0.10 (Hexanes/EtOAc 1:1 v/v); ^1^H NMR (300 MHz, DMSO-*d*_*6*_): δ 8.86 (dd, *J* = 4.3 and 1.7 Hz, 1H), 8.33 (dd, *J* = 8.2 and 1.7 Hz, 1H), 7.97 (s, 1H), 7.95 (d, *J* = 8.2 Hz, 1H), 7.63 (dd, *J* = 8.5 and 1.7 Hz, 1H), 7.49 (dd, *J* = 8.2 and 4.2 Hz, 1H), 7.31 (dd, *J* = 8.5 and 1.7 Hz, 1H), 7.24–7.19 (m, 2H), 6.59 (d, *J* = 8.2 Hz, 1H), 6.49–6.41 (m, 1H), 4.61 (s, 2H); ^13^C NMR (75 MHz, DMSO-*d*_*6*_): δ 150.6, 147.8, 140.9, 137.1, 135.8, 135.8, 130.9, 128.2, 127.0, 126.4, 126.3, 121.1, 115.7, 113.4, 112.8, 46.8; ESI-HRMS (*m*/*z*): calcd. for C_16_H_14_N_4_O_2_Na [M + Na]^+^ 317.1009; found 317.1009; calcd. for C_16_H_15_N_4_O_2_ [M + H]^+^ 295.1190; found 295.1189.

#### General Procedure for the Cyclization of Diamines **8** and **9**

To an ice-cold solution of trimethyl orthoformate (0.13 mL, 1.2 mmol) in DCM (4 mL), BF_3_·Et_2_O (0.1 mL, 0.8 mmol) was added dropwise, followed by the addition of **8** or **9** (0.4 mmol). The resulting mixture was stirred at RT for 5 h and quenched with sat. aqueous NaHCO_3_. The aqueous phase was extracted twice with DCM. The combined organic layers dried over Na_2_SO_4_ and evaporated to dryness. Compounds **11** and **12** were obtained in pure form after FCC purification.

#### 1-(Naphthalen-2-ylmethyl)-4-nitro-1*H*-benzo[*d*]imidazole (**11**) (**CKP-20**)

Compound **11** was prepared starting from **8** (0.12 g, 0.4 mmol) according to the general procedure described above. White solid, 0.114 g (94% yield); *R*_f_ = 0.14 (Hexanes/EtOAc 1:1 v/v); ^1^H NMR (600 MHz, acetone-*d*_*6*_): δ 8.64 (s, 1H), 8.03 (dd, *J* = 7.8 and 1.2 Hz, 2H), 7.96 (dd, *J* = 8.4 and 1.2 Hz, 1H), 7.92–7.86 (m, 3H), 7.58–7.49 (m, 2H), 7.48 (dd, *J* = 8.5 and 1.9 Hz, 1H), 7.39 (t, *J* = 8.1 Hz, 1H), 5.87 (s, 2H); ^13^C NMR (150 MHz, acetone-*d*_*6*_): δ 148.3, 140.5, 138.2, 137.8, 134.5, 134.3, 133.9, 129.7, 128.7, 128.6, 127.4, 127.3, 127.2, 126.0, 122.8, 119.2, 118.1, 49.7; ESI-HRMS (*m*/*z*): calcd. for C_18_H_14_N_3_O_2_ [M + H]^+^ 304.1086; found 304.1076. HPLC analysis: *t*_R_ = 6.70 min, purity = 99.7%.

#### 1-([1,1′-Biphenyl]-4-ylmethyl)-4-nitro-1*H*-benzo[*d*]imidazole (**12**) (**CKP-21**)

Compound **12** was prepared starting from **9** (0.13 g, 0.4 mmol) according to the general procedure A described above. White solid, 0.121 g (92% yield); *R*_f_ = 0.15 (Hexanes/EtOAc 1:1 v/v); ^1^H NMR (600 MHz, acetone-*d*_*6*_): δ 8.61 (s, 1H), 8.05 (d, *J* = 8.4 Hz, 1H), 7.98 (d, *J* = 8.4 Hz, 1H), 7.67–7.64 (m, 2H), 7.63–7.60 (m, 2H), 7.48–7.42 (m, 5H), 7.37–7.33 (m, 1H), 5.75 (s, 2H); ^13^C NMR (150 MHz, acetone-*d*_*6*_): δ 148.2, 141.7, 141.0, 140.6, 138.2, 137.7, 136.2, 129.7 (two C), 128.9 (two C), 128.4, 128.2 (two C), 127.7 (two C), 122.8, 119.2, 118.0, 49.1; ESI-HRMS (*m*/*z*): calcd. for C_20_H_16_N_3_O_2_ [M + H]^+^ 330.1243; found 330.1232. HPLC analysis: *t*_R_ = 7.41 min, purity = 100%.

#### 7-((4-Nitro-1*H*-benzo[*d*]imidazol-1-yl)methyl)quinoline (**13**) (**CKP-48**)

A mixture of **10** (0.12 g, 0.4 mmol), trimethyl orthoformate (1.2 mmol) and catalytic amount of *p*-TsOH·H_2_O (0.08 mmol) in toluene (0.1 M) was stirred at 80 °C for 2 h. White solid, 0.106 g (87% yield); *R*_f_ = 0.25 (DCM/EtOAc 5:3 v/v); ^1^H NMR (600 MHz, acetone-*d*_*6*_): δ 8.90 (s, 1H), 8.68 (s, 1H), 8.32 (d, *J* = 8.5 Hz, 1H), 8.05 (dd, *J* = 8.1 and 2.6 Hz, 1H), 8.01 (d, *J* = 4.3 Hz, 2H), 7.96 (dd, *J* = 8.5 and 2.6 Hz, 1H), 7.56 (d, *J* = 8.1 Hz, 1H), 7.51 (dt, *J* = 8.1 and 3.4 Hz, 1H), 7.43 (dt, *J* = 8.1 and 4.3 Hz, 1H), 5.96 (s, 2H); ^13^C NMR (150 MHz, acetone-*d*_*6*_): δ 152.0, 149.1, 148.3, 140.6, 138.4, 138.2, 137.7, 136.6, 129.8, 128.7, 128.6, 126.4, 122.9, 122.6, 119.3, 118.1, 49.4; ESI-HRMS (*m*/*z*): calcd. for C_17_H_12_N_4_O_2_Na [M + Na]^+^ 327.0852; found 327.0853; calcd. for C_17_H_13_N_4_O_2_ [M + H]^+^ 305.1033; found 305.1032. HPLC analysis: *t*_R_ = 5.51 min, purity = 98%.

#### General Procedure A for Nitro Group Reduction

To a suspension of **3** or **4** or **11** or **12** or **13** (0.3 mmol) in gl. CH_3_CO_2_H (1.1 mL), conc. HCl (0.44 mL) was added followed by the addition of SnCl_2_·2H_2_O (0.45 g, 2 mmol). The reaction mixture stirred at RT for 30 min. Solvents were azeotropically removed with PhMe and to the residue, an aq. solution NaOH (10 M) and brine were added. The aqueous phase was extracted three times with DCM and the combined organic layers dried over Na_2_SO_4_ and evaporated to dryness to afford the desired compounds.

#### 1-(Naphthalen-2-ylmethyl)-1*H*-benzo[*d*]imidazol-5-amine (**5**) (**CKP-39**)

Compound **5** was prepared starting from **3** (0.09 g, 0.3 mmol) according to the general procedure A described above. White solid, 0.071 g (86% yield); *R*_f_ = 0.26 (DCM/MeOH 95:5 v/v); ^1^H NMR (600 MHz, acetone-*d*_*6*_): δ 8.08 (s, 1H), 7.90–7.84 (m, 4H), 7.81 (s, 1H), 7.52–7.48 (m, 2H), 7.40 (dd, *J* = 8.4 and 1.8 Hz, 1H), 7.14 (d, *J* = 8.4 Hz, 1H), 6.95 (d, *J* = 2.4 Hz, 1H), 5.59 (s, 2H); ^13^C NMR (150 MHz, acetone-*d*_*6*_): δ 145.9, 145.0, 144.1, 135.4, 134.3, 133.9, 129.5, 129.4, 128.7, 128.6, 128.5, 127.3, 127.2, 127.1, 116.7, 111.2, 110.3, 49.3; ESI-HRMS (*m*/*z*): calcd. for C_18_H_16_N_3_ [M + H]^+^ 274.1344; found 274.1334. HPLC analysis: *t*_R_ = 4.48 min, purity = 97.2%.

#### 1-([1,1′-Biphenyl]-4-ylmethyl)-1*H*-benzo[*d*]imidazol-5-amine (**6**) (**CKP-45**)

Compound **6** was prepared starting from **4** (0.1 g, 0.3 mmol) according to the general procedure A described above. White solid, 0.074 g (90% yield); *R*_f_ = 0.28 (DCM/MeOH 95:5 v/v); ^1^H NMR (300 MHz, DMSO-*d*_*6*_): δ 8.17 (s, 1H), 7.62 (d, *J* = 7.7 Hz, 4H), 7.44 (t, *J* = 7.5 Hz, 2H), 7.35 (d, *J* = 7.7 Hz, 3H), 7.18 (d, *J* = 8.6 Hz, 1H), 6.79 (d, *J* = 2.0 Hz, 1H), 6.56 (dd, *J* = 8.6, 2.0 Hz, 1H), 5.40 (s, 2H); ^13^C NMR (75 MHz, DMSO-*d*_*6*_): δ 144.9, 144.1, 143.3, 139.7, 139.5, 136.5, 128.9 (two C), 127.9 (two C), 127.5, 126.9 (two C), 126.7 (two C), 126.2, 112.3, 110.5, 102.6, 47.3; ESI-HRMS (*m*/*z*): calcd. for C_20_H_18_N_3_ [M + H]^+^ 300.1501; found 300.1489. HPLC analysis: *t*_R_ = 5.62 min, purity = 99.3%.

#### 1-(Naphthalen-2-ylmethyl)-1*H*-benzo[*d*]imidazol-4-amine (**14**) (**CKP-29**)

Compound **14** was prepared starting from **11** (0.09,0.3 mmol) according to the general procedure A described above. White solid, 0.073 g (89% yield); *R*_f_ = 0.25 (DCM/MeOH 97:3 v/v); ^1^H NMR (600 MHz, acetone-*d*_*6*_): δ 8.11 (s, 1H), 7.89–7.84 (m, 4H), 7.81 (s, 1H), 7.51–7.47 (m, 3H), 7.41 (dd, *J* = 8.4 and 1.8 Hz, 1H), 6.90 (t, *J* = 7.8 Hz, 1H), 6.69 (dd, *J* = 8.4 and 1.2 Hz, 1H), 6.44 (dd, *J* = 7.8 and 1.2 Hz, 1H), 5.60 (s, 2H); ^13^C NMR (150 MHz, acetone-*d*_*6*_): δ 143.8, 142.0, 141.2, 135.7, 134.3, 133.8, 129.4, 128.7, 128.5, 127.2, 127.0, 126.9, 126.1, 124.6, 123.8, 105.6, 99.6, 49.2; ESI-HRMS (*m*/*z*): calcd. for C_18_H_16_N_3_ [M + H]^+^ 274.1344; found 274.1333. HPLC analysis: *t*_R_ = 6.46 min, purity = 99%.

#### 1-([1,1′-Biphenyl]-4-ylmethyl)-1*H*-benzo[*d*]imidazol-4-amine (**15**) (**CKP-28**)

Compound **15** was prepared starting from **12** (0.1 g, 0.3 mmol) according to the general procedure A described above. White solid, 0.082 g (91% yield); *R*_f_ = 0.27 (EtOAc); ^1^H NMR (600 MHz, acetone-*d*_*6*_): δ 7.97 (s, 1H), 7.53–7.50 (m, 4H), 7.35–7.30 (m, 2H), 7.29–7.21 (m, 3H), 6.82 (t, *J* = 7.8 Hz, 1H), 6.60 (dd, *J* = 8.4 and 1.2 Hz, 1H), 6.34 (dd, *J* = 8.4 and 1.2 Hz, 1H), 5.39 (s, 2H), 4.85 (br.s, 2H); ^13^C NMR (150 MHz, acetone-*d*_*6*_): δ 141.9, 141.3, 141.2, 137.4 (two C), 135.6, 134.0, 129.7 (two C), 128.7 (two C), 128.3, 128.0 (two C), 127.7 (two C), 124.6, 105.6, 99.6, 48.6; ESI-HRMS (*m*/*z*): calcd. for C_20_H_18_N_3_ [M + H]^+^ 300.1501; found 300.1489. HPLC analysis: *t*_R_ = 7.36 min, purity = 99.9%.

#### 1-(Quinolin-7-ylmethyl)-1*H*-benzo[*d*]imidazol-4-amine (**16**) (**CKP-49**)

Compound **16** was prepared starting from **13** (0.09 g, 0.3 mmol) according to the general procedure A described above. White solid, 0.07 g (89% yield); *R*_f_ = 0.16 (DCM/Acetone 5:3 v/v); ^1^H NMR (600 MHz, acetone-*d*_*6*_): δ 8.88–8.86 (m, 1H), 8.29 (d, *J* = 8.5 Hz, 1H), 8.23 (s, 1H), 7.93 (d, *J* = 4.3 Hz, 1H), 7.91 (d, *J* = 8.5 Hz, 1H), 7.53–7.45 (m, 2H), 6.91 (t, *J* = 7.8 Hz, 1H), 6.72 (d, *J* = 8.1 Hz, 1H), 6.46 (d, *J* = 7.8 Hz, 1H), 5.72 (s, 2H); ^13^C NMR (150 MHz, acetone-*d*_*6*_): δ 151.8, 149.2, 142.1, 139.5, 136.5 (two C), 129.5 (two C), 128.5, 128.2, 126.5 (two C), 124.7, 122.3, 105.7, 99.5, 48.9; ESI-HRMS (*m*/*z*): calcd. for C_17_H_15_N_4_ [M + H]^+^ 275.1291; found 275.1287. HPLC analysis: *t*_R_ = 5.24 min, purity = 99.4%.

#### General Procedure for *N-*Alkylation of *tert*-Butyl (3-Methoxy-2-nitrophenyl)carbamate

NaH (60% w/w dispersion in mineral oil, 1.2 equiv) was added at 0 °C to a solution of *tert*-butyl (3-methoxy-2-nitrophenyl)carbamate **18** (1 equiv) in DMF (0.1 M). The resulting suspension was stirred at the same temperature for 20 min and then the appropriate bromide (1.2 equiv) was added. The reaction mixture was stirred at room temperature for 3 h. Upon completion, the reaction was cooled to 0 °C, quenched with sat. aq. NH_4_Cl and extracted with ethyl acetate. The organic layer was washed with brine, dried over Na_2_SO_4_, filtered and the solvent was evaporated under reduced pressure. The crude residue was used in the next step without further purification

#### *tert*-Butyl (3-Methoxy-2-nitrophenyl)(naphthalen-2-ylmethyl)carbamate (**19**)

Compound **19** was prepared using **18** (0.15 g, 0.56 mmol) and 2-(bromomethyl)naphthalene (0.15 g, 0.67 mmol) according to the above general procedure above. The compound was used in the next step without further purification, *R*_f_ = 0.35 (Hexanes/EtOAc 80:20 v/v).

#### *tert*-Butyl ([1,1′-Biphenyl]-4-ylmethyl)(3-methoxy-2-nitrophenyl)carbamate (**20**)

Compound **20** was prepared using **18** (0.15 g, 0.56 mmol) and 4-biphenylmethyl bromide (0.17 g, 0.67 mmol) according to the above general procedure above. The compound was used in the next step without further purification, *R*_f_ = 0.29 (Hexanes/EtOAc 80:20 v/v).

#### *tert*-Butyl (3-Methoxy-2-nitrophenyl)(quinolin-7-ylmethyl)carbamate (**21**)

Compound **21** was prepared using **18** (0.08 g, 0.30 mmol) and 7-(bromomethyl)quinoline hydrobromide (0.11 g, 0.36 mmol) according to the above general procedure above. The compound was used in the next step without further purification, *R*_f_ = 0.10 (Hexanes/EtOAc 60:40 v/v).

#### General Procedure for *N*-Boc Deprotection of *N-*Aryl-*tert*-butyl(3-methoxy-2-nitrophenyl)carbamate

To a solution of *tert*-butyl carbamate **19** or **20** or **21** (1 equiv) in dry DCM (0.1M) was added TFA (20 equiv) at 0 °C and the reaction mixture was stirred at RT for 1 h. The solvent was evaporated in vacuo and the residue was diluted with sat. aq. NaHCO_3_ and extracted with EtOAc. The organic phase was washed with brine, dried over Na_2_SO_4_, filtered and evaporated in vacuo. The residue was purified by FCC to afford the desired compound.

#### 3-Methoxy-*N*-(naphthalen-2-ylmethyl)-2-nitroaniline (**22**)

The title compound **22** was synthesized from **19** (0.23 g, 0.56 mmol) following the above general procedure. The desired product was obtained after FCC (Hexanes/EtOAc 8:2 v/v) as a yellow solid, 0.14 g, (79% yield over 2 steps from **18**); mp: 111–112 °C; ^1^H NMR (600 MHz, CDCl_3_): δ 7.86–7.72 (m, 4H), 7.55–7.37 (m, 3H), 7.16 (t, *J* = 8.4 Hz, 1H), 6.37 (d, *J* = 8.6 Hz, 1H), 6.29 (d, *J* = 8.3 Hz, 1H), 4.59 (s, 2H), 3.88 (s, 3H); ^13^C NMR (150 MHz, CDCl_3_): δ 155.2, 144.0, 135.4, 133.7, 133.6, 133.0, 128.8, 127.9, 127.8, 126.5, 126.1, 125.7, 125.2, 105.8, 100.3, 56.6, 47.8; ESI-HRMS (*m*/*z*): calcd. for C_18_H_17_N_2_O_3_ [M + H]^+^ 309.1234; found 309.1228.

#### *N*-([1,1′-Biphenyl]-4-ylmethyl)-3-methoxy-2-nitroaniline (**23**)

The title compound **23** was synthesized from **20** (0.24 g, 0.56 mmol) following the above general procedure. The desired product was obtained after FCC (Hexanes/EtOAc 8:2 v/v) as an orange solid, 0.14 g (77% over 2 steps from **18**); mp: 129–130 °C; ^1^H NMR (600 MHz, acetone-*d*_*6*_): δ 7.73–7.60 (m, 4H), 7.50–7.42 (m, 4H), 7.37–7.32 (m, 1H), 7.22 (t, *J* = 8.4 Hz, 1H), 6.45 (t, *J* = 7.7 Hz, 3H), 4.59 (d, *J* = 5.9 Hz, 2H), 3.86 (s, 3H); ^13^C NMR (150 MHz, acetone-*d*_*6*_): δ 154.7, 142.9, 141.5, 140.7, 139.2, 133.5, 129.7, 128.4, 128.1, 127.9, 127.6, 106.5, 100.8, 56.7, 47.1; ESI-MS (*m*/*z*): 357.34 (M+Na)^+^, 690.80 (2M+Na)^+^.

#### 3-Methoxy-2-nitro-*N*-(quinolin-7-ylmethyl)aniline (**24**)

The title compound **24** was synthesized from **21** (0.11 g, 0.27 mmol) following the above general procedure. The desired product was obtained as an orange solid, 0.07 g, (80% over 2 steps from **18**); mp: 176–177 °C; ^1^H NMR (600 MHz, CDCl_3_): δ 9.01–8.79 (m, 1H), 8.18 (d, *J* = 8.3 Hz, 1H), 8.08 (s, 1H), 7.83 (d, *J* = 8.5 Hz, 1H), 7.54 (d, *J* = 8.4 Hz, 1H), 7.42 (dd, *J* = 8.5 and 4.1 Hz, 1H), 7.15 (t, *J* = 8.4 Hz, 1H), 6.72 (s, 1H), 6.34 (d, *J* = 8.6 Hz, 1H), 6.29 (d, *J* = 8.3 Hz, 1H), 4.67 (d, *J* = 5.8 Hz, 2H), 3.88 (s, 3H); ^13^C NMR (75 MHz, CDCl_3_): δ 154.8, 149.7, 143.3, 136.6, 133.3, 128.3, 127.4, 126.2, 125.7, 120.8, 105.2, 100.2, 56.2, 47.1; ESI-HRMS (*m*/*z*): calcd. for C_17_H_16_N_3_O_3_ [M + H]^+^ 310.1186; found 310.1177.

#### General Procedure B for Nitro Group Reduction

SnCl_2_ (5 equiv) was added to a mixture of the nitro compound **22** or **23** or **24** (1 equiv) in MeOH (0.1M) and conc. HCl (70 equiv) and stirred at 50 °C overnight. The mixture was concentrated in vacuo. The residue was taken up in EtOAc and washed with sat. aq. NaHCO_3_, brine, dried over Na_2_SO_4_, filtered and evaporated in vacuo. The crude was purified by FCC to give the desired compound.

#### 3-Methoxy-*N*^1^-(naphthalen-2-ylmethyl)benzene-1,2-diamine (**25**)

The title compound **25** was prepared starting from **22** (0.12 g, 0.39 mmol) according to the above general procedure B. Purification of the compound with FCC (Hexanes/EtOAc 8:2 v/v) gave a brown oil, 0.089 g (82% yield); *R*_f_ = 0.25 (Hexanes/EtOAc 8:2 v/v); ^1^H NMR (600 MHz, CDCl_3_): δ 7.89–7.74 (m, 4H), 7.57–7.35 (m, 3H), 6.79–6.75 (m, 1H), 6.45 (t, *J* = 8.1 Hz, 2H), 4.51 (s, 2H), 3.87 (s, 3H), 3.70 (bs, 3H); ^13^C NMR (150 MHz, CDCl_3_): δ 148.4, 137.1, 133.6, 132.9, 128.4, 127.9, 127.8, 126.2, 126.2, 126.1, 125.8, 122.9, 119.8, 105.8, 102.1, 55.9, 49.1; ESI-MS (*m*/*z*): 279.22 (M+H)^+^.

#### *N*^1^-([1,1′-Biphenyl]-4-ylmethyl)-3-methoxybenzene-1,2-diamine (**26**)

The title compound **26** was prepared starting from **23** (0.120 g, 0.36 mmol) according to the above general procedure B. Purification of the compound with FCC (Hexanes/EtOAc 8:2 v/v) gave a brown oil, 0.082 g (75% yield); *R*_f_ = 0.20 (Hexanes/EtOAc 8:2 v/v); ^1^H NMR (600 MHz, CDCl_3_): δ 7.61 (dd, *J* = 11.8 and 8.0 Hz, 4H), 7.48 (dd, *J* = 16.5 and 8.0 Hz, 4H), 7.38 (t, *J* = 7.4 Hz, 1H), 6.80 (t, *J* = 8.1 Hz, 1H), 6.47 (dd, *J* = 14.8 and 8.1 Hz, 2H), 4.41 (s, 2H), 3.88 (s, 3H), 3.68 (bs, 3H); ^13^C NMR (150 MHz, CDCl_3_): δ 148.4, 141.0, 140.3, 138.5, 138.0, 128.9, 128.3, 127.4, 127.3, 127.2, 122.9, 119.8, 105.8, 102.2, 55.9, 48.6; ESI-HRMS (*m*/*z*): calcd. for C_20_H_21_N_2_O [M + H]^+^ 305.1648; found 305.1649.

#### 3-Methoxy-*N*^1^-(quinolin-7-ylmethyl)benzene-1,2-diamine (**27**)

The title compound **27** was prepared starting from **24** (0.058 g, 0.19 mmol) according to the above general procedure B. Purification of the compound with FCC (Hexanes/EtOAc 7:3 to 6:4 v/v) gave a brown oil, 0.043 g (83% yield); *R*_f_ = 0.08 (Hexanes/EtOAc 6:4 v/v); ^1^H NMR (600 MHz, CDCl_3_): δ 9.02–8.83 (m, 1H), 8.24–8.05 (m, 2H), 7.80 (d, *J* = 8.4 Hz, 1H), 7.61 (d, *J* = 8.1 Hz, 1H), 7.40 (dd, *J* = 8.2 and 4.3 Hz, 1H), 6.72 (t, *J* = 8.2 Hz, 1H), 6.41 (dd, *J* = 22.6 and 8.2 Hz, 2H), 4.57 (s, 2H), 3.85 (s, 3H), 3.54 (bs, 3H); ^13^C NMR (150 MHz, CDCl_3_): δ 150.2, 148.4, 147.9, 141.6, 137.9, 136.2, 128.0, 127.4, 127.0, 126.6, 122.7, 120.8, 119.7, 105.5, 102.0, 55.7, 48.5; ESI-HRMS (*m*/*z*): calcd. for C_17_H_18_N_3_O [M + H]^+^ 280.1444; found 280.1437.

#### General Procedure B for Cyclization Diamines **25–27**

A mixture of the diamine **25** or **26** or **27** (1 equiv), trimethyl orthoformate (3 equiv) and catalytic amount of *p*-TsOH·H_2_O (0.2 equiv) in toluene (0.1 M) was stirred at 80 °C for 2–18 h. After completion of the reaction the solvent was removed in vacuo. The residue was purified by FCC to afford the desired compound.

#### 4-Methoxy-1-(naphthalen-2-ylmethyl)-1*H*-benzo[*d*]imidazole (**28**) (**CKP-25**)

Compound **28** was prepared using **25** (0.088 g, 0.31 mmol) according to the general procedure B described above. Purification with FCC (DCM/Acetone 9:1 v/v) gave a white solid, 0.061 g (67% yield); *R*_f_ = 0.40 (DCM/Acetone 9:1 v/v); mp: 163–164 °C; ^1^H NMR (600 MHz, acetone-*d*_*6*_): δ 8.21 (s, 1H), 7.90–7.81 (m, 4H), 7.49 (dd, *J* = 6.5 and 2.9 Hz, 2H), 7.42 (d, *J* = 8.4 Hz, 1H), 7.11–7.05 (m, 2H), 6.69 (d, *J* = 7.5 Hz, 1H), 5.68 (s, 2H), 4.00 (s, 3H); ^13^C NMR (150 MHz, acetone-*d*_*6*_): δ 153.0, 143.3, 137.0, 135.6, 134.4, 134.0, 129.6, 128.8, 128.7, 127.4, 127.1, 127.1, 126.1, 124.3, 104.6, 104.2, 56.5, 49.3; ESI-HRMS (*m*/*z*): calcd for C_19_H_17_N_2_O [M + H]^+^ 289.1335, found 289.1336. HPLC analysis: *t*_R_ = 5.89 min, purity = 100%.

#### 1-([1,1′-Biphenyl]-4-ylmethyl)-4-methoxy-1*H*-benzo[*d*]imidazole (**29**) (**CKP-27**)

Compound **29** was prepared using **26** (0.080 g, 0.26 mmol) according to the general procedure B described above. Purification with FCC (DCM/Acetone 8:2 v/v) gave a white solid, 0.062 g (76% yield); *R*_f_ = 0.35 (DCM/Acetone 9:1 v/v); mp: 150–151 °C. ^1^H NMR (600 MHz, acetone-*d*_*6*_): δ 8.18 (s, 1H), 7.63 (dd, *J* = 7.9 and 4.1 Hz, 4H), 7.44 (t, *J* = 7.6 Hz, 2H), 7.39 (d, *J* = 7.9 Hz, 2H), 7.35 (t, *J* = 7.3 Hz, 2H), 7.13 (t, *J* = 7.9 Hz, 1H), 7.08 (d, *J* = 7.8 Hz, 1H), 6.71 (d, *J* = 7.8 Hz, 1H), 5.57 (s, 3H), 4.01 (s, 4H); ^13^C NMR (150 MHz, acetone-*d*_*6*_): δ 152.0, 142.2, 140.3, 136.2, 128.8, 127.8, 127.4, 127.2, 126.8, 123.3, 103.6, 103.2, 55.5, 47.8; ESI-HRMS (*m*/*z*): calcd for C_21_H_19_N_2_O [M + H]^+^ 315.1492, found 315.1494. HPLC analysis: *t*_R_ = 5.98 min, purity = 99.3%.

#### 7-((4-Methoxy-1*H*-benzo[d]imidazol-1-yl)methyl)quinoline (**30**) (**CKP-44**)

Compound **30** was prepared using **27** (0.026 g, 0.093 mmol) according to the general procedure B described above. Purification with FCC (DCM/Acetone 8:2 v/v) gave a white solid, 0.020 g (74% yield); *R*_f_ = 0.25 (DCM/Acetone 9:1 v/v); mp: 161–162 °C; ^1^H NMR (600 MHz, acetone-*d*_*6*_): δ 8.88 (dd, *J* = 4.3 and 1.8 Hz, 1H), 8.32–8.23 (m, 2H), 7.98–7.91 (m, 2H), 7.50 (ddd, *J* = 14.1, 8.4, and 3.0 Hz, 2H), 7.16–7.07 (m, 2H), 6.72 (dd, *J* = 7.5 and 1.3 Hz, 1H), 5.78 (s, 2H), 4.01 (s, 3H); ^13^C NMR (150 MHz, acetone-*d*_*6*_): δ 152.9, 151.8, 149.1, 143.2, 139.3, 136.8, 136.6, 129.6, 128.6, 128.2, 126.5, 124.4, 122.4, 104.6, 104.1, 56.4, 49.0; ESI-HRMS (*m*/*z*): calcd for C_18_H_16_N_3_O [M + H]^+^ 290.1288, found 290.1292. HPLC analysis: *t*_R_ = 5.17 min, purity = 99.8%.

#### General Procedure for Methoxy Group Removal

To an ice-cold solution of the **28** or **29** (1 equiv) in DCM (0.05 Μ), BF_3_S(CH_3_)_2_ (10 equiv, per methoxy group) was added and the reaction mixture was stirred at RT for 2 d. After completion of the reaction, the solvent and excess reagent were evaporated under argon stream. To the residue sat. aq. NaHCO_3_ was added and extracted with ethyl acetate. The organic layer was washed with sat. aq. NaCl, dried over Na_2_SO_4_ and the solvent was evaporated in vacuo. The residue was purified by FCC to obtain the desired compound.

#### 1-(Naphthalen-2-ylmethyl)-1*H*-benzo[*d*]imidazol-4-ol (**31**) (**CKP-26**)

Compound **31** was synthesized from **28** (0.026 g, 0.09 mmol) following the above general method. Purification with FCC (DCM/Acetone 9:1 v/v) gave a white solid, 0.015 g (60% yield); *R*_f_ = 0.28 (DCM/Acetone 8:2 v/v); mp: 235–236 °C; ^1^H NMR (600 MHz, CD_3_OD): δ 8.19 (s, 1H), 7.85–7.78 (m, 3H), 7.72 (s, 2H), 7.47 (dd, *J* = 5.9 and 3.3 Hz, 1H), 7.35 (d, *J* = 8.5 Hz, 1H), 7.04 (t, *J* = 7.9 Hz, 1H), 6.91 (d, *J* = 8.2 Hz, 1H), 6.63 (d, *J* = 7.7 Hz, 1H), 5.61 (s, 2H); ^13^C NMR (150 MHz, CD_3_OD): δ 150.4, 143.4, 137.1, 135.2, 134.8, 134.4, 133.9, 129.8, 128.9, 128.7, 127.5, 127.3, 127.2, 125.9, 125.3, 108.2, 103.0, 49.8; ESI-HRMS (*m*/*z*): calcd for C_18_H_15_ON_2_ [M + H]^+^ 275.1179, found 275.1182.; HPLC analysis: *t*_R_ = 5.53 min, purity = 99.4%.

#### 1-([1,1′-Biphenyl]-4-ylmethyl)-1*H*-benzo[*d*]imidazol-4-ol (**32**) (**CKP-30**)

Compound **32** was synthesized from **29** (0.039 g, 0.12 mmol) following the above general method. Purification with FCC (DCM/Acetone 9:1 v/v) gave an off-white solid, 0.023 g, (62% yield); *R*_f_ = 0.20 (DCM/Acetone 8:2 v/v); mp: 258–259 °C; ^1^H NMR (600 MHz, CDCl_3_): δ 8.31 (s, 1H), 7.55 (dd, *J* = 20.7 and 7.8 Hz, 4H), 7.42 (t, *J* = 7.6 Hz, 2H), 7.36–7.32 (m, 1H), 7.29 (d, *J* = 7.8 Hz, 2H), 7.23–7.19 (m, 2H), 6.88 (dd, *J* = 22.3 and 8.0 Hz, 2H), 5.42 (s, 2H); ^13^C NMR (150 MHz, CDCl_3_): δ 147.6, 141.6, 139.9, 132.8, 128.8, 127.8, 127.8, 127.6, 126.9, 125.9, 109.7, 101.8, 49.4; ESI-HRMS (*m*/*z*): calcd for C_20_H_17_ON_2_ [M + H]^+^ 301.1335, found 301.1339. HPLC analysis: *t*_R_ = 5.55 min, purity = 100%.

#### General Procedure for *N-*Alkylation of 1*H*-Benzo[*d*]imidazole

To an ice-cold solution of 1*H*-benzo[*d*]imidazole (**1**) (1 equiv) in THF (0.3 M), NaH (60% w/w dispersion in mineral oil, 1.2 equiv) was added portion wise. The reaction was warmed to RT and stirred at the same temperature for 30 min followed by the addition of the appropriate bromide or mesylate (1.05 equiv). The reaction mixture was stirred at RT for 17 h, diluted with water and extracted twice with EtOAc. The combined organic layers were washed with water, dried over Na_2_SO_4_ and evaporated to dryness. Pure compound was obtained after FCC.

#### 1-(Naphthalen-2-ylmethyl)-1*H*-benzo[*d*]imidazole^122^ (**CKP-22**)

Compound **CKP-22** was prepared according to the above general procedure using 1 (0.08 g, 0.66 mmol) and 2-(bromomethyl)naphthalene (0.15 g, 0.69 mmol). The desired product was obtained after FCC (Hexanes/EtOAc 1:1 v/v) as a white solid 0.17 g (99% yield); mp: 139–140 °C; ^1^H NMR (600 MHz, acetone-*d*_*6*_): δ 8.32 (s, 1H), 7.95–7.80 (m, 4H), 7.73–7.65 (m, 1H), 7.54–7.47 (m, 3H), 7.44 (dd, *J* = 8.5 and 1.8 Hz, 1H), 7.19 (tt, *J* = 7.2 and 5.5 Hz, 2H), 5.72 (s, 2H); ^13^C NMR (150 MHz, acetone-*d*_*6*_): δ 145.3, 144.8, 135.4, 135.1, 134.3, 133.9, 129.5, 128.7, 128.5 127.3, 127.1, 126.1, 123.3, 122.5, 120.7, 111.3, 49.2.

#### 1-(2-(Naphthalen-2-yl)ethyl)-1*H*-benzo[*d*]imidazole (**33**) (**CKP-46**)

Compound **33** was prepared according to the above general procedure using **1** (0.08 g, 0.66 mmol) and 2-(2-bromoethyl)naphthalene (0.19 g, 0.79 mmol). The desired product was obtained after FCC (Hexanes/EtOAc 2:1 v/v) as an off-white solid, 0.04 g (24% yield); mp: 139–142 °C; ^1^H NMR (600 MHz, acetone-*d*_6_): δ 7.90 (s, 1H), 7.88–7.83 (m, 1H), 7.82 (d, *J* = 8.4 Hz, 1H), 7.78–7.74 (m, 1H), 7.68–7.62 (m, 3H), 7.49–7.42 (m, 2H), 7.38 (dd, *J* = 8.4 and 1.9 Hz, 1H), 7.27–7.24 (m, 1H), 7.23–7.19 (m, 1H), 4.68 (t, *J* = 7.3 Hz, 2H), 3.38 (t, *J* = 7.3 Hz, 2H); ^13^C NMR (150 MHz, acetone-*d*_*6*_): δ 144.4, 136.8, 133.3, 129.0, 128.4, 128.3, 128.2, 128.0, 126.9, 126.4, 123.2, 122.3, 120.6, 111.0, 46.7, 36.9; ESI-HRMS (*m*/*z*): calcd. for C_19_H_17_N_2_ [M + H]^+^ 273.1386; found 273.1384. HPLC analysis: *t*_R_ = 4.16 min, purity = 99.6%.

#### 1-([1,1′-Biphenyl]-4-ylmethyl)-1*H*-benzo[*d*]imidazole (**34**) (**CKP-23**)^123^

Compound **34** was prepared according to the above general procedure using **1** (0.07 g, 0.60 mmol) and 4-biphenylmethyl bromide (0.15 g, 0.63 mmol). The desired product was obtained after FCC (Hexanes/EtOAc 1:1 v/v) as a white solid, 0.16 g (95% yield); mp: 184–186 °C; ^1^H NMR (600 MHz, acetone-*d*_6_): δ 8.30 (s, 1H), 7.73–7.67 (m, 1H), 7.66–7.60 (m, 4H), 7.56–7.48 (m, 1H), 7.48–7.39 (m, 4H), 7.39–7.29 (m, 1H), 7.22 (dd, *J* = 6.5 and 2.7 Hz, 2H), 5.61 (s, 2H); ^13^C NMR (150 MHz, acetone-*d*_*6*_): δ 145.3, 144.4, 141.4, 141.2, 137.0, 135.0, 129.7, 128.7, 128.3, 128.1, 127.7, 123.3, 122.5, 120.7, 111.3, 48.6.^123^

#### 7-((1*H*-Benzo[*d*]imidazol-1-yl)methyl)quinoline (**35**) (**CKP-36**)

Compound **35** was prepared according to the above general procedure using **1** (0.08 g, 0.66 mmol) and 7-(bromomethyl)quinoline hydrobromide (0.22 g, 0.72 mmol). The desired product was obtained after FCC (Hexanes/EtOAc 1:1 v/v) as a beige solid, 0.05 g (27% yield); mp: 166–168 °C decompose; ^1^H NMR (600 MHz, CDCl_3_): δ 8.92 (dd, *J* = 4.3 and 1.8 Hz, 1H), 8.19 (s, 1H), 8.14 (dd, *J* = 8.4 and 1.8 Hz, 1H), 8.00 (s, 1H), 7.85 (d, *J* = 8.0 Hz, 1H), 7.79 (d, *J* = 8.4 Hz, 1H), 7.42 (dd, *J* = 8.3 and 4.2 Hz, 1H), 7.33 (td, *J* = 5.2 and 2.5 Hz, 2H), 7.31–7.18 (m, 2H), 5.60 (s, 2H); ^13^C NMR (150 MHz, CDCl_3_): δ 151.3, 148.2, 143.2, 136.9, 136.1, 129.2, 128.1, 127.9, 125.4, 123.7, 122.9, 121.8, 120.4, 110.3, 49.2; ESI-HRMS (*m*/*z*): calcd. for C_17_H_14_N_3_ [M + H]^+^ 260.1182; found 260.1178. HPLC analysis: *t*_R_ = 3.05 min, purity = 100%.

#### 1-(3,4-Dimethoxybenzyl)-1*H*-benzo[*d*]imidazole (**36**) (**CKP-24**)^124^

Compound 36 was prepared according to the above general procedure using **1** (0.15 g, 1.30 mmol) and 3,4-dimethoxybenzyl methanesulfonate (0.34 g, 1.37 mmol). The desired product was obtained after FCC (Hexanes/EtOAc 1:1 v/v) as a white solid, 0.17 g (49% yield); mp: 131–132 °C; ^1^H NMR (600 MHz, acetone-*d*_6_): δ 8.21 (s, 1H), 7.72–7.60 (m, 1H), 7.56–7.44 (m, 1H), 7.26–7.14 (m, 2H), 7.05 (s, 1H), 6.95–6.82 (m, 2H), 5.44 (s, 2H), 3.76 (d, *J* = 6.6 Hz, 6H); ^13^C NMR (150 MHz, acetone-*d*_*6*_): δ 150.5, 150.1, 145.3, 144.5, 135.0, 130.1, 123.1, 122.3, 120.8, 120.6, 112.6, 112.4, 111.4, 56.0, 55.9, 48.8.

#### 4-((1*H*-Benzo[*d*]imidazol-1-yl)methyl)benzene-1,2-diol (**37**) (**CKP-35**)

Compound **37** was synthesized from **36** (0.050 g, 0.19 mmol) following the above general method. Purification with FCC (DCM/MeOH 9:1 v/v) gave a white solid, 0.017 g (37% yield); mp: 188–190 °C (dec.); ^1^H NMR (600 MHz, DMSO-*d*_6_): δ 8.92 (d, *J* = 10.7 Hz, 2H), 8.48 (s, 1H), 7.66 (d, *J* = 7.6 Hz, 1H), 7.54 (d, *J* = 7.6 Hz, 1H), 7.34–7.18 (m, 2H), 6.51–6.89 (m, 3H), 5.32 (s, 2H); ^13^C NMR (150 MHz, DMSO-*d*_*6*_): δ 145.4, 145.0, 143.8, 133.4, 127.4, 122.6, 122.0, 118.9, 118.7, 115.5, 114.9, 111.1, 47.7; ESI-HRMS (*m*/*z*): calcd. for C_14_H_13_N_2_O_2_ [M + H]^+^ 241.0972; found 241.0971. HPLC analysis: *t*_R_ = 3.63 min, purity = 99.6%.

#### General Procedure for the Quaternization

To a solution of the appropriate *N*^1^-substituted benzimidazole **28** or **29** or **CKP-22** or **33**, or **34** or **36** (1 equiv) in 1,4-dioxane (0.03 M) the appropriate alkylating agent (1.3 equiv) was added and the resulting mixture was stirred at 100 °C for 24 h. Then, the reaction was cooled to RT. The precipitate was filtered and the solid was washed with dioxane and diethyl ether. The resulting compound was pure as obtained or was recrystallized from acetone when necessary.

#### (*E*)-1-(Naphthalen-2-ylmethyl)-3-(3-(4-nitrophenyl)allyl)-1*H*-benzo[*d*]imidazol-3-ium Bromide (**38**) (**CKP-31**)

The title compound **38** was prepared according to the above general procedure using 1-(naphthalen-2-ylmethyl)-1*H*-benzo[*d*]imidazole **CKP-22** (0.022 g, 0.082 mmol) and (*E*)-1-(3-bromoprop-1-en-1-yl)-4-nitrobenzene (0.026 g, 0.107 mmol). Pure compound was obtained as off-white solid, 0.031 g (75% yield); mp: 189–191 °C; ^1^H NMR (600 MHz, CD_3_OD): δ 9.76 (s, 1H), 8.21 (d, *J* = 8.9 Hz, 2H), 8.10–8.00 (m, 2H), 7.95 (dd, *J* = 8.4 and 4.2 Hz, 2H), 7.89 (dd, *J* = 6.5 and 3.4 Hz, 2H), 7.73–7.66 (m, 3H), 7.56 (ddd, *J* = 13.6, 7.4, and 2.6 Hz, 2H), 7.04 (d, *J* = 15.9 Hz, 1H), 6.81 (dt, *J* = 15.9 and 6.4 Hz, 1H), 5.95 (s, 2H), 5.42 (d, *J* = 5.9 Hz, 2H); ^13^C NMR (150 MHz, CD_3_OD): δ 149.0, 143.4, 135.3, 134.9, 134.8, 133.2, 133.1, 131.8, 130.5, 129.2, 129.1, 128.9, 128.8, 128.53, 128.50, 128.1, 128.0, 126.9, 126.3, 125.0, 115.1, 114.9, 52.3, 50.2; ESI-HRMS (*m*/*z*): calcd for C_27_H_22_N_3_O_2_ (M – Br)^+^ 420.1706; found 420.1704; HPLC analysis: *t*_R_ = 5.57 min, purity = 98.9%.

#### (*E*)-3-(3-(4-Chlorophenyl)allyl)-1-(naphthalen-2-ylmethyl)-1*H*-benzo[*d*]imidazol-3-ium Bromide (**39**) (**CKP-32**)

The title compound **39** was prepared according to the above general procedure using 1-(naphthalen-2-ylmethyl)-1*H*-benzo[*d*]imidazole **CKP-22** (0.021 g, 0.081 mmol) and (*E*)-1-(3-bromoprop-1-en-1-yl)-4-chlorobenzene (0.025 g, 0.106 mmol). Pure compound was obtained as white solid, 0.03 g (74% yield); Solid purification by recrystallization with acetone; mp: 166–167 °C; ^1^H NMR (600 MHz, CD_3_OD): δ 8.03 (d, *J* = 6.1 Hz, 1H), 7.94 (t, *J* = 7.0 Hz, 1H), 7.89 (t, *J* = 4.9 Hz, 1H), 7.69 (dt, *J* = 7.7 and 24.2 Hz, 1H), 7.52–7.59 (m, 2H), 7.47 (d, *J* = 8.1 Hz, 1H), 7.35 (d, *J* = 8.6 Hz, 1H), 6.93 (d, *J* = 15.9 Hz, 1H), 6.57 (dt, *J* = 6.5 and 14.1 Hz, 1H), 5.93 (s, 1H), 5.49 (s, 2H), 5.34 (d, *J* = 6.6 Hz, 2H); ^13^C NMR (150 MHz, CD_3_OD): δ 136.7, 135.7, 135.5, 134.9, 134.8, 133.2, 133.1, 131.8, 130.5, 129.9, 129.4, 129.1, 128.9, 128.5, 128.1, 128.0, 126.2, 122.6, 115.0, 114.9, 52.2, 50.4; ESI-HRMS (*m*/*z*): calcd for C_27_H_22_ClN_2_ (M – Br)^+^ 409.1466; found 409.1461; HPLC analysis: *t*_R_ = 5.84 min, purity = 100%.

#### (*E*)-1-(2-(Naphthalen-2-yl)ethyl)-3-(3-(4-nitrophenyl)allyl)-1*H*-benzo[*d*]imidazol-3-ium Bromide (**40**) (**CKP-47**)

The title compound **40** was prepared according to the above general procedure using 1-(2-(naphthalen-2-yl)ethyl)-1*H*-benzo[*d*]imidazole **33** (**CKP-46**) (0.020 g, 0.073 mmol) and (*E*)-1-(3-bromoprop-1-en-1-yl)-4-nitrobenzene (0.023 g, 0.095 mmol). Pure compound was obtained as off-white solid, 0.021 g (54% yield); mp: 176–177 °C decomposition; ^1^H NMR (600 MHz, CD_3_OD): δ 8.20 (d, *J* = 8.8 Hz, 2H), 8.07–8.03 (m, 1H), 8.00–7.97 (m, 1H), 7.81–7.70 (m, 5H), 7.65 (d, *J* = 7.9 Hz, 1H), 7.53–7.48 (m, 2H), 7.46 (s, 1H), 7.40 (dddd, *J* = 18.1 and 8.2 and 6.9 and 1.4 Hz, 2H), 7.28 (dd, *J* = 8.4 and 1.8 Hz, 1H), 6.75 (d, *J* = 15.9 Hz, 1H), 6.42 (dt, *J* = 15.8, 6.4 Hz, 1H), 5.22 (d, *J* = 6.4 Hz, 2H), 4.95 (t, *J* = 6.8 Hz, 2H), 3.49 (t, *J* = 6.7 Hz, 2H); ^13^C NMR (150 MHz, CD_3_OD): δ 149.0, 143.1, 135.3, 135.2, 134.9, 134.0, 132.82, 132.79, 129.9, 128.8, 128.7, 128.6, 128.52, 128.50, 127.7, 127.5, 127.1, 126.4, 124.9, 114.8, 114.7, 49.8, 49.6, 36.1; ESI-HRMS (*m*/*z*): calcd for C_28_H_24_N_3_O_2_ (M – Br)^+^ 434.1863; found 434.1859; HPLC analysis: *t*_R_ = 4.28 min, purity = 99.5%.

#### *(E*)-1-([1,1′-Biphenyl]-4-ylmethyl)-3-(3-(4-nitrophenyl)allyl)-1*H*-benzo[*d*]imidazol-3-ium Bromide (**41**) (**CKP-33**)

The title compound **42** was prepared according to the above general procedure using 1-([1,1′-biphenyl]-4-ylmethyl)-1*H*-benzo[*d*]imidazole **34** (**CKP-23**) (0.020 g, 0.071 mmol) and (*E*)-1-(3-bromoprop-1-en-1-yl)-4-nitrobenzene (0.022 g, 0.092 mmol). Pure compound was obtained as white solid, 0.014 g (36.9% yield); mp: 236.0–238.0 °C decomposition; δ 8.21 (d, *J* = 8.3 Hz, 2H), 8.04 (d, *J* = 7.9 Hz, 1H), 7.96 (d, *J* = 7.8 Hz, 1H), 7.71 (t, *J* = 7.5 Hz, 7H), 7.60 (t, *J* = 7.2 Hz, 4H), 7.44 (t, *J* = 7.6 Hz, 2H), 7.36 (t, *J* = 7.5 Hz, 1H), 7.04 (d, *J* = 15.8 Hz, 1H), 6.81 (dt, *J* = 16.0, 6.5 Hz, 1H), 5.83 (s, 2H), 5.42 (d, *J* = 6.4 Hz, 2H); ^13^C NMR (150 MHz, CD_3_OD): δ 149.0, 143.6, 143.4, 141.3, 135.3, 133.4, 133.2, 133.0, 130.1, 130.0, 129.0, 128.8, 128.6, 128.5, 128.0, 126.9, 125.0, 115.1, 114.9, 51.8, 50.2; ESI-HRMS (*m*/*z*): calcd for C_29_H_24_N_3_O_2_ (M – Br)^+^ 446.1863; found 446.1859; HPLC analysis: *t*_R_ = 4.66 min, purity = 99.8%.

#### *(E*)-1-(3,4-Dimethoxybenzyl)-3-(3-(4-nitrophenyl)allyl)-1*H*-benzo[*d*]imidazol-3-ium Bromide (**42**) (**CKP-34**)

The title compound **41** was prepared according to the above general procedure using 1-(3,4-dimethoxybenzyl)-1*H*-benzo[*d*]imidazole **36** (0.021 g, 0.076 mmol) and (*E*)-1-(3-bromoprop-1-en-1-yl)-4-nitrobenzene (0.024 g, 0.099 mmol). Pure product was obtained as off-white solid, 0.018 g (55% yield); mp: 146–148 °C (dec.); ^1^H NMR (600 MHz, CD_3_OD): δ 9.62 (s, 1H), 8.21 (d, *J* = 8.8 Hz, 2H), 7.99 (dd, *J* = 29.6 and 7.7 Hz, 2H), 7.77–7.67 (m, 4H), 7.17 (s, 1H), 7.13–6.98 (m, 3H), 6.85–6.75 (m, 1H), 5.68 (s, 2H), 5.40 (d, *J* = 6.4 Hz, 2H), 3.83 (s, 6H);^13^C NMR (150 MHz, CD_3_OD): δ 152.7, 152.4, 150.2, 144.62, 144.60, 144.2, 136.6, 136.4, 134.4, 134.3, 130.1, 130.0, 129.75, 129.71, 129.1, 128.24, 128.23, 128.1, 127.78, 127.77, 126.2, 124.1, 116.3, 116.12, 116.06, 114.8, 114.4, 57.9, 57.7, 53.3, 51.4. ESI-HRMS (*m*/*z*): calcd for C_25_H_24_N_3_O_4_ (M – Br)^+^ 430.1761; found 430.1759. HPLC analysis: *t*_R_ = 3.78 min, purity = 100%.

#### (*E*)-4-Methoxy-1-(naphthalen-2-ylmethyl)-3-(3-(4-nitrophenyl)allyl)-1*H*-benzo[*d*]imidazol-3-ium Bromide (**43**) (**CKP-42**)

The title compound **43** was prepared according to the above general procedure using 4-methoxy-1-(naphthalen-2-ylmethyl)-1*H*-benzo[*d*]imidazole **28 (CKP-25)** (0.017 g, 0.059 mmol) and (*E*)-1-(3-bromoprop-1-en-1-yl)-4-nitrobenzene (0.019 g, 0.077 mmol). Pure compound was obtained as yellowish solid, 0.026 g (84% yield); mp: 227–228 °C; ^1^H NMR (600 MHz, CD_3_OD): δ 9.63 (s, 1H), 8.21 (d, *J* = 8.7 Hz, 2H), 8.02 (s, 1H), 7.94 (d, *J* = 8.5 Hz, 1H), 7.91–7.87 (m, 2H), 7.69 (d, *J* = 8.7 Hz, 2H), 7.59–7.52 (m, 4H), 7.47 (d, *J* = 8.5 Hz, 1H), 7.22 (d, *J* = 8.2 Hz, 1H), 6.90 (d, *J* = 15.9 Hz, 1H), 6.84–6.79 (m, 1H), 5.88 (s, 2H), 5.52 (d, *J* = 6.2 Hz, 2H), 4.09 (s, 3H); ^13^C NMR (150 MHz, CD_3_OD): δ 150.1, 148.9, 143.6, 142.9, 135.0, 134.8, 134.7, 134.5, 131.8, 130.4, 129.8, 129.1, 129.06, 128.8, 128.7, 128.4, 128.1, 127.9, 126.2, 124.9, 123.0, 109.4, 106.7, 57.1, 52.5, 52.3; ESI-HRMS (*m*/*z*): calcd for C_28_H_24_O_3_N_3_ [M – Br]^+^ 450.1812, found 450.1808; HPLC analysis: *t*_R_ = 4.11 min, purity = 97.8%.

#### (*E*)-3-(3-(4-Chlorophenyl)allyl)-4-methoxy-1-(naphthalen-2-ylmethyl)-1*H*-benzo[*d*]imidazol-3-ium Bromide (**44**) (**CKP-43**)

The title compound **44** was prepared according to the above general procedure using 4-methoxy-1-(naphthalen-2-ylmethyl)-1*H*-benzo[*d*]imidazole **28** (**CKP-27**) (0.020 g, 0.069 mmol) and (*E*)-1-(3-bromoprop-1-en-1-yl)-4-chlorobenzene (0.021 g, 0.090 mmol). Pure compound was obtained as white solid, 0.013 g (36% yield); mp: 228–229 °C; ^1^H NMR (600 MHz, CDCl_3_): δ 11.73 (s, 1H), 7.97 (s, 1H), 7.85–7.77 (m, 3H), 7.54 (d, *J* = 8.5 Hz, 1H), 7.48 (dd, *J* = 6.2 and 3.2 Hz, 2H), 7.38 (d, *J* = 8.1 Hz, 3H), 7.25 (d, *J* = 8.4 Hz, 2H), 7.14 (d, *J* = 8.4 Hz, 1H), 6.99–6.92 (m, 2H), 6.55 (dt, *J* = 15.1 and 6.8 Hz, 1H), 5.96 (s, 2H), 5.55 (d, *J* = 6.8 Hz, 2H), 4.04 (s, 3H); ^13^C NMR (150 MHz, CDCl_3_): δ 148.5, 135.9, 134.2, 133.8, 133.18, 133.16, 132.9, 129.9, 129.4, 128.7, 128.3, 128.2, 128.0, 127.8, 127.7, 126.8, 126.7, 125.1, 122.0, 121.2, 107.5, 105.5, 56.3, 51.7; ESI-HRMS (*m*/*z*): calcd for C_28_H_24_ON_2_Cl [M – Br]^+^ 439.1572, found 439.1566; HPLC analysis: *t*_R_ = 5.08 min, purity = 99.4%.

#### (*E*)-1-([1,1′-Biphenyl]-4-ylmethyl)-4-methoxy-3-(3-(4-nitrophenyl)allyl)-1*H*-benzo[*d*]imidazol-3-ium Bromide (**45**) (**CKP-40**)

The title compound **45** was prepared according to the above general procedure using 1-([1,1′-biphenyl]-4-ylmethyl)-4-methoxy-1*H*-benzo[*d*]imidazole **29** (**CKP-27**) (0.020 g, 0.064 mmol) and (*E*)-1-(3-bromoprop-1-en-1-yl)-4-nitrobenzene (0.020 g, 0.083 mmol). Pure compound was obtained as off-white solid, 0.033 g (92% yield); mp: 203–204 °C; ^1^H NMR (600 MHz, CD_3_OD): δ 8.21 (d, *J* = 8.4 Hz, 2H), 7.70 (d, *J* = 8.2 Hz, 4H), 7.62–7.58 (m, 4H), 7.55 (d, *J* = 7.9 Hz, 1H), 7.50–7.41 (m, 3H), 7.39–7.34 (m, 1H), 7.24 (d, *J* = 8.2 Hz, 1H), 6.90 (d, *J* = 16.1 Hz, 1H), 6.81 (dt, *J* = 16.0 and 6.2 Hz, 1H), 5.75 (s, 2H), 5.52 (d, *J* = 6.3 Hz, 2H), 4.09 (s, 3H); ^13^C NMR (150 MHz, CD_3_OD): δ 150.0, 148.8, 143.5, 141.2, 134.8, 134.4, 133.3, 129.9, 129.7, 128.8, 128.7, 128.6, 128.2, 127.8, 124.8, 109.3, 106.5, 57.0, 52.4, 51.7; ESI-HRMS (*m*/*z*): calcd for C_30_H_26_O_3_N_3_ [M – Br]^+^ 476.1969, found 476.1966. HPLC analysis: *t*_R_ = 4.65 min, purity = 100%.

#### (*E*)-1-([1,1′-Biphenyl]-4-ylmethyl)-3-(3-(4-chlorophenyl)allyl)-4-methoxy-1*H*-benzo[*d*]imidazol-3-ium Bromide (**46**) (**CKP-41**)

The title compound **46** was prepared according to the above general procedure using 1-([1,1′-biphenyl]-4-ylmethyl)-4-methoxy-1*H*-benzo[*d*]imidazole **29** (**CKP-27**) (0.014, 0.062 mmol) and (*E*)-1-(3-bromoprop-1-en-1-yl)-4-chlorobenzene (0.015 g, 0.048 mmol). Pure product was obtained as white solid, 0.018 g (50% yield); mp: 205–206 °C; ^1^H NMR (600 MHz, CD_3_OD): δ 7.68 (d, *J* = 7.8 Hz, 2H), 7.62–7.53 (m, 6H), 7.50–7.41 (m, 5H), 7.34 (dd, *J* = 12.2 and 7.8 Hz, 3H), 7.22 (d, *J* = 8.2 Hz, 1H), 6.82 (d, *J* = 15.8 Hz, 1H), 6.59 (dt, *J* = 13.4 and 6.5 Hz, 1H), 5.75 (s, 2H), 5.46 (d, *J* = 6.5 Hz, 2H), 4.09 (s, 3H); ^13^C NMR (150 MHz, CD_3_OD): δ 150.2, 143.5, 141.4, 136.0, 135.9, 134.9, 133.5, 130.0, 129.95, 129.90, 129.8, 129.4, 128.9, 128.8, 128.0, 124.0, 109.3, 106.7, 57.1, 52.8, 51.8; ESI-HRMS (*m*/*z*): calcd for C_30_H_26_ON_2_Cl [M – Br]^+^ 465.1728, found 465.1722; HPLC analysis: *t*_R_ = 5.75 min, purity = 98.9%.

### Biological Evalution

#### Generation of Expression Clones of S Protein RBD and ACE2 Receptor (aa19–615)

A 672bp DNA fragment corresponding to the S protein RBD (aa residues 319–541) of SARS-CoV-2 and a 1,797bp DNA fragment corresponding to its interacting region of (aa residues 19–615) ACE2 receptor were PCR amplified from pDONR223 SARS-CoV-2 S (Addgene #149329) and pENTR223 ACE2 (Addgene #149719) plasmids, respectively using KAPA HiFi DNA Polymerase (KAPA biosystems). Amplified DNA fragments were purified from a 1% agarose gel and combined with the pDONR221 plasmid (1:3 ratio) in the presence of BP clonase enzyme mix (ThermoFisher). Reaction mixtures were incubated overnight at 25 °C before used for the transformation of competent Mach1 *E. coli* cells. Transformants were grown on LB agar plates supplemented with Kanamycin. Extraction of plasmid DNA was carried out using the NucleoSpin Plasmid Kit (Macherey-Nagel). The identity of generated entry clones was verified by BsrGI restriction digestion. The cDNAs encoding the S protein RBD and ACE2 receptor (aa19–615) (Addgene #167013 and #167012) were shuttled into pcDNA3.1 PA-mCitrine-GW and pcDNA3.1 c-myc-NL-GW destination vectors, respectively^125^ in the presence of LR clonase enzyme mix (ThermoFisher). Reaction mixtures were incubated overnight at 25 °C and used for the transformation of Mach1 *E. coli* cells. Transformants were grown on LB agar plates supplemented with 100 μg/mL Ampicillin. Plasmid extraction was carried out using the Nucleospin Plasmid Kit (Macherey-Nagel) and the identity of plasmids was verified by BsrGI restriction digestion.

#### Immunoblotting

HEK293T cells (2 × 10^4^) were seeded in a 96-well plate and transiently transfected with 0.2 ug plasmid DNA using Polyethylenimine (PEI) (1:3 ratio). Cells were lysed 48 h post transfection in RIPA buffer containing protease and phosphatase inhibitors (Thermofisher) and benzonase (Calbiochem-Novagen). Production of mCitrine-S protein RBD and NL-ACE2 (aa19–615) was verified by Western Blotting using an anti-Spike (69323, CST) and an anti-ACE2 antibody (4355, CST), respectively. GAPDH was used as loading control and was detected with a rabbit anti-GAPDH antibody (5174, CST). Finally, blots were incubated for 2 h with the appropriate secondary alkaline phosphatase-conjugated antibodies and protein bands were visualized with NBT/BCIP (Applichem).

#### Cytotoxicity Assay

Chemical compounds were dissolved and serially diluted in DMSO. Their cytotoxicity was assessed using an MTT assay. Briefly, HEK293T, SH-SY5Y or Vero E6 cells were cultured in 96-well plates (4 × 10^4^ cells/well) at 37 °C in a 5% CO_2_ atmosphere. Cells were treated for 48 h with varying concentrations of the compounds (100–0.1 μM) or solvent. The final concentration of DMSO in culture did not exceed 1%. Treated cells were incubated for 4 h with 0,5 μg/mL MTT (Applichem). After incubation, the MTT-containing medium was removed and formazan crystals were dissolved in DMSO. The optical density (OD) of formazan solution at 570 and 630 nm was measured in a SPARK plate reader (Tecan).

#### LuTHy Assay for Spike RBD and ACE2 Receptor (aa19–615)

The effect of chemical compounds in the interaction of Spike RBD and ACE2 receptor (aa19–615) was assessed using LuTHy assay, as previously described.^125^ In brief, HEK293T cells were transfected with pcDNA3.1 PA-mCitrine-S RBD and pcDNA3.1 c-myc-NL- ACE2 receptor (aa19–615). The next day, compounds were added in varying noncytotoxic concentrations and BRET/cBRET signals corresponding to Spike RBD/ACE2 (aa19–615) protein interaction was quantified 48 h later. The nonspecific/autofluorescence signal of each compound was calculated and subtracted from all relevant interactions. For dose-dependent LuTHy assays, HEK293T were treated with decreasing concentrations of compounds for 8 h. The % inhibitory effect of compounds was calculated compared to control cBRET signal in cells treated with the solvent.

#### SARS-CoV-2 Pseudovirus Assay

The effect of chemical compounds in SH-SY5Y cells was assessed using a pseudoviral assay (Montana Molecular). In brief, cells were seeded in a 96-well plate (2 × 10^4^ cells per well) and treated for 24 h with a transduction mixture containing red ACE2 BacMam (3.3 × 10^8^ VG/mL) and 2 mM sodium butyrate in culture medium. Chemical compounds at a noncytotoxic concentration were preincubated with a transduction mixture containing green SARS-CoV-2 pseudovirus (3.3 × 10^8^ VG/mL) and 2 mM sodium butyrate for 30 min at 37 °C under shaking, before added in cell cultures. After incubation for 24 h, medium was removed, cells were washed with PBS and green/red fluorescence were measured at 488/535 nm and 558/603 nm excitation/emission wavelengths, respectively. The background fluorescence of chemical compounds in green and red wavelengths was measured in treated cells producing only red ACE2 BacMam or uninfected cells, respectively (normalized green and red fluorescence) and was subtracted from the total fluorescence. The inhibitory effect of compounds was calculated by dividing the normalized green fluorescence signal (cells infected with the SARS-CoV-2 pseudovirus) to the normalized red fluorescence signal (viable cells producing BacMam).

#### SARS-CoV-2 Culture

SARS-CoV-2 (isolate 30–287) was obtained through culture in Vero E6 cells (ATCC CRL-1586) from a COVID-19 patient in Alexandroupolis, Greece. The virus was recovered from a nasopharyngeal swab, after rinsing with 1 mL saline and double filtering through a 0.22 nm filter. Viral stock was prepared by infecting fully confluent Vero E6 cells in DMEM, supplemented with 10% fetal bovine serum (FBS) and 1% antibiotics (penicillin-streptomycin) at 37 °C and 5% CO_2_. Four days after inoculation, the supernatant was frozen at −80 °C until use.

#### Treatment of Vero E6 Cells

Infections were carried out in 24-well plates, using SARS-CoV-2 (M.O.I. of 0.01) on Vero E6 cells. Before cell treatment, the virus was preincubated for 30 min at 37 °C and 5% CO_2_ with chemical substances (final concentration ranging from 50 to 0.01 μM) and nirmatrelvir (Paxlovid, Pfizer) (final concentration of 300 nM in DMEM). After the initial incubation, the medium containing the corresponding compound and the virus was used to treat Vero E6 cells for 48 h. As controls, cells were treated only with SARS-CoV-2 or were left untreated for 48 h. Cell morphology was observed by phase contrast in an inverted microscope in order to record cytopathic effect (CPE) after treatment. CPE values were calculated as percentile percentages of the respective values of cells treated only with SARS-CoV-2 and were used for the calculation of half maximal inhibitory concentration (IC_50_) of chemical substances. For the calculation of half maximal tissue culture infectious dose (TCID_50_), Vero E6 cells were infected with serial dilutions (1/10–1/10^8^) of supernatants collected from the infection of Vero E6 cells in the presence or absence of chemical substances, followed by incubation at 37 °C and 5% CO_2_ for 5 days. Cells incubated only with DMEM were used as control. To determine the SARS-CoV-2 viral load, RNA was extracted from supernatants (100 μL) using NucleoSpin Dx Virus kit (Macherey Nagel). Multitarget real-time PCR was performed using COVID-19 SARS-CoV-2 Real-TM kit according to the manufacturer’s instructions (Sacace Biotechnologies).

#### Statistics and Data Fitting

All assays were performed in technical triplicates. Data are presented as mean ± standard deviation and p-values were calculated in a standard *t* test. For the calculation of the half-maximal inhibitory concentration (IC_50_), inhibition data were converted to percent inhibition and fitted with standard log inhibitor vs normalized response model, using nonlinear regression in GraphPad Prism 9 software.

#### Microbial Mutagenicity Assay: Ames Test

The mutagenicity assay for **CKP-25** was conducted following the methodology outlined by Maron and Ames.^126^ All required media and the bacterial strain *E. coli* WP2 uvrA were obtained from TrinovaBiochem. The plate incorporation method was used for this assay both in the absence or presence of the liver microsomal metabolic activation (S9) mixture at a concentration of 10% v/v. Bacterial cultures were initially grown in nutrient broth under shaking conditions (150 rpm) at 37 °C for 15 h, until reaching a density of (1–2) × 10^9^ bacteria/mL (OD 650 nm approximately 1.0–1.4). For the plate incorporation procedure, 100 μL of the bacterial suspension was mixed with 100 μL of either **CKP-25** or the negative control (DMSO) in 2 mL of tryptophan-supplemented top agar, maintained at 50 °C. This mixture was then poured onto selective agar plates and incubated at 37 °C for 3 days. Five concentrations of **CKP-25** (12.5, 25, 50, 100, and 200 μg/plate) were tested. Positive control substances included 2.5 μL/plate of methylmethanesulfonate (for tests without metabolic activation) and 10 μg/plate of 2-aminoanthracene (for tests with metabolic activation). Each concentration of **CKP-25** and the controls were tested in duplicate. Genotoxicity was determined by assessing the fold increase in the number of revertant colonies. A biologically relevant increase was defined as a 2-fold increase in the mean number of revertant colonies in the *WP2 uvrA* strain, compared to the negative control.

#### Metabolic Stability

Metabolic stability was evaluated using pooled human liver microsomes at a 0.5 mg/mL concentration. The incubation conditions were optimized to ensure linear formation of metabolites, taking into account both reaction time and protein concentration. A 1 mM concentration of NADPH was used as a cofactor. Each test-compound was assessed at a concentration of 1 μM. To differentiate between low and rapid clearance rates, appropriate negative and positive controls were included. The reactions were conducted in triplicate at a temperature of 37 °C and were terminated after 1 h. The depletion of test compounds was then profiled, and the residual percentage compared to time zero was determined. The readouts were recorded using a Lionheart FX system from Agilent BioTek (Winooski, VT, USA).

#### Isozyme-Specific CYP450-Metabolism

Human cytochrome P450 (CYP450) isoenzymes were expressed in Baculosomes obtained from Thermo Fisher Scientific (Waltham, MA, USA). Reagents were handled and prepared following the manufacturer’s guidelines. Compound **CKP-25** was evaluated at a concentration of 1 μM according to our established standard operating procedures and protocols as described previously.^127,128^ The enzymatic activity of various CYP450 isoforms (CYP1A2, CYP2A6, CYP2B6, CYP2C9, CYP2C19, CYP2D6, and CYP3A4) was measured, considering the kinetic model for each isoform. The reactions were conducted in triplicate at 20 °C. NADP+ (10 mM in 100 mM potassium phosphate, pH 8.0) was converted to NADPH using a regeneration system containing glucose-6-phosphate (333 mM) and glucose-6-phosphate dehydrogenase (30 U/mL in 100 mM potassium phosphate, pH 8.0). Upon the addition of the fluorescent substrate, signal monitoring was performed immediately (within 2 min) using appropriate excitation and emission wavelengths, recorded by an Agilent BioTek Lionheart FX (Winooski, VT, USA). CYP450 inhibition (%) was calculated based on the reaction rates (fluorescence intensity changes per unit time). In total, n = 60 meaurements per minute were recorded over a 60 min period.
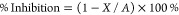
where *X*, the rate observed in the presence of a test-compound; *A*, the rate observed in the presence of negative (solvent, DMSO) control.

## Data Availability

The source code and the trained model utilized to run the virtual screening can be found in https://github.com/stemylonas/ResNetVS. The model was trained on the DUD-E data set (https://dude.docking.org/). The following third-party software was used. The structures were drawn using ChemDraw 20.0 (PerkinElmer, https://perkinelmerinformatics.com). NMR spectra were. analyzed with MestReNova v14.1.0 (Mestrelab Research; https://mestrelab.com). HPLC chromatograms were analyzed with ChromQuest 5.0. (Thermo Fisher Scientific; https://tools.thermofisher.com). HRMS spectra were analyzed with XCALIBURTM 2.1W/FOUNDATION 1.0.1 (Thermo Fisher Scientific; https://tools.thermofisher.com). Compound filtering: OpenBabel (https://sourceforge.net/projects/openbabel/), PyRx (https://sourceforge.net/projects/pyrx/), PyMol (https://pymol.org/).

## Supporting Information Available

Supplementary Tables S1 and S2, Supplementary Figures S1–S5, ^1^H NMR, ^13^C NMR and HR-MS spectra, HPLC traces and Supplementary Charts S1-S4 of the final compounds (PDF)
Molecular formula strings (CSV)

## Abbreviations

ACE2: angiotensin-converting enzyme 2
ADME-Tox: absorption, distribution, metabolism, excretion and toxicity
CCL2: CC chemokine ligand 2
CCR2: CC chemokine receptor 2
CCR5: CC chemokine receptor 5
CNN: convolutional neural network
CPE: cytopathic effect
DPPA: diphenyl phosphorylazide
FCC: Flash column chromatography
Mpro: main protease
PAINS: pan-assay interfering compounds
PK: pharmacokinetic
PPID: protein–protein interaction drug
RBD: receptor binding domain
RBM: receptor binding motif
RdRp: RNA-dependent RNA polymerase
PTSA: p-Toluenesulfonic acid
qPCR: quantitative real-time polymerase chain reaction
SARS-CoV-2: severe-acute-respiratory syndrome coronavirus 2
SBDD: structure-based drug design
TCID50: tissue culture infectious dose
TLC: thin layer chromatography
TMPRSS2: transmembrane serine protease 2
tPSA: topological polar surface area
VS: virtual-screening
WHO: World Health Organization
