# Supplementary material for: AI Promoted Virtual Screening, Structure-Based Hit Optimization, and Synthesis of Novel COVID-19 S-RBD Domain Inhibitors
Source: J Chem Inf Model. 2024 Nov 13;64(22):8562–85. doi: 10.1021/acs.jcim.4c01110 (PMC11600510; doi:10.1021/acs.jcim.4c01110)
Supplement: Supplementary file 1 — ci4c01110_si_001.pdf [file ci4c01110_si_001.pdf]

Supporting Information

AI promoted virtual screening, structure-based hit optimization and synthesis of novel COVID-19 S-RBD domain inhibitors

Ioannis Gkekas[1, #,] Sotirios Katsamakas[2, 3, 4, #], Stelios Mylonas[3, #], Theano Fotopoulou[2], George E. Magoulas[2], Alia Cristina Tenchiu[2], Marios Dimitriou[5], Apostolos Axenopoulos[3], Simona Kostova[6], Erich E. Wanker[6], Demetris Papahatjis[2], Vassilis G Gorgoulis[7], Maria Koufaki[2*], Ioannis Karakasiliotis[5], Theodora Calogeropoulou[2*], Petros Daras[3] and Spyros Petrakis [1*].

[1] Institute of Applied Biosciences, Centre for Research and Technology Hellas, Thessaloniki, Greece

[2] Institute of Chemical Biology, National Hellenic Research Foundation, Leof. Vassileos Constantinou 48, Athens 11635, Greece

[3] Information Technologies Institute, Centre for Research and Technology Hellas, Thessaloniki, Greece

[4] Laboratory of Pharmaceutical Chemistry, School of Pharmacy, Faculty of Health Sciences, Aristotle University of Thessaloniki, Campus, Thessaloniki 54124, Greece

[5]Laboratory of Biology, Department of Medicine, Democritus University of Thrace, Alexandroupolis, Greece

[6] Max-Delbrueck-Center for Molecular Medicine in the Helmholtz Association, Berlin, Germany

[7] Molecular Carcinogenesis Group, Department of Histology and Embryology, Medical School, National and Kapodistrian University of Athens, Athens

[#] equal contribution

Table of contents

Supplemetary Tables S3

Supplementray Figures S5

Experimental procedures S8

Copies of ^{1}H-NMR, ^{13}C-NMR and HRMS spectra of final compounds. S13

HPLC chromatograms of final compounds S67

Supplementary Charts S80

References S82

Supplementary Tables

Table S1. Spike (RBD)/ACE2(19-615) inhibitors identified through AI-enabled VS.

Name	ChemBridge ID	IUPAC NAME	Binding scores
#1	5609384	4-(1-naphthyl)-3,4-dihydrobenzo[h]quinolin-2(1H)-one	0.972
#2	5979742	4-(2-naphthyl)-3,4-dihydrobenzo[h]quinolin-2(1H)-one	0.897
#3	6238693	3'-[(9H-fluoren-2-ylcarbonyl)amino]-4-biphenylyl acetate	0.902
#4	5635769	2-[2-oxo-2-(10H-phenothiazin-10-yl)ethyl]-1H-isoindole-1,3(2H)-dione	0.875
#5	6954709	5-(4-biphenylyl)-4-phenyl-1,3-thiazol-2-amine	0.874
#6	7226620	2-(4-biphenylylsulfonyl)-1,2,3,4-tetrahydroisoquinoline	0.857
#7	6384272	1-[(4-methoxy-1-naphthyl)sulfonyl]-1H-1,2,3-benzotriazole	0.864
#8	6431096	1-benzofuran-2-yl(4-biphenylyl)methanone	0.894
#9	6996505	2-[4-(2,2-dimethyl-4-oxo-1,2,3,4,5,6-hexahydrobenzo[a]phenanthridin-5-yl)phenoxy]acetamide	0.881
#10	5260662	1-(4-biphenylylcarbonyl)-1H-benzimidazole	0.916
#11	5668688	2,2,4-trimethyl-1,2-dihydro-6-quinolinyl (1,3-dioxo-1H-benzo[de]isoquinolin-2(3H)-yl)acetate	0.868
#12	5837562	5-(1,3-benzodioxol-5-yl)-1-benzoyl-3-phenyl-4,5-dihydro-1H-pyrazole	0.9
#13	7262001	N-[2-(4-morpholinyl)phenyl]-2-naphthamide	0.857
#14	5315079	1-[2-(9H-fluoren-2-yl)-2-oxoethyl]-2,3-dimethylpyridinium bromide	0.891
#15	6085984	N-2-naphthyl-2-(2-naphthyloxy)acetamide	0.871
#16	6541298	1,1'-(1,4-phenylene)bis[3-(1,3-benzodioxol-5-yl)-2-propen-1-one]	0.877
#17	5465521	(1,3-benzodioxol-5-ylmethyl)(4-methoxy-3-biphenylyl)amine	0.857
#18	5733393	1,3-dioxo-2-[4-(5-phenyl-1,3,4-oxadiazol-2-yl)phenyl]-5-isoindolinecarboxylic acid	0.884
#19	7306847	N-(4-{[(4,5-diphenyl-1,3-oxazol-2-yl)amino]sulfonyl}phenyl)acetamide	0.91
#20	5524376	2-[(2-naphthylamino)(4-pyridinyl)methyl]cycloheptanone	0.895
#21	7113299	5-[(8-quinolinyloxy)acetyl]-10,11-dihydro-5H-dibenzo[b,f]azepine	0.866
#22	5755483	2'-({[4-(3-oxo-3-phenyl-1-propen-1-yl)phenyl]amino}carbonyl)-2-biphenylcarboxylic acid	0.865
#23	5979349	1-(2-naphthylmethyl)-1H-benzimidazole	0.871
#24	6038144	[4-(benzyloxy)phenyl](1-methyl-1H-benzimidazol-2-yl)methanol	0.885

#25	6104275	1,3-dimethyl-5-phenyl-5,11-dihydro-1H-indeno[2',1':5,6]pyrido[2,3-d]pyrimidine-2,4,6(3H)-trione	0.896
#26	7154899	N-(4-methoxy-1-naphthyl)-4-biphenylcarboxamide	0.85
#27	5160674	2-dibenzo[b,d]furan-3-yl-1,3-dioxo-5-isoindolinecarboxylic acid	0.862
#28	6048589	4-{4-[(diphenylacetyl)amino]benzoyl}phthalic acid	0.905
#29	6659989	3-(4-biphenylyloxy)-7-hydroxy-4H-chromen-4-one	0.889
#30	6052953	2-({[4-(2-naphthyloxy)phenyl]amino}carbonyl)benzoic acid	0.924
#31	6573636	N-(4-anilinophenyl)-2-naphthamide	0.884

Table S2. RMSD values of the aligned protein structures of various SARS-CoV-2 strains.

**PDB entries**	**6m0j Å**
6m0j	0
7ekf	0.248
7ekg	0.210
7ekc	0.229
7wbq	0.298
7tez	0.521
7u0n	0.518
7xo9	0.492
7xb1	0.475
7zxu	0.377

Supplementary Figures

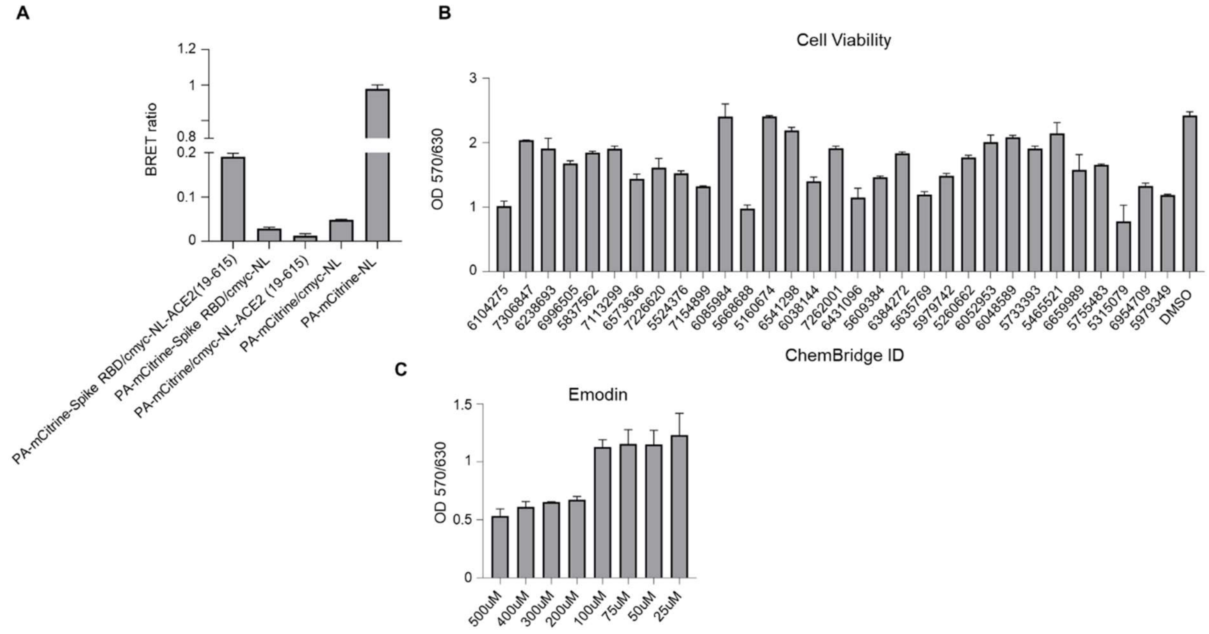

Figure S1. Quantification of Spike-RBD/ACE2 (19-615) interaction, cytotoxicity of computationally-predicted compounds in HEK293T cells **A)** Establishment of cell-based LuTHy assay for the quantification of Spike-RBD/ACE2 (19-615) interaction. The positive control PA-mCitrine-NL and the interacting proteins PA-mCitrine-Spike-RBD and NL-ACE2 (aa 19-615) show high corrected BRET (cBRET) ratios. Data are presented as mean ± SD. **B-C)** Viability of HEK293T cells treated with **(B)** hit compounds in a concentration of 100 μM or **(C)** the commercial inhibitor Emodin. Cell viability was measured 48 h later using an MTT assay Cells treated with DMSO were used as control. **C)** dvc sdcvsd

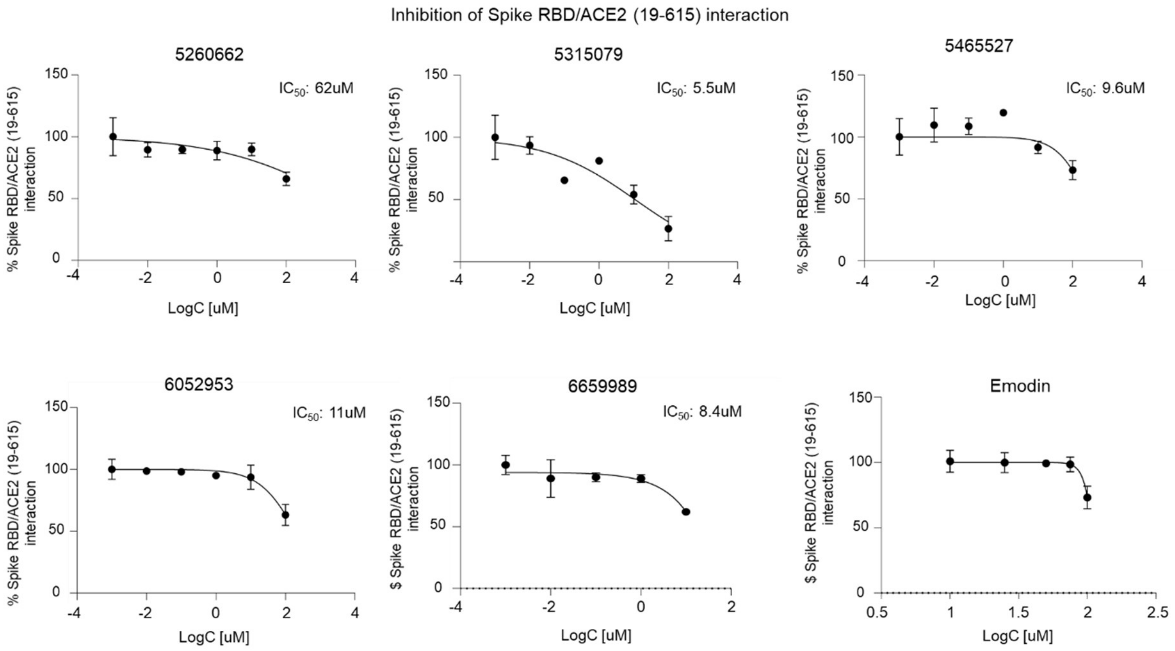

Figure S2. Concentration-dependent inhibition of Spike RBD/ACE2 (19-615) interaction by the active compounds and the positive control (Emodin), as determined by LuTHy assay. Data were presented as mean ± SD and fitted with standard sigmoid curves for IC_{50} determination.

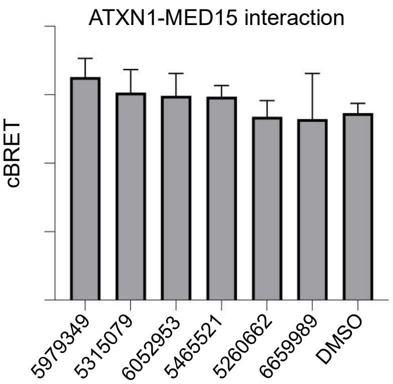

Figure S3. Effect of compounds in ATXN1/MED15 interaction. Validation of six compounds' specificity as inhibitors to Spike RBD/ACE2 interaction. Compounds were checked for their inhibitory activity against ATXN1/MED15 interaction in LuTHy assay. cBRET ratios as compared to control DMSO are shown in diagram, confirming their interaction-based specificity.

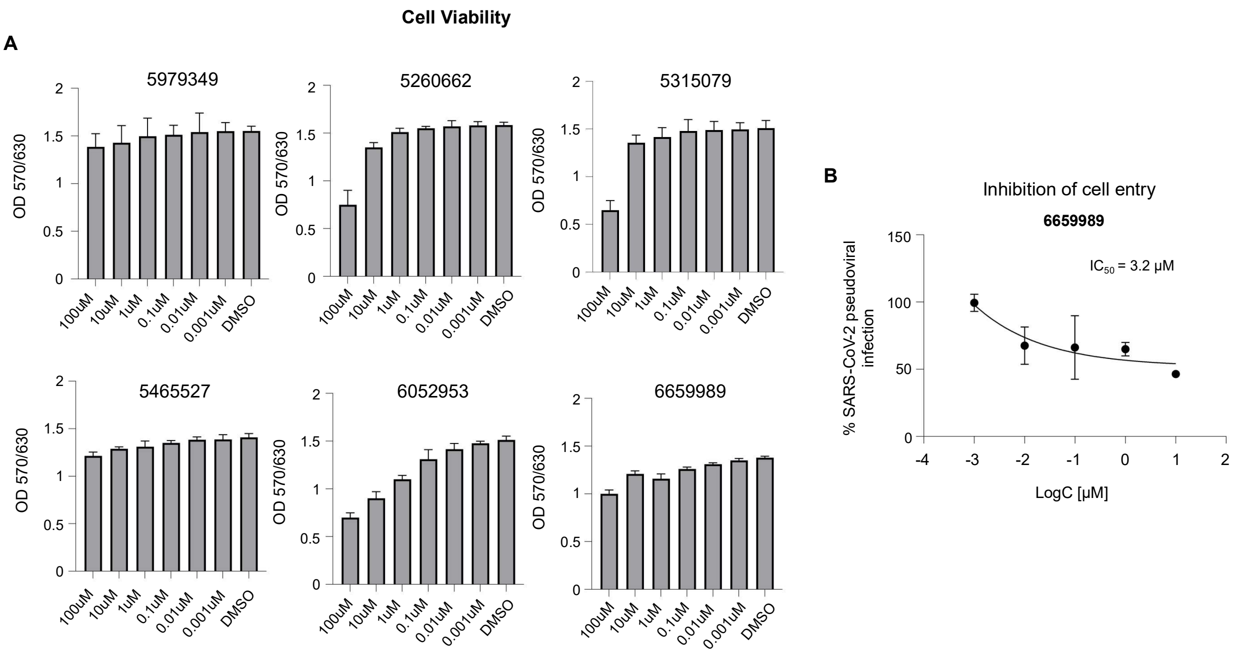

Figure S4. A) Cytotoxicity of the active six compounds in SH-SY5Y cells. Cells were incubated with gradient concentrations of compounds for 48 h. Cell viability was measured by MTT assay**. B)** Concentration-response curve obtained for the inhibition of SARS-CoV-2 pseudovirus entry in a cell-based assay. Compound 6659989 blocks the entry of SARS-COV-2 pseudovirus with an IC_{50} of 3.2 µM. Data are mean ± SD and fitted with standard sigmoid curves for IC_{50} determination.

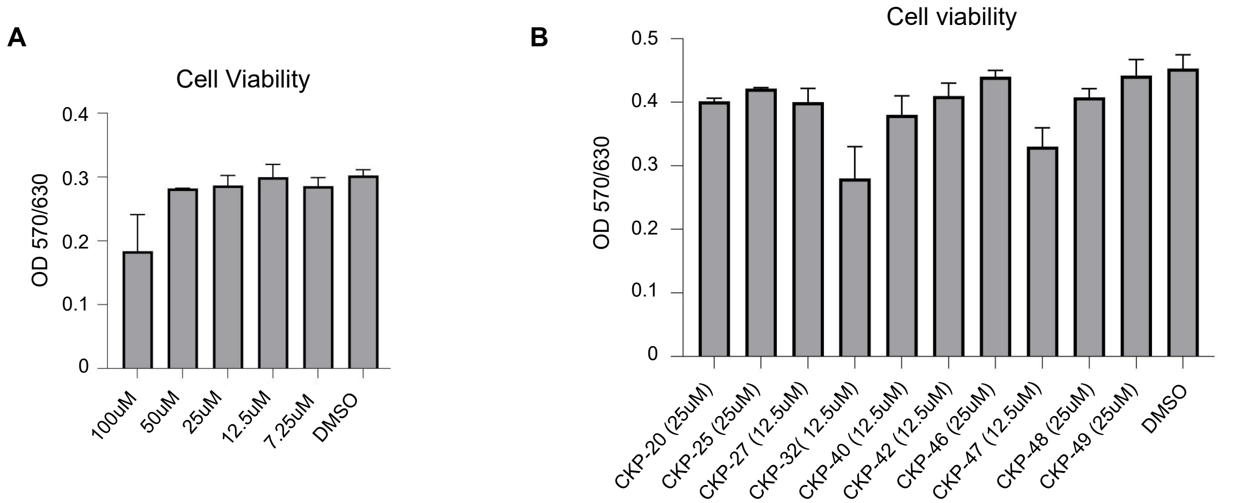

Figure S5. Toxicity assessment of **A) CKP-22** (7.25-100 µM final concentration) and **B)** its derivatives in Vero E6 cells. Cell viability was monitored at 48hrs post treatment using MTT assay.

Experimental procedures

5-nitro-1*H*-benzo[*d*]imidazole (2) [1]

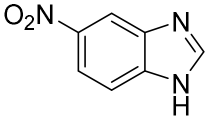

To an ice-cold mixture of benzimidazole (1.0 g, 8.46 mmol) in conc. H_2SO_4 (6 mL), fuming HNO_3 (6 mL) was added dropwise and the resulting solution was stirred at 0 °C for 3h. Then, reaction mixture was poured slowly to ice-water with stirring. The precipitated product was filtered, washed with cold water and dried. White solid, 1.38 g (quant.); R_f = 0.29 (DCM/MeOH 9:1 v/v); ^{1}H-NMR (300 MHz, DMSO-d_6): δ 12.35 (s, 1H), 9.39 (unresolved d, 1H), 8.65 (s, 1H), 8.32 (dd, J = 9.0 and 2.4 Hz, 1H), 7.97 (d, J = 9.0 Hz, 1H); ^{13}C-NMR (75 MHz, DMSO-d_6): δ 145.7, 144.6, 136.5, 132.6, 120.3, 115.5, 111.8.

Synthesis of bromides S7 and S8

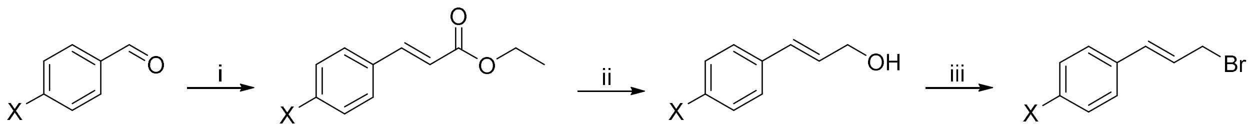

S1: X = Cl
S2: X= NO_2

S3: X = Cl
S4: X= NO_2

S5: X = Cl
S6: X= NO_2

S7: X = Cl
S8: X= NO_2

Scheme S1. Synthesis of bromides **S7** and **S8**. *Reagents and conditions*. (i) triethyl phosphonoacetate, LiCl, DBU, MeCN, rt, overnight; (ii) DIBAL-H (1.2 M in PhMe), PhMe, -78 °C, 0.5-1 h; (iii) PBr_3, Et_2O, 0 °C, 0.5 h.

General procedure for the Horner-Emmons reaction [2].

To an ice-cold suspension of LiCl (0.47 g, 11 mmol) in MeCN (30 mL), triethyl phosphonoacetate (1.98 mL, 10 mmol) and the appropriate benzaldehyde **S1** or **S2** (10 mmol) were added. After 5 min, DBU (1.49 mL, 10 mmol) was added dropwise and the resulting mixture was allowed to attend slowly rt and stirred overnight. Upon completion, solvent was evaporated under reduced pressure and the residue was partitioned between diethyl ether and brine. The organic layer was washed with brine, dried over Na_2SO_4, filtered and evaporated. The residue was subjected to FCC to afford the desired ethyl ester **S3** or **S4** respectively, in pure form.

Ethyl (*E*)-3-(4-chlorophenyl)acrylate (S3). [3]

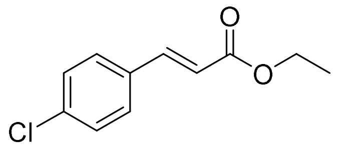

Prepared according to the above general procedure using 4-chlorobenzaldehyde (**S1**) (1.41 g, 10 mmol) as starting material. 2.04 g (97 %), colorless oil; R_f = 0.29 (Hexane/EtOAc 20:1); ^{1}H NMR (300 MHz, $CDCl_3$): δ 7.60 (d, J = 16.1 Hz, 1H), 7.44 (d, J = 9.0 Hz, 2H), 7.34 (d, J = 9.0 Hz, 2H), 6.39 (d, J = 16.1 Hz, 1H), 4.25 (q, J = 6.6 Hz, 1H), 1.32 (t, J = 6.6 Hz, 1H) ppm.

Ethyl (*E*)-3-(4-nitrophenyl)acrylate (S4). [3]

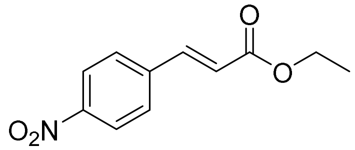

Prepared according to the above general procedure using 4-nitrobenzaldehyde (**S2**) (1.51 g, 10 mmol) as starting material. 1.94 g (88%), yellow solid; mp. 136–138 °C; R_f = 0.25 (Hexane/EtOAc 5:1); ^{1}H NMR (300 MHz, $CDCl_3$): δ 8.25 (d, J = 9.0 Hz, 2H), 7.71 (d, J = 16.1 Hz, 1H), 7.67 (d, J = 9.0 Hz, 2H), 6.56 (d, J = 16.1 Hz, 1H), 4.29 (q, J = 7.1 Hz, 1H), 1.35 (t, J = 7.1 Hz, 1H) ppm.

General procedure for the synthesis of cinnamyl alcohols. **[2]**

To a solution of the appropriate *α,β*-unsaturated ester **S3** or **S4** (8 mmol) in PhMe (65 mL) at -78 °C, DIBAL-H (13.5 mL, 16 mmol, 1.2 M in PhMe) was added dropwise. When starting material was fully consumed (0.5 – 1h), the reaction was quenched with MeOH (3 mL) and a sat. aqueous NH_4Cl solution (15 mL), it was allowed to warm slowly at rt and it was stirred for additional 20 min. The resulting salts were passed through a Celite pad and the filtrate was transferred to a separatory funnel. The aqueous phase was extracted thrice with DCM and the combined organic layers washed with brine, dried over Na_2SO_4, filtered and evaporated. The desired allylic alcohols **S5** or **S6** respectively, were obtained in pure form after FCC purification.

(*E*)-3-(4-chlorophenyl)prop-2-en-1-ol (S5) [4]

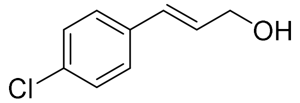

Prepared according to the above general procedure using ethyl ester **S3** (1.69 g, 8 mmol) as starting material. 1.35 g (quant.), yellow solid; mp. 62–65 °C; R_f = 0.20 (petroleum ether/Ethyl acetate 4:1); ^{1}H NMR (300 MHz, $CDCl_3$): δ 7.31–7.26 (m, 4H), 6.58 (d, J = 15.9 Hz, 1H), 6.38–6.31 (m, 1H), 4.33 (dd, J = 5.6 and 1.5 Hz, 2H); ^{13}C NMR (75 MHz, $CDCl_3$): δ 135.3, 133.4, 129.9, 129.3; 128.9 (two C), 127.8 (two C), 63.7 ppm

(*E*)-3-(4-nitrophenyl)prop-2-en-1-ol (S6) [5]

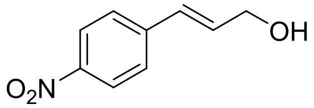

Prepared according to the above general procedure using ethyl ester **S4** (1.77 g, 8 mmol) as starting material. 1.12 g (78%), yellow solid; mp. 123–125 °C; R_f = 0.14 (petroleum ether/Ethyl acetate 3:1); ^{1}H NMR (300 MHz, $CDCl_3$): δ 8.18 (d, J = 8.6 Hz, 2H), 7.52 (d, J = 8.6 Hz, 2H), 6.76 (d, J = 16.1 Hz, 1H), 6.54 (dt, J = 16.1 and 5.0 Hz, 1H), 4.40 (d, J = 5.0 Hz, 2H); ^{13}C NMR (75 MHz, $CDCl_3$): δ 146.4, 143.6, 133.7; 128.4; 127.1 (two C), 124.2 (two C), 63.3 ppm.

General procedure for the synthesis of cinnamyl bromides. [6]

To an ice-cold solution of cinnamyl alcohol **S5** or **S6** (5 mmol) in diethyl ether (12.5 mL), PBr_3 (0.62 mL, 6.5 mmol) was added. The reaction mixture was stirred at 0 °C until the full consumption of starting material (20-30 min), it was diluted with diethyl ether (12.5 mL) and it was carefully quenched with a sat. aqueous $NaHCO_3$ solution (2-3 mL). The mixture was allowed to attain rt, stirred for additional 15 min and transferred to a separatory funnel. The layers were separated and the aqueous phase was extracted thrice with diethyl ether. The combined organic layers were washed with a sat. aqueous $Na_2S_2O_3$ solution and brine, dried over Na_2SO_4, filtered and evaporated to afford cinnamyl bromides **S7** and **S8** respectively.

(*E*)-1-(3-bromoprop-1-en-1-yl)-4-chlorobenzene (S7) [6]

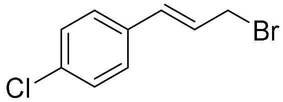

Prepared according to the above general procedure using cinnamyl alcohol **S5** (0.84 g, 5 mmol) as starting material. 0.83 g (72%), white solid; mp. 60–61 °C; ^{1}H NMR (600 MHz, $CDCl_3$): δ 7.35–7.28 (m, 4H), 6.60 (d, J = 15.6 Hz, 1H), 6.41–6.32 (m, 1H), 4.14 (d, J = 7.8 Hz, 1H); ^{13}C NMR (150 MHz, $CDCl_3$): δ 134.3, 134.0, 133.2, 128.9 (two C), 127.9 (two C), 125.8, 33.0 ppm.

(*E*)-1-(3-bromoprop-1-en-1-yl)-4-nitrobenzene (S8). [6]

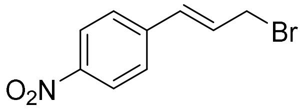

Prepared according to the above general procedure using cinnamyl alcohol **S6** (0.90 g, 5 mmol) as starting material. 0.64 g (53%), yellow solid (recrystallized from Et_2O/Hex 95:5); m.p. 76–78 °C; ^{1}H NMR (600 MHz, $CDCl_3$): δ 8.19 (d, J = 8.4 Hz, 2H), 7.52 (d, J = 8.4 Hz, 2H), 6.70 (d, J = 15.6 Hz, 1H), 6.58 – 6.56 (m, 1H), 4.16 (dd, J = 7.8 and 0.8 Hz, 1H); ^{13}C NMR (150 MHz, $CDCl_3$): δ 147.6, 142.2, 132.1, 129.9 (two C), 127.3, 124.0 (two C), 31.8 ppm.

Synthesis of bromide S12.

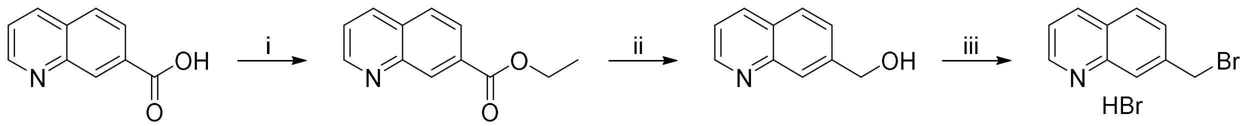

S9 S10 S11 S12

Scheme S2. Synthesis of bromide **S12**. *Reagents and conditions*. (i) conc. H_2SO_4, EtOH, reflux, 72 h; (ii) DIBAL-H (1.0 M in DCM), THF, -78 to 0 °C, 1.5 h; (iii) 33% HBr solution in CH_3CO_2H, reflux, overnight.

Ethyl quinoline-7-carboxylate (S10)

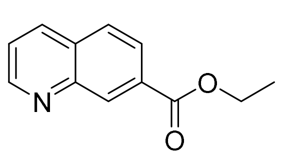

To a solution of quinoline-7-carboxylic acid (**S9**) (0.5 g, 2.88 mmol) in EtOH (20 mL), conc. H_2SO_4 (1.1 mL) was added and the resulting mixture was refluxed for 72 h. The mixture was left to attain rt and all the volatile components were evaporated under vacuo. The residue was taken up in EtOAc and washed with a sat. aqueous $NaHCO_3$ solution and brine. The organic layer was dried over Na_2SO_4, filtered and evaporated to furnish **S10** which was used to the next step without further purification.
0.6 g (quant.); off-white solid, mp. 55–56 °C; ^{1}H NMR (300 MHz, acetone-d_6): δ 9.05 (unresolved dd, 1H), 8.74 m, 1H), 8.50 (m, 1H), 8.15 (m, 2H), 7.69 (m, 1H), 4.45 (q, J = 7.0 Hz 2H), 1.44 (t, J = 7.0 Hz, 3H); ESI-MS: 424.63 (2M+Na), 202.25 (M+H).

Quinolin-7-ylmethanol (S11)

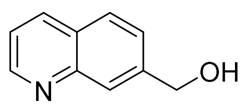

A solution of ester **S10** (0.5 g, 2.5 mmol) in THF (12.5 mL) was cooled to –78 °C and then a solution of DIBAL-H (1M in DCM, 7.5 mL) in THF (12.5 mL) was added dropwise. Upon addition, the reaction mixture was stirred at 0 °C for 1.5 h, quenched with MeOH (12.5 mL) and CH_3COOH (2.1 mL), stirred for 5 min and then a sat. aqueous sodium potassium tartrate solution (25 mL) was added. The resulting mixture was stirred for further 20 min and the EtOAc was added. The aqueous phase was extracted with EtOAc twice, and the combined organic layers were washed with brine, dried over Na_2SO_4, filtered and concentrated in vacuo. The residue was subjected to FCC (petroleum ether/EtOAc 1:1 to EtOAc) to afford alcohol **S11** in pure form which was used directly to the next step. 0.16 g (40% or 84% brsm); white solid, m.p 58–60 °C

7-(bromomethyl)quinoline hydrobromide salt (S12)

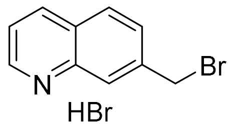

To a rb-flask containing alcohol **S11** (0.16 g, 1.0 mmol) a 33% HBr solution in CH_3CO_2H (1.2 mL) was added and the mixture was heated at 70 °C overnight. The reaction mixture was evaporated to dryness and co-evaporated three times with PhMe to remove excess CH_3CO_2H until an off-white solid was formed.

0.28 g (92%), off-white solid, m.p. 67–70 °C; ^{1}H NMR (300 MHz, DMSO-d_6): δ 9.22 (dd, J = 5.0 and 1.6 Hz, 1H), 8.95 (d, J = 8.4 Hz, 1H), 8.27 (d, J = 8.4 Hz, 1H), 8.25 – 8.23 (m, 1H), 7.95 (dd, J = 8.4 and 5.0 Hz, 1H), 7.90 (dd, J = 8.4 and 1.6 Hz, 1H), 5.02 (s, 2H).

Copies of ^{1}H-NMR, ^{13}C-NMR and HRMS spectra of final compounds.

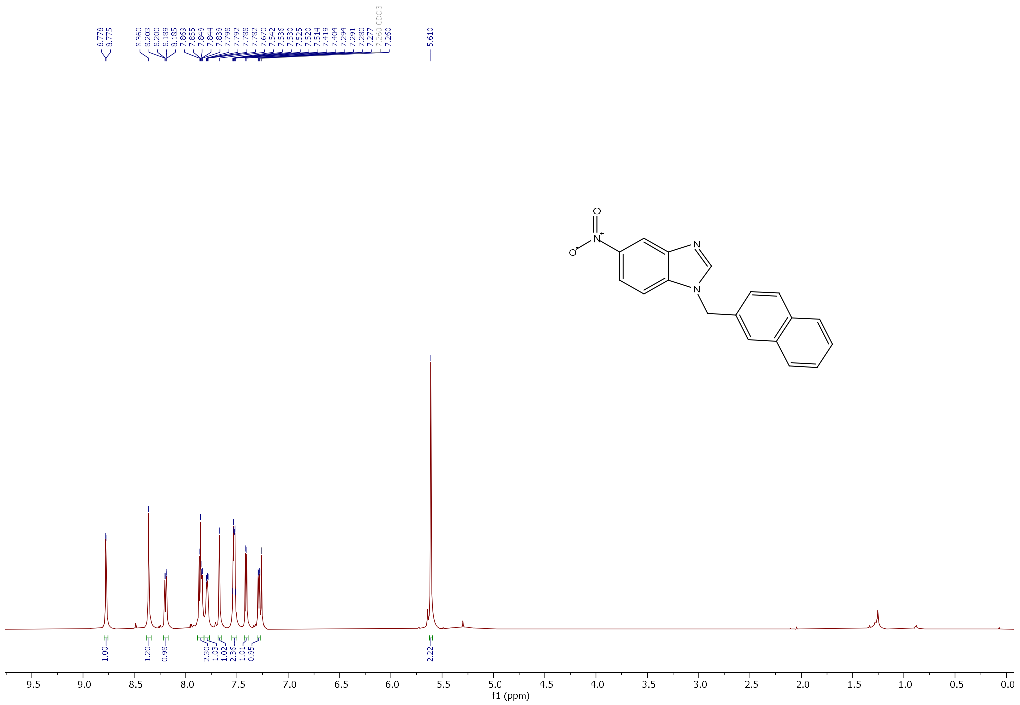

Figure S6. ^{1}H-NMR of compound **3**.

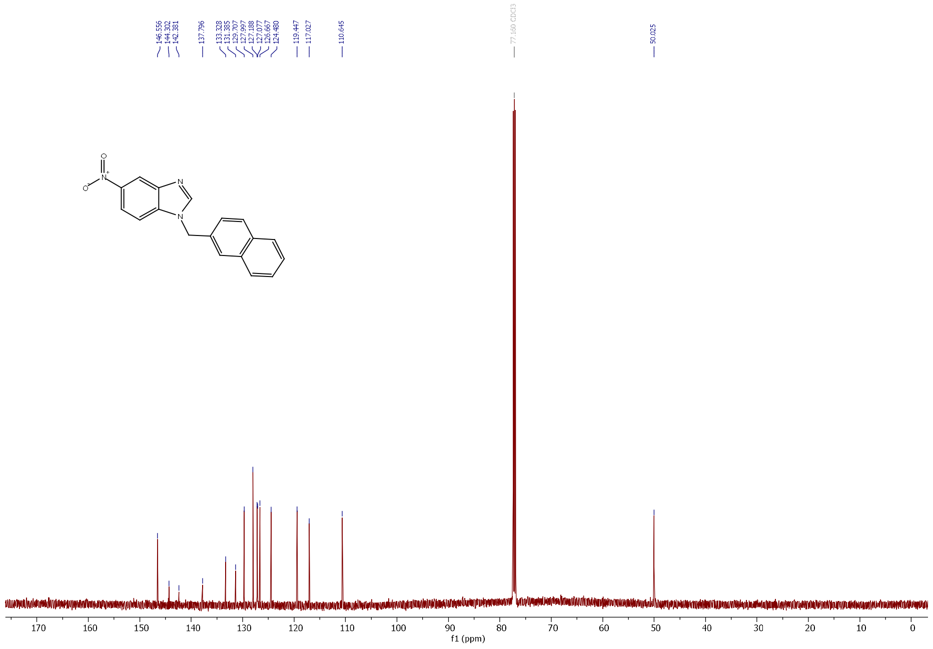

Figure S7. ^{13}C-NMR of compound **3**.

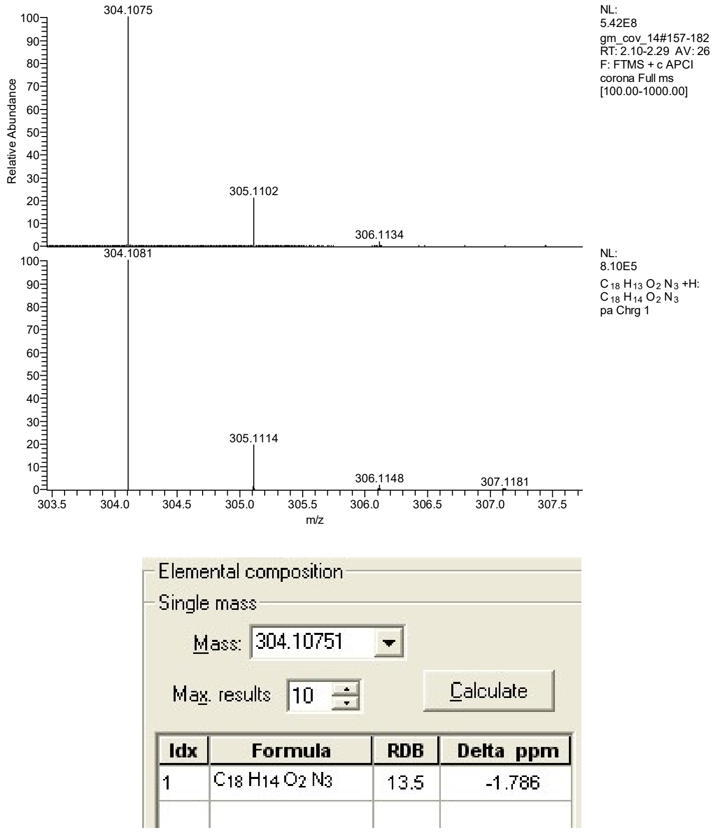

Figure S8. HRMS of compound **3**.

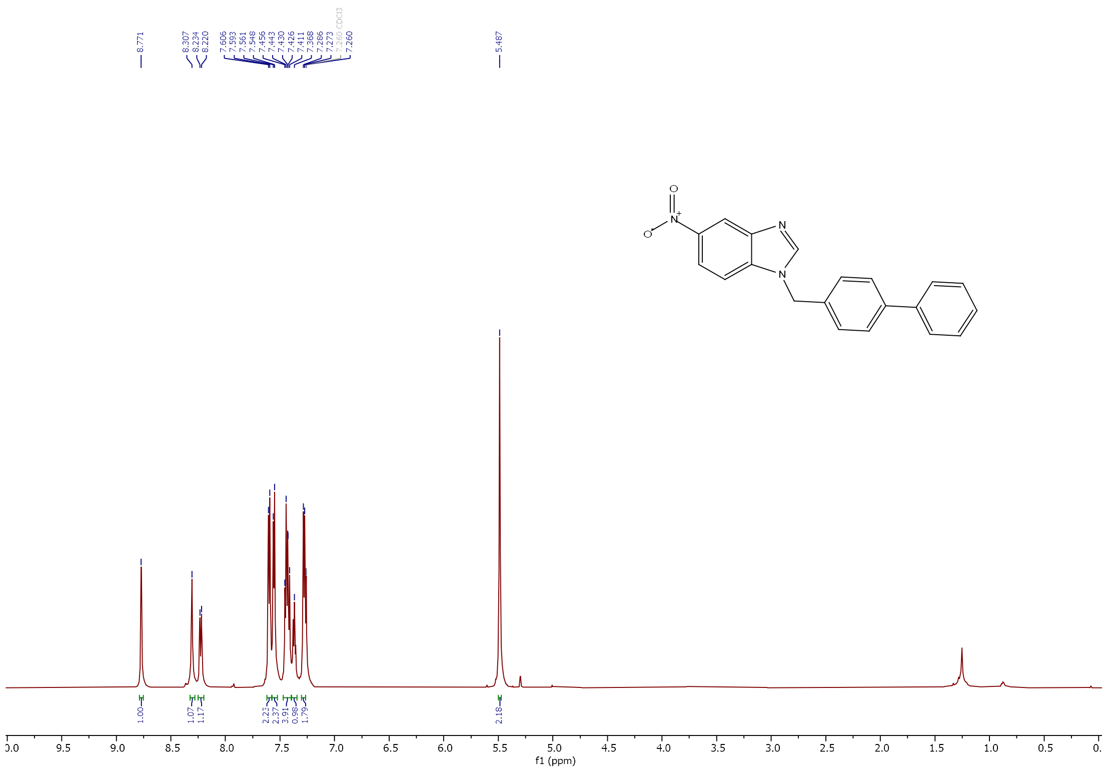

Figure S9. ^{1}H-NMR of compound **4**.

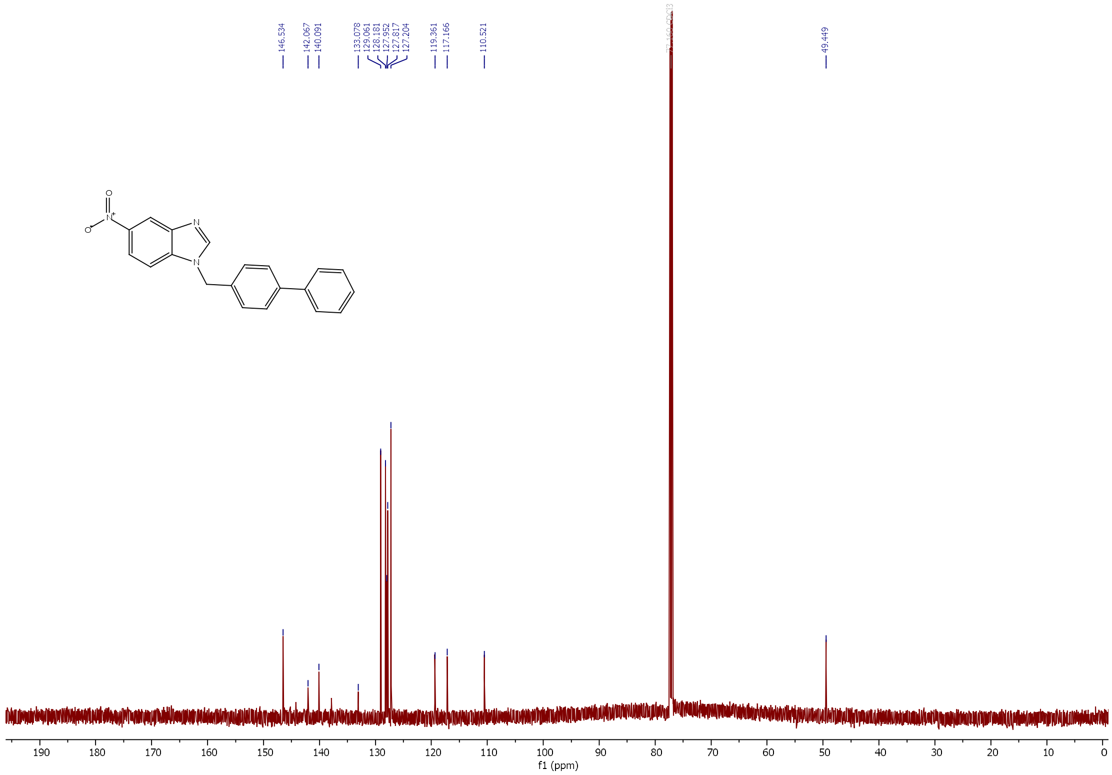

Figure S10. ^{13}C-NMR of compound **4**.

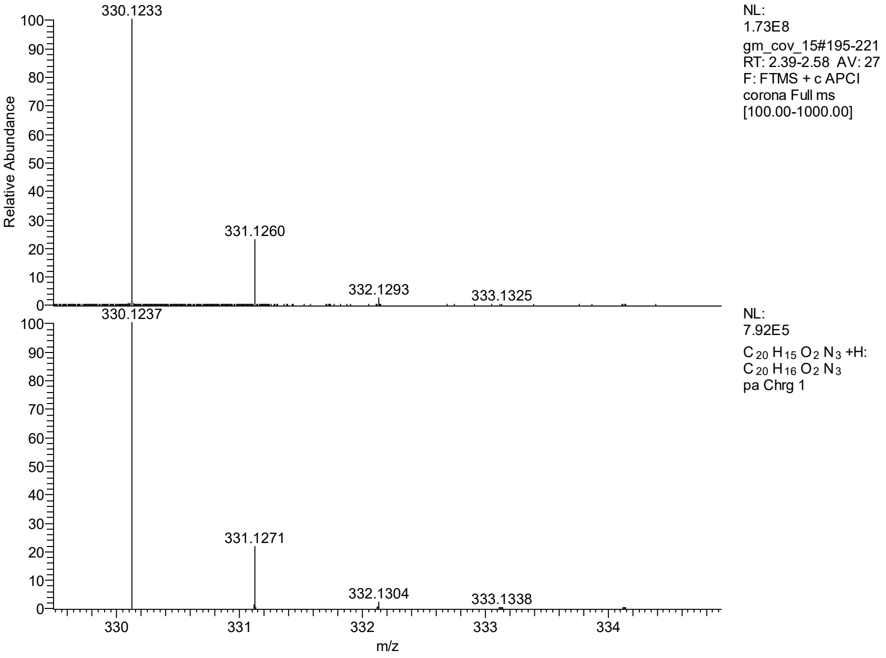

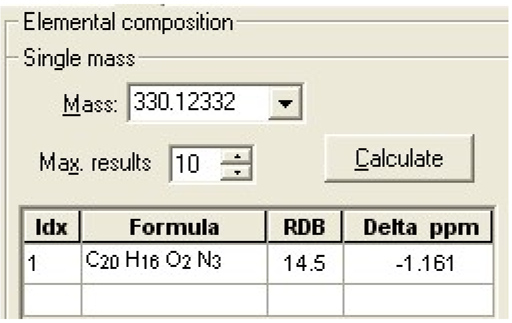

Idx	Formula	RDB	Delta ppm
1	$C_{20}H_{16}O_2N_3$	14.5	-1.161

Figure S11. HRMS of compound **4**.

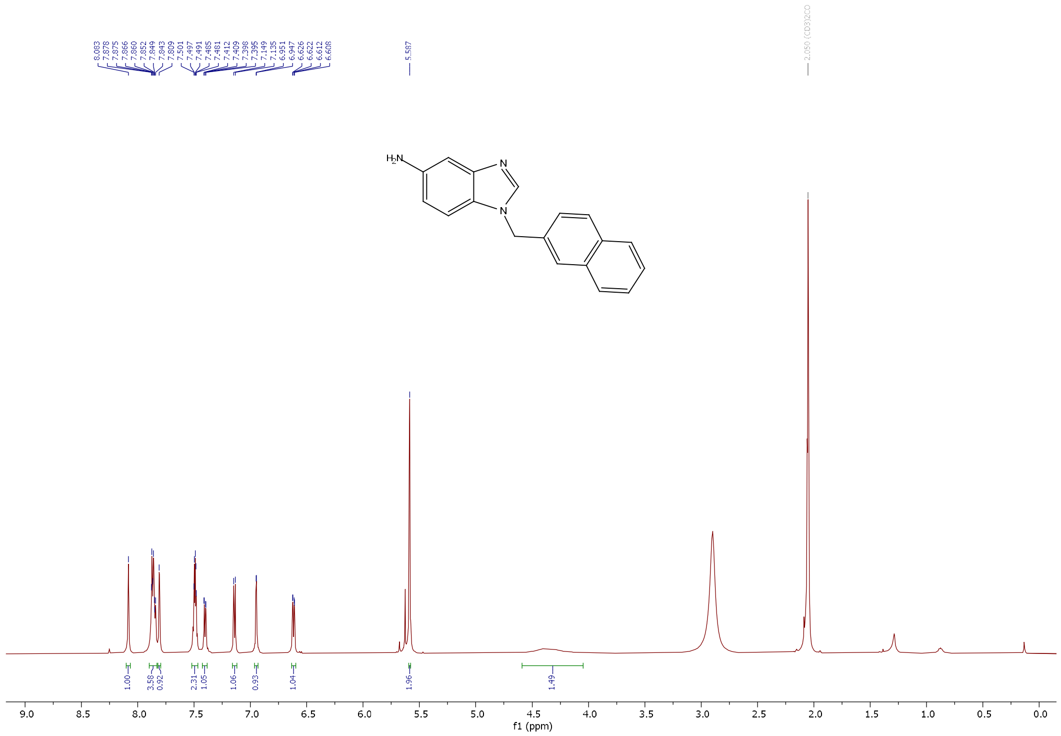

Figure S12. ^{1}H-NMR of compound **5**.

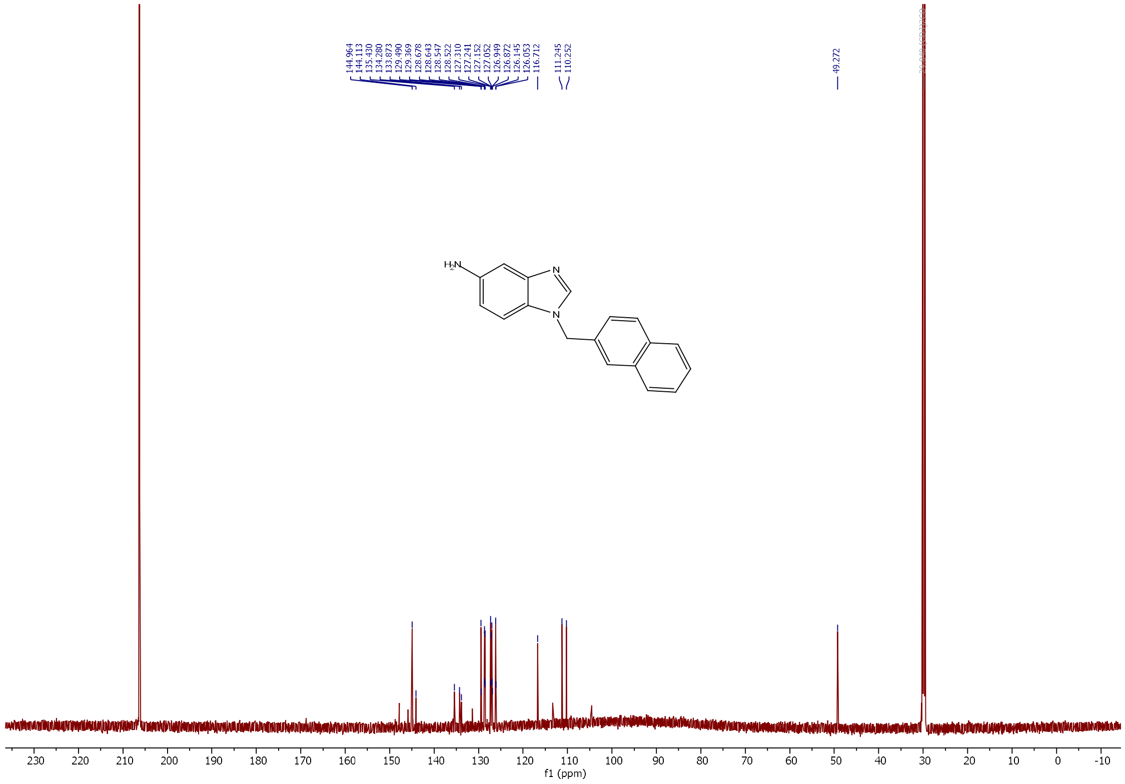

Figure S13. ^{13}C-NMR of compound **5**.

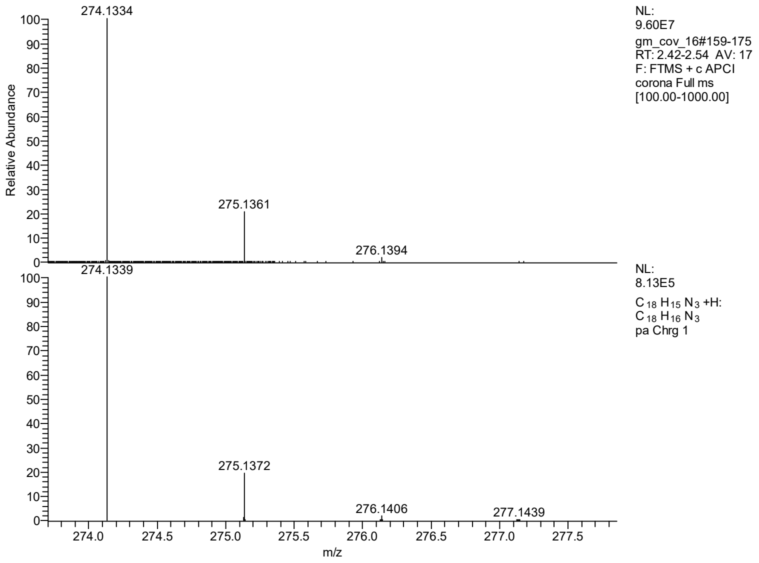

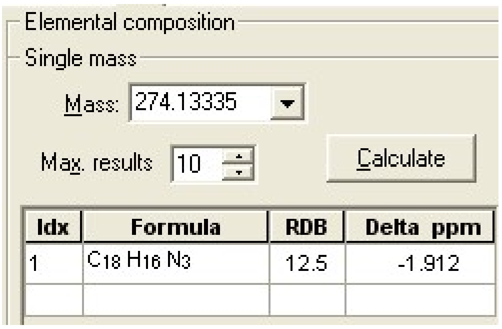

Figure S14. HRMS of compound **5**.

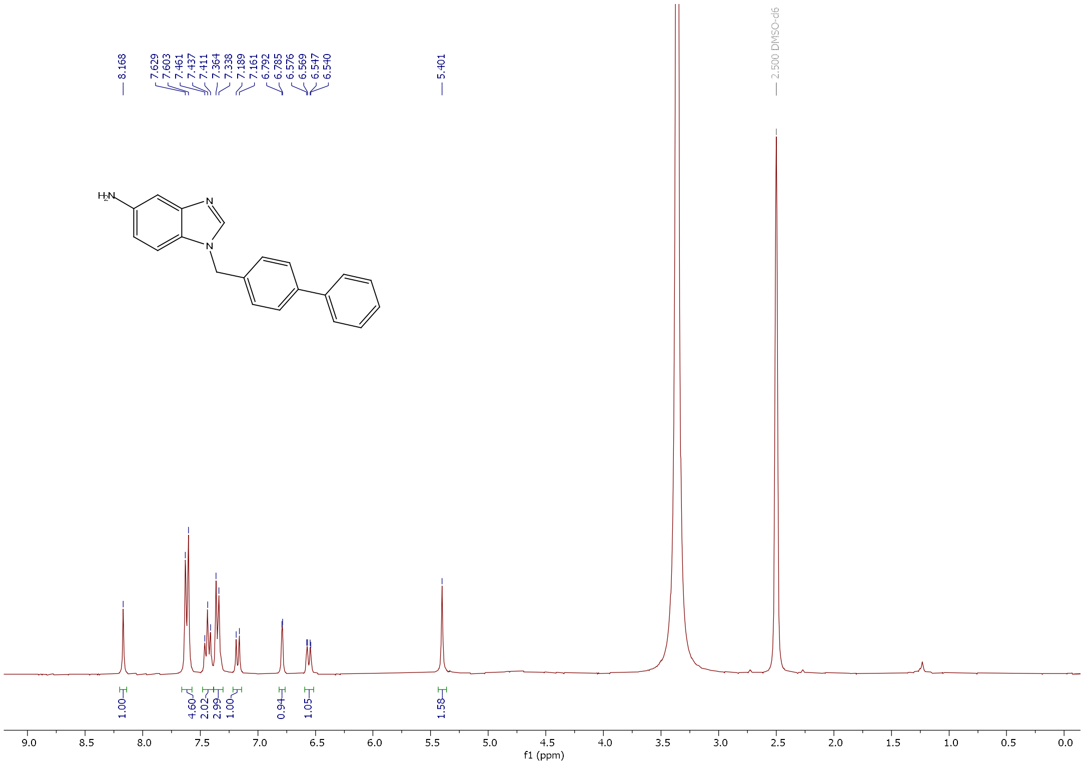

Figure S15. ^{1}H-NMR of compound **6**.

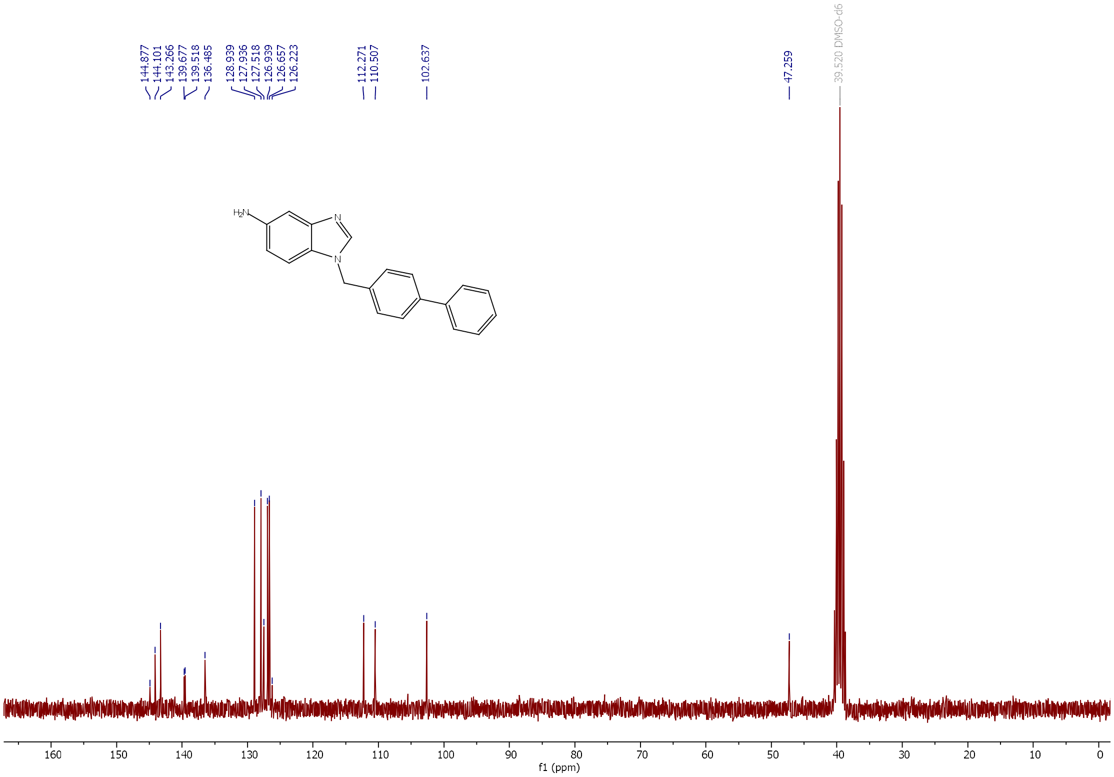

Figure S16. ^{13}C-NMR of compound **6**.

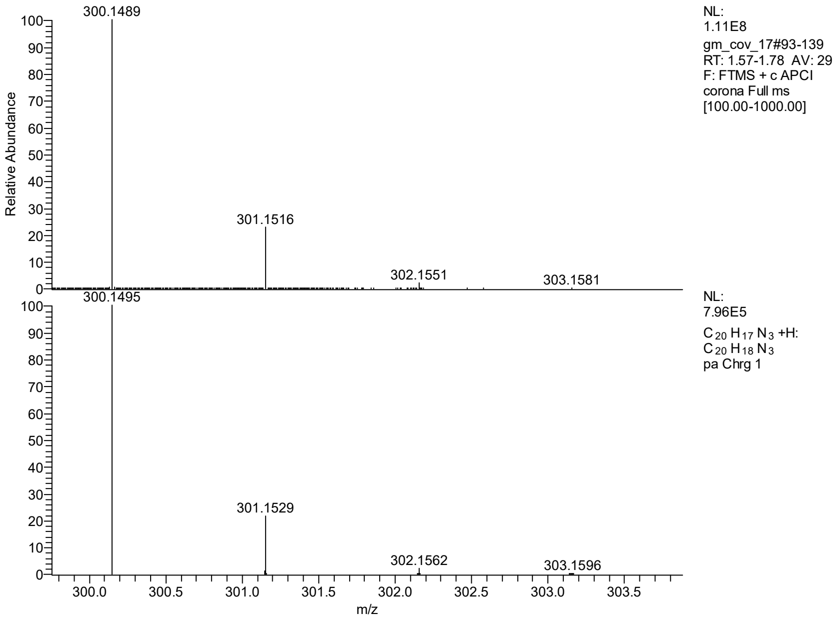

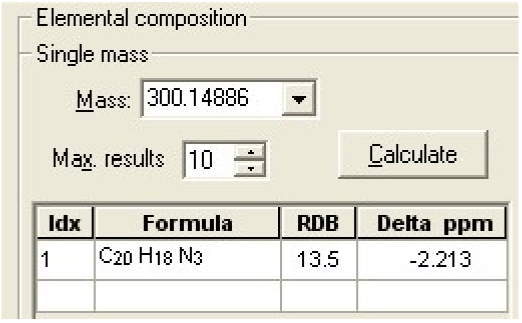
Elemental composition
Single mass
Mass: 300.14886
Max. results 10
Calculate

Idx	Formula	RDB	Delta ppm
1	$C_{20}H_{18}N_3$	13.5	-2.213

Figure S17. HRMS of compound **6**.

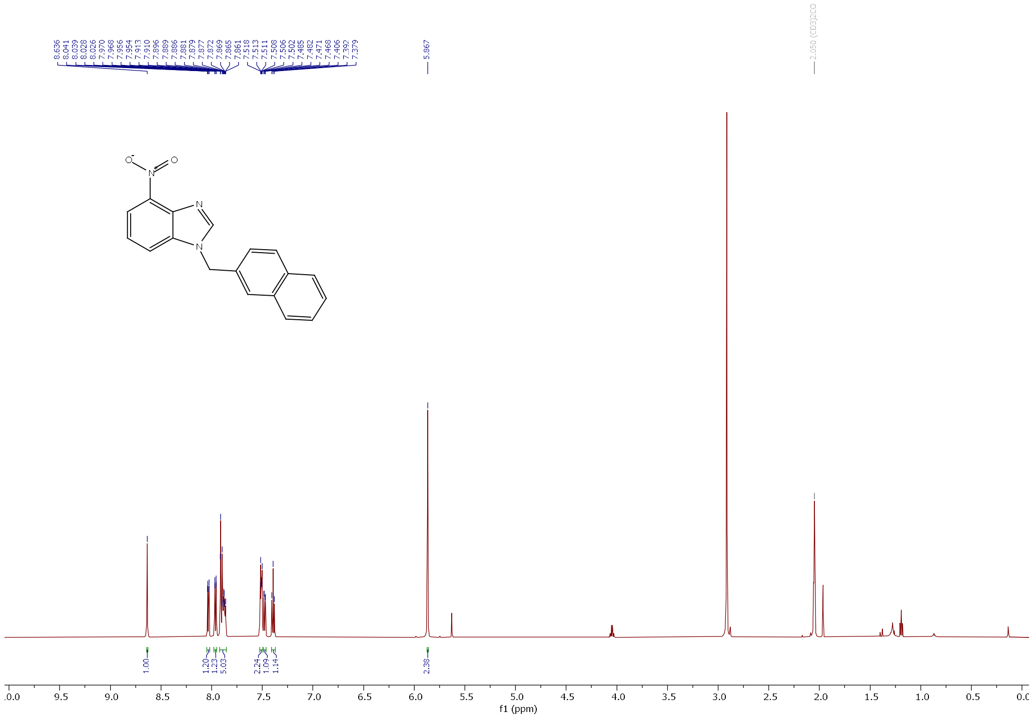

Figure S18. ^{1}H-NMR of compound **11**.

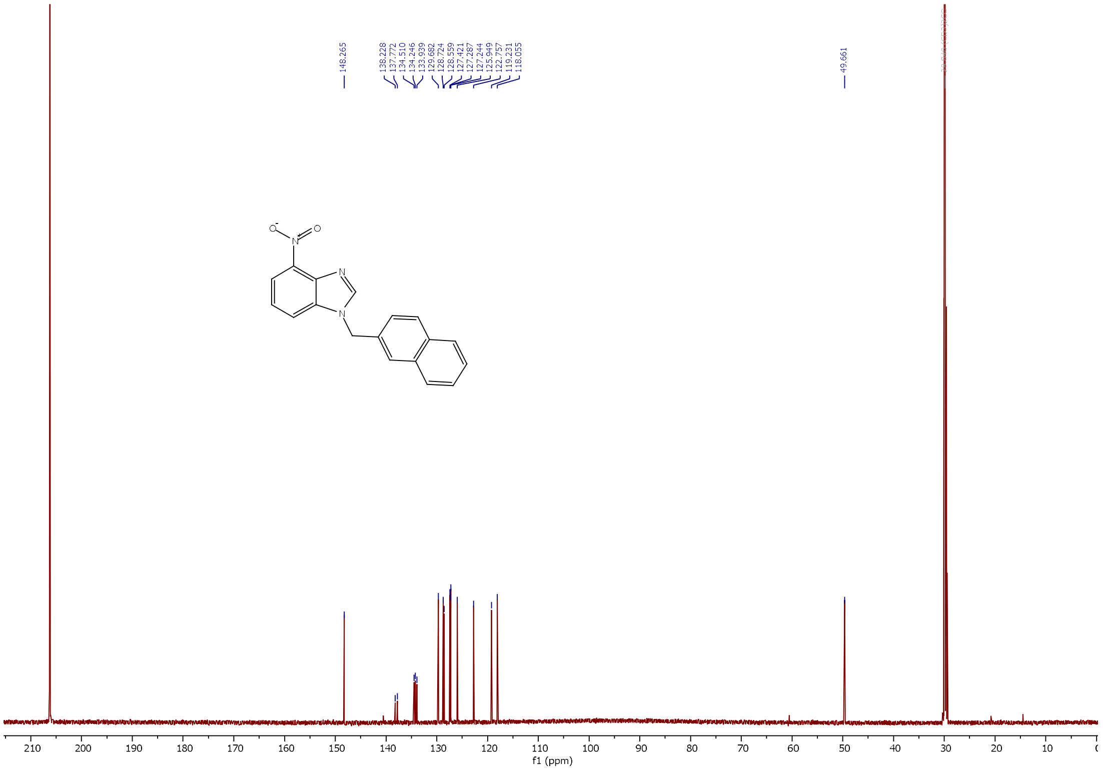

Figure S19. ^{13}C-NMR of compound **11**.

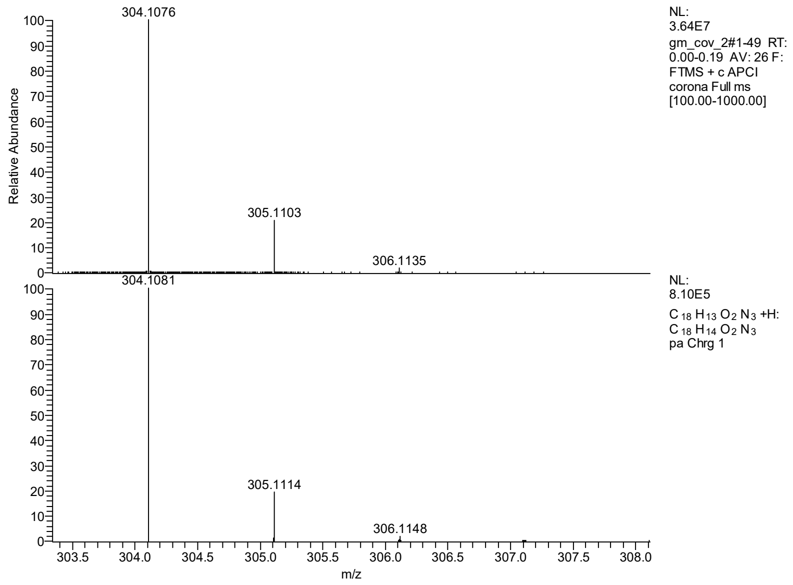

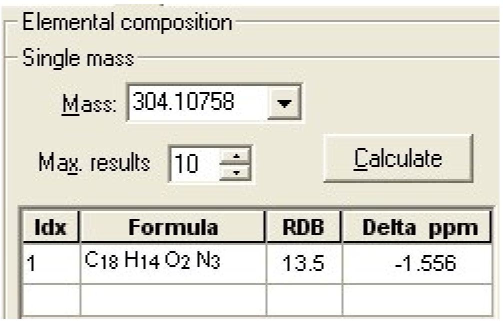

Figure S20. HRMS of compound **11**.

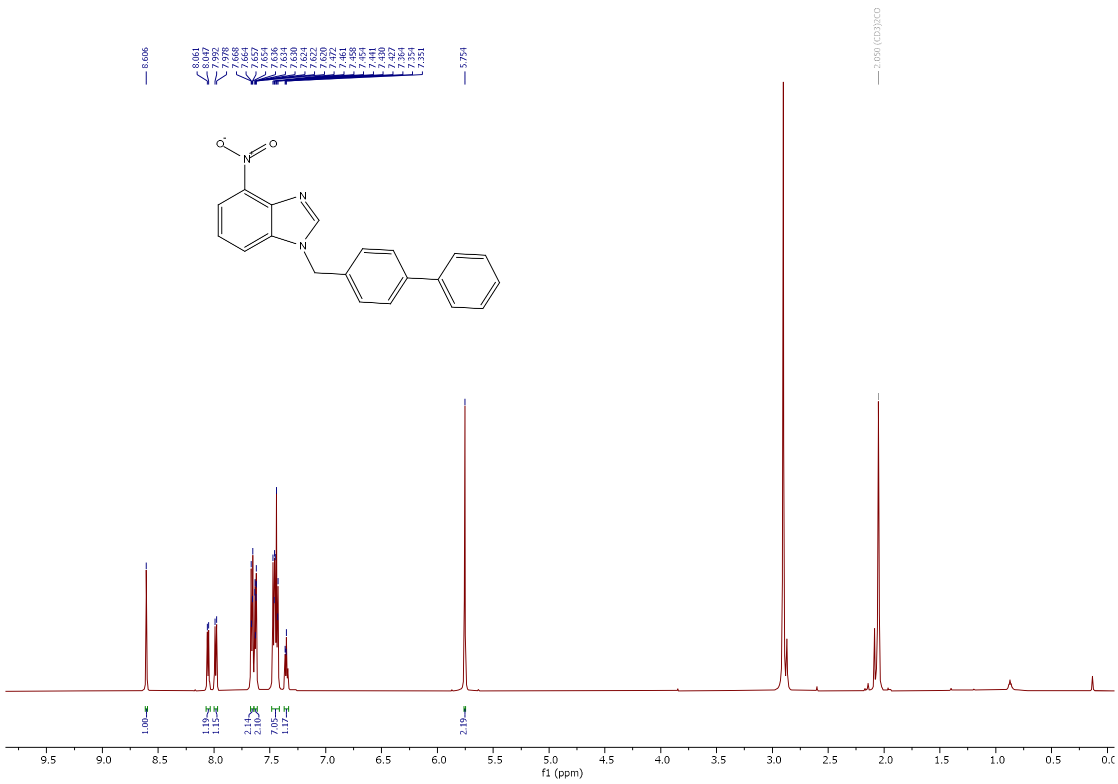

Figure S21. ^{1}H-NMR of compound **12**.

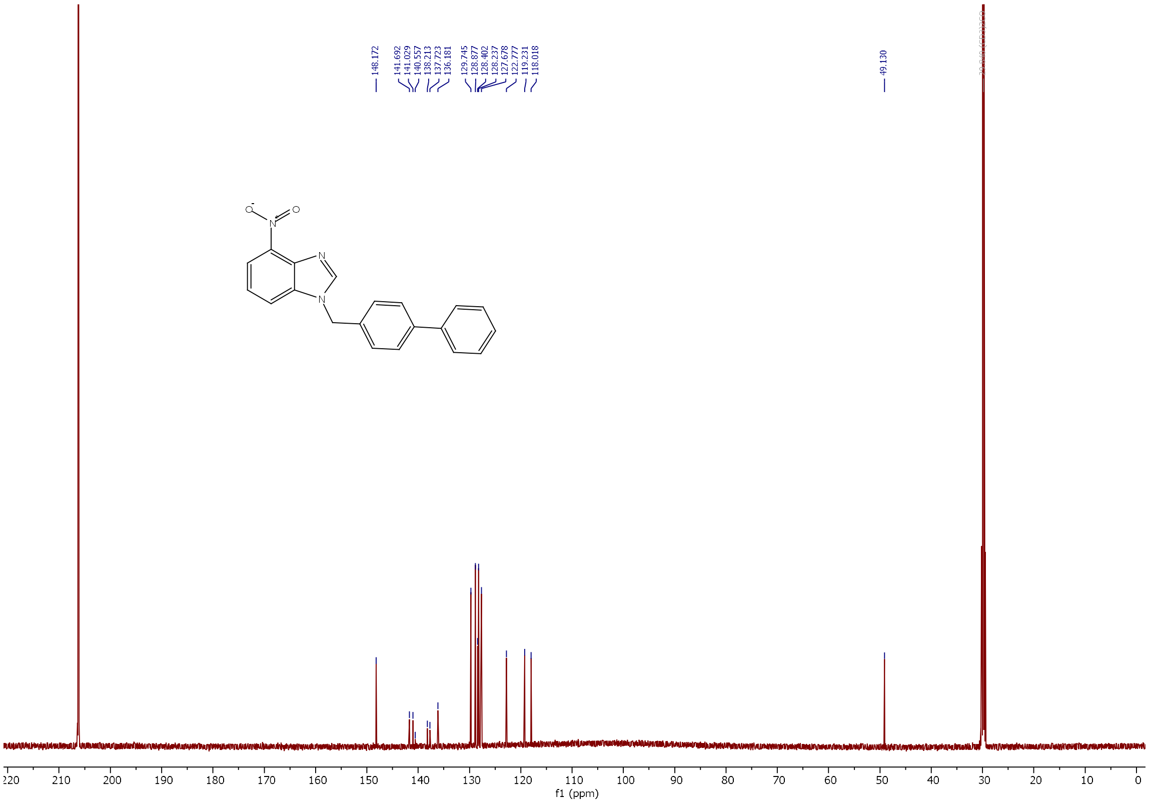

Figure S22. ^{13}C-NMR of compound **12**.

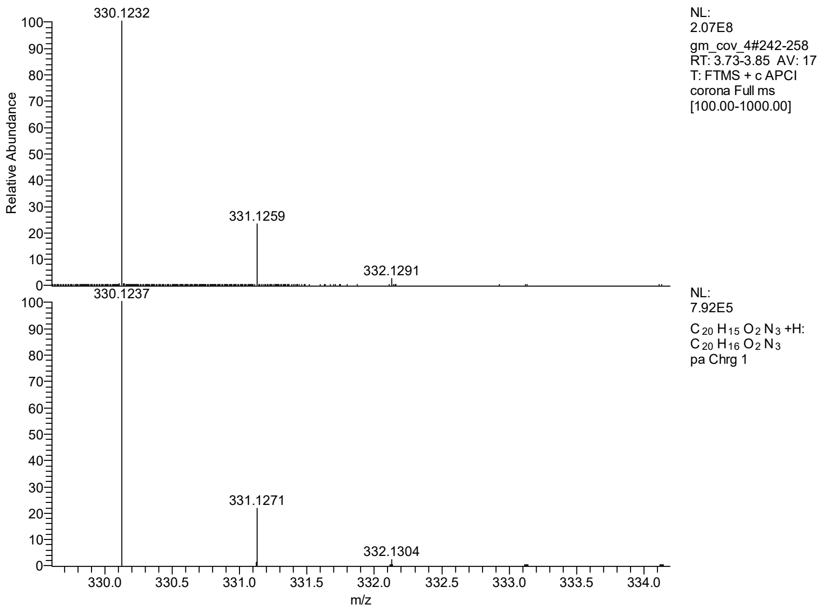

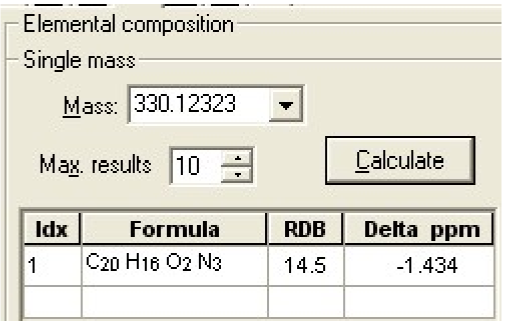

Figure S23. HRMS of compound **12**.

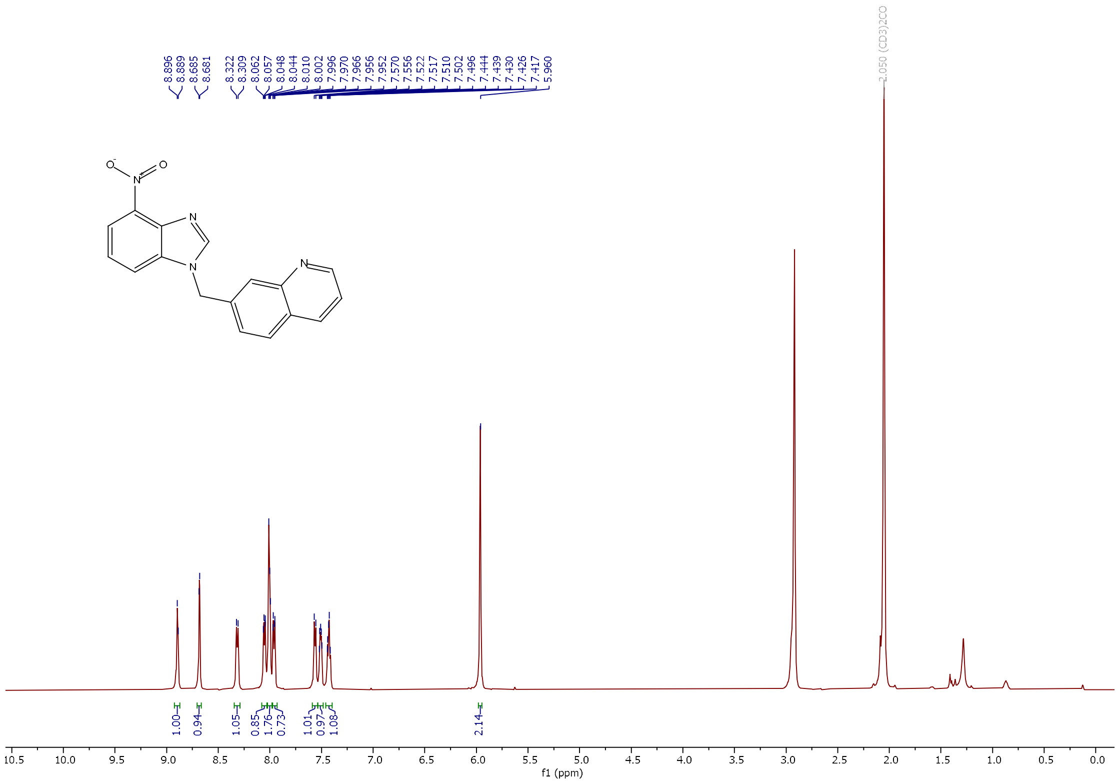

Figure S24. ^{1}H-NMR of compound **13**.

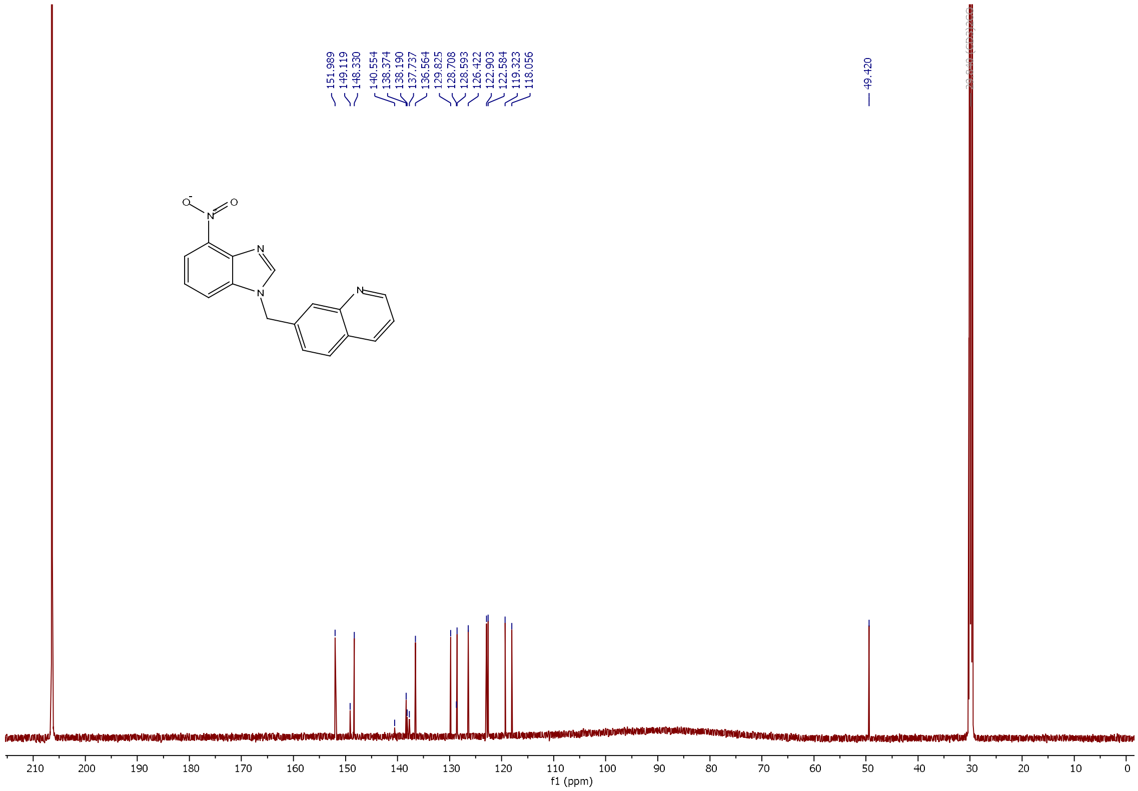

Figure S25. ^{13}C-NMR of compound **13**.

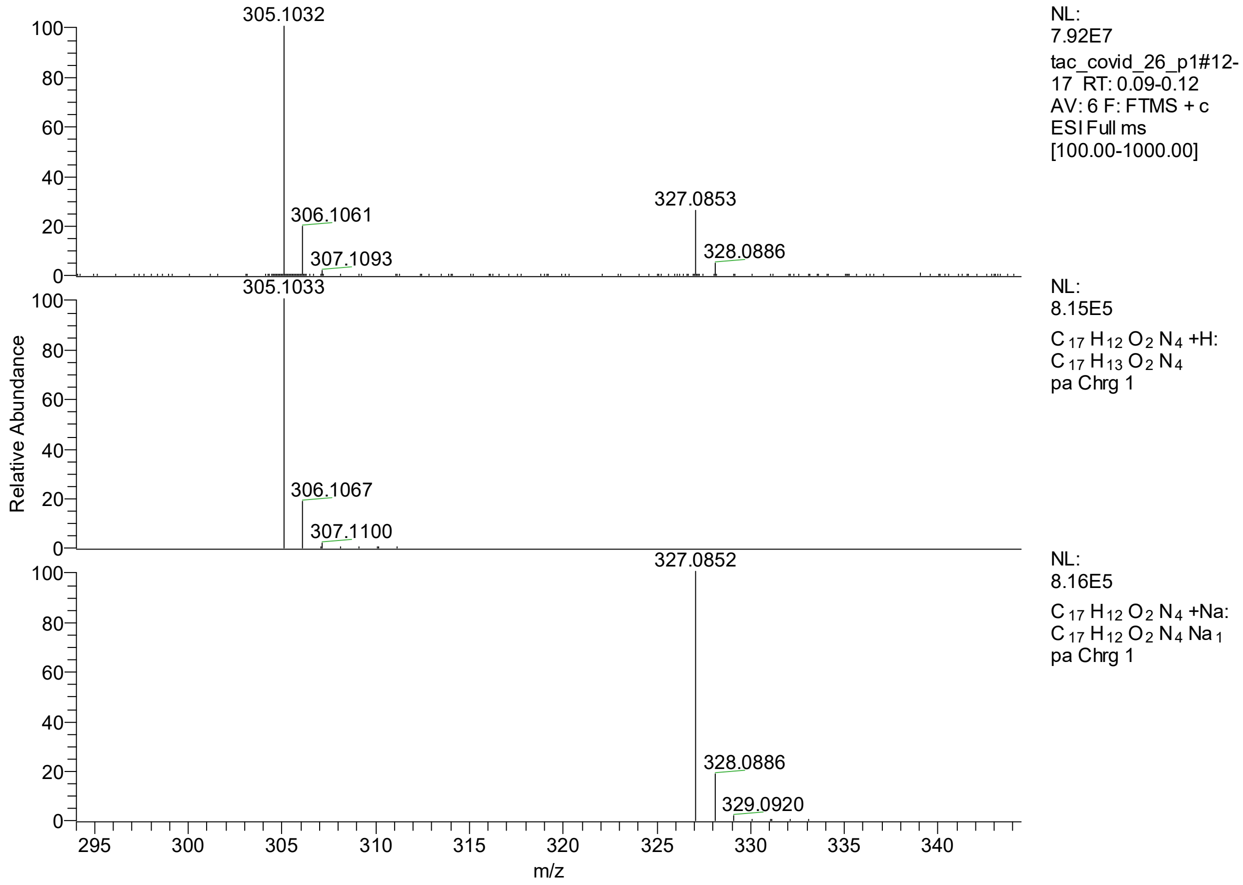

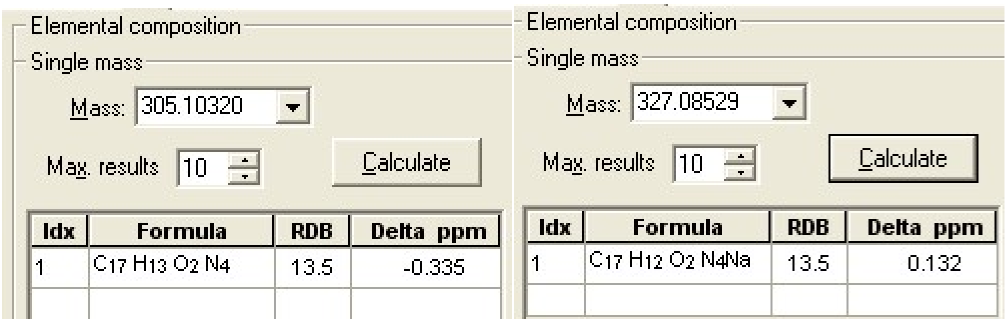

Elemental composition
Single mass
Mass: 305.10320
Max. results 10
Calculate

Idx	Formula	RDB	Delta ppm
1	$C_{17}H_{13}O_2N_4$	13.5	-0.335

Elemental composition
Single mass
Mass: 327.08529
Max. results 10
Calculate

Idx	Formula	RDB	Delta ppm
1	$C_{17}H_{12}O_2N_4Na$	13.5	0.132

Figure S26. HRMS of compound **13**.

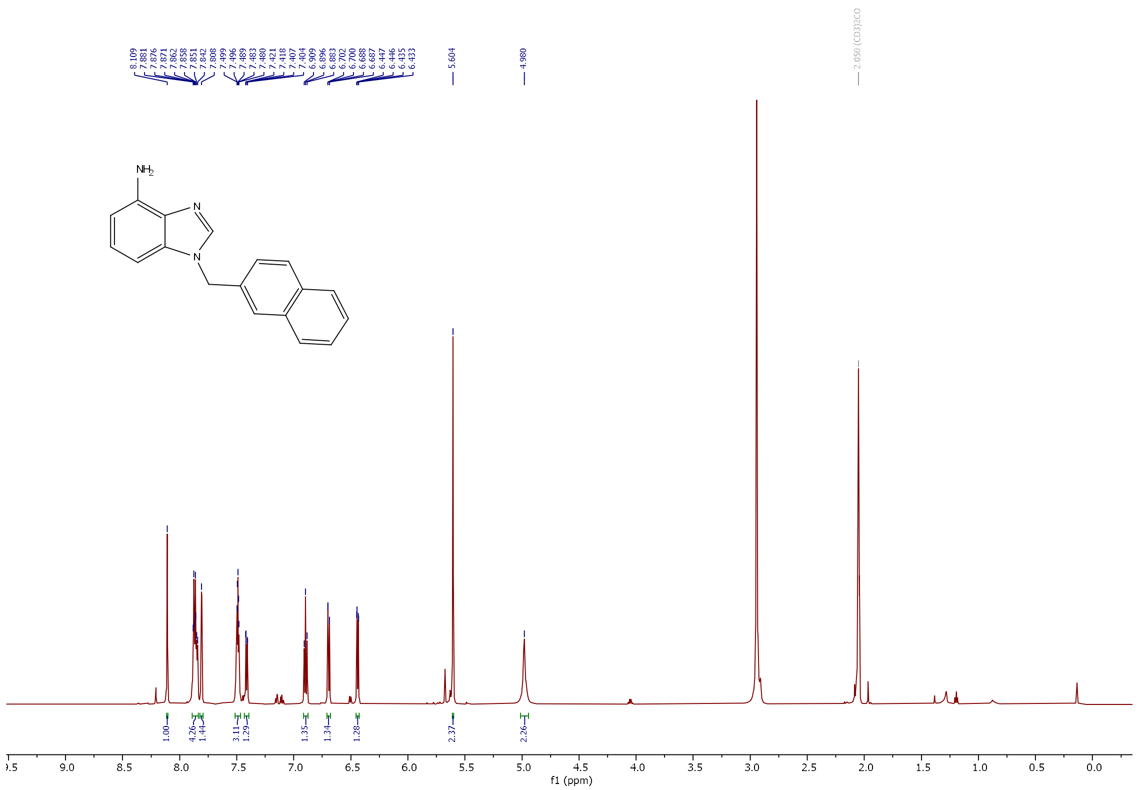

Figure S27. ^{1}H-NMR of compound **14**.

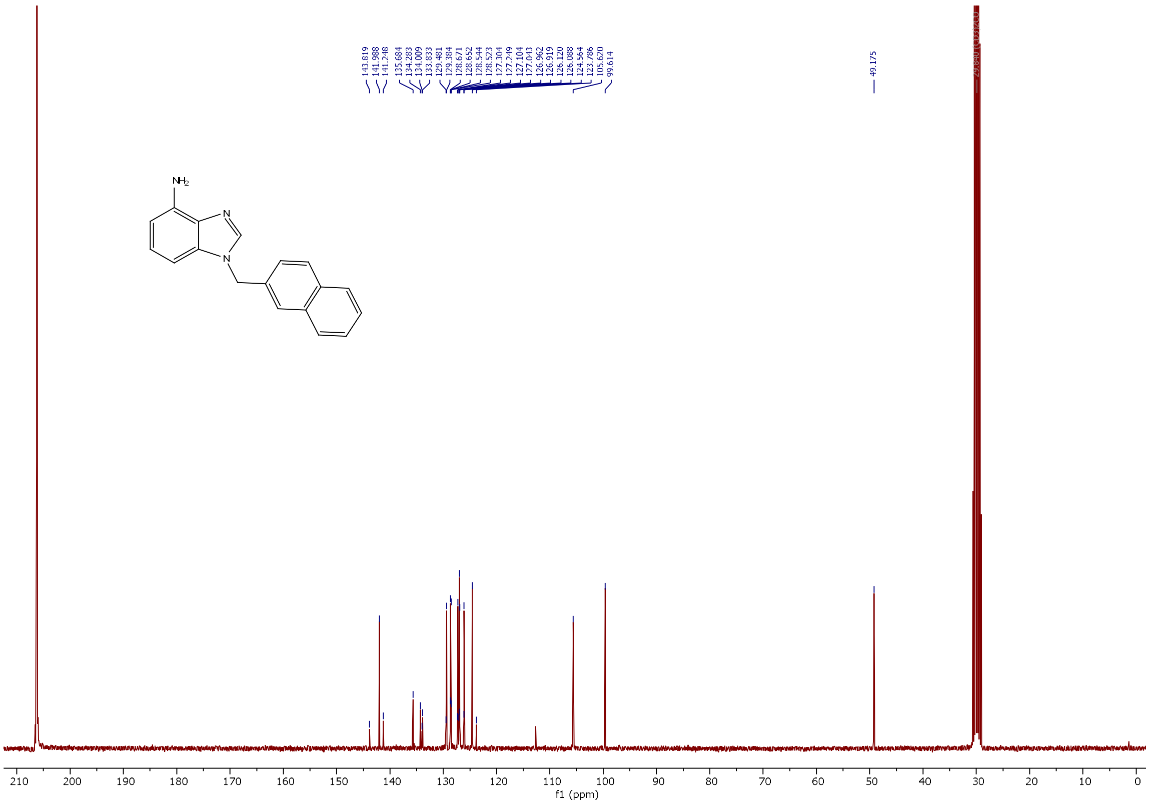

Figure S28. ^{13}C-NMR of compound **14**.

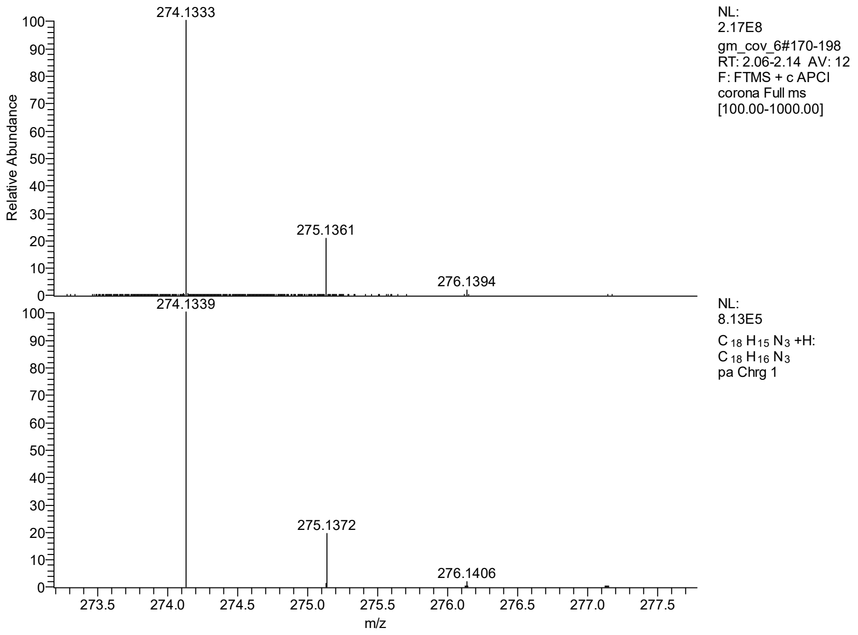

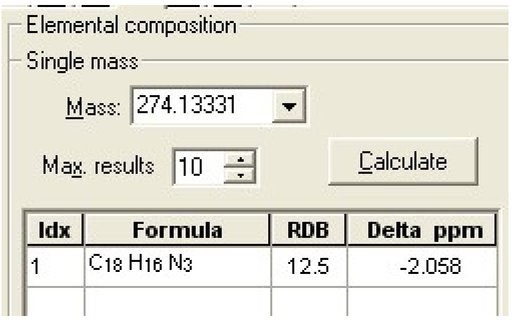

Figure S29. HRMS of compound **14**.

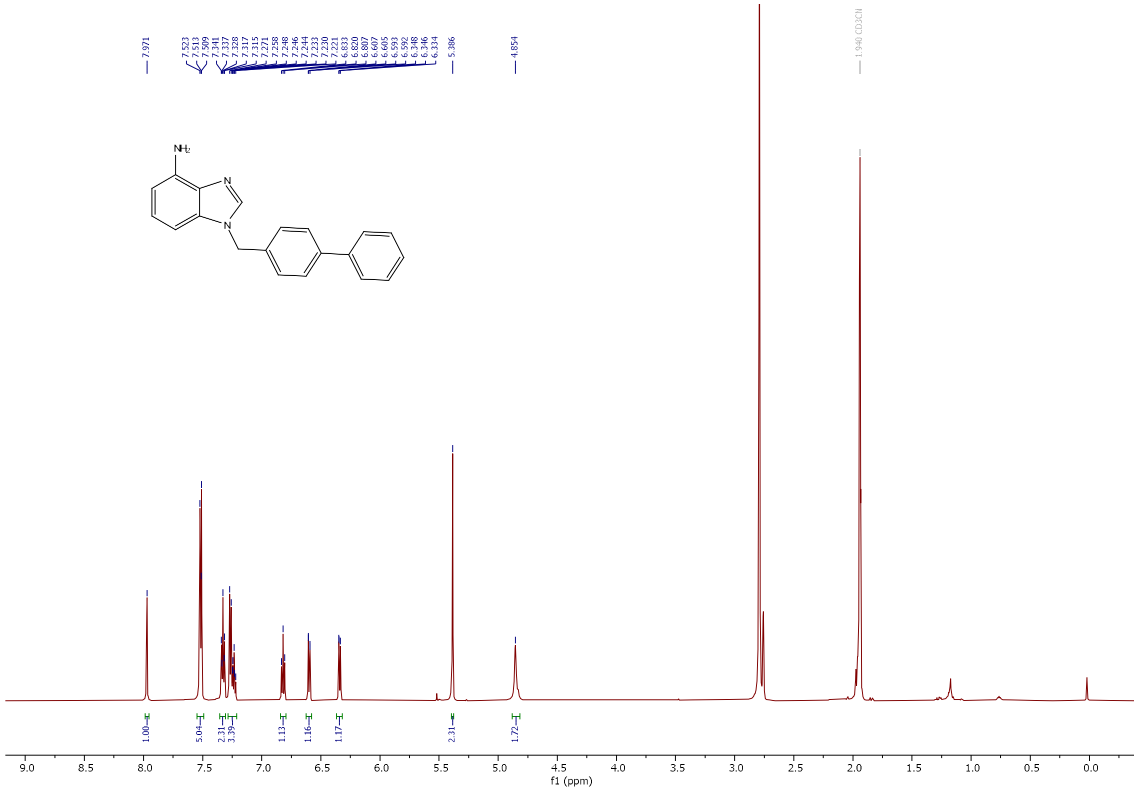

Figure S30. ^{1}H-NMR of compound **15**.

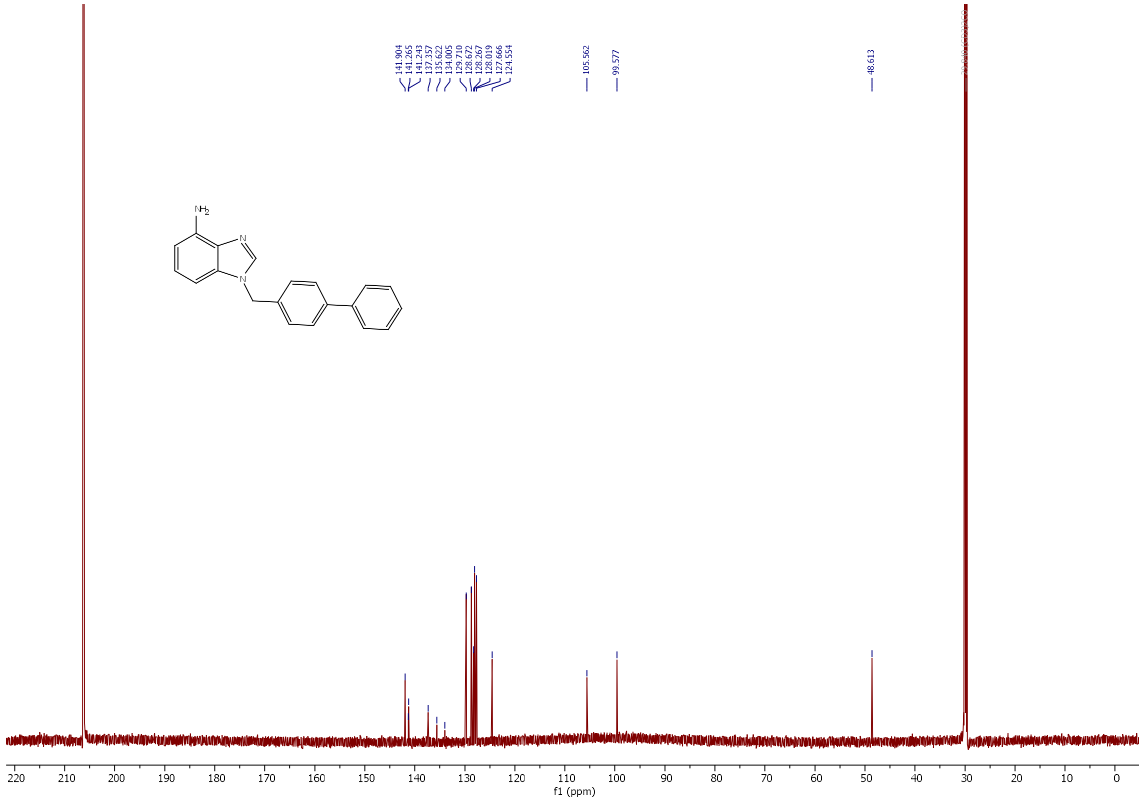

Figure S31. ^{13}C-NMR of compound **15**.

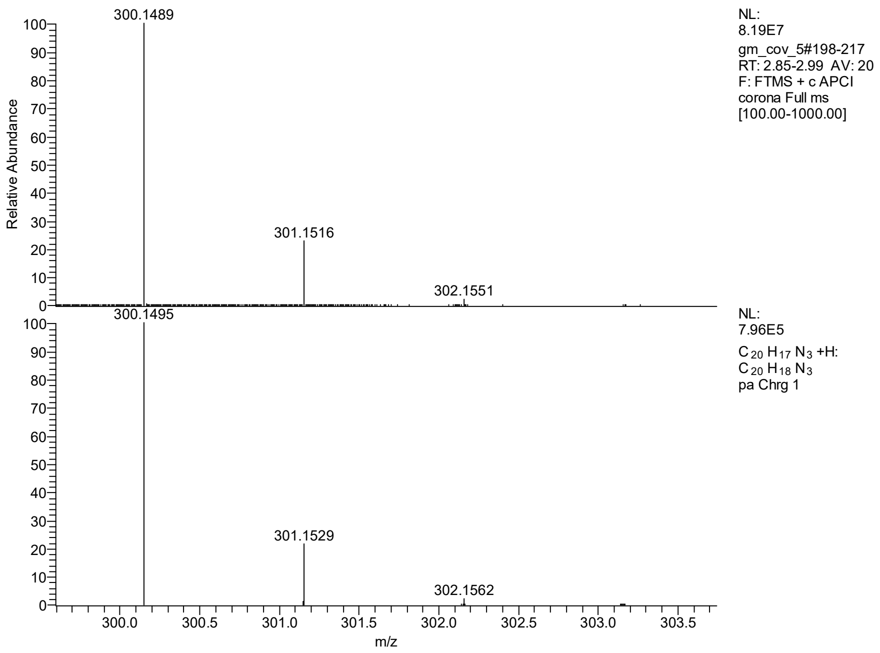

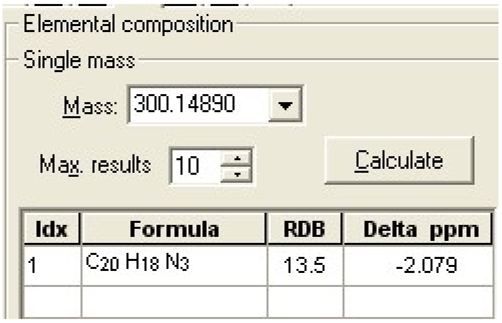
Elemental composition

Single mass

Mass: 300.14890

Max. results 10

Calculate

Idx	Formula	RDB	Delta ppm
1	$C_{20}H_{18}N_3$	13.5	-2.079

Figure S32. HRMS of compound **15**.

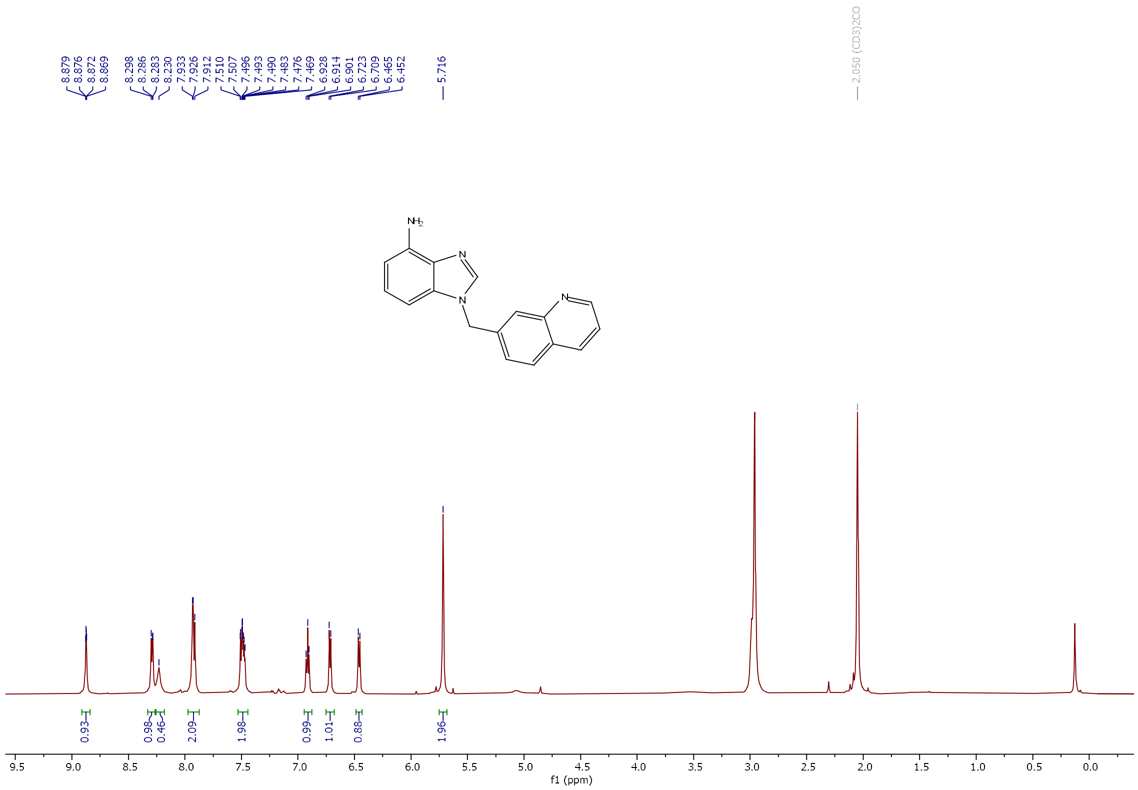

Figure S33. ^{1}H-NMR of compound **16**.

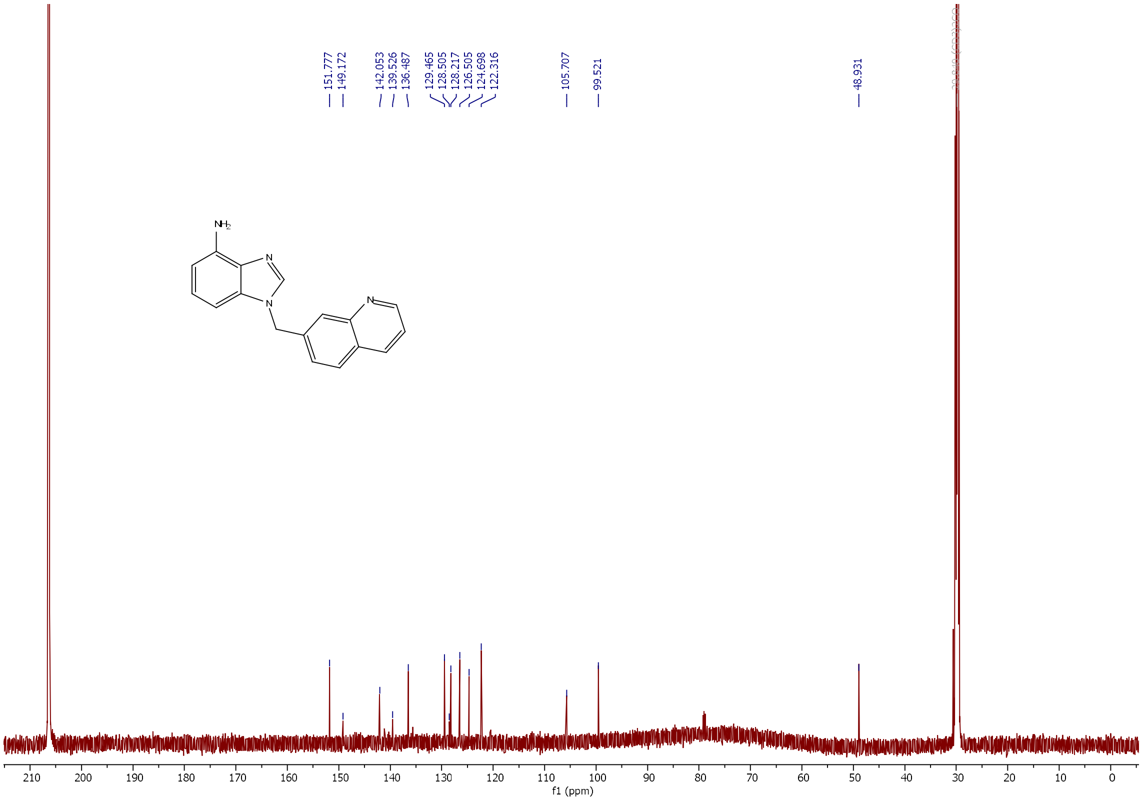

Figure S34. ^{13}C-NMR of compound **16**.

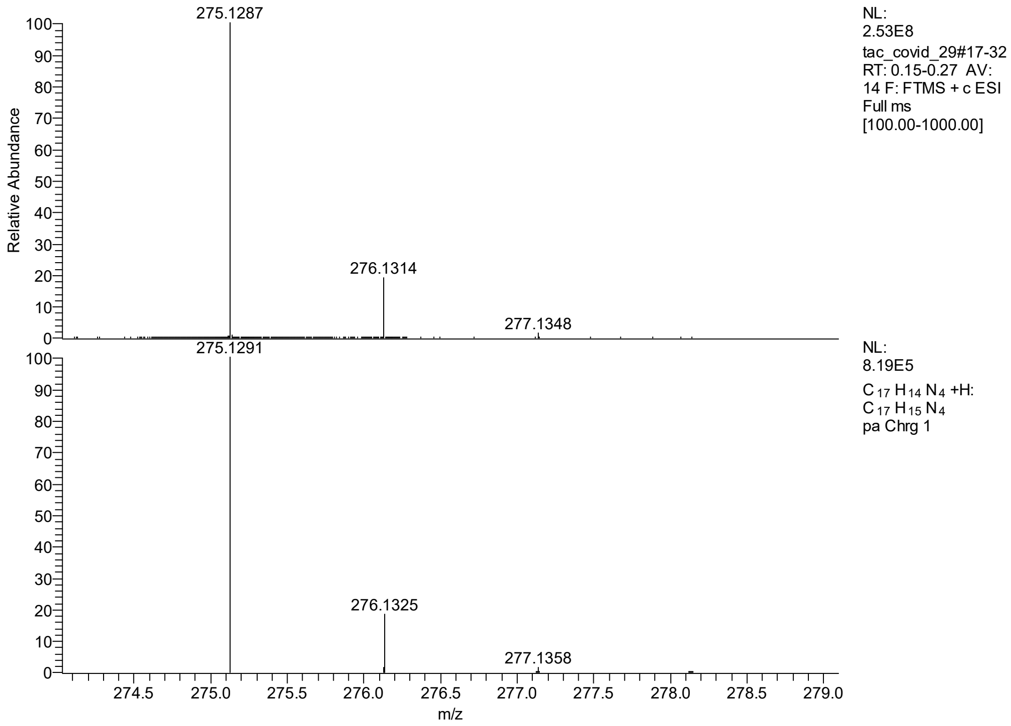

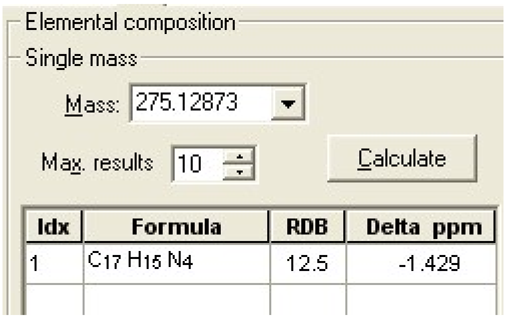

Idx	Formula	RDB	Delta ppm
1	$C_{17}H_{15}N_4$	12.5	-1.429

Figure S35. HRMS of compound **16**.

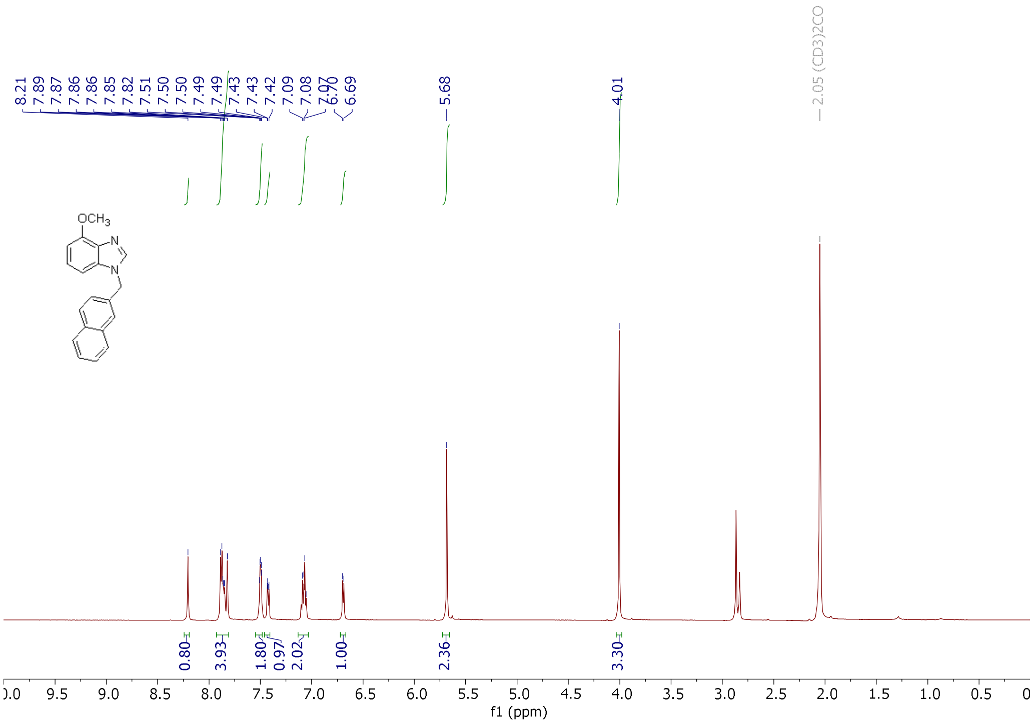

Figure S36. ^{1}H-NMR of compound **28**.

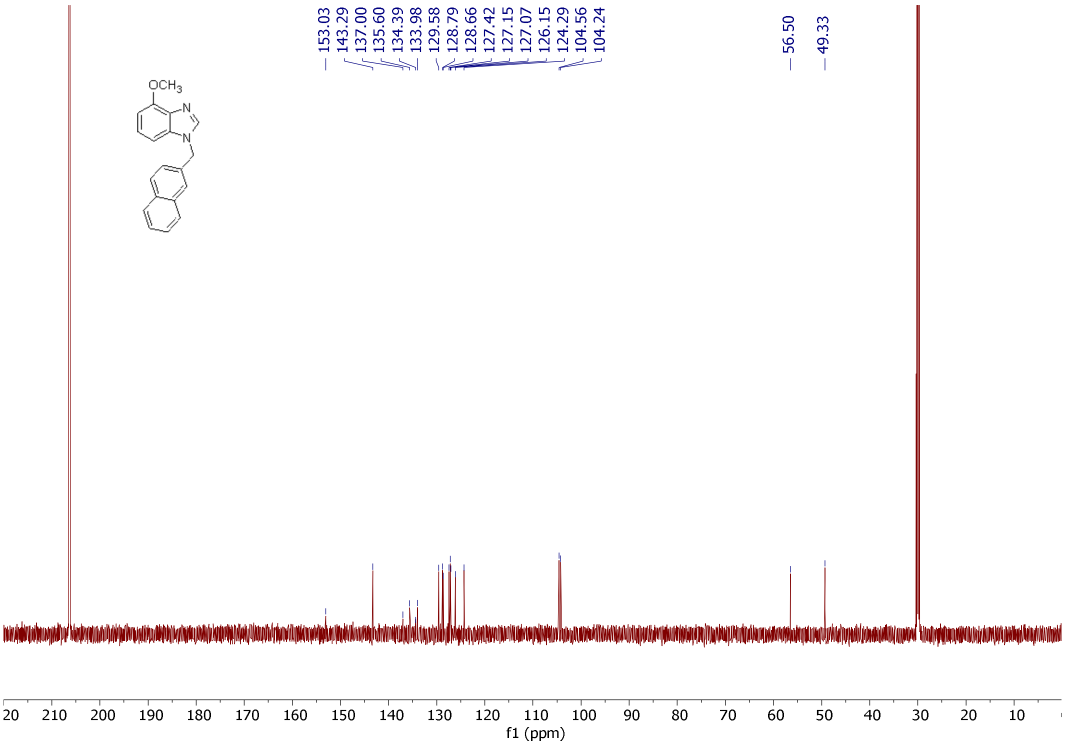

Figure S37. ^{13}C-NMR of compound **28**.

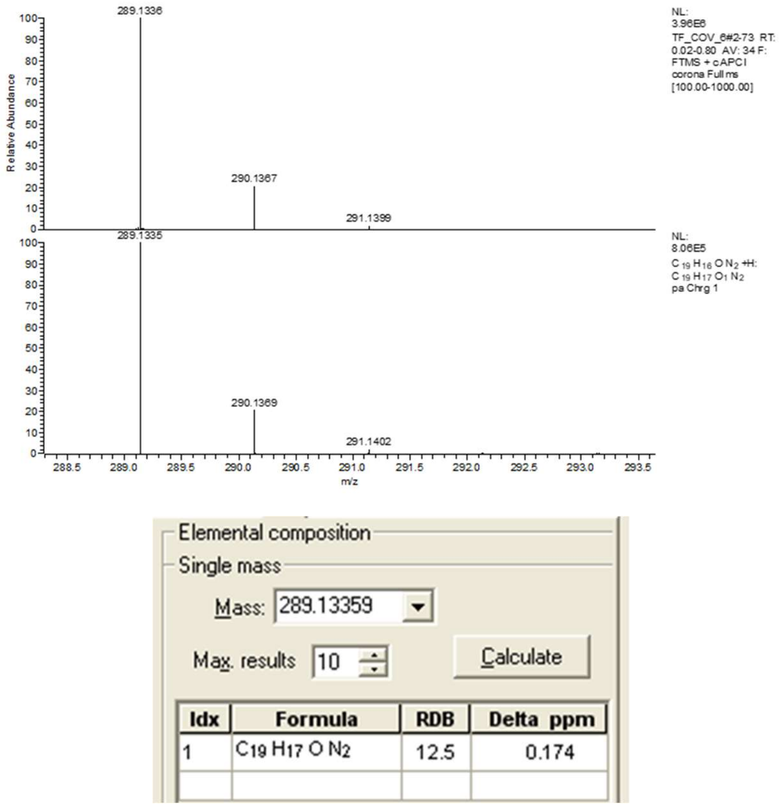

Figure S38. HRMS of compound **28**.

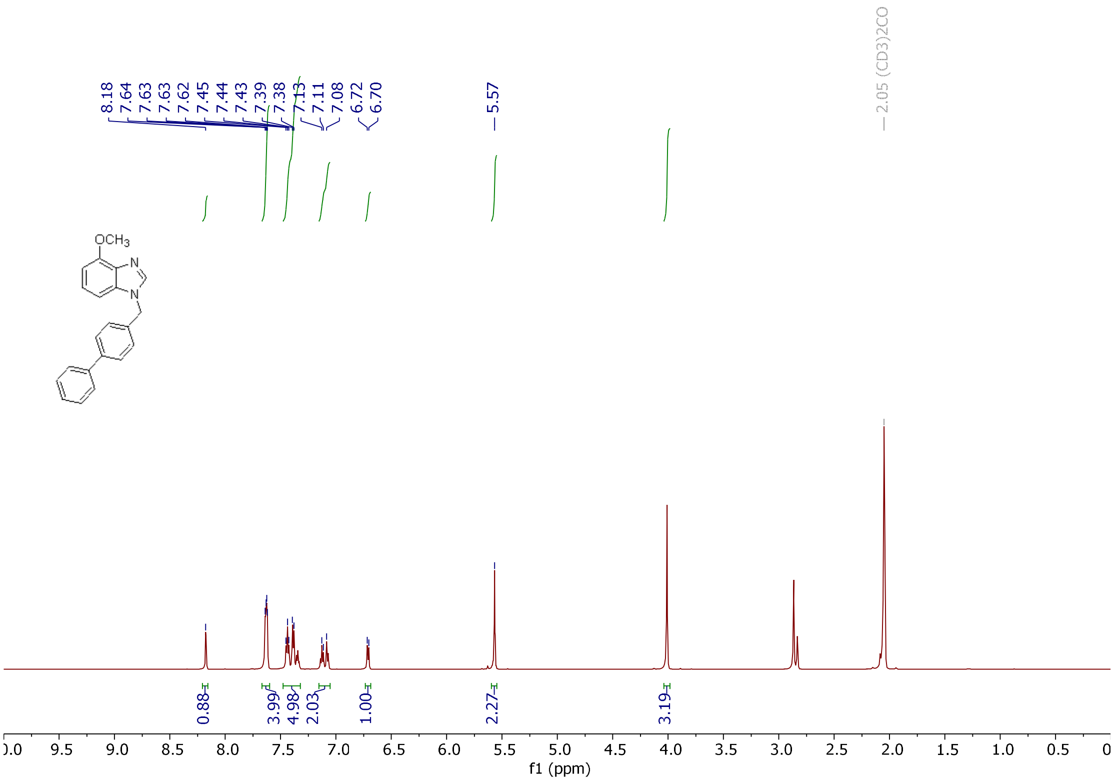

Figure S39. ^{1}H-NMR of compound **29**.

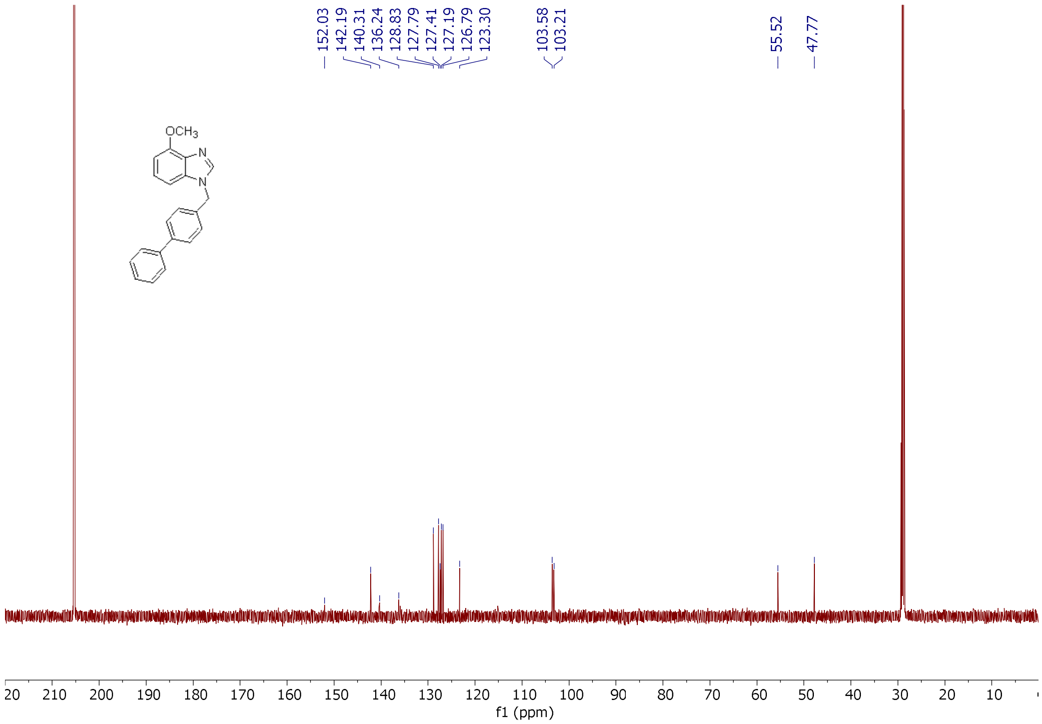

Figure S40. ^{13}C-NMR of compound **29**.

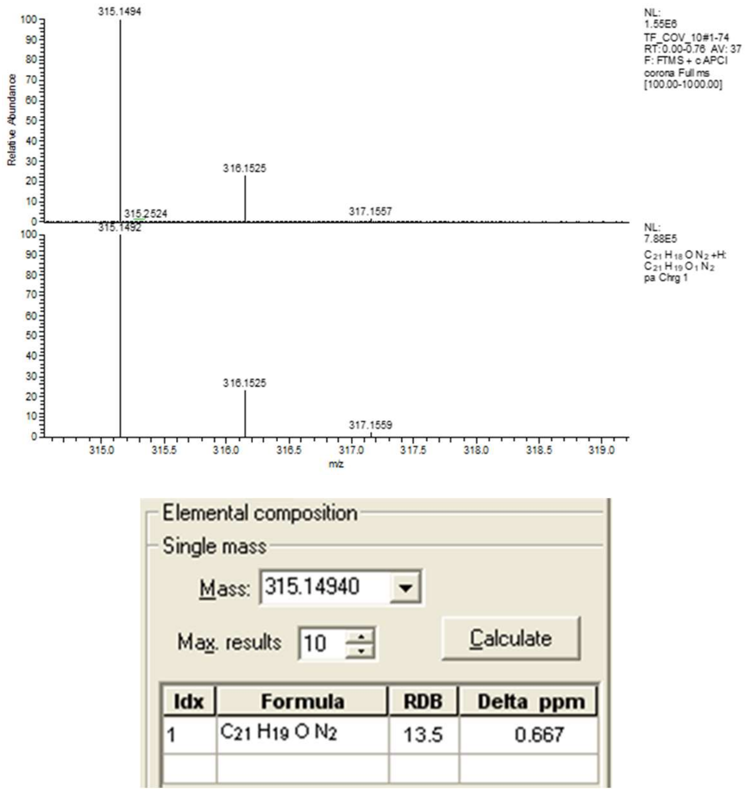

Figure S41. HRMS of compound **29**.

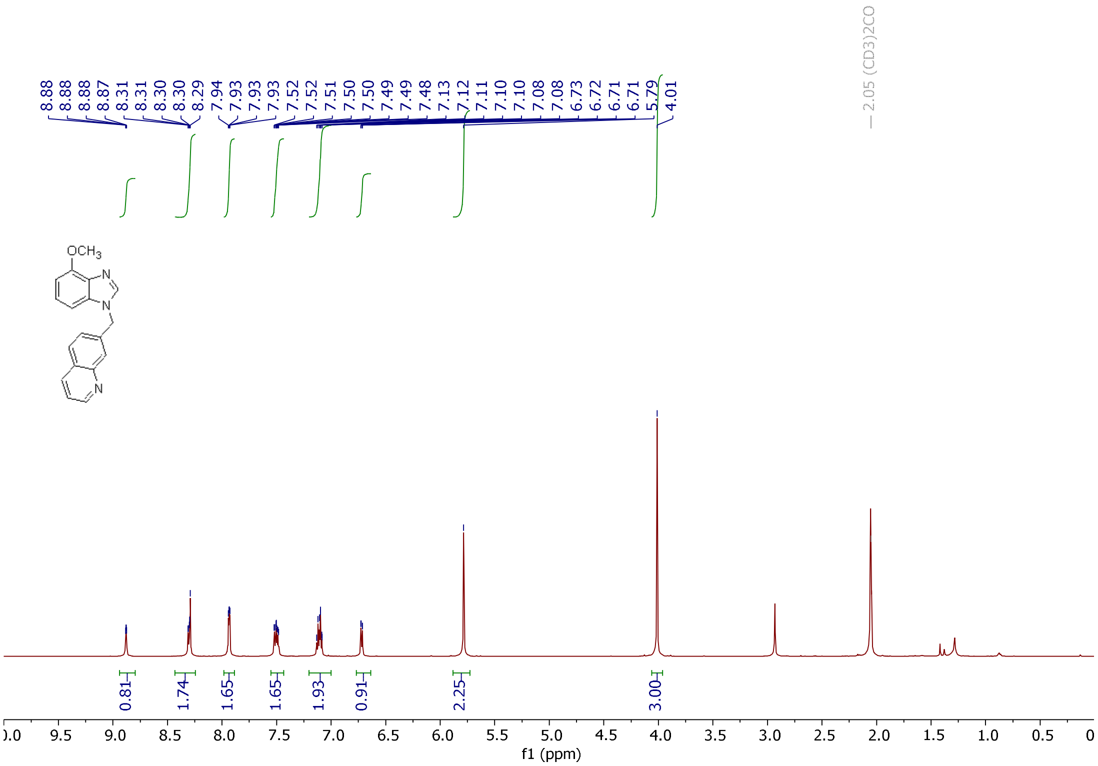

Figure S42. ^{1}H-NMR of compound **30**.

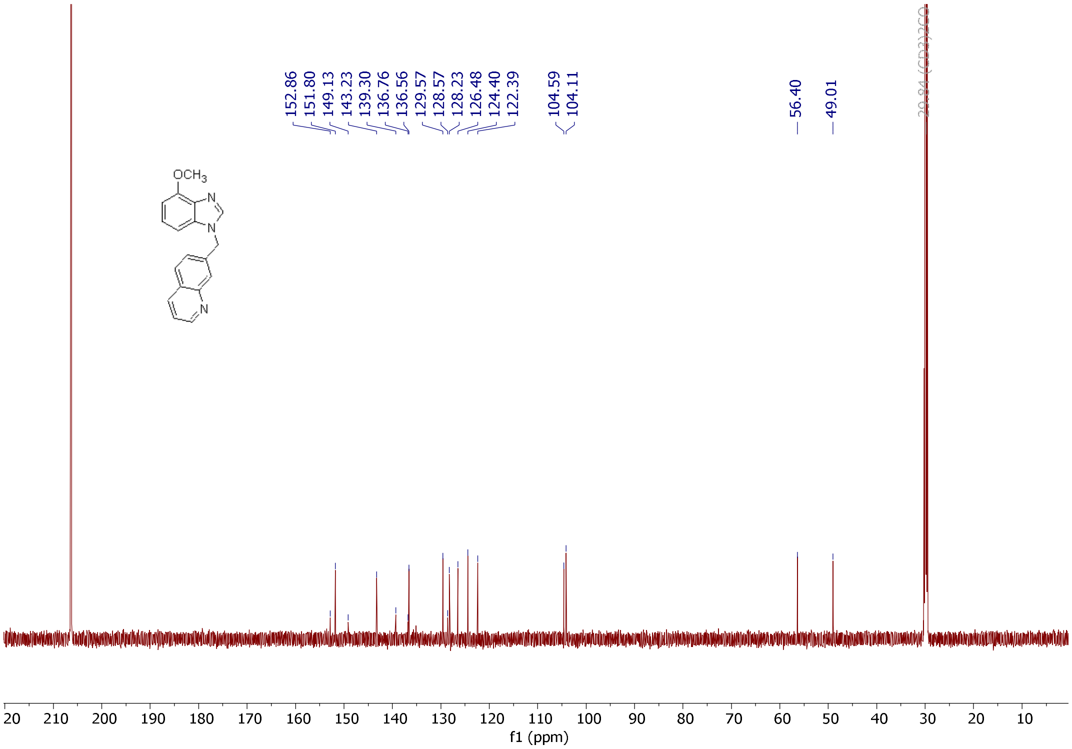

Figure S43. ^{13}C-NMR of compound **30**.

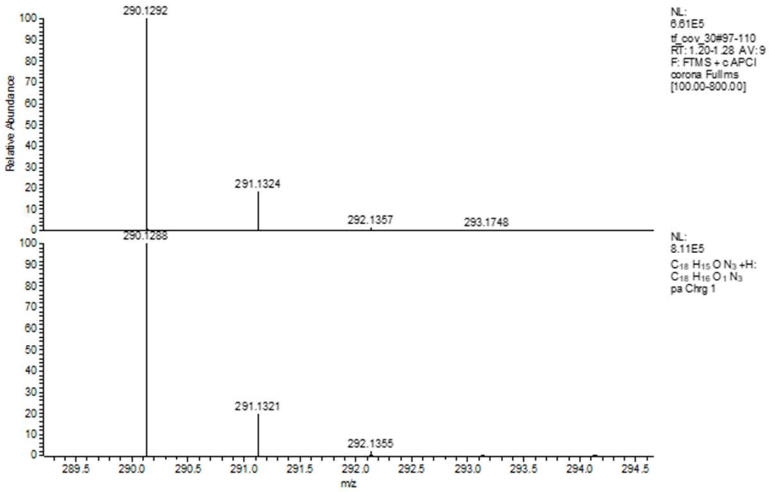

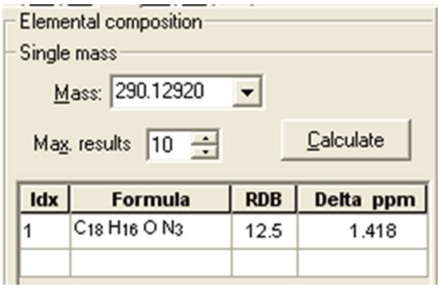

Idx	Formula	RDB	Delta ppm
1	$C_{18}H_{16}ON_3$	12.5	1.418

Figure S44. HRMS of compound **30**.

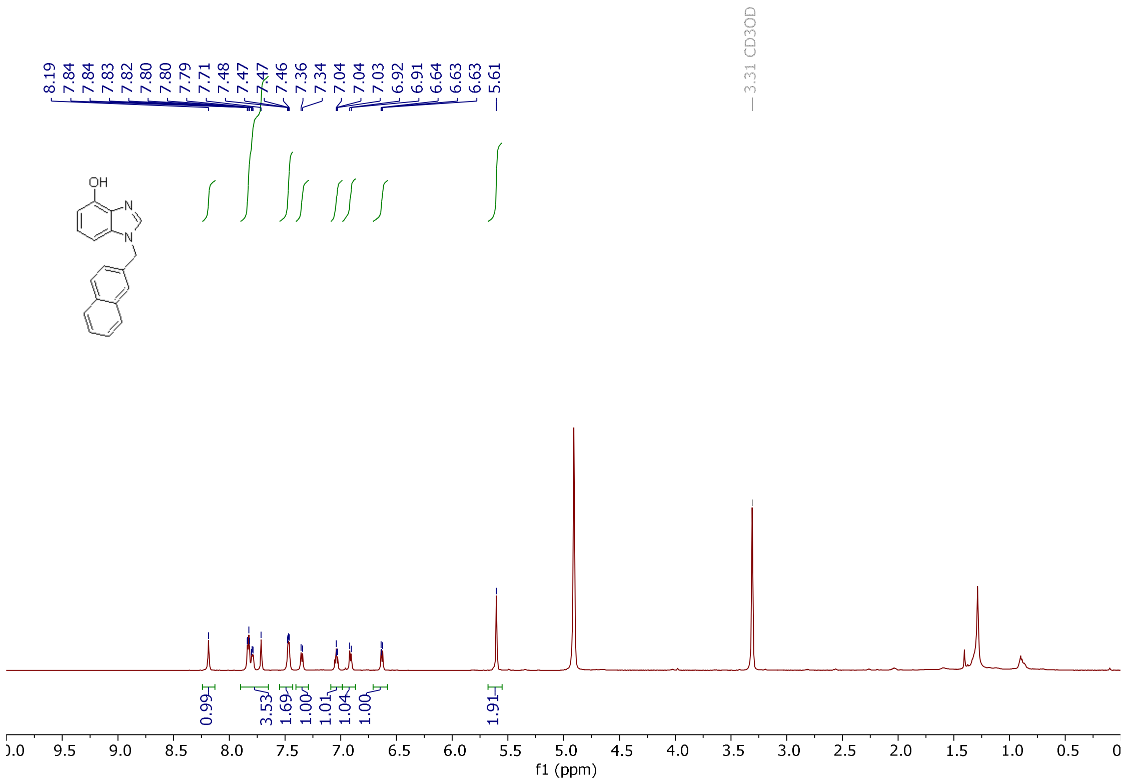

Figure S45. ^{1}H-NMR of compound **31**.

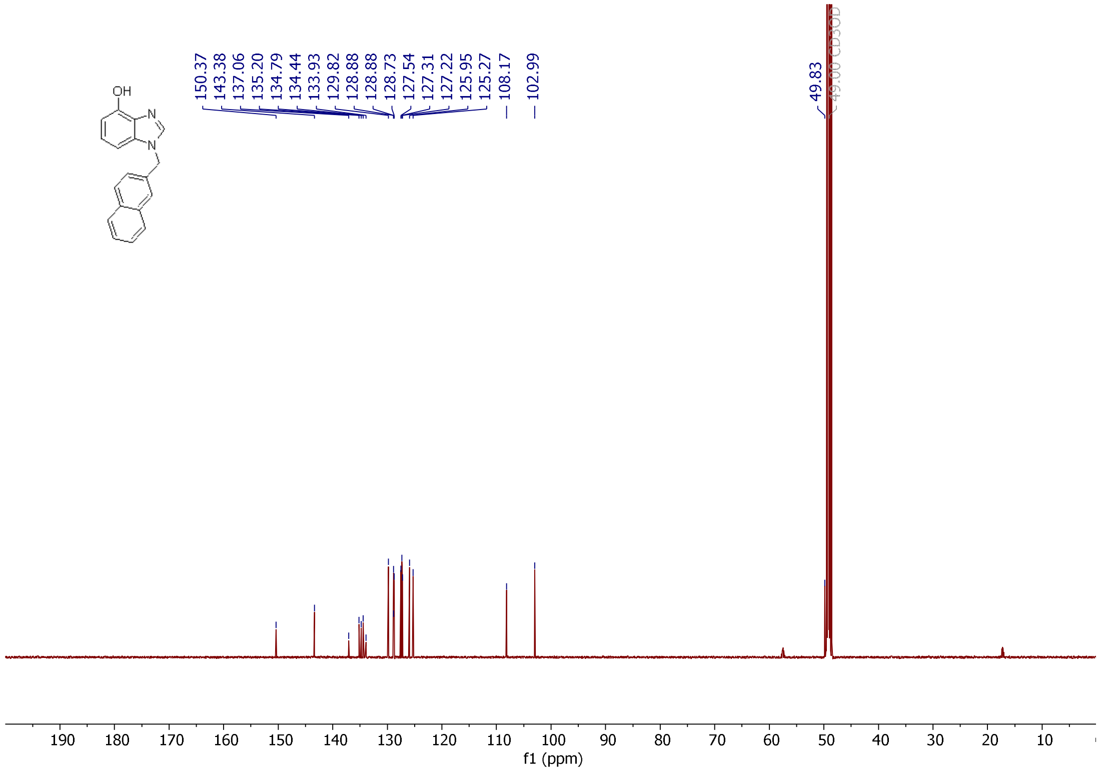

Figure S46. ^{13}C-NMR of compound **31**.

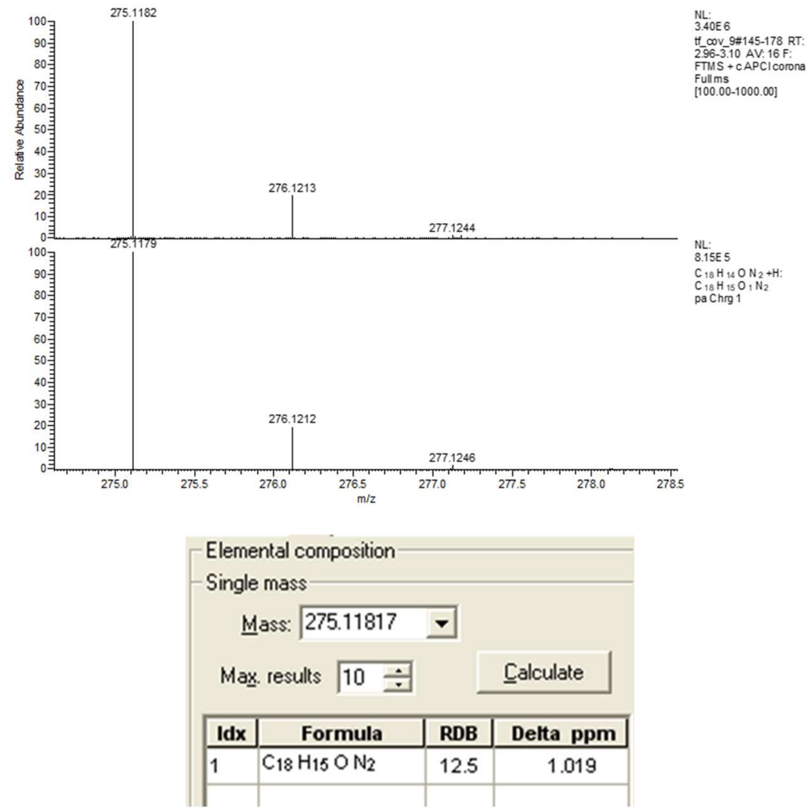

Figure S47. HRMS of compound **31**.

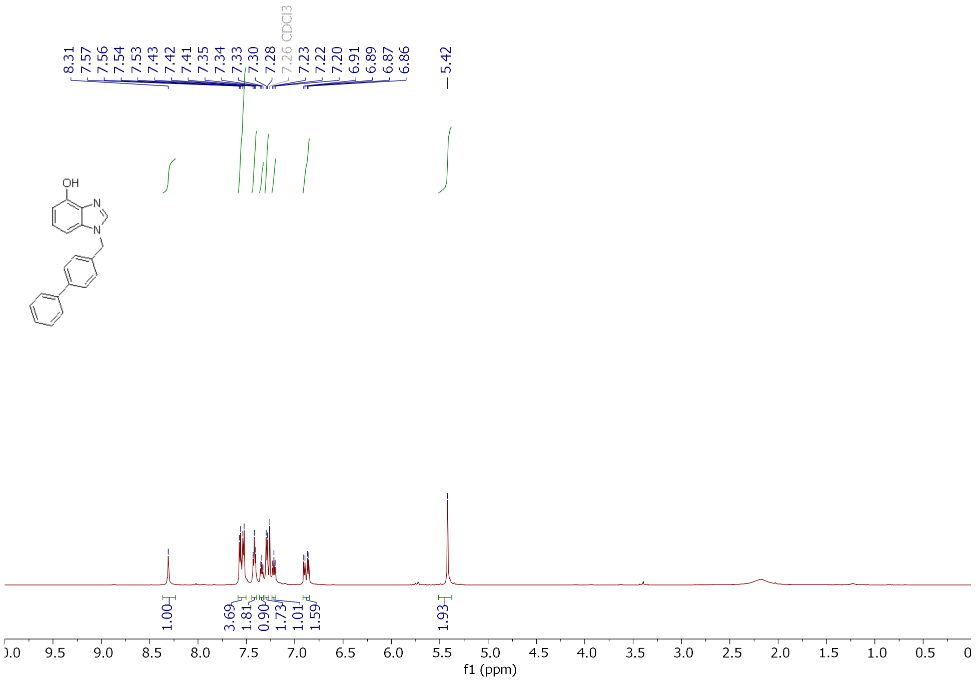

Figure S48. ^{1}H-NMR of compound **32**.

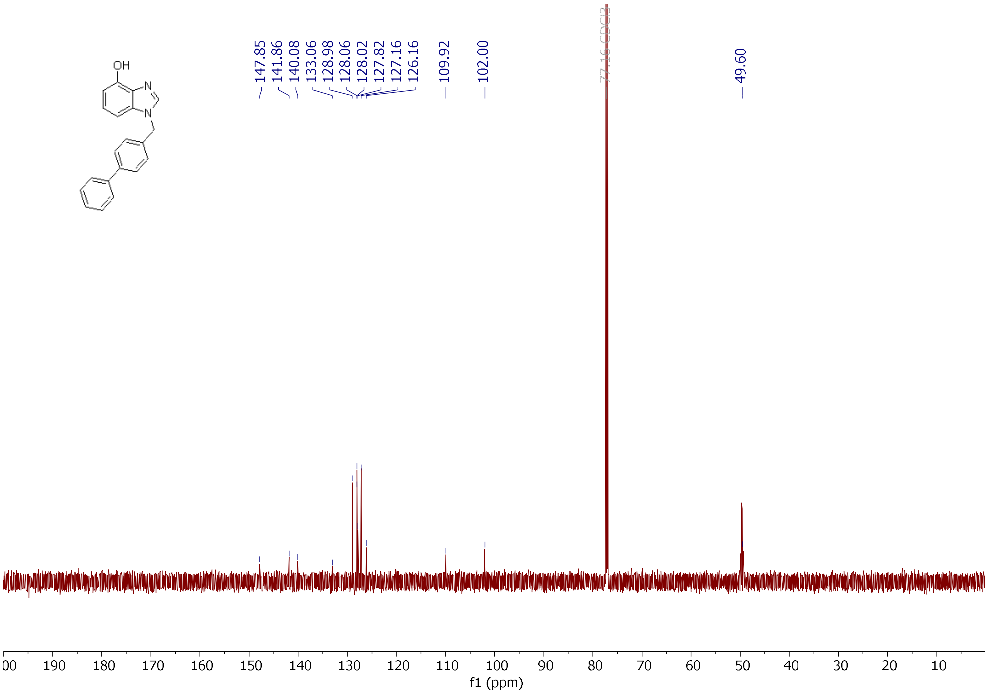

Figure S49. ^{13}C-NMR of compound **32**.

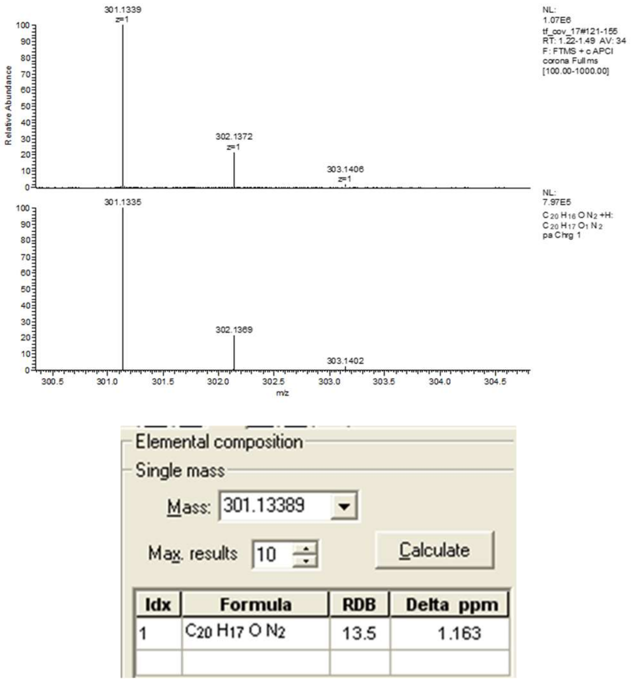

Figure S50. HRMS of compound **32**.

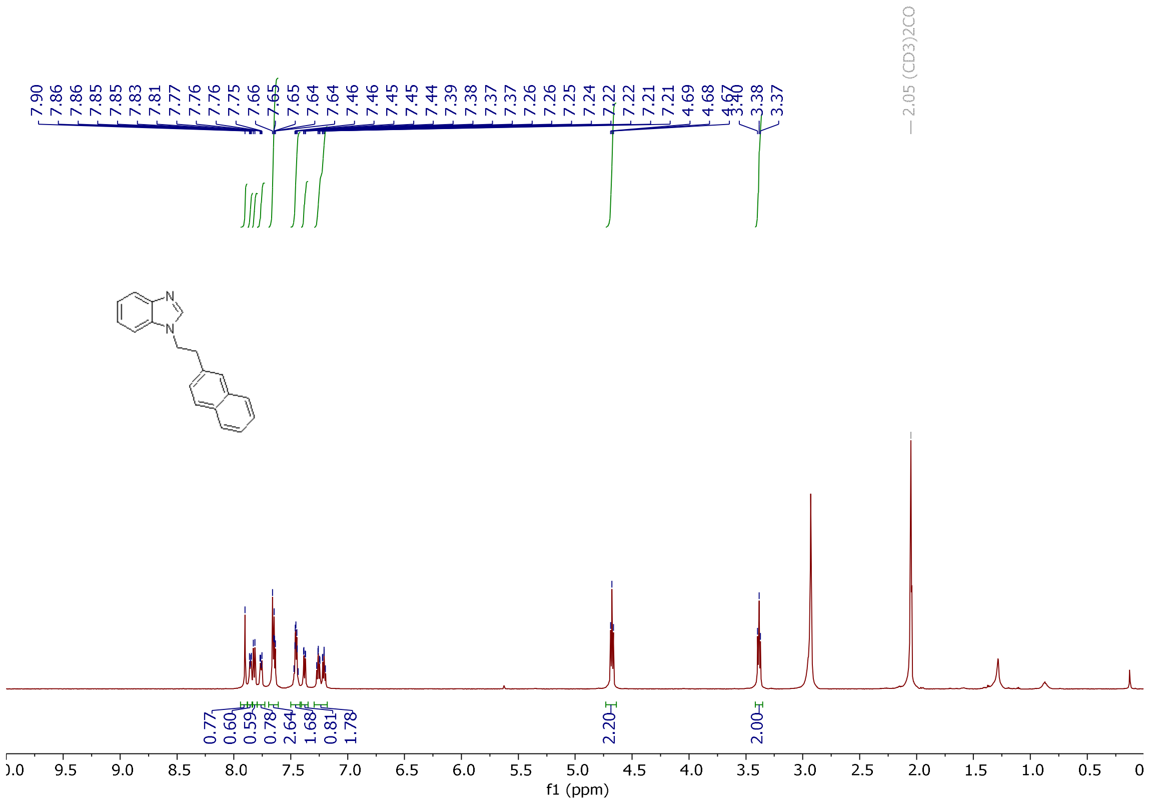

Figure S51. ^{1}H-NMR of compound **33**.

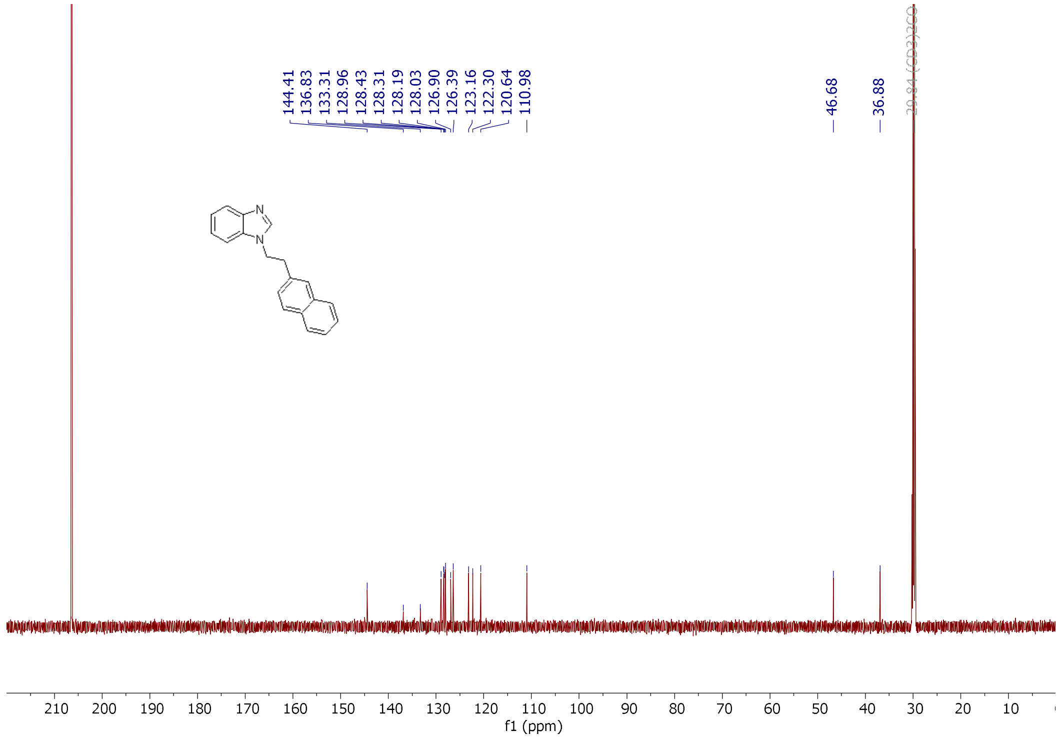

Figure S52. ^{13}C-NMR of compound **33**.

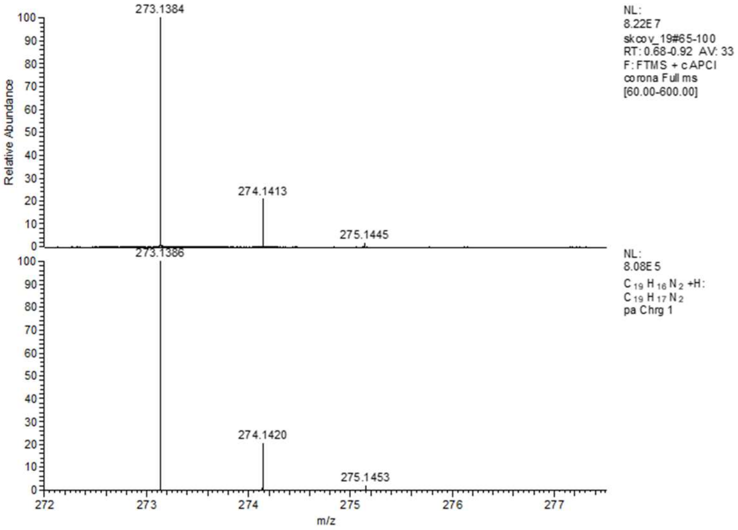

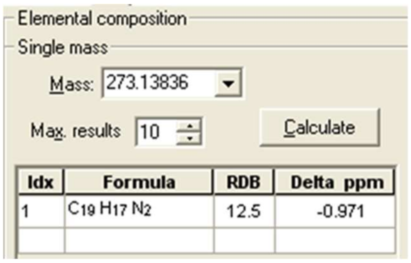
Elemental composition

Single mass

Mass: 273.13836

Max. results 10

Calculate

Idx	Formula	RDB	Delta ppm
1	$C_{19}H_{17}N_2$	12.5	-0.971

Figure S53. HRMS of compound **33**.

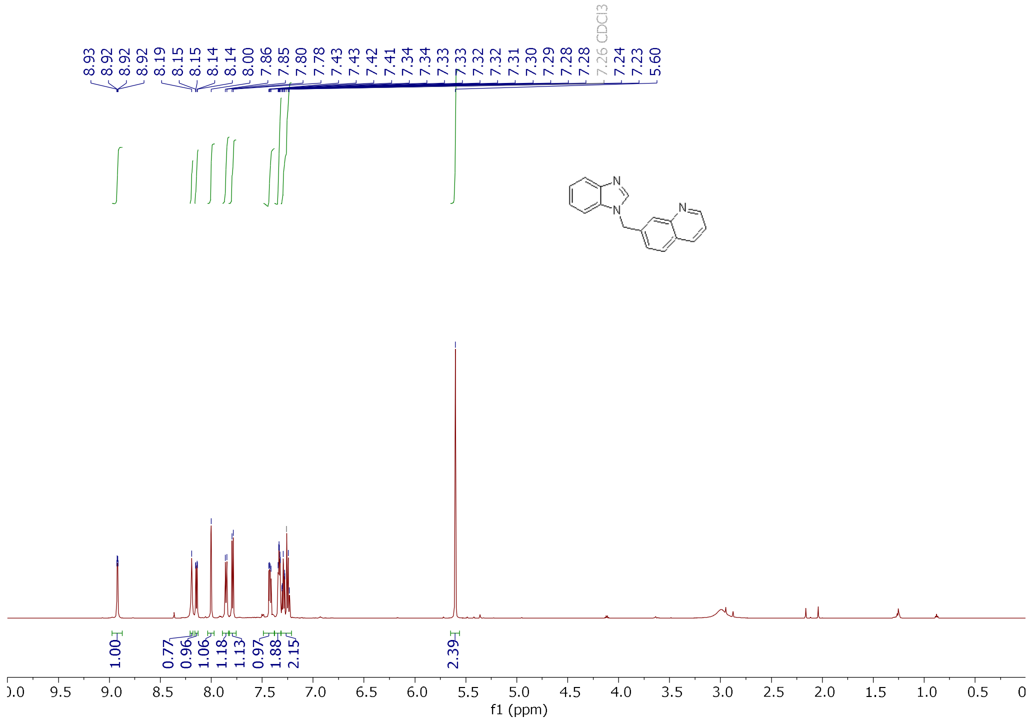

Figure S54. ^{1}H-NMR of compound **35**.

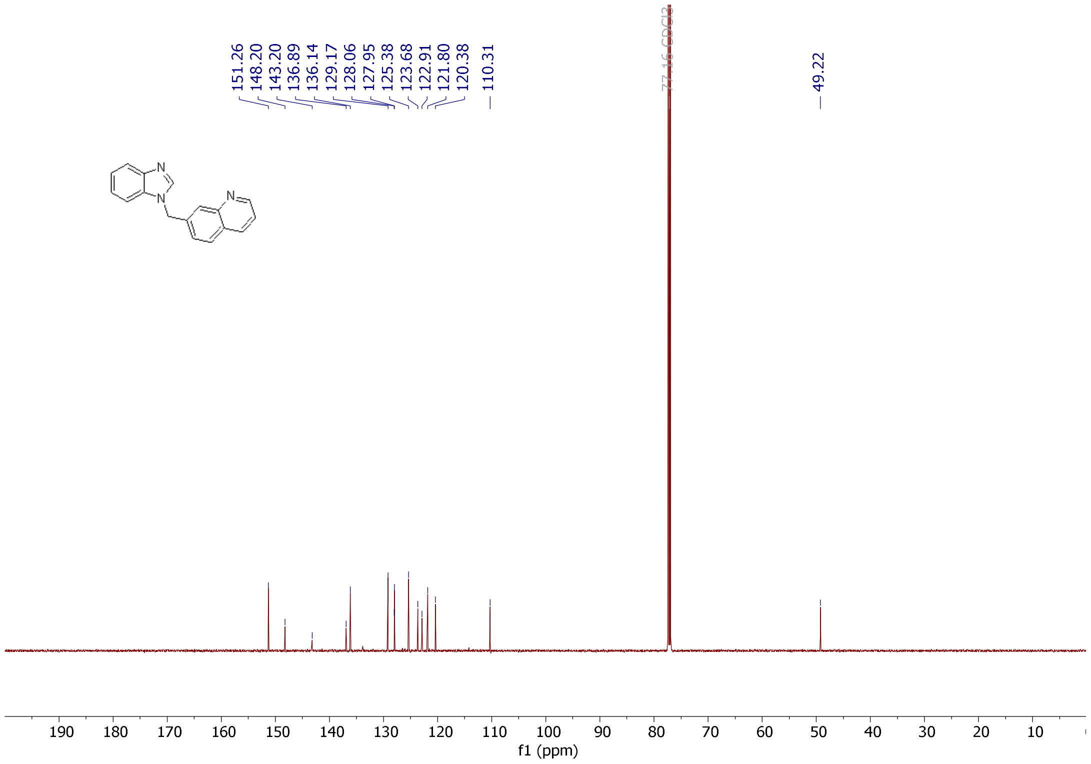

Figure S55. ^{13}C-NMR of compound **35**.

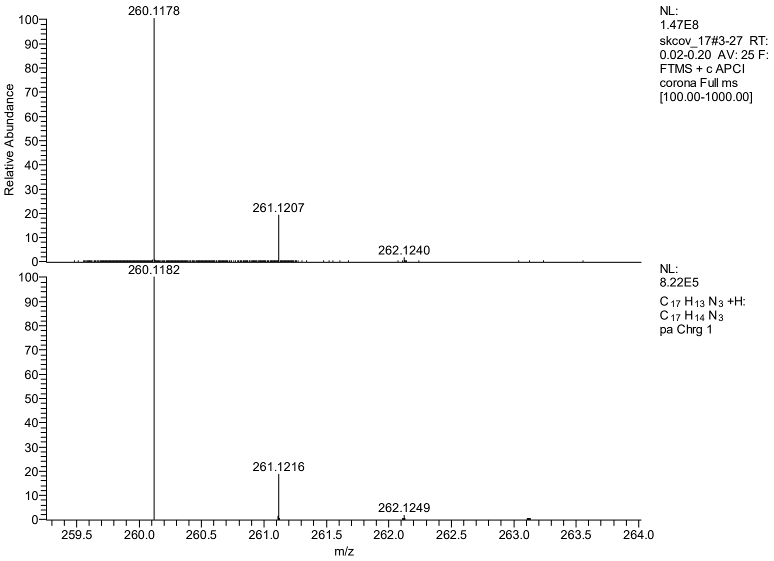

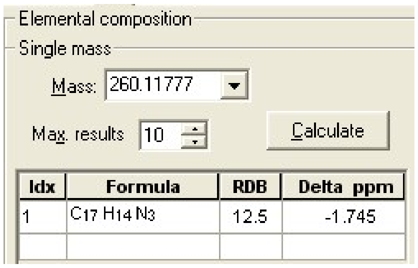

Idx	Formula	RDB	Delta ppm
1	$C_{17}H_{14}N_3$	12.5	-1.745

Figure S56. HRMS of compound **35**.

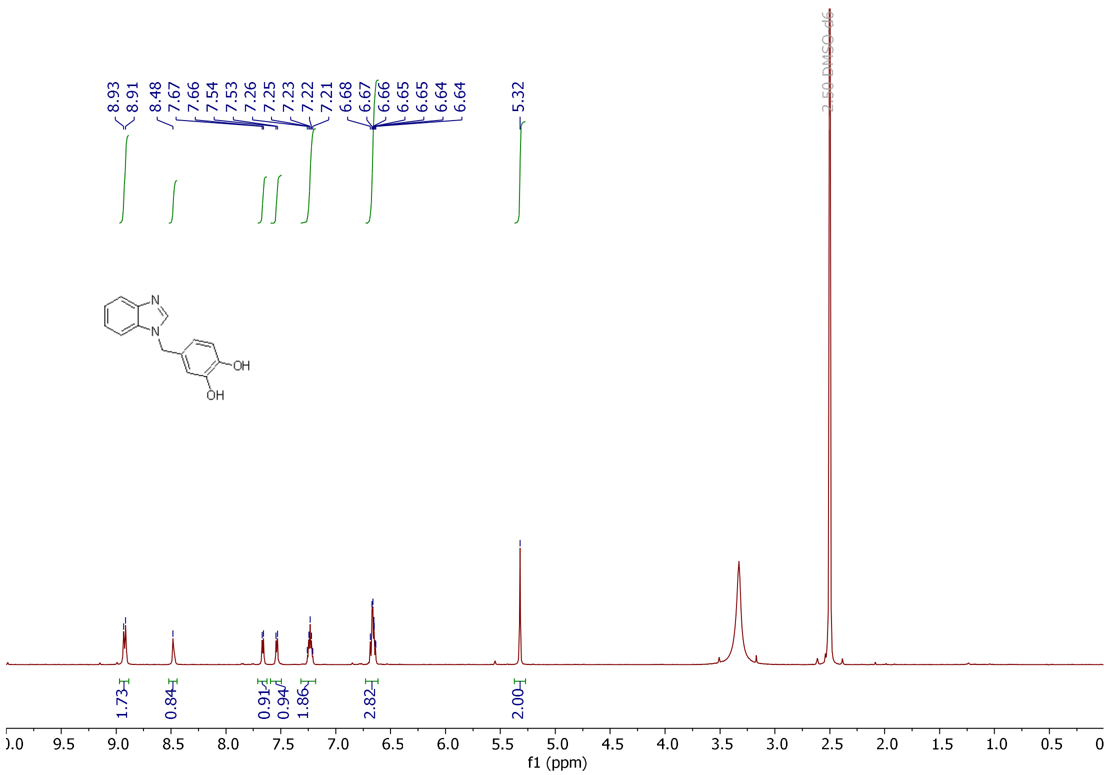

Figure S57. ^{1}H-NMR of compound **37**.

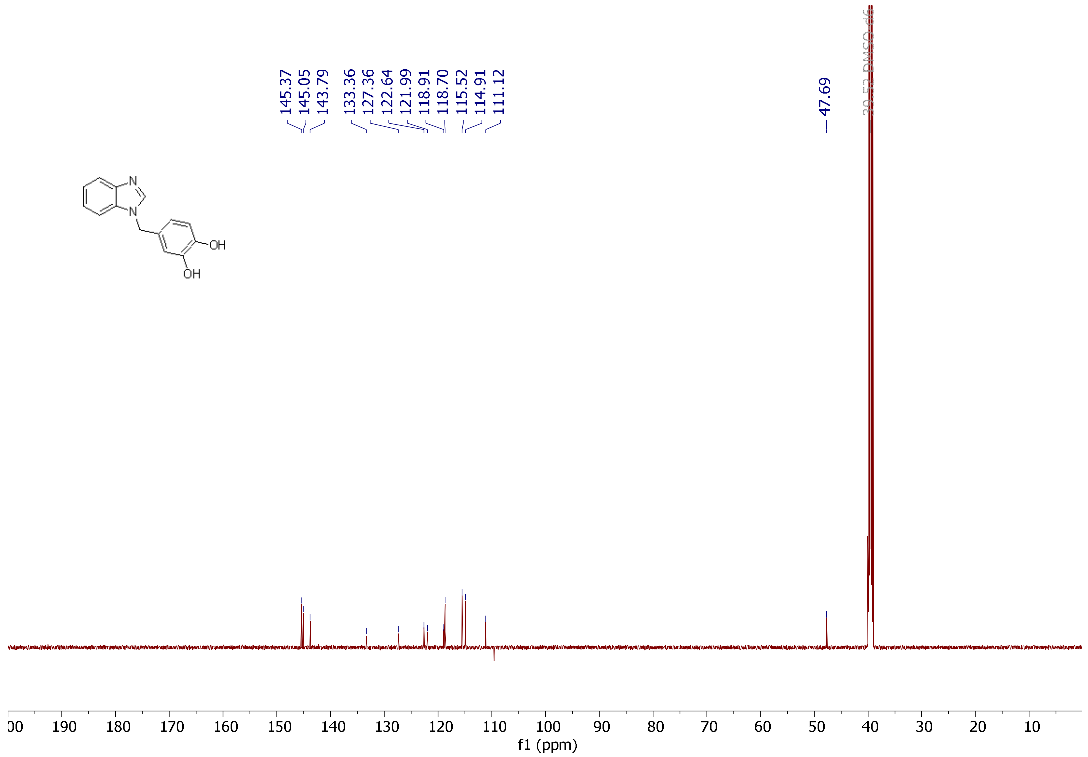

Figure S58. ^{13}C-NMR of compound **37**.

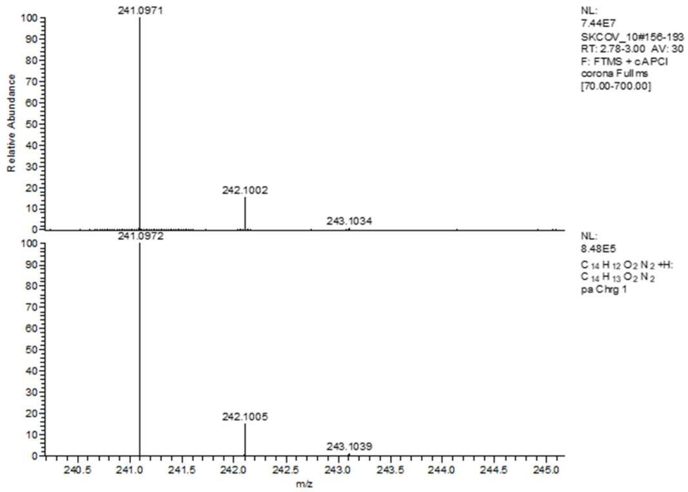

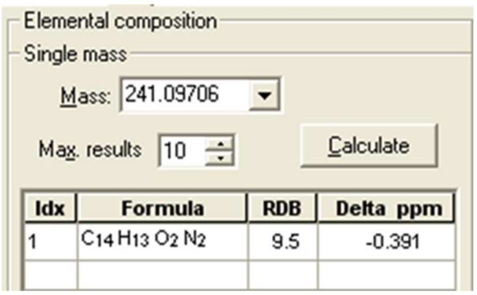
Elemental composition

Single mass

Mass: 241.09706

Max. results 10

Calculate

Idx	Formula	RDB	Delta ppm
1	$C_{14}H_{13}O_2N_2$	9.5	-0.391

Figure S59. HRMS of compound **37**.

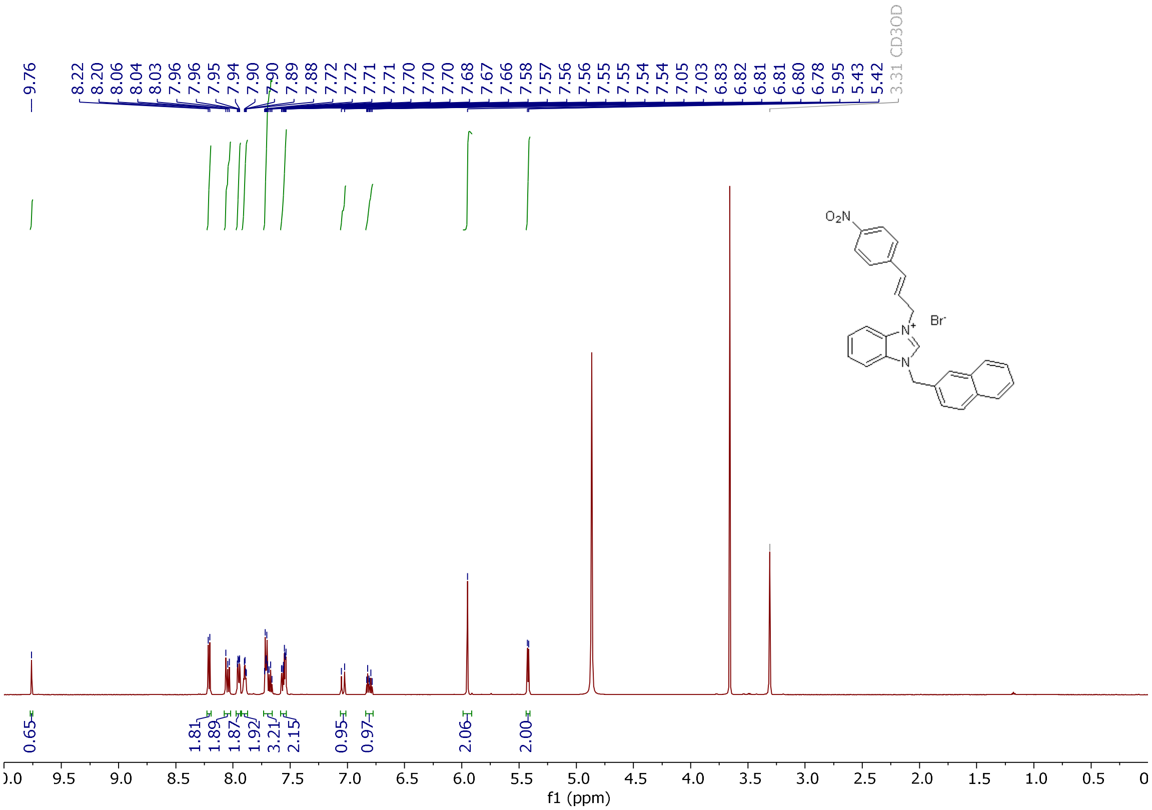

Figure S60. ^{1}H-NMR of compound **38**.

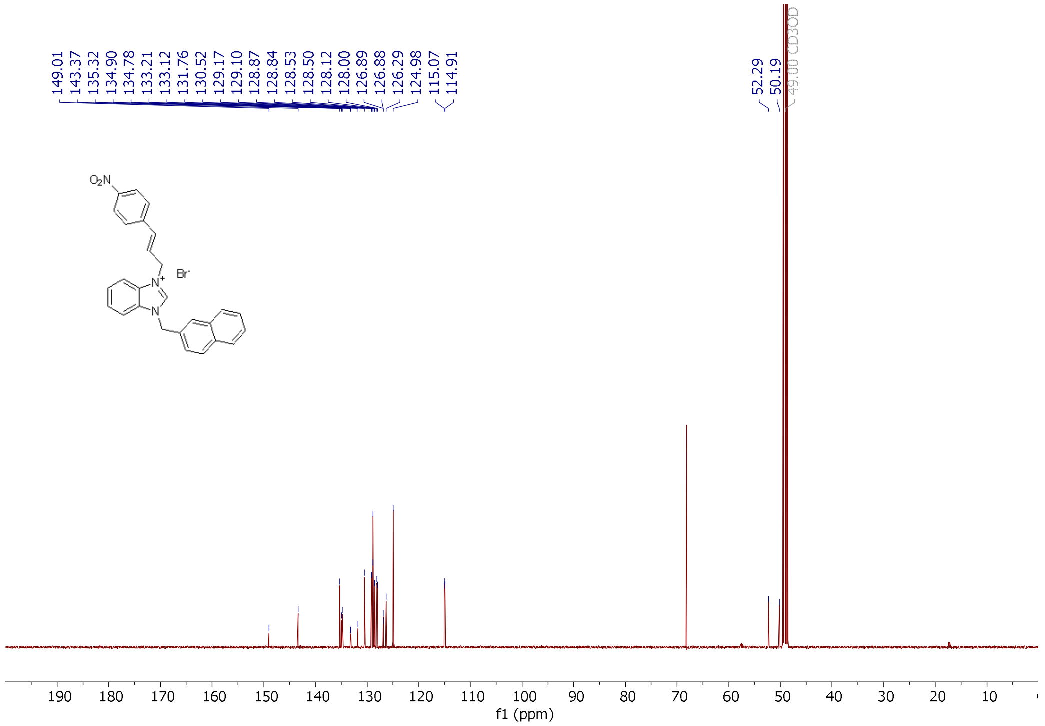

Figure S61. ^{13}C-NMR of compound **38**.

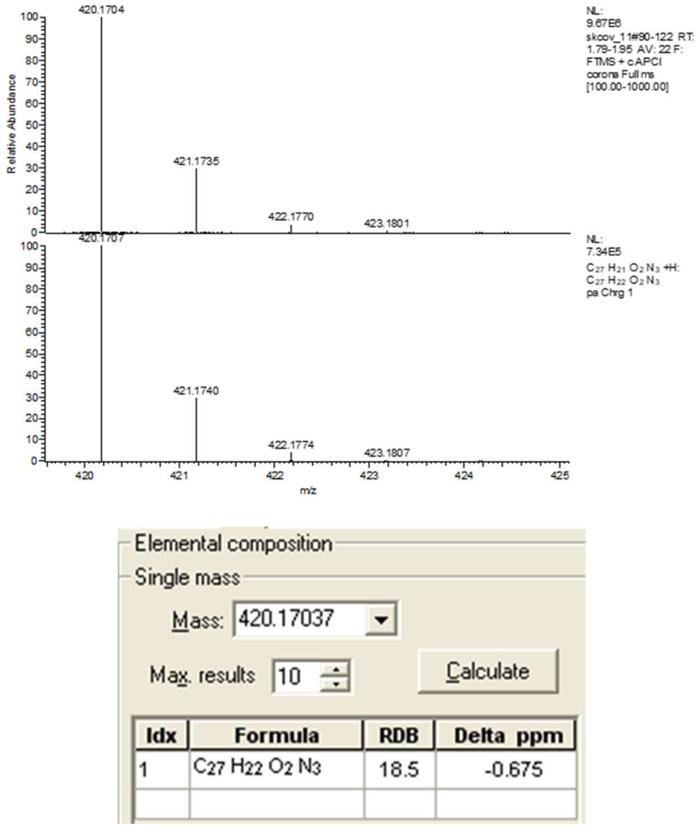

Figure S62. HRMS of compound **38**.

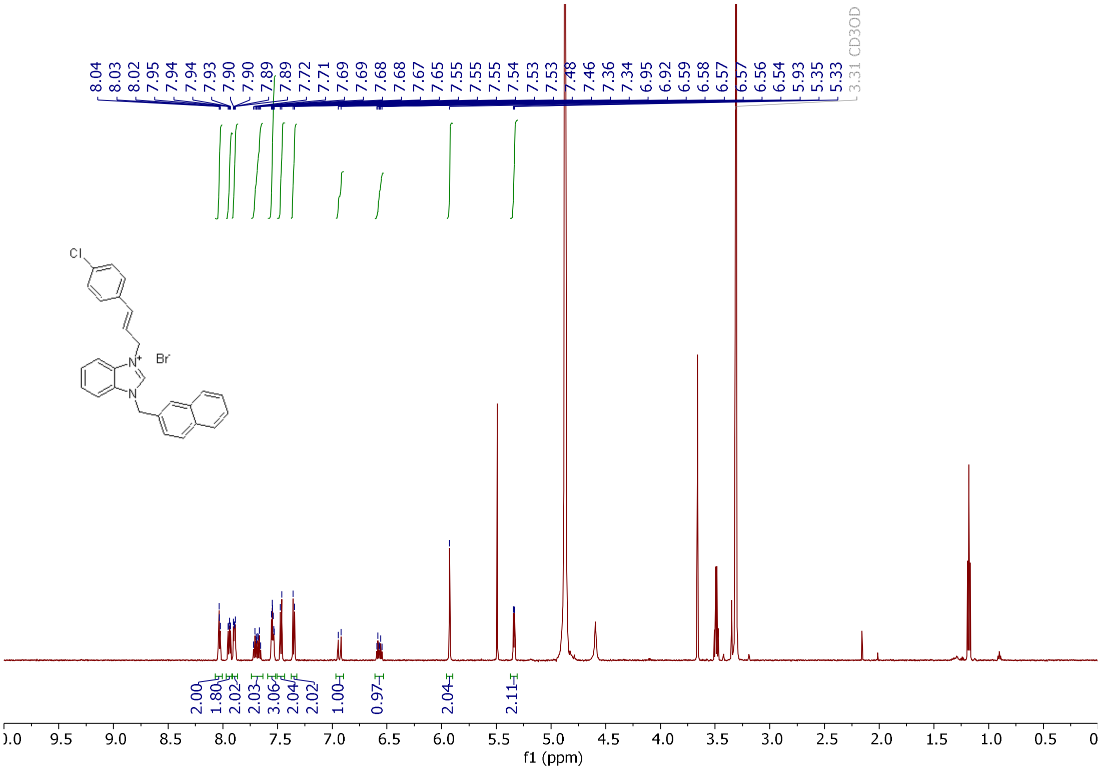

Figure S63. ^{1}H-NMR of compound **39**.

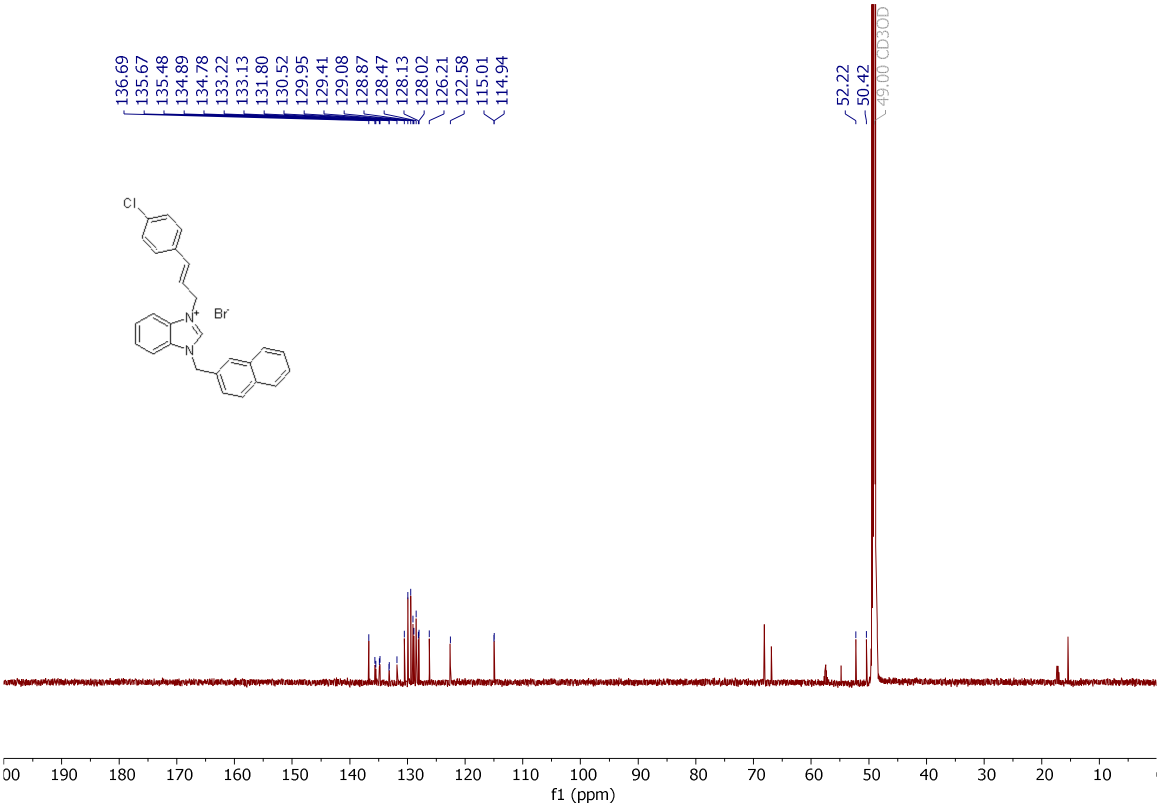

Figure S64. ^{13}C-NMR of compound **39**.

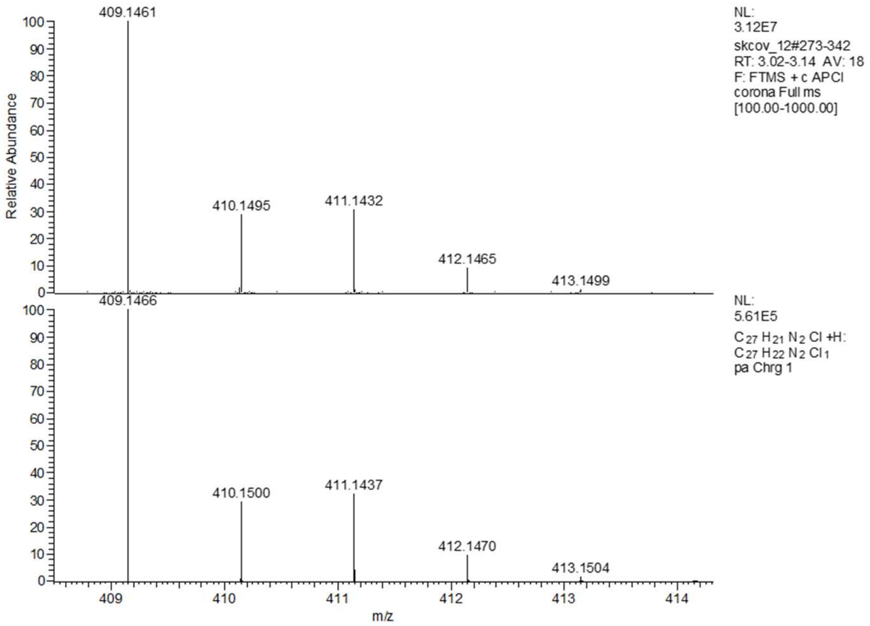

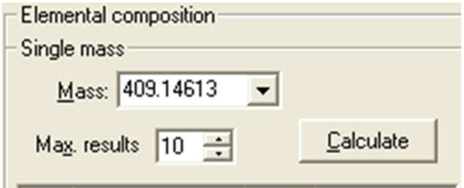

Idx	Formula	RDB	Delta ppm
1	$C_{27}H_{22}N_2Cl$	17.5	-1.156

Figure S65. HRMS of compound **39**.

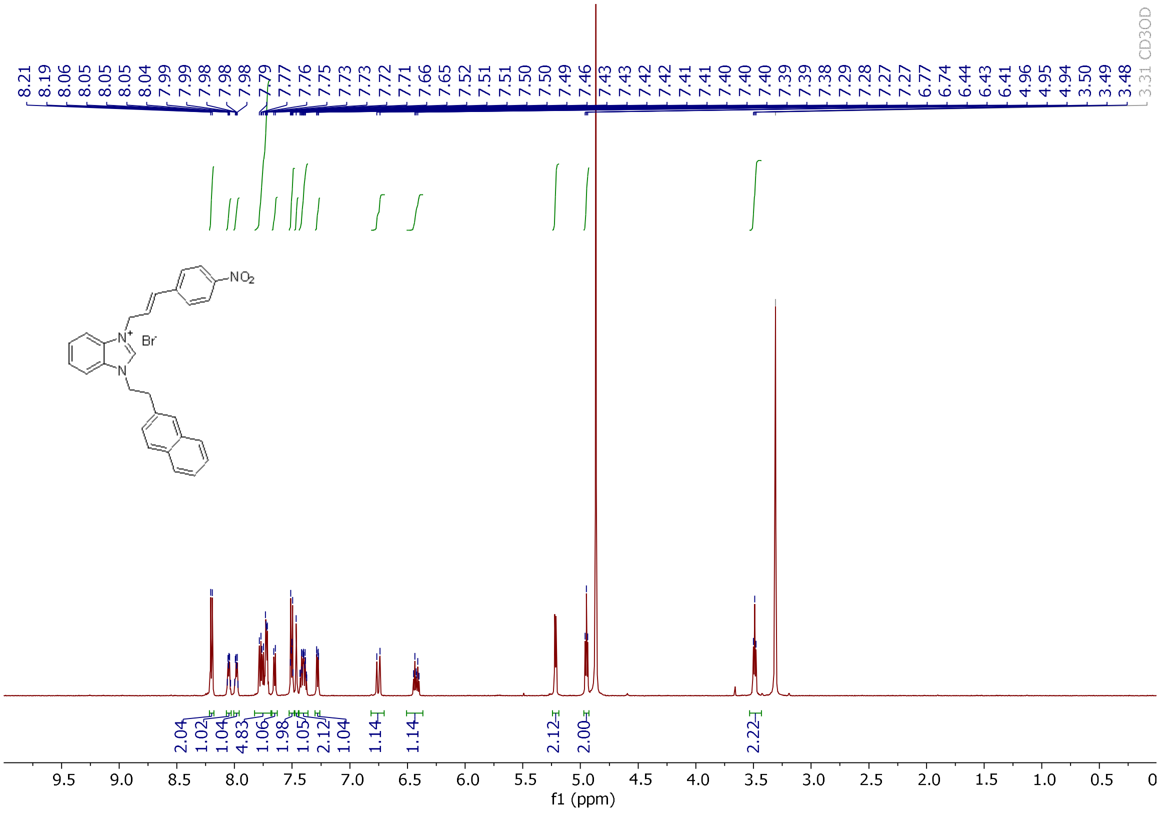

Figure S66. ^{1}H-NMR of compound **40**.

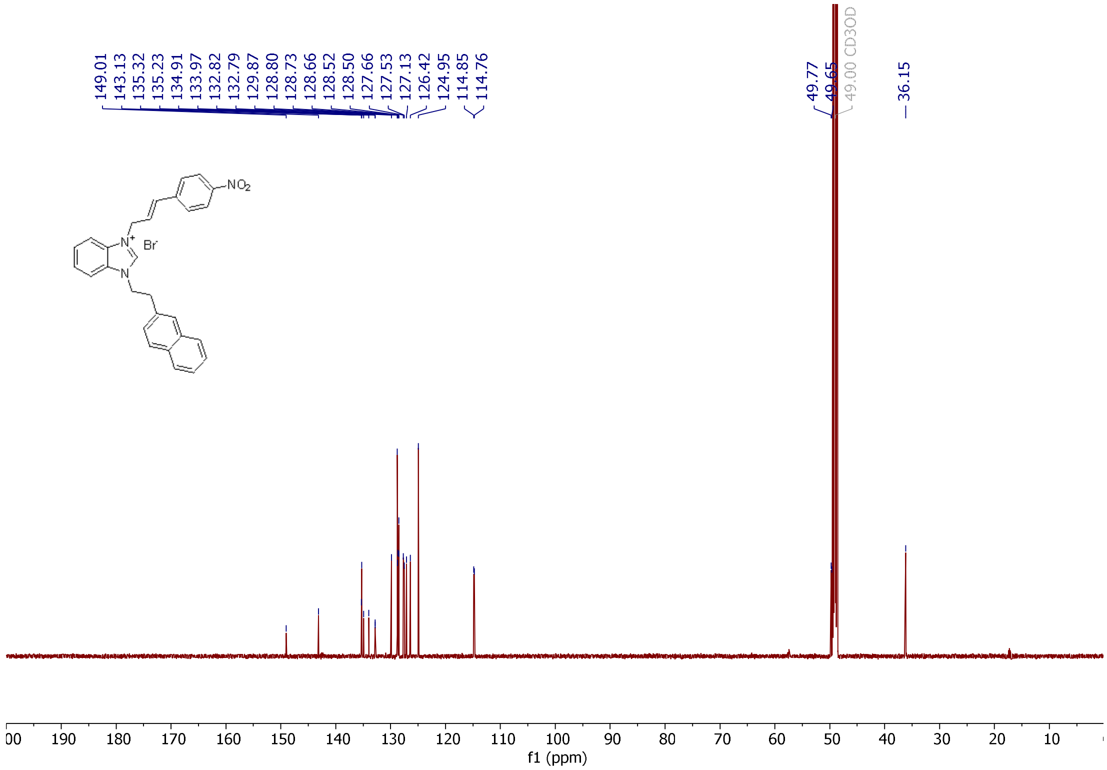

Figure S67. ^{13}C-NMR of compound **40**.

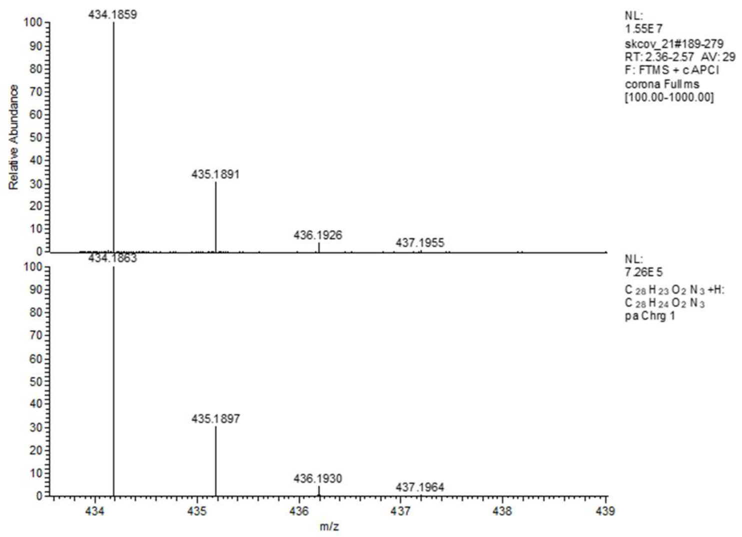

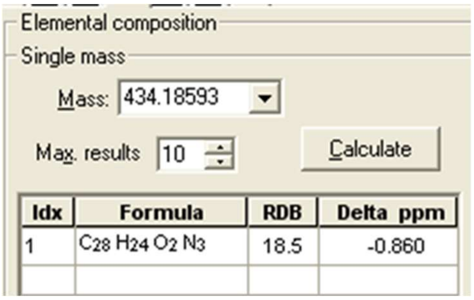
Elemental composition

Single mass

Mass: 434.18593

Max. results 10

Calculate

Idx	Formula	RDB	Delta ppm
1	$C_{28}H_{24}O_2N_3$	18.5	-0.860

Figure S68. HRMS of compound **40**.

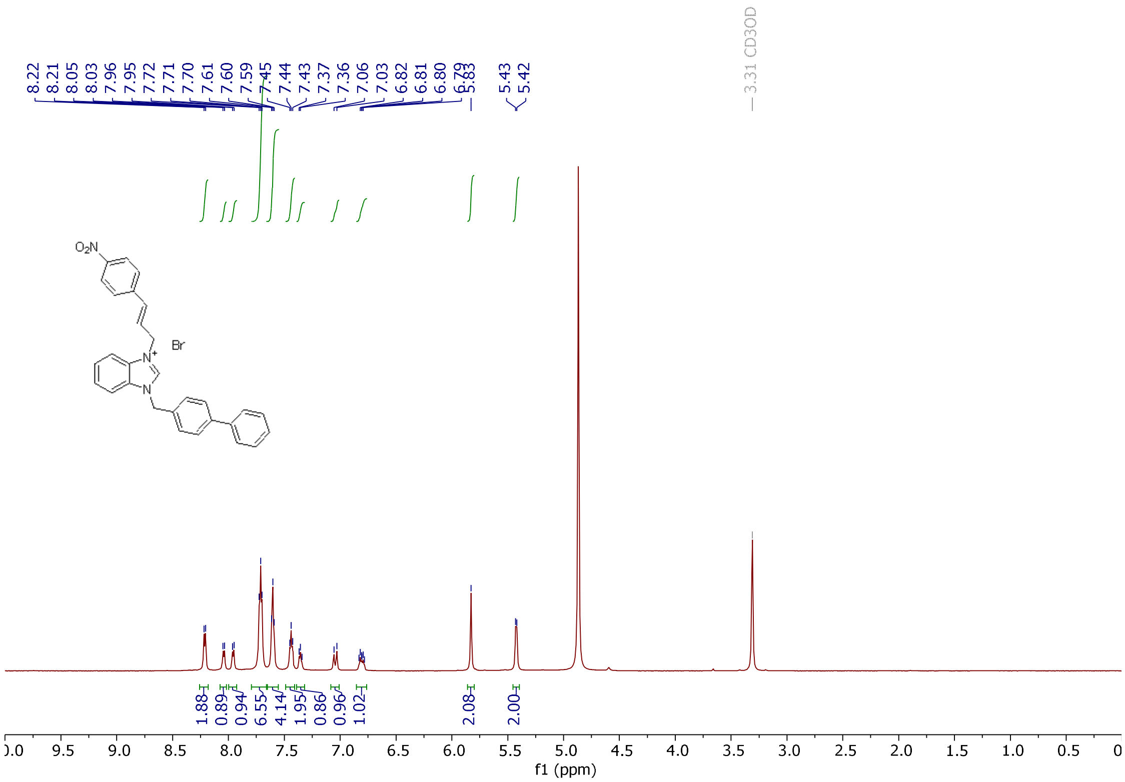

Figure S69. ^{1}H-NMR of compound **41**.

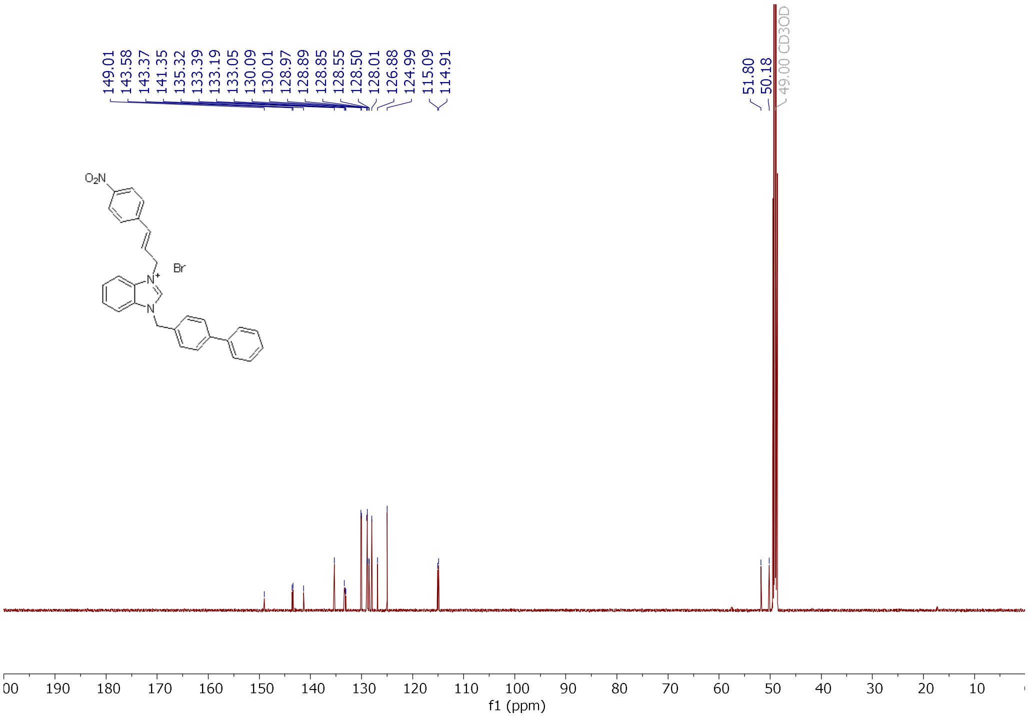

Figure S70. ^{13}C-NMR of compound **41**.

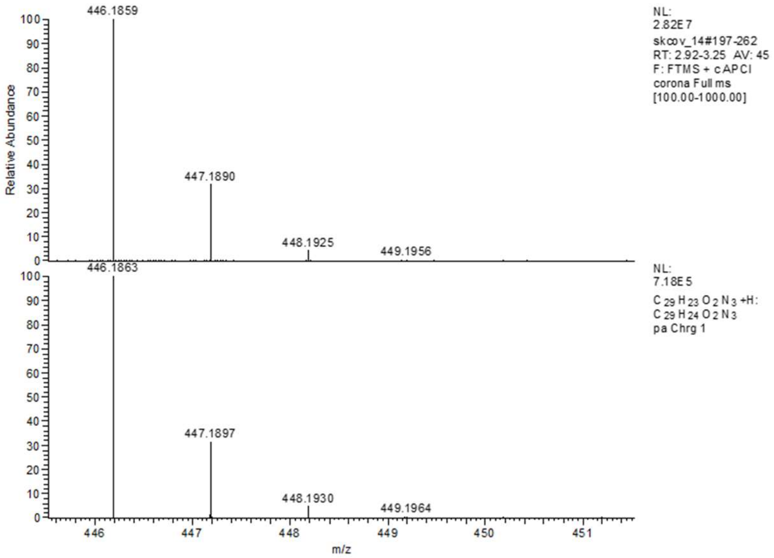

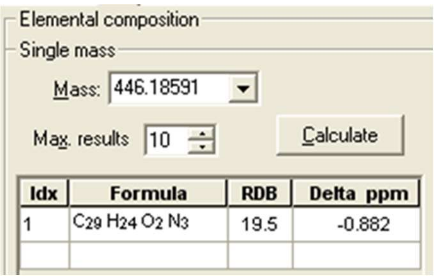

Idx	Formula	RDB	Delta ppm
1	$C_{29}H_{24}O_2N_3$	19.5	-0.882

Figure S71. HRMS of compound **41**.

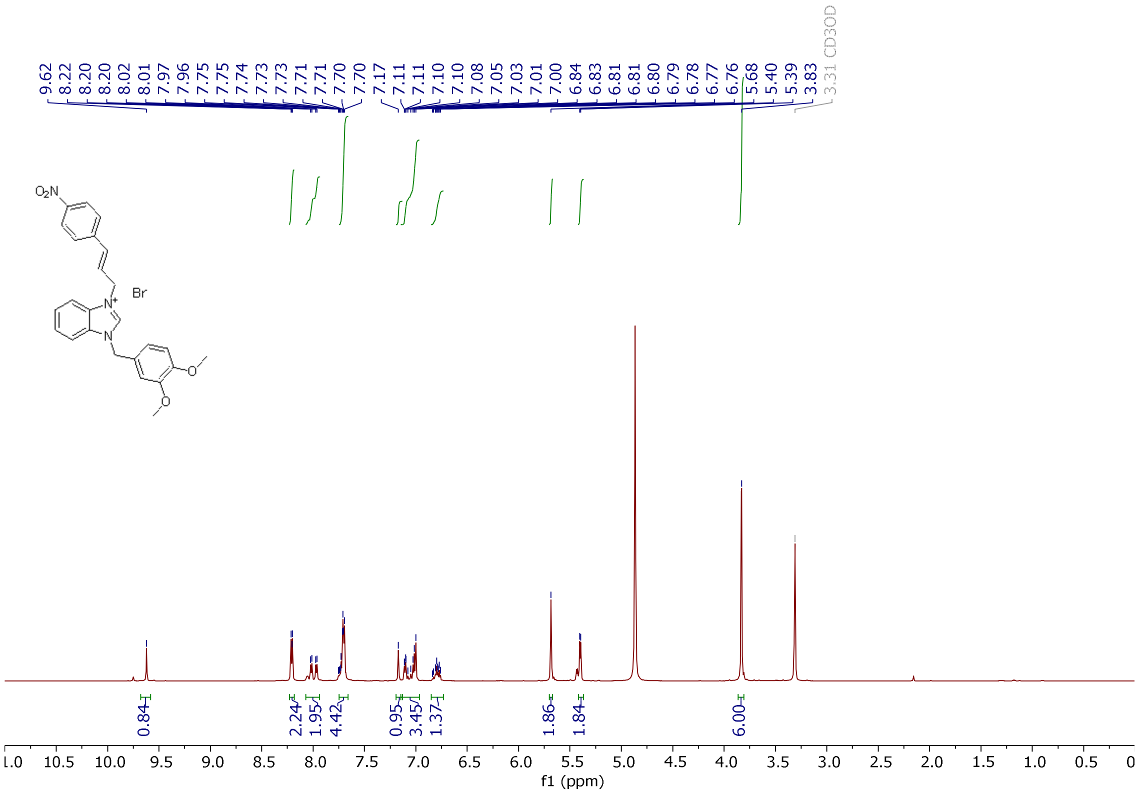

Figure S72. ^{1}H-NMR of compound **42**.

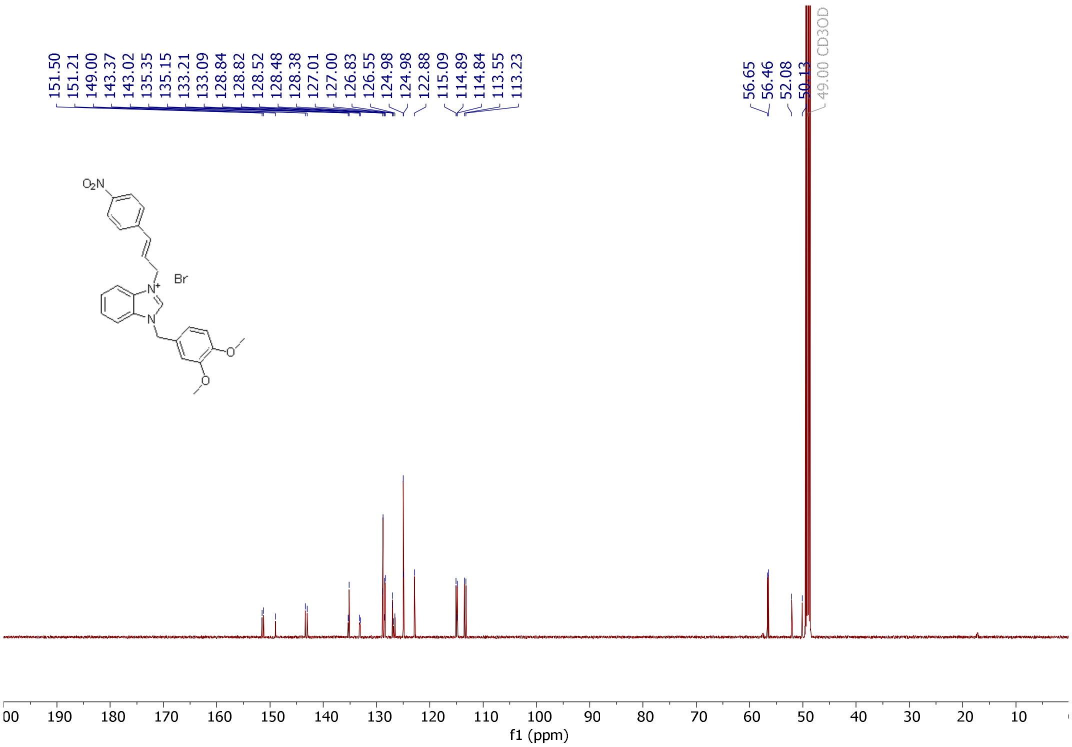

Figure S73. ^{13}C-NMR of compound **42**.

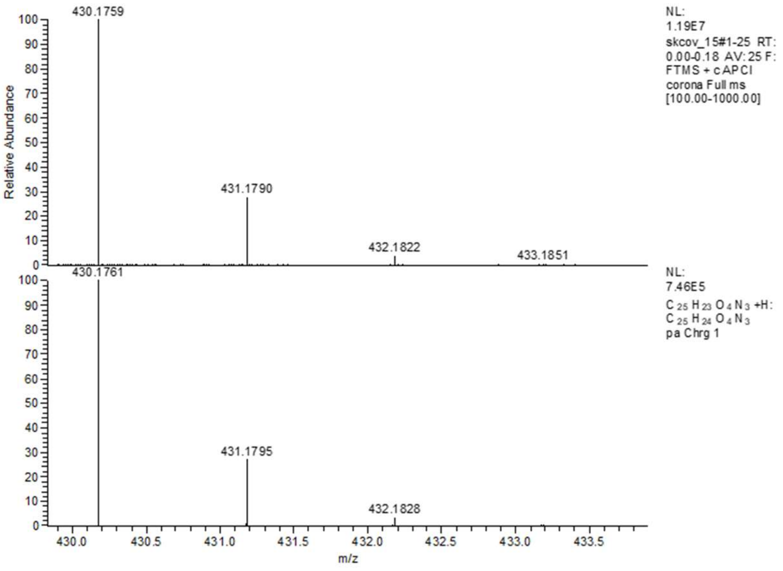

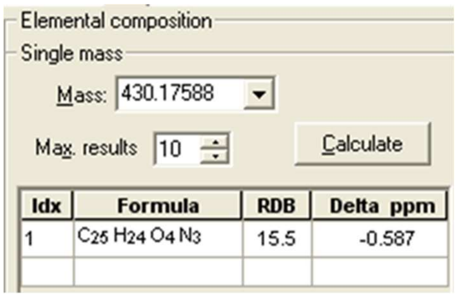

Idx	Formula	RDB	Delta ppm
1	$C_{25}H_{24}O_4N_3$	15.5	-0.587

Figure S74. HRMS of compound **42**.

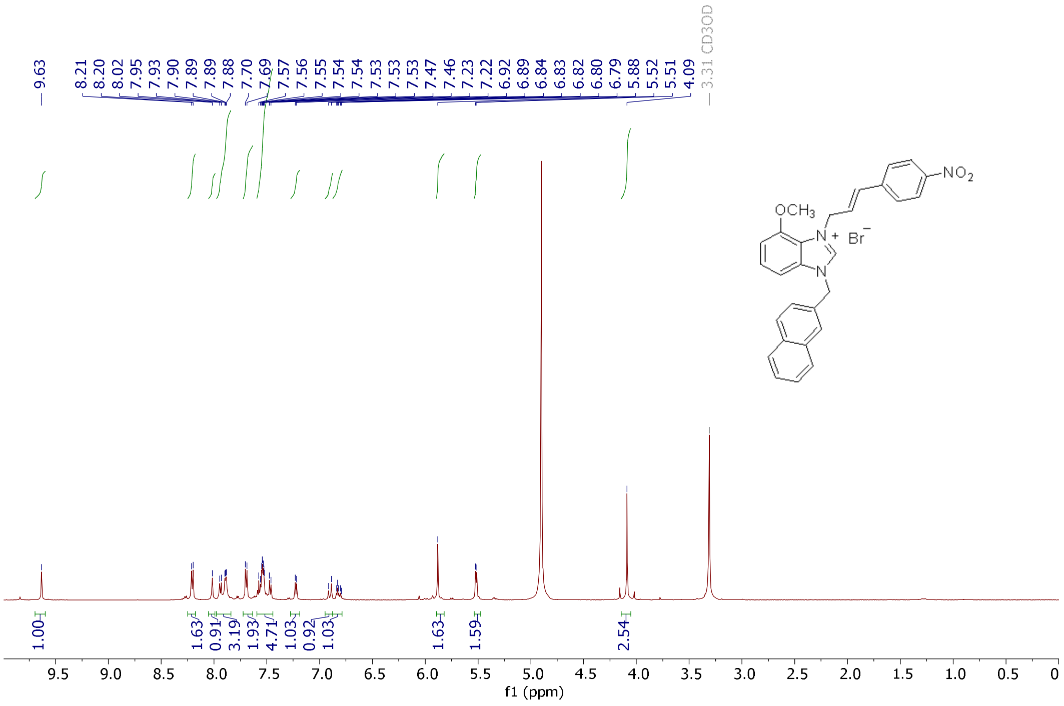

Figure S75. ^{1}H-NMR of compound **43**.

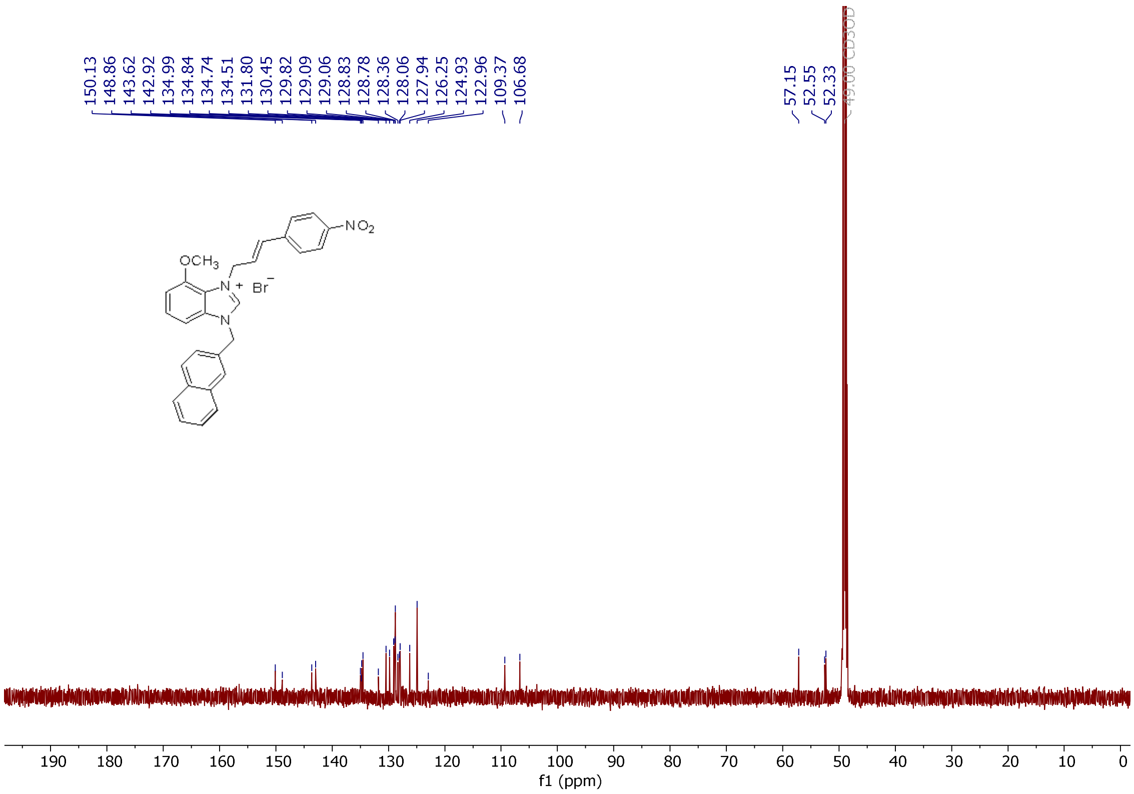

Figure S76. ^{13}C-NMR of compound **43**.

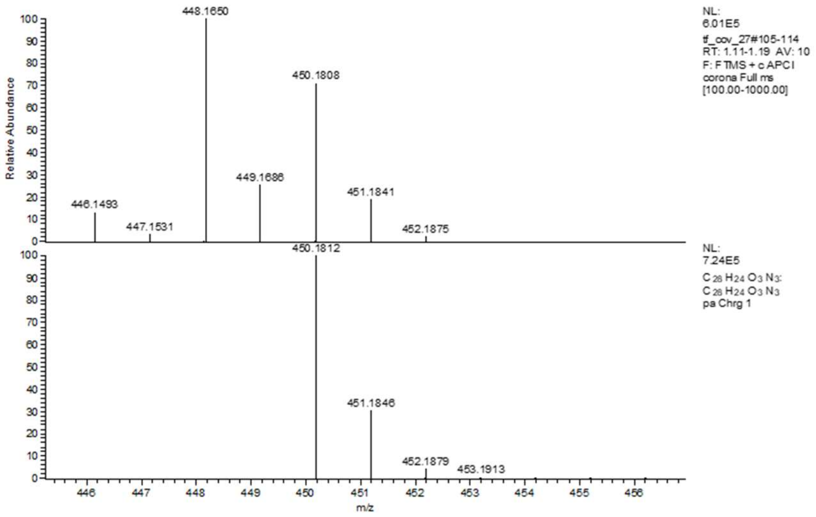

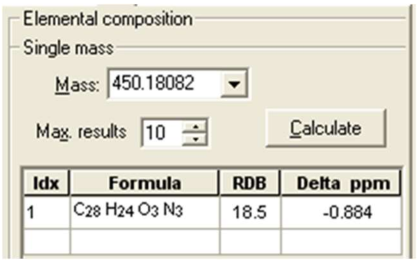
Elemental composition

Single mass

Mass: 450.18082

Max. results 10 Calculate

Idx	Formula	RDB	Delta ppm
1	$C_{28}H_{24}O_3N_3$	18.5	-0.884

Figure S77. HRMS of compound **43**.

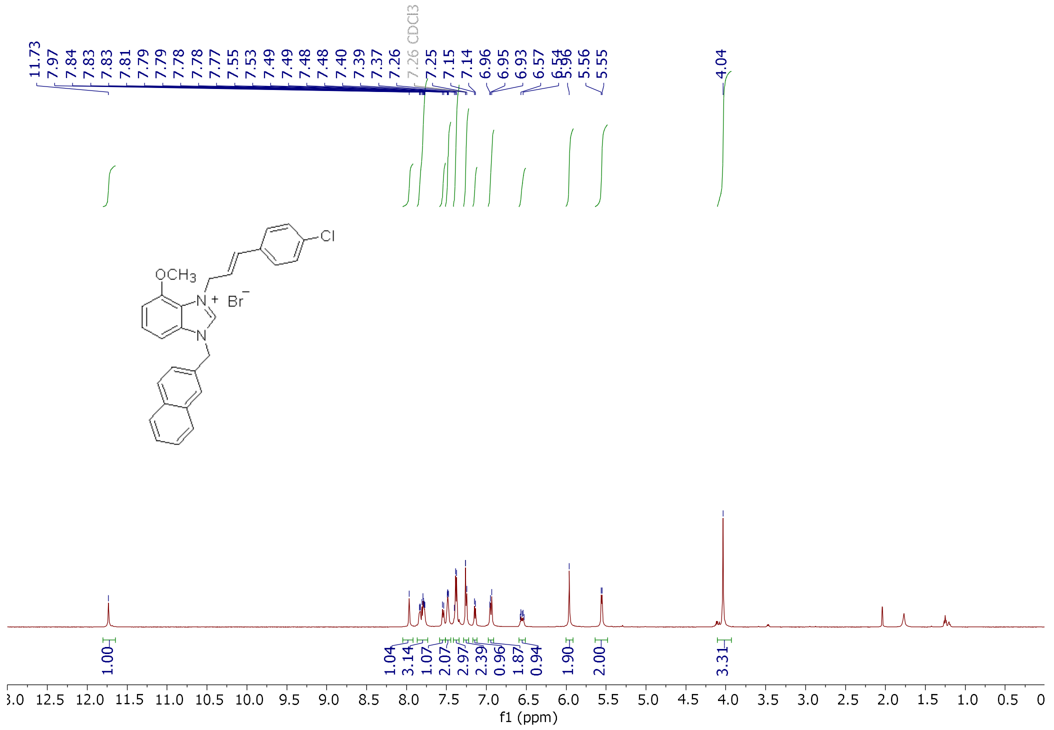

Figure S78. ^{1}H-NMR of compound **44**.

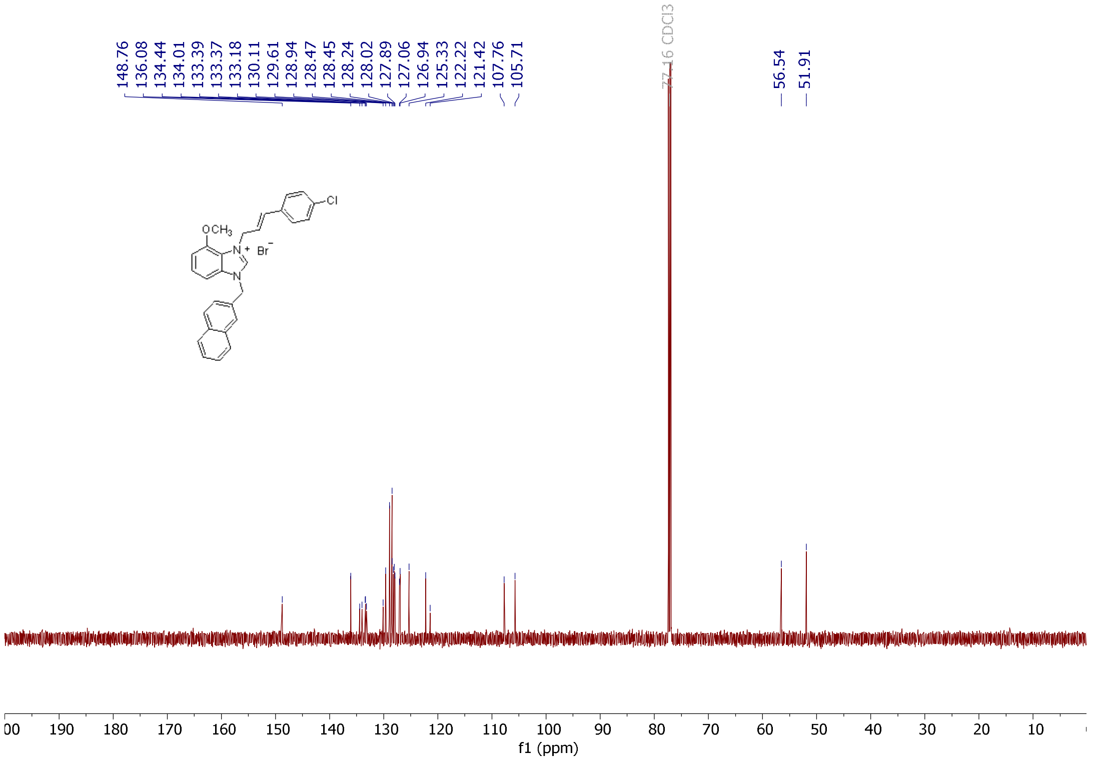

Figure S79. ^{13}C-NMR of compound **44**.

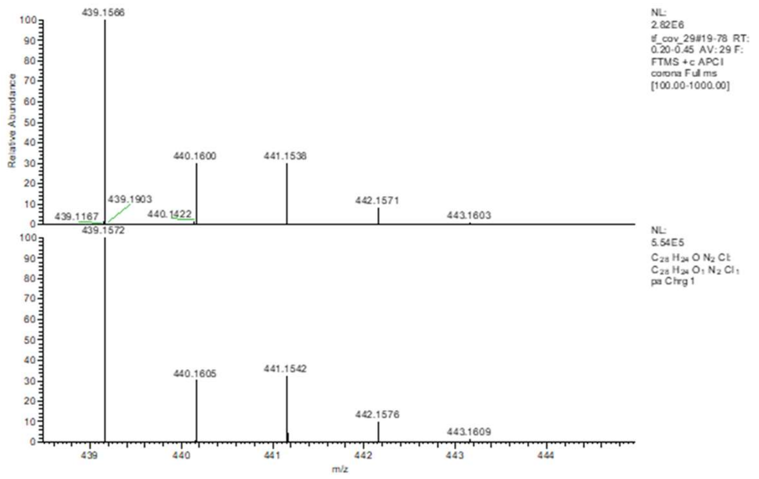

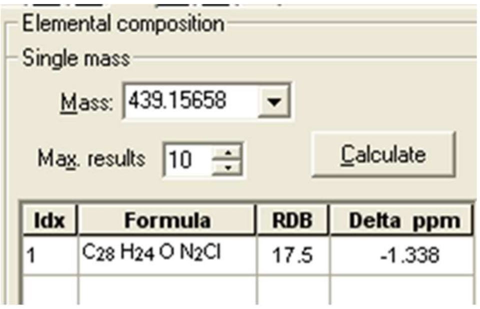

Elemental composition

Single mass

Mass: 439.15658

Max. results 10

Calculate

Idx	Formula	RDB	Delta ppm
1	$C_{28}H_{24}ON_2Cl$	17.5	-1.338

Figure S80. HRMS of compound **44**.

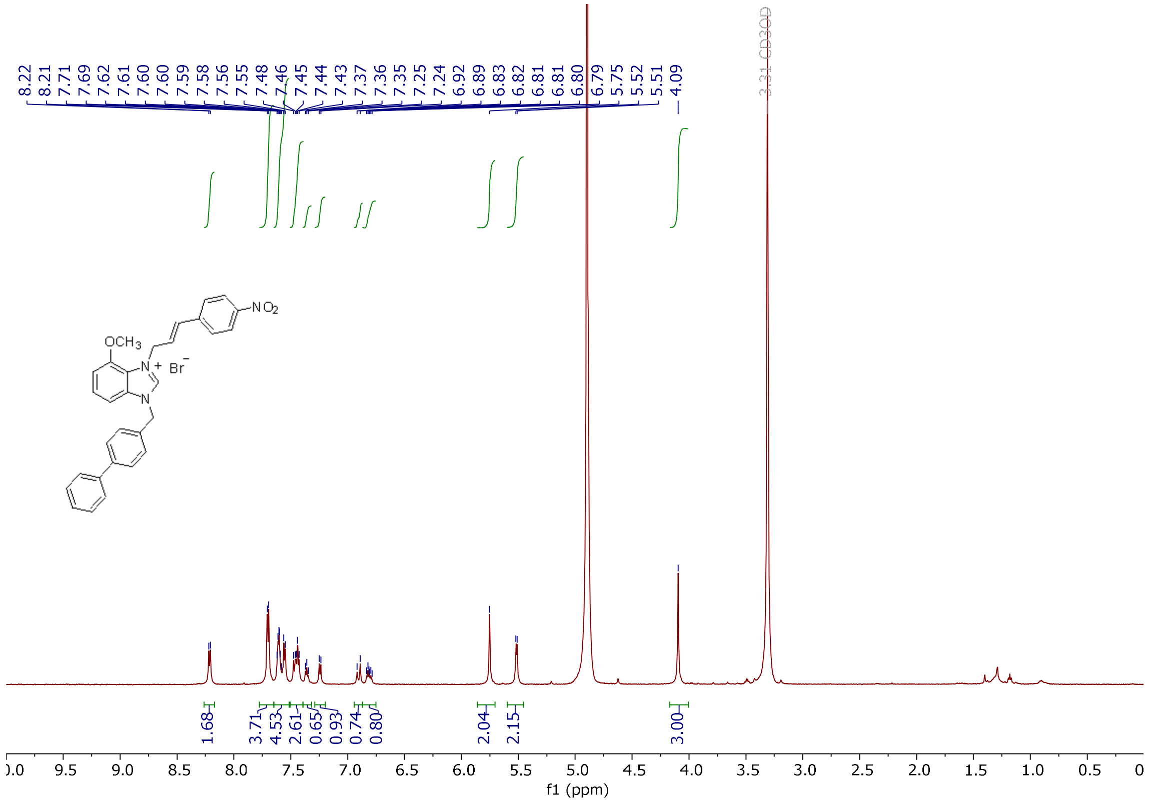

Figure S81. ^{1}H-NMR of compound **45**.

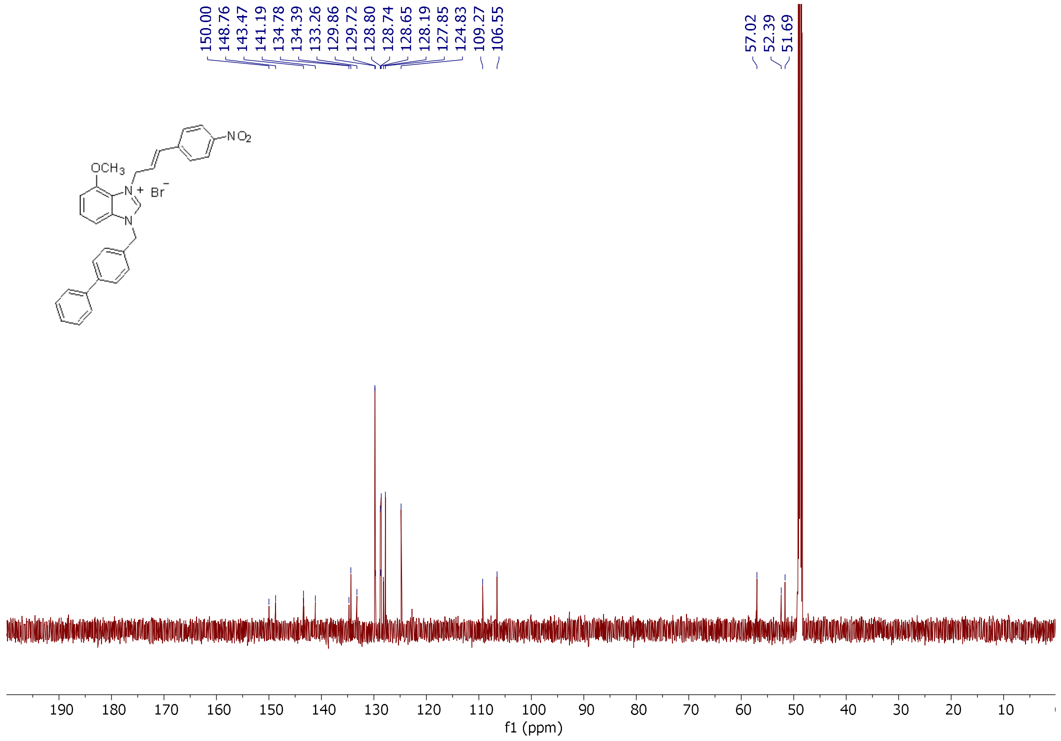

Figure S82. ^{13}C-NMR of compound **45**.

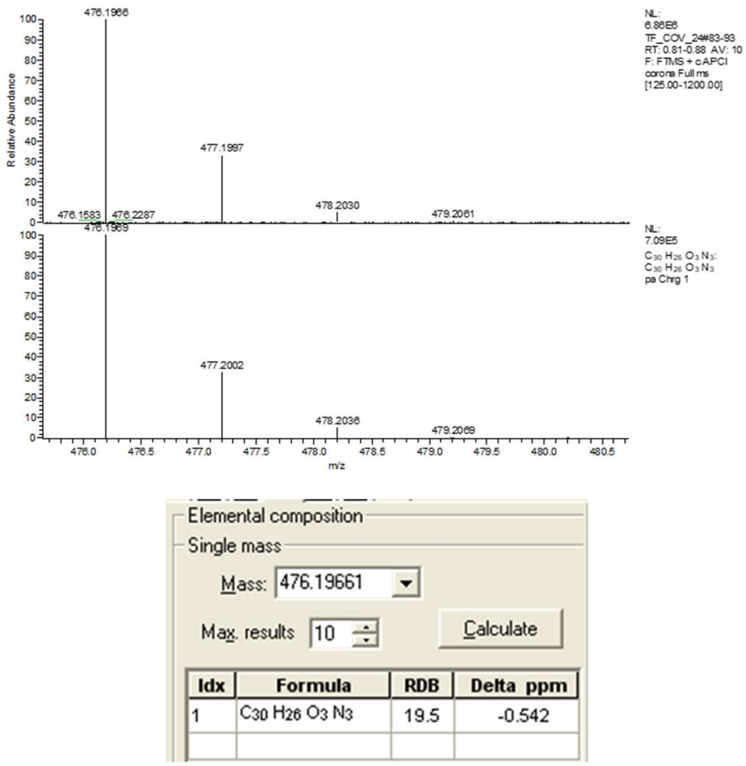

Figure S83. HRMS of compound **45**.

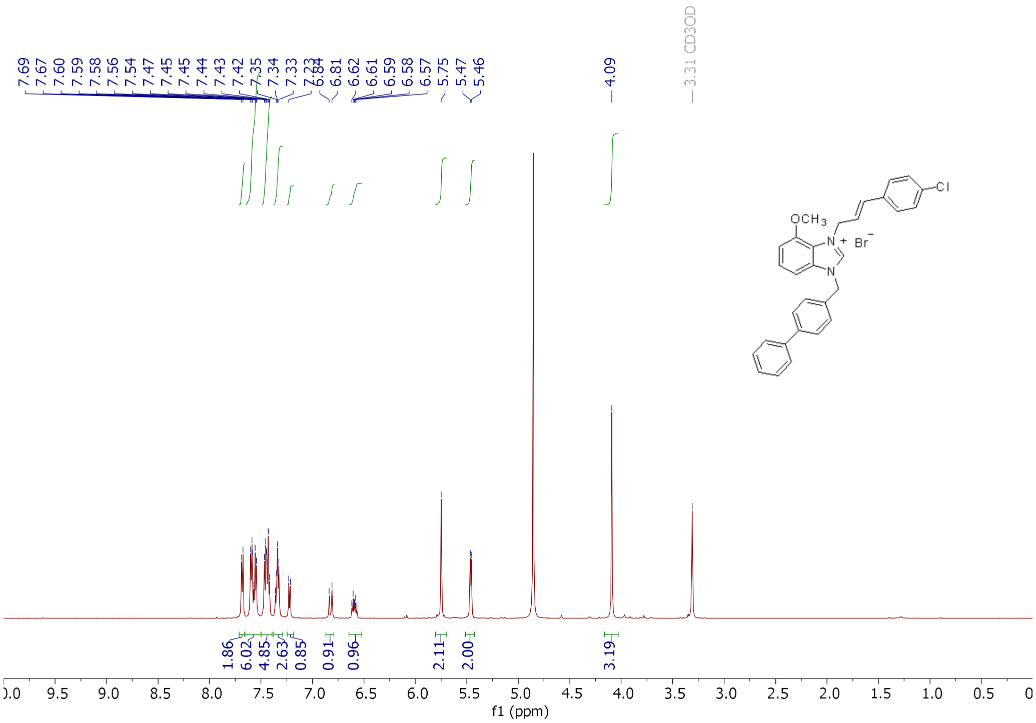

Figure S84. ^{1}H-NMR of compound **46**.

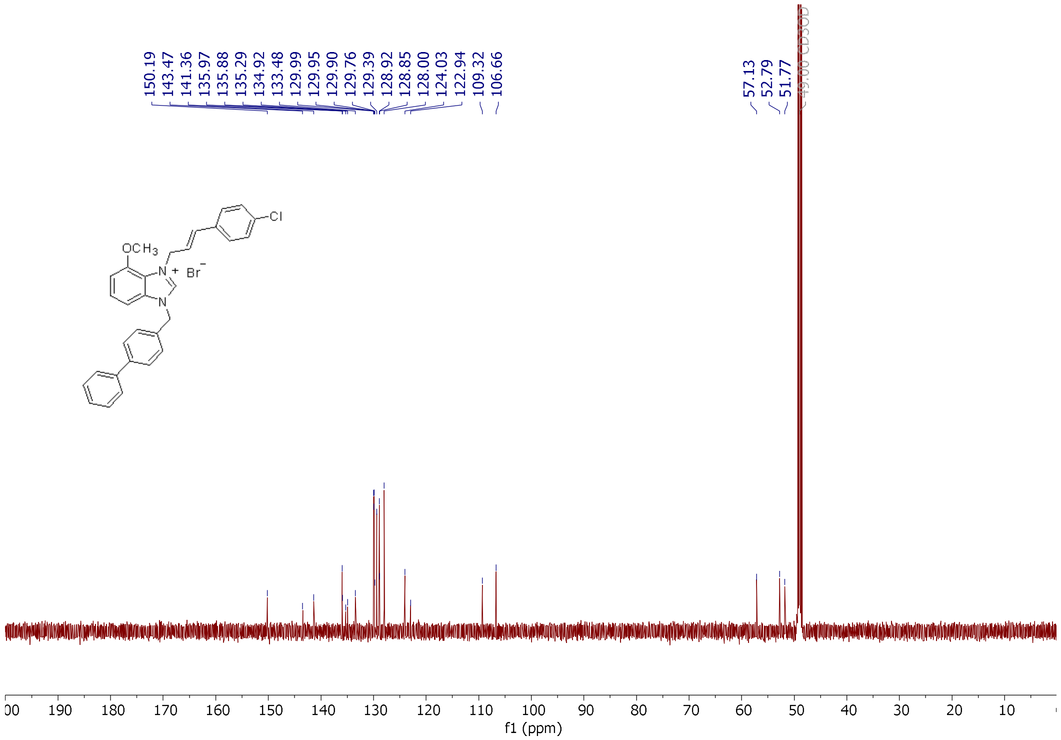

Figure S85. ^{13}C-NMR of compound **46**.

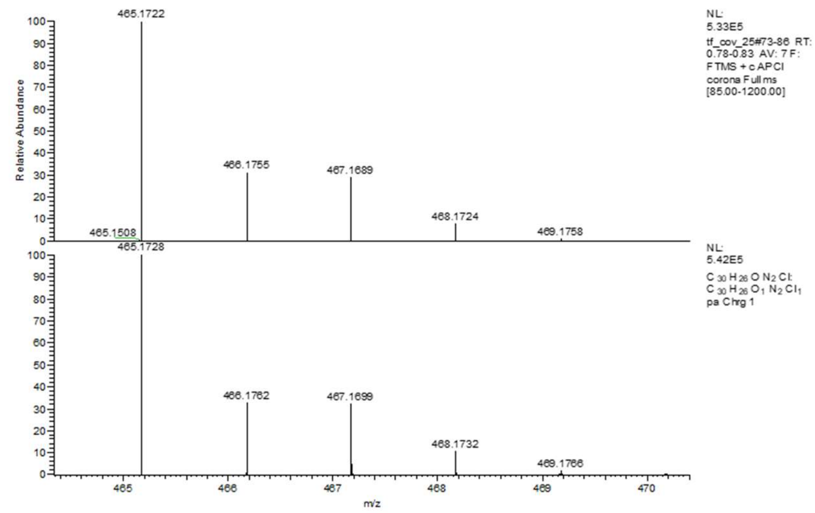

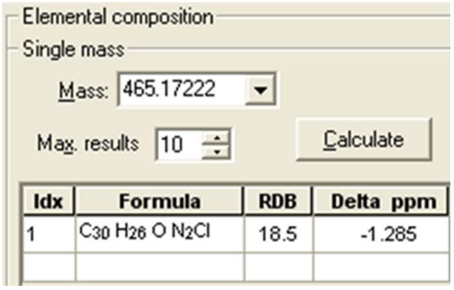

Figure S86. HRMS of compound **46**.

HPLC chromatograms of final compounds
Compound 3

Page 1 of 1

Area % Report

Data File: C:\ChromQuest\Enterprise\Projects\Default\Data\Covid Project\CKP37_.dat
Method:
C:\ChromQuest\Enterprise\Projects\Default\Method\Covid\H2O_0.1%TFA_ACN_20_80_Isocratic.met
Acquired: 10/30/2023 4:00:02 PM
Printed: 10/31/2023 5:43:41 PM

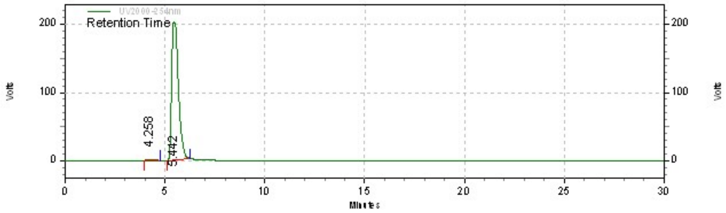

UV2000-254nm Results (System (10/31/2023 5:43:37 PM) (Reprocessed))

Retention Time	Area	Area %	Height	Height %
4.258	37637	0.78	1700	0.83
5.442	4759276	99.22	202418	99.17
Totals	4796913	100.00	204118	100.00

Figure S87. HPLC of compound **3**.

Compound 4

Page 1 of 1

Area % Report

Data File: C:\ChromQuest\Enterprise\Projects\Default\Data\Covid Project\CKP38.dat
Method:
C:\ChromQuest\Enterprise\Projects\Default\Method\Covid\H2O_0.1%TFA_ACN_20_80_Isocratic.met
Acquired: 10/30/2023 2:38:57 PM
Printed: 10/31/2023 5:46:29 PM

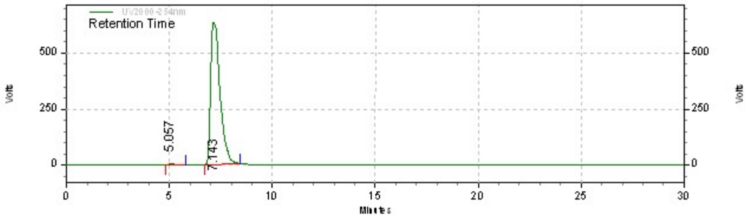

UV2000-254nm Results (System (10/31/2023 5:46:26 PM) (Reprocessed))

Retention Time	Area	Area %	Height	Height %
5.057	108028	0.54	4733	0.74
7.143	20082872	99.46	634225	99.26
Totals	20190900	100.00	638958	100.00

Figure S88. HPLC of compound **4**.

Compound 5

Page 1 of 1

Area % Report

Data File: C:\ChromQuest\Enterprise\Projects\Default\Data\Covid Project\CKP39.dat
Method:
C:\ChromQuest\Enterprise\Projects\Default\Method\Covid\H2O_0.1%TFA_ACN_20_80_Isocratic.met
Acquired: 10/30/2023 3:12:36 PM
Printed: 10/31/2023 5:48:21 PM

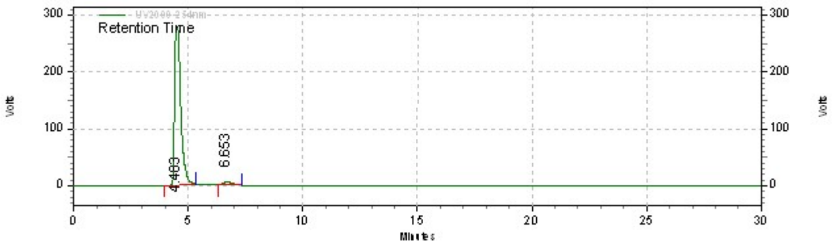

UV2000-254nm Results (System (10/31/2023 5:48:19 PM) (Reprocessed))

Retention Time	Area	Area %	Height	Height %
4.483	5167965	97.18	278479	98.07
6.653	149770	2.82	5487	1.93
Totals	5317735	100.00	283966	100.00

Figure S89. HPLC of compound **5**.

Compound 6

Page 1 of 1

Area % Report

Data File: C:\ChromQuest\Enterprise\Projects\Default\Data\Covid Project\CKP45_.dat
Method:
C:\ChromQuest\Enterprise\Projects\Default\Method\Covid\H2O_0.1%TFA_ACN_20_80_Isocratic.met
Acquired: 10/30/2023 2:03:56 PM
Printed: 10/31/2023 5:50:45 PM

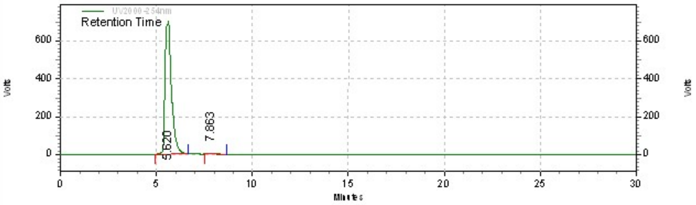

UV2000-254nm Results (System (10/31/2023 5:50:41 PM) (Reprocessed))

Retention Time	Area	Area %	Height	Height %
5.620	16335588	99.27	703101	99.44
7.863	120591	0.73	3959	0.56
Totals	16456179	100.00	707060	100.00

Figure S90. HPLC of compound **6**.

Compound 11

Page 1 of 1

Area % Report

Data File: C:\ChromQuest\Enterprise\Projects\Default\Data\Covid Project\CKP20.dat
Method:
C:\ChromQuest\Enterprise\Projects\Default\Method\Covid\H2O_0.1%TFA_ACN_20_80_Isocratic.met
Acquired: 10/26/2023 2:36:01 PM
Printed: 10/26/2023 3:31:55 PM

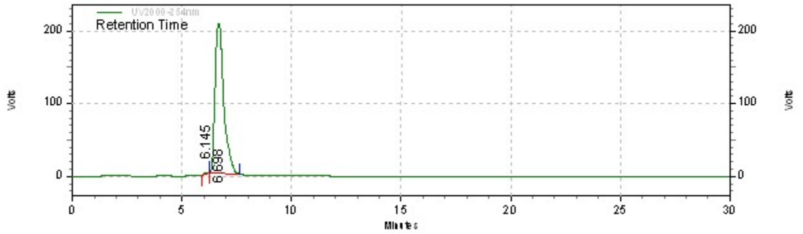

UV2000-254nm Results (System (10/26/2023 3:31:52 PM) (Reprocessed))

Retention Time	Area	Area %	Height	Height %
6.145	17736	0.31	1353	0.65
6.698	5649294	99.69	205356	99.35
Totals	5667030	100.00	206709	100.00

Figure S91. HPLC of compound **11**.

Compound 12

Page 1 of 1

Area % Report

Data File: C:\ChromQuest\Enterprise\Projects\Default\Data\Covid Project\CKP21_.dat
Method:
C:\ChromQuest\Enterprise\Projects\Default\Method\Covid\H2O_0.1%TFA_ACN_20_80_Isocratic.met
Acquired: 10/26/2023 3:38:44 PM
Printed: 10/26/2023 4:23:27 PM

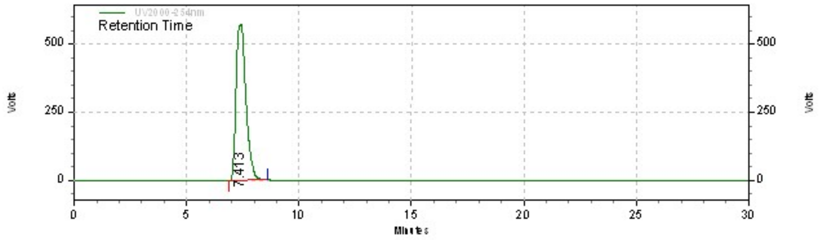

UV2000-254nm Results (System (10/26/2023 4:23:26 PM) (Reprocessed))

Retention Time	Area	Area %	Height	Height %
7.413	17527345	100.00	572896	100.00
Totals	17527345	100.00	572896	100.00

Figure S92. HPLC of compound **12**.

Compound 13

Page 1 of 1

Area % Report

Data File: C:\ChromQuest\Enterprise\Projects\Default\Data\Covid Project\CKP48.dat
Method:
C:\ChromQuest\Enterprise\Projects\Default\Method\Covid\H2O_0.1%TFA_ACN_20_80_Isocratic.met
Acquired: 10/26/2023 4:19:02 PM
Printed: 10/26/2023 4:53:17 PM

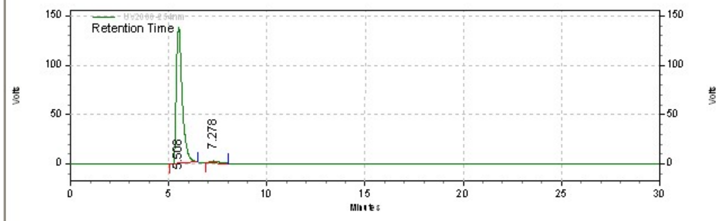

UV2000-254nm Results (System (10/26/2023 4:53:15 PM) (Reprocessed))

Retention Time	Area	Area %	Height	Height %
5.508	2881676	98.03	138029	98.65
7.278	57918	1.97	1894	1.35
Totals	2939594	100.00	139923	100.00

Figure S93. HPLC of compound **13**.

Compound 14

Page 1 of 1

Area % Report

Data File: C:\ChromQuest\Enterprise\Projects\Default\Data\Covid Project\CKP29.dat
Method:
C:\ChromQuest\Enterprise\Projects\Default\Method\Covid\H2O_0.1%TFA_ACN_20_80_Isocratic__.met
Acquired: 11/1/2023 1:57:44 PM
Printed: 11/1/2023 4:40:29 PM

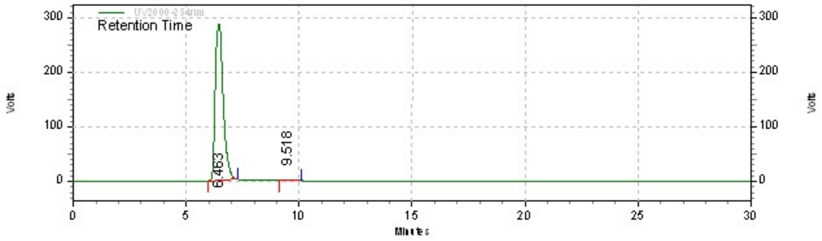

UV2000-254nm Results (System (11/1/2023 4:40:26 PM) (Reprocessed))

Retention Time	Area	Area %	Height	Height %
6.463	6984807	99.05	285845	99.27
9.518	67276	0.95	2110	0.73
Totals	7052083	100.00	287955	100.00

Figure S94. HPLC of compound **14**.

Compound 15

Page 1 of 1

Area % Report

Data File: C:\ChromQuest\Enterprise\Projects\Default\Data\Covid Project\CKP28_.dat
Method:
C:\ChromQuest\Enterprise\Projects\Default\Method\Covid\H2O_0.1%TFA_ACN_20_80_Isocratic.met
Acquired: 10/30/2023 1:00:02 PM
Printed: 10/31/2023 5:38:18 PM

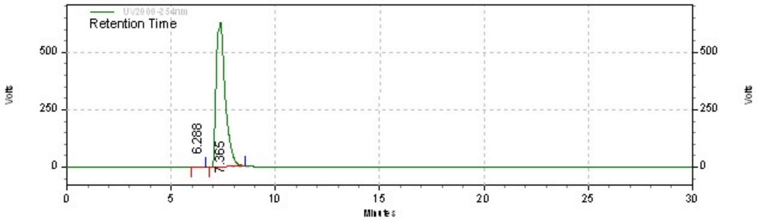

UV2000-254nm Results (System (10/31/2023 5:38:15 PM) (Reprocessed))

Retention Time	Area	Area %	Height	Height %
6.288	8752	0.05	385	0.06
7.365	18887090	99.95	628596	99.94
Totals	18895842	100.00	628981	100.00

Figure S95. HPLC of compound **15**.

Compound 16

Page 1 of 1

Area % Report

Data File: C:\ChromQuest\Enterprise\Projects\Default\Data\Covid Project\CKP49.dat
Method:
C:\ChromQuest\Enterprise\Projects\Default\Method\Covid\H2O_0.1%TFA_ACN_20_80_Isocratic.met
Acquired: 10/26/2023 4:50:12 PM
Printed: 10/26/2023 5:30:23 PM

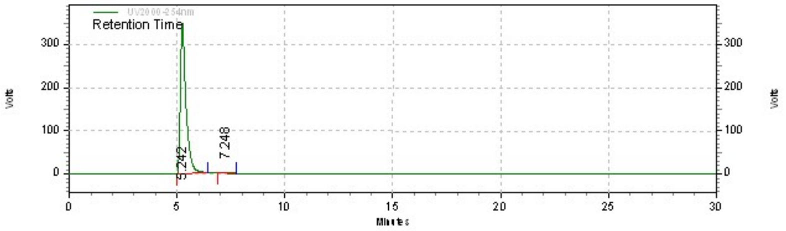

UV2000-254nm Results (System (10/26/2023 5:30:21 PM) (Reprocessed))

Retention Time	Area	Area %	Height	Height %
5.242	6283685	99.37	348873	99.63
7.248	39646	0.63	1313	0.37
Totals	6323331	100.00	350186	100.00

Figure S96. HPLC of compound **16**.

Compound 28

Page 1 of 1

Area % Report

Data File: C:\ChromQuest\Enterprise\Projects\Default\Data\Covid Project\CKP25_251023_TFA.dat
Method:
C:\ChromQuest\Enterprise\Projects\Default\Method\Covid\H2O_0.1%TFA_ACN_20_80_Isocratic.met
Acquired: 10/25/2023 2:47:13 PM
Printed: 10/26/2023 12:51:13 PM

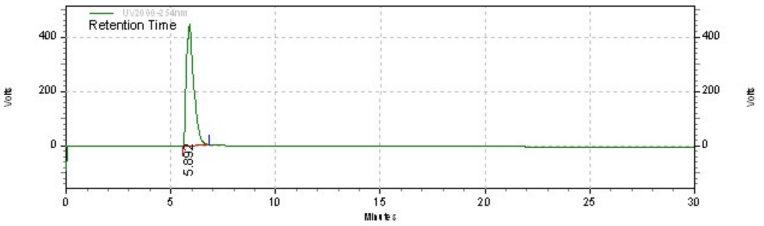

UV2000-254nm Results (System (10/25/2023 3:52:06 PM) (Reprocessed))

Retention Time	Area	Area %	Height	Height %
5.892	10806884	100.00	447104	100.00
Totals	10806884	100.00	447104	100.00

Figure S97. HPLC of compound **28**.

Compound 29

Page 1 of 1

Area % Report

Data File: C:\ChromQuest\Enterprise\Projects\Default\Data\Covid Project\CKP27_1.dat
Method:
C:\ChromQuest\Enterprise\Projects\Default\Method\Covid\H2O_0.1%TFA_ACN_20_80_Isocratic.met
Acquired: 10/25/2023 4:00:22 PM
Printed: 10/26/2023 3:40:36 PM

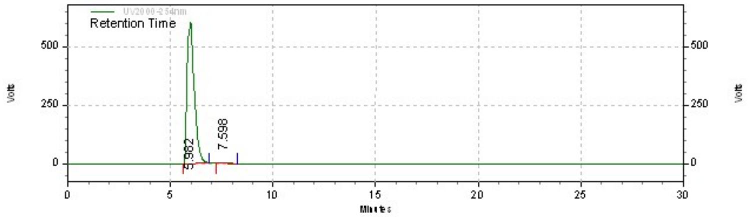

UV2000-254nm Results (System (10/26/2023 3:40:33 PM) (Reprocessed))

Retention Time	Area	Area %	Height	Height %
5.982	14636128	99.25	602166	99.37
7.598	110597	0.75	3792	0.63
Totals	14746725	100.00	605958	100.00

Figure S98. HPLC of compound **29**.

Compound 30

Page 1 of 1

Area % Report

Data File: C:\ChromQuest\Enterprise\Projects\Default\Data\Covid Project\CKP44_1.dat
Method:
C:\ChromQuest\Enterprise\Projects\Default\Method\Covid\H2O_0.1%TFA_ACN_20_80_Isocratic.met
Acquired: 10/25/2023 5:27:03 PM
Printed: 10/26/2023 5:32:16 PM

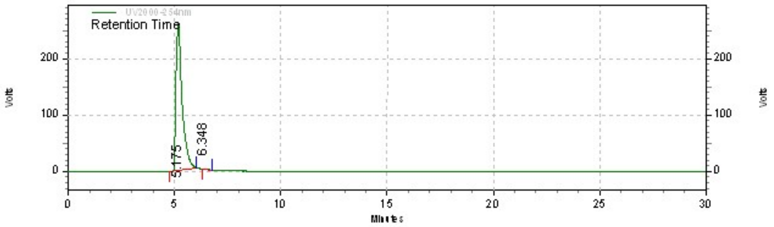

UV2000-254nm Results (System (10/25/2023 6:00:12 PM) (Reprocessed))

Retention Time	Area	Area %	Height	Height %
5.175	5233713	99.84	260697	99.88
6.348	8538	0.16	317	0.12
Totals	5242251	100.00	261014	100.00

Figure S99. HPLC of compound **30**.

Compound 31

Page 1 of 1

Area % Report

Data File: C:\ChromQuest\Enterprise\Projects\Default\Data\Covid Project\CKP26_1.dat
Method:
C:\ChromQuest\Enterprise\Projects\Default\Method\Covid\H2O_0.1%TFA_ACN_20_80_Isocratic.met
Acquired: 10/26/2023 12:23:28 PM
Printed: 10/26/2023 12:59:25 PM

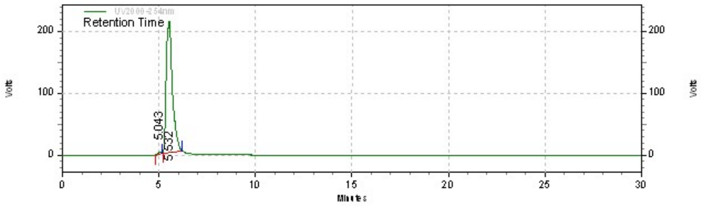

UV2000-254nm Results (System (10/26/2023 12:59:21 PM) (Reprocessed))

Retention Time	Area	Area %	Height	Height %
5.043	29090	0.62	2450	1.15
5.532	4684877	99.38	211444	98.85
Totals	4713967	100.00	213894	100.00

Figure S100. HPLC of compound **31**.

Compound 32

Page 1 of 1

Area % Report

Data File: C:\ChromQuest\Enterprise\Projects\Default\Data\Covid Project\CKP30.dat
Method:
C:\ChromQuest\Enterprise\Projects\Default\Method\Covid\H2O_0.1%TFA_ACN_20_80_Isocratic.met
Acquired: 10/26/2023 1:08:29 PM
Printed: 10/26/2023 3:44:17 PM

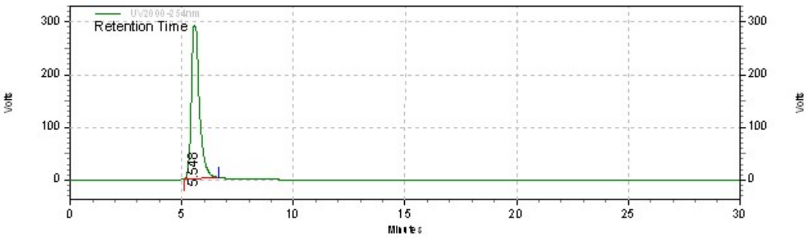

UV2000-254nm Results (System (10/26/2023 3:43:59 PM) (Reprocessed))

Retention Time	Area	Area %	Height	Height %
5.548	7357293	100.00	291779	100.00
Totals	7357293	100.00	291779	100.00

Figure S101. HPLC of compound **32**.

Compound 33

Page 1 of 1

Area % Report

Data File: C:\ChromQuest\Enterprise\Projects\Default\Data\Covid Project\CKP46.dat
Method:
C:\ChromQuest\Enterprise\Projects\Default\Method\Covid\H2O_0.1%TFA_ACN_20_80_Isocratic.met
Acquired: 10/30/2023 5:06:10 PM
Printed: 10/31/2023 5:53:11 PM

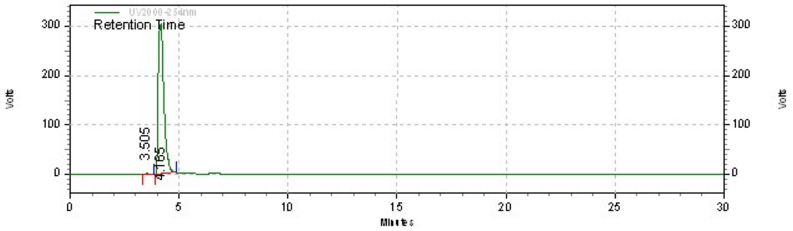

UV2000-254nm Results (System (10/31/2023 5:53:08 PM) (Reprocessed))

Retention Time	Area	Area %	Height	Height %
3.505	21085	0.39	1525	0.50
4.165	5369981	99.61	303581	99.50
Totals	5391066	100.00	305106	100.00

Figure S102. HPLC of compound **33**.

Compound 35

Page 1 of 1

Area % Report

Data File: C:\ChromQuest\Enterprise\Projects\Default\Data\Covid Project\CKP36__.dat
Method:
C:\ChromQuest\Enterprise\Projects\Default\Method\Covid\H2O_0.1%TFA_ACN_20_80_Isocratic.met
Acquired: 2/13/2024 6:36:50 PM
Printed: 2/13/2024 7:11:30 PM

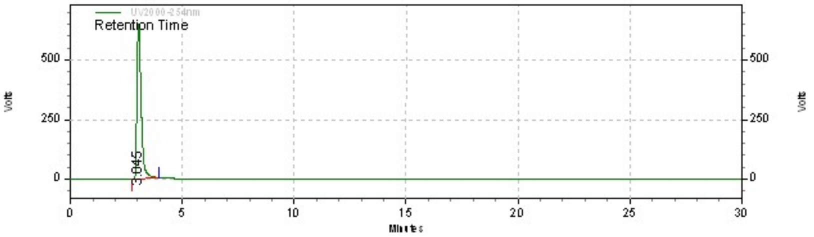

UV2000-254nm Results (System (2/13/2024 7:11:25 PM) (Reprocessed))

Retention Time	Area	Area %	Height	Height %
3.045	8654473	100.00	651293	100.00
Totals	8654473	100.00	651293	100.00

Figure S103. HPLC of compound **35**.

Compound 37

Page 1 of 1

Area % Report

Data File: C:\ChromQuest\Enterprise\Projects\Default\Data\Covid Project\CKP35.dat
Method:
C:\ChromQuest\Enterprise\Projects\Default\Method\Covid\H2O_0.1%TFA_ACN_20_80_Isocratic.met
Acquired: 10/30/2023 4:34:23 PM
Printed: 10/31/2023 6:04:11 PM

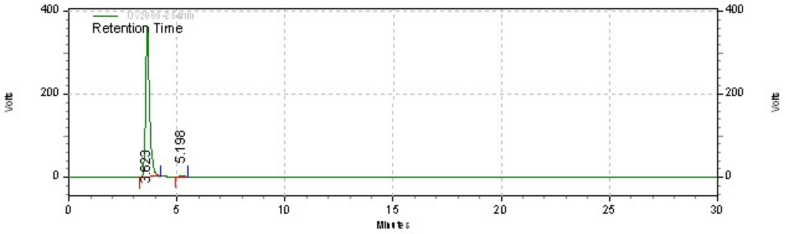

UV2000-254nm Results (System (10/31/2023 6:04:09 PM) (Reprocessed))

Retention Time	Area	Area %	Height	Height %
3.623	4641126	99.56	360922	99.68
5.198	20467	0.44	1167	0.32
Totals	4661593	100.00	362089	100.00

Figure S104. HPLC of compound **37**.

Compound 38

Page 1 of 1

Area % Report

Data File: C:\ChromQuest\Enterprise\Projects\Default\Data\Covid Project\CKP31.dat
Method:
C:\ChromQuest\Enterprise\Projects\Default\Method\Covid\H2O_0.1%TFA_ACN_20_80_Isocratic.met
Acquired: 10/31/2023 11:26:20 AM
Printed: 11/1/2023 11:51:49 AM

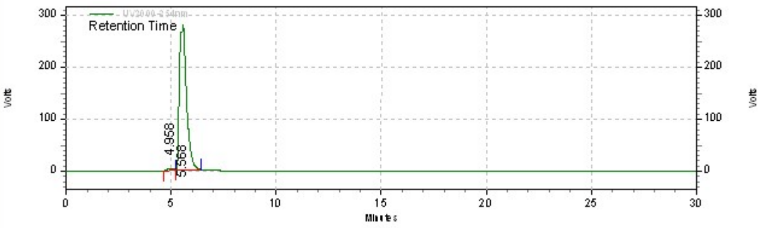

UV2000-254nm Results (System (11/1/2023 11:51:44 AM) (Reprocessed))

Retention Time	Area	Area %	Height	Height %
4.958	69349	1.05	3810	1.35
5.568	6509647	98.95	279285	98.65
Totals	6578996	100.00	283095	100.00

Figure S105. HPLC of compound **38**.

Compound 39

Page 1 of 1

Area % Report

Data File: C:\ChromQuest\Enterprise\Projects\Default\Data\Covid Project\CKP32_.dat
Method:
C:\ChromQuest\Enterprise\Projects\Default\Method\Covid\H2O_0.1%TFA_ACN_20_80_Isocratic.met
Acquired: 10/31/2023 12:24:49 PM
Printed: 11/1/2023 11:54:33 AM

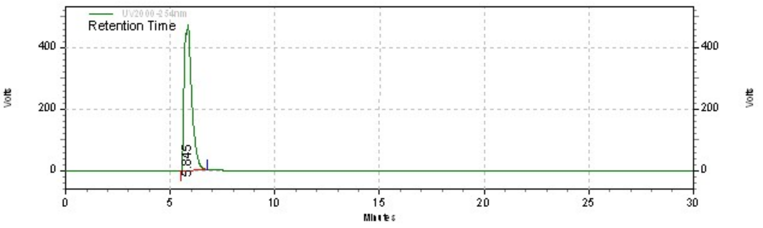

UV2000-254nm Results (System (11/1/2023 11:54:29 AM) (Reprocessed))

Retention Time	Area	Area %	Height	Height %
5.845	11831939	100.00	473833	100.00
Totals	11831939	100.00	473833	100.00

Figure S106. HPLC of compound **39**.

Compound 40

Page 1 of 1

Area % Report

Data File: C:\ChromQuest\Enterprise\Projects\Default\Data\Covid Project\CKP47.dat
Method:
C:\ChromQuest\Enterprise\Projects\Default\Method\Covid\H2O_0.1%TFA_ACN_20_80_Isocratic.met
Acquired: 10/31/2023 2:28:39 PM
Printed: 11/1/2023 12:15:48 PM

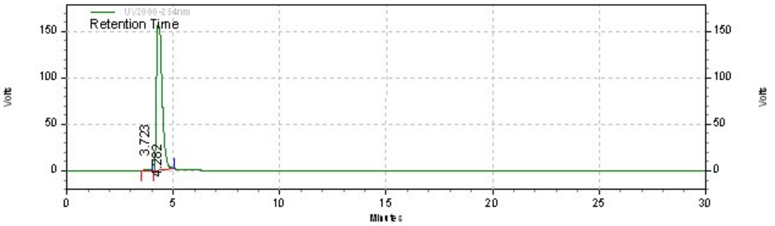

UV2000-254nm Results (System (11/1/2023 12:15:44 PM) (Reprocessed))

Retention Time	Area	Area %	Height	Height %
3.723	14664	0.50	1018	0.64
4.282	2946552	99.50	158576	99.36
Totals	2961216	100.00	159594	100.00

Figure S107. HPLC of compound **40**.

Compound 41

Page 1 of 1

Area % Report

Data File: C:\ChromQuest\Enterprise\Projects\Default\Data\Covid Project\CKP33.dat
Method:
C:\ChromQuest\Enterprise\Projects\Default\Method\Covid\H2O_0.1%TFA_ACN_20_80_Isocratic.met
Acquired: 10/31/2023 1:08:42 PM
Printed: 11/1/2023 11:58:20 AM

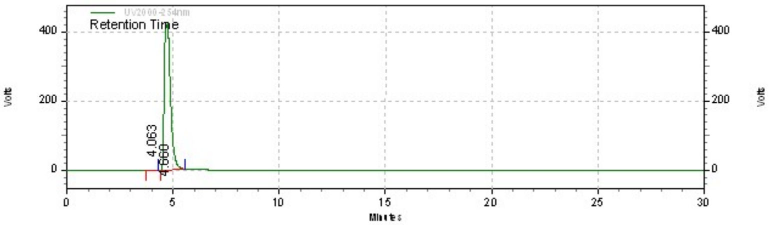

UV2000-254nm Results (System (11/1/2023 11:58:13 AM) (Reprocessed))

Retention Time	Area	Area %	Height	Height %
4.063	14150	0.16	1078	0.25
4.660	8653336	99.84	423063	99.75
Totals	8667486	100.00	424141	100.00

Figure S108. HPLC of compound **41**.

Compound 42

Page 1 of 1

Area % Report

Data File: C:\ChromQuest\Enterprise\Projects\Default\Data\Covid Project\CKP34.dat
Method:
C:\ChromQuest\Enterprise\Projects\Default\Method\Covid\H2O_0.1%TFA_ACN_20_80_Isocratic.met
Acquired: 10/31/2023 1:56:31 PM
Printed: 11/1/2023 12:00:47 PM

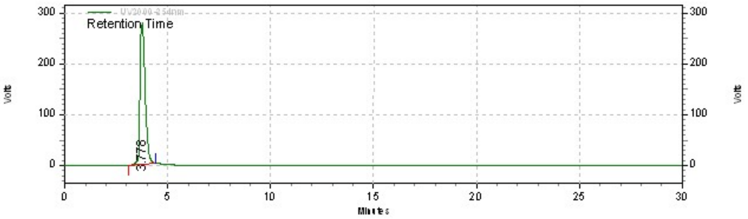

UV2000-254nm Results (System (11/1/2023 12:00:41 PM) (Reprocessed))

Retention Time	Area	Area %	Height	Height %
3.778	4835475	100.00	278789	100.00
Totals	4835475	100.00	278789	100.00

Figure S109. HPLC of compound **42**.

Compound 43

Page 1 of 1

Area % Report

Data File: C:\ChromQuest\Enterprise\Projects\Default\Data\Covid Project\CKP42_.dat
Method:
C:\ChromQuest\Enterprise\Projects\Default\Method\Covid\H2O_0.1%TFA_ACN_20_80_Isocratic.met
Acquired: 10/31/2023 4:44:26 PM
Printed: 11/1/2023 12:09:55 PM

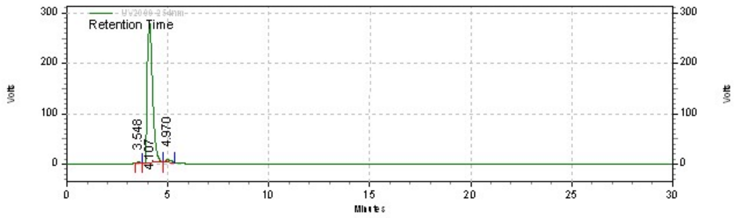

UV2000-254nm Results (System (11/1/2023 12:09:51 PM) (Reprocessed))

Retention Time	Area	Area %	Height	Height %
3.548	13872	0.27	1237	0.44
4.107	5032663	97.85	275829	97.65
4.970	96700	1.88	5397	1.91
Totals	5143235	100.00	282463	100.00

Figure S110. HPLC of compound **43**.

Compound 44

Page 1 of 1

Area % Report

Data File: C:\ChromQuest\Enterprise\Projects\Default\Data\Covid Project\CKP43.dat
Method:
C:\ChromQuest\Enterprise\Projects\Default\Method\Covid\H2O_0.1%TFA_ACN_20_80_Isocratic.met
Acquired: 10/31/2023 3:48:17 PM
Printed: 11/1/2023 12:13:28 PM

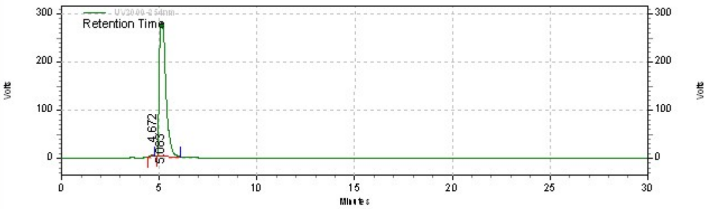

UV2000-254nm Results (System (11/1/2023 12:13:25 PM) (Reprocessed))

Retention Time	Area	Area %	Height	Height %
4.672	37987	0.58	3107	1.11
5.083	6549200	99.42	278000	98.89
Totals	6587187	100.00	281107	100.00

Figure S111. HPLC of compound **44**.

Compound 45

Page 1 of 1

Area % Report

Data File: C:\ChromQuest\Enterprise\Projects\Default\Data\Covid Project\CKP40.dat
Method:
C:\ChromQuest\Enterprise\Projects\Default\Method\Covid\H2O_0.1%TFA_ACN_20_80_Isocratic.met
Acquired: 10/31/2023 3:00:09 PM
Printed: 11/1/2023 12:04:47 PM

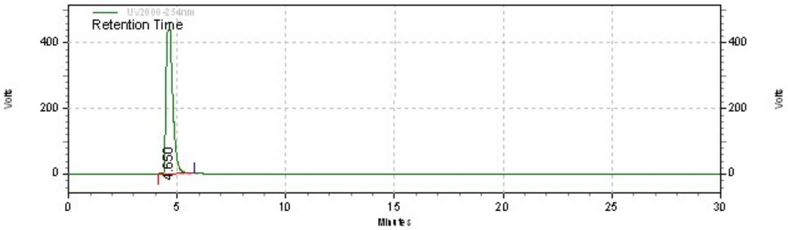

UV2000-254nm Results (System (11/1/2023 12:04:44 PM) (Reprocessed))

Retention Time	Area	Area %	Height	Height %
4.650	9451277	100.00	456574	100.00
Totals	9451277	100.00	456574	100.00

Figure S112. HPLC of compound **45**.

Compound 46

Page 1 of 1

Area % Report

Data File: C:\ChromQuest\Enterprise\Projects\Default\Data\Covid Project\CKP41_011123.dat
Method:
C:\ChromQuest\Enterprise\Projects\Default\Method\Covid\H2O_0.1%TFA_ACN_20_80_Isocratic.met
Acquired: 11/1/2023 11:50:03 AM
Printed: 11/1/2023 1:58:22 PM

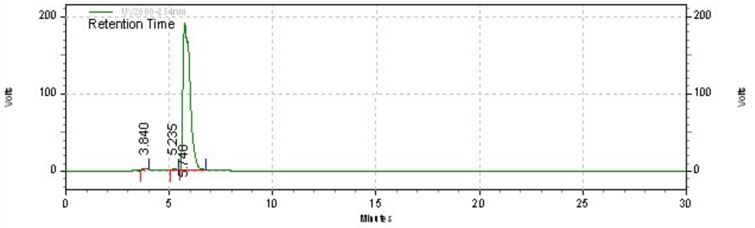

UV2000-254nm Results (System (11/1/2023 12:27:31 PM) (Reprocessed))

Retention Time	Area	Area %	Height	Height %
3.840	29085	0.64	1977	1.02
5.235	18444	0.40	1648	0.85
5.748	4511155	98.96	189861	98.13
Totals	4558684	100.00	193486	100.00

Figure S113. HPLC of compound **46**.

Supplementary Charts
Chart S1

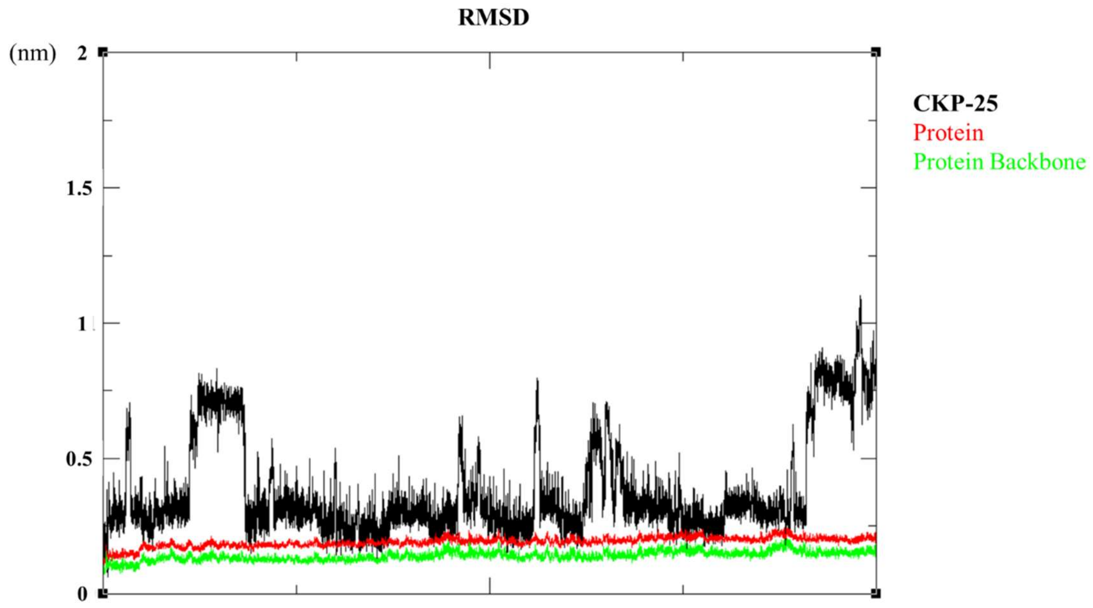

Chart S1, RMSD plot of the MD simulation results for **CKP-25**, the protein and the protein backbone with respective coloring provided.

Chart S2

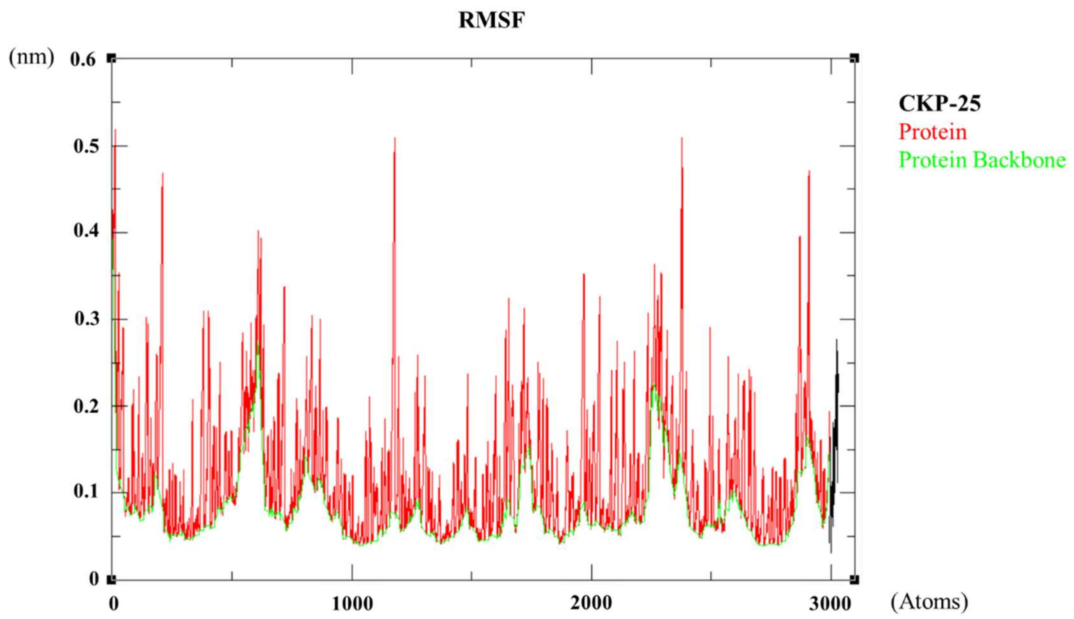

Chart S2. Atom fluctuation plot of the MD simulation results for CKP-25, the protein and the protein backbone with respective coloring provided.

Chart S3

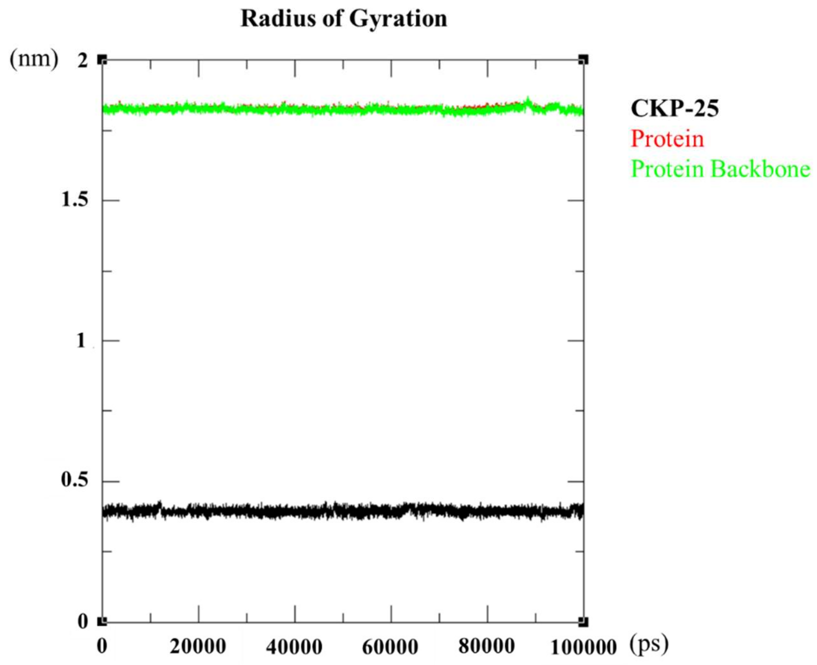

Chart S3. Radius of gyration plot of the MD simulation results for CKP-25, the protein and the protein backbone with respective coloring provided.

Chart S4

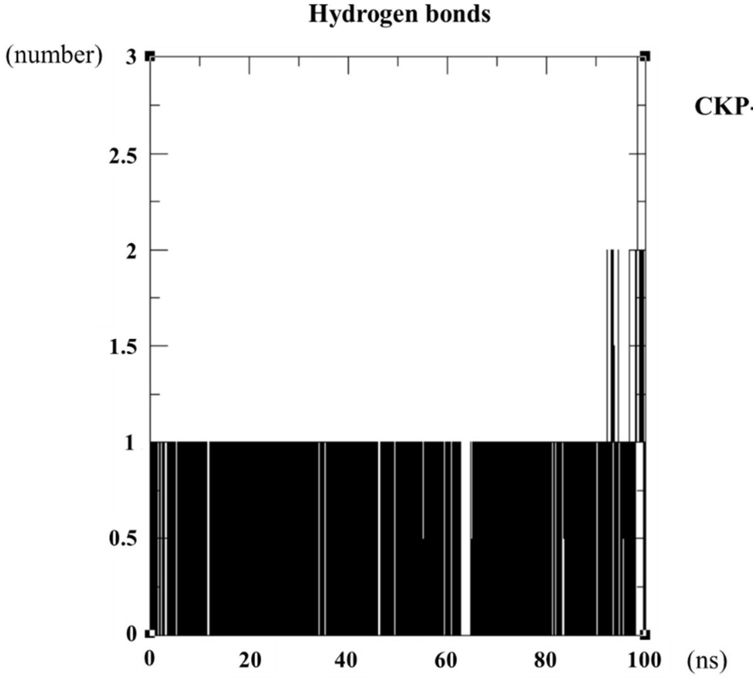

Chart S4. Number of hydrogen bonds plot for the MD simulation results regarding compound CKP-25.

References

1. Yu, B.; Zhang, H.; Zhao, Y.; Chen, S.; Xu, J.; Huanga, C.; Liu, Z. Cyclization of *o*-phenylenediamines by CO_2 in the presence of H_2 for the synthesis of benzimidazoles. *Green Chem*., **2013**, *15*, 95-99. https://doi.org/10.1039/C2GC36517K

2. De Jesús Cruz, P.; Crawford, E.T.; Liu, S.; Jeffrey S. Johnson, J.S. Stereodivergent Nucleophilic Additions to Racemic *β*-Oxo Acid Derivatives: Fast Addition Outcompetes Stereoconvergence in the Archetypal Configurationally Unstable Electrophile. *J. Am. Chem. Soc.* **2021**, *143*, 16264−16273. https://doi.org/10.1021/jacs.1c07702

3. Bouziane, A.; Hélou, M.; Carboni, B.; Carreaux, F.; Demerseman, B.; Bruneau, C.; Renaud, J-L. Ruthenium-Catalyzed Synthesis of Allylic Alcohols: Boronic Acid as a Hydroxide Source. *Chem. Eur. J.* **2008**, *14*, 5630-5637. https://doi.org/10.1002/chem.200702030

4. Guo, S.-R.; Yuan, Y.-Q. Copper-Catalyzed Alkenylation of Alcohols with β-Nitrostyrenes via a Radical Addition–Elimination Process. *Synlett* **2015**, *26*, 1961– 1968. DOI: 10.1055/s-0034-1380445

5. Spadafora, B.P.; Moreira Ribeiro, F.W.; Matsushima, J.E.; Ariga, E.M.; Omari, I.; Arruda Soares, P.M.; de Oliveira-Silva, D.; Vinhato, E.; McIndoe, J.S.; Correra, T.C.; Rodrigues, A. Regio- and diastereoselective Pd-catalyzed aminochlorocyclization of allylic carbamates: scope, derivatization, and mechanism. *Org. Biomol. Chem.*, **2021**, *19*, 5595-5606. https://doi.org/10.1039/D1OB00670C

6. Burmaoglu, S.; Yilmaz, A.O.; Taslimi, P.; Algul, O.; Kilic, D.; Gulcin, I. Synthesis and biological evaluation of phloroglucinol derivatives possessing α-glycosidase, acetylcholinesterase, butyrylcholinesterase, carbonic anhydrase inhibitory activity. *Archiv. Der Pharm*. **2018**, *351*, 1700314. https://doi.org/10.1002/ardp.201700314
